# Global assessment of small RNAs reveals a non-coding transcript involved in biofilm formation and attachment in *Acinetobacter baumannii* ATCC 17978

**DOI:** 10.1371/journal.pone.0182084

**Published:** 2017-08-01

**Authors:** Laura Álvarez-Fraga, Soraya Rumbo-Feal, Astrid Pérez, Manuel J. Gómez, Carmen Gayoso, Juan A. Vallejo, Emily J. Ohneck, Jaione Valle, Luis A. Actis, Alejandro Beceiro, Germán Bou, Margarita Poza

**Affiliations:** 1 Departamento de Microbiología, Instituto de Investigación Biomédica (INIBIC), Complejo Hospitalario Universitario (CHUAC), A Coruña, Spain; 2 Department of Microbiology, Miami University, Oxford, Ohio, United States of America; 3 Department of Molecular Evolution, Center for Astrobiology, INTA-CSIC, Torrejón de Ardoz, Madrid, Spain; 4 Centro Nacional de Investigaciones Cardiovasculares Carlos III, Madrid, Spain; 5 Departamento de Biofilms Microbianos, Instituto de Agrobiotecnología, Navarra, Spain; University of Kansas Medical Center, UNITED STATES

## Abstract

Many strains of *Acinetobacter baumannii* have been described as being able to form biofilm. Small non-coding RNAs (sRNAs) control gene expression in many regulatory circuits in bacteria. The aim of the present work was to provide a global description of the sRNAs produced both by planktonic and biofilm-associated (sessile) cells of *A*. *baumannii* ATCC 17978, and to compare the corresponding gene expression profiles to identify sRNAs molecules associated to biofilm formation and virulence. sRNA was extracted from both planktonic and sessile cells and reverse transcribed. cDNA was subjected to 454-pyrosequencing using the GS-FLX Titanium chemistry. The global analysis of the small RNA transcriptome revealed different sRNA expression patterns in planktonic and biofilm associated cells, with some of the transcripts only expressed or repressed in sessile bacteria. A total of 255 sRNAs were detected, with 185 of them differentially expressed in the different types of cells. A total of 9 sRNAs were expressed only in biofilm cells, while the expression of other 21 coding regions were repressed only in biofilm cells. Strikingly, the expression level of the sRNA 13573 was 120 times higher in biofilms than in planktonic cells, an observation that prompted us to further investigate the biological role of this non-coding transcript. Analyses of an isogenic mutant and over-expressing strains revealed that the sRNA 13573 gene is involved in biofilm formation and attachment to A549 human alveolar epithelial cells. The present work serves as a basis for future studies examining the complex regulatory network that regulate biofilm biogenesis and attachment to eukaryotic cells in *A*. *baumannii* ATCC 17978.

## Introduction

*Acinetobacter baumannii* is a non-fermentative, oxidase negative and non-flagellated Gram-negative bacillus. Although it is a normal inhabitant of the human skin flora, the intestinal tract, and the respiratory system, it has been recently described as a dangerous opportunistic pathogen [1]. *A*. *baumannii* exhibits a remarkable ability to develop antibiotic resistance that may lead to multiresistant patterns in a short time [2]. Its high genetic versatility facilitates the rapid adaptation to stressing or unfavourable conditions. In the last years, outbreaks caused by resistant strains of *A*. *baumannii* have emerged, causing serious health problems [3–11]. Many bacterial species can grow as biofilms: sessile lifestyle forms that increase protection against antimicrobial agents and host defences [12–17]. The growth of *A*. *baumannii* on mucous surfaces and medical devices, such as intravascular catheters or endotracheal intubation devices, may result in biofilm formation, increasing the risk of bloodstream and respiratory infections [16]. Biofilms are difficult to eliminate and may cause persistent and recurrent infections. The ability of *A*. *baumannii* to adhere and persist as biofilm structures onto surfaces may be the key to understanding its pathogenic mechanisms. In general, processes involved in adhesion and the subsequent formation and development of biofilms are controlled by complex regulatory networks that coordinate the expression of multiple genes [18–24]. Since biofilm formation on abiotic surfaces contributes to the unique survival pattern of *A*. *baumannii* in hospital settings, several studies have focused on the description of the proteins and genes involved in the adoption of that lifestyle. However, little is known about the involvement of small non-coding RNA transcripts (sRNAs) in controlling biofilm formation. sRNA molecules serve a wide range of regulatory functions in bacteria and modulate almost every aspect of cell metabolism [25–32]. RNA regulators have several advantages over protein regulators. They are less costly and can be produced faster than proteins, as they are shorter than most mRNAs and do not require the extra step of translation. Moreover, RNA regulators usually act at a level complementary to protein regulators, most often functioning at the post-transcriptional level as opposed to transcription factors that act before sRNAs or enzymes such as kinases or proteases that act after sRNAs. Different combinations of these proteins and RNA regulators can provide a variety of regulatory outcomes, such as extremely tight repression, an expansion in the genes regulated in response to a single signal or, conversely, an increase in the number of signals sensed by a given gene [33]. sRNAs, usually ranging from 50 to 500 nt in length, are typically encoded within intergenic regions and are independently transcribed, using their own promoter and terminator regions [30]. Although regulatory RNAs may control gene expression by a variety of mechanisms, including the modulation of transcription, translation, mRNA stability and DNA maintenance or silencing, most of the studied small RNA regulators act through base pairing with particular RNA targets, usually affecting the translation and stability of mRNAs. Moreover, many sRNAs have been described as involved in the regulation of important bacterial survival responses [30]. Although sRNAs were first discovered in bacteria many years ago, recent technical advances such as multilayered computational searches, deep sequencing or tiled microarrays, have facilitated the discovery of hundreds of potential regulatory sRNA genes [25, 34–40]. In fact, new high throughput sequencing approaches allowed the characterization of complete bacterial transcriptomes and the description of sRNAs in different microorganisms [29, 32, 41–46]. Although studies of sRNAs involved in bacterial infection are still in the early stages, certain sRNAs are related to virulence and pathogenicity in *Salmonella*, *Erwinia*, *Yersinia*, *Vibrio*, *Listeria*, *Pseudomonas*, *Shigella* and *Staphylococcus* [47–54]. Schilling *et al*. [28] detected a small RNA gene (Aar) in *A*. *baylyi* involved in the regulation of amino acid metabolism. Iron-regulated sRNAs have been associated to biofilm formation in *Aggregatibacter actinomycetemcomitans* [55]. Recently, Sharma *et al*. [56] identified novel regulatory sRNAs in *A*. *baumannii* by bioinformatic approaches and described three of them as differentially expressed in different phases of the bacterial growth curve. One of them, sRNA AbsR25, was suggested to be involved in regulating the expression of a transporter.

The aim of the present study was to use deep sequencing technologies to characterize the small RNA transcriptome of *A*. *baumannii* and to gain insight into the mechanisms behind the ability of this organism to form biofilms. The small noncoding RNA transcriptomes of both planktonic and biofilm-associated cells of *A*. *baumannii* strain ATCC 17978 were then compared to identify differences in sRNA gene expression profiles. One of these sRNA molecules was described as involved in biofilm formation and in attachment to eukaryotic cells.

## Materials and methods

### Bacterial strains and culture conditions

*A*. *baumannii* ATCC 17978 was routinely grown in Mueller-Hinton (MH) or Luria-Bertani (LB) broth or agar. *Escherichia coli* TG1 was grown in LB broth and used for cloning procedures. *E*. *coli* OP50 (*Caenorhabditis* Genetics Center) was grown in LB and used for *Caenorhabditis briggsae* virulence assays. All strains were grown at 37°C and stored at -80°C in LB containing 10% glycerol. The concentration of antibiotic used for selection of transformants was 50 μg/mL of kanamycin (Sigma-Aldrich, St. Louis, MO). For obtaining planktonic cells, a single colony of *A*. *baumannii* ATCC 17978 was isolated on LB agar and grown in 5 mL of LB broth overnight at 37°C in an orbital shaker at 180 rpm. The resulting culture was diluted 100-fold in 500 mL of LB broth in 1L-flasks and incubated under the same conditions. Optical density (OD) was evaluated at 600 nm each 30 min. Cells were harvested at exponential (OD_600nm_ = 0.4) and late stationary (OD_600nm_ = 2.0) phases of growth after inoculation. Finally, exponential and stationary cells were resuspended in RNA later reagent (Sigma-Aldrich, St. Louis, MO), frozen using liquid nitrogen and stored at -80°C until RNA extraction.

### Biofilm generation in microfermentors

Biofilm formation under flow conditions was performed as previously describe by Tormo *et al*. with some modifications [57]. Briefly, *A*. *baumannii* ATCC 17978 biofilms were obtained in the Fermentation Laboratory of the Agrobiotechnology Institute (Navarra, Spain) under a continuous flow culture system in LB broth medium, consisting of 60-mL microfermentors (Institute Pasteur, Paris, France) with a continuous aeration flow of 40 mL/h of compressed sterile air. Submerged glass slides served as growing substratum. A sample from an overnight culture of *A*. *baumannii* ATCC 17978 grown in LB broth was used to inoculate the microfermentors, which were then maintained at 37°C for 24 h. Biofilms formed on the slides were removed with a cell scraper and immediately frozen in liquid nitrogen and stored at -80°C.

### Isolation of small RNA molecules

Three independent samples of planktonic bacteria collected at the exponential and stationary growth phases and the biofilm-associated bacteria generated in the microfermentor, all grown in LB media, were reduced to powder using mortar and pestle in the presence liquid nitrogen. RNA isolation was achieved using the *mirVana miRNA Isolation kit* (Ambion) following the manufacturer’s protocols. This kit allowed the separation of a fraction enriched in small RNA molecules (sRNA). Ribosomal RNA species were removed using the Microbexpress kit (Ambion). sRNA samples were treated with DNAse I (Invitrogen), purified using phenol-chloroform and concentrated using standard ethanol precipitation. The concentration and the purity grade of the RNA samples were evaluated using a NanoDrop ND-1000 (Thermo Fisher Scientific) and the integrity and size using a Bioanalyzer 2100 and a Small RNA Analysis Kit (Agilent Technologies Inc., Germany).

### Construction of cDNA libraries

The *SOLID small RNA expression kit* (SREK, Ambion) was employed for obtaining double stranded cDNA from sRNA following the manufacturer’s instructions and using the primers listed in S1 Table. Briefly, sRNA molecules were hybridized and ligated to adaptors, reverse transcribed and treated with RNase H. Then, the second strand was obtained through amplification. Quantification integrity and size determination of samples were done using a Bioanalyzer 2100 as described above.

### Deep sequencing procedures

Three double-stranded cDNA libraries, derived from sRNA samples obtained from liquid cultures in the exponential and stationary phases of growth, as well as biofilm cells were pyrosequenced in the Roche 454 Sequencing Center (Connecticut, USA) using the GS FLX Titanium chemistry and following the manufacturer’s protocols. Raw data obtained from pyrosequencing have been deposited in the Short Read Archive database (SRA, NCBI) under accession numbers SRX591862, SRX591861 and SRX591774.

### 454 read processing and gene expression profile comparison

DNA sequences were pre-processed with cross-match (Phred-Pharp-Consed package) and TrimSeq (Emboss package), to mask and trim the linkers added by the SREK kit (S1 Table). The sequences were then aligned with cross-match against the *A*. *baumannii* ATCC 17978 chromosome and plasmids [Genbank: NC_009085, NC_009083 and NC_009084]. Alignments were combined and processed with *ad-hoc* Perl scripts to identify read-covered regions and to exclude those overlapping with known genes. The average coverage for the remaining read-covered regions was calculated for each of the growing conditions, and normalized according to the number of mapped reads for each sample, to obtain relative abundance values (RA). RA for known sRNAs (tRNAs and 5S rRNAs) were also obtained, and the lowest (that corresponding to an arginine tRNA expressed in the exponential growth sample) was used as the upper threshold to filter out candidate transcribed regions. Remaining chromosomal regions were considered to be truly expressed. To identify those that could be differentially expressed in the different growth conditions, a 2-fold change was used as threshold. Additional details about 454 read data processing can be found in Supplementary material (S1 Text).

### Real time PCR assays

To analyse the expression levels of a set of differentially expressed sRNA molecules *Taqman Micro RNA Reverse Transcription Kit* and *Taqman Universal PCR Master Mix* as well as TaqMan probes and primers were ordered from Applied Biosystems (Life Technologies). Total RNA from planktonic and biofilm cells obtained as described above was isolated using the *RNeasy mini kit* (Qiagen). 200 ng of this RNA were reverse transcribed and 2 μL of the RT mixture were used for real time PCR assays. The expression of *gyrB* was used as a constitutively expressed control. The program followed for reverse transcription consisted in 30 min at 16°C, 30 min at 42°C and 5 min at 85°C. The program followed for real-time PCR was 1 cycle of 10 min at 95°C, 45 cycles of 95°C for 15 s, 60°C for 1 min and final cycle at 40°C for 20 s. Three biological replicates were tested for each sample. Student's t-test was performed to evaluate the statistical significance of observed differences.

### Cloning of sRNA gene in an expression vector

The gene coding for sRNA 13573, which is over-expressed in biofilm cells, was PCR-amplified using ATCC 17978 genomic DNA as template using specific primers that were designed using the complete genome sequence [Genbank: NC_009085] and included *Xba*I and/or *Nco*I restriction sites (S1 Table). The amplicon was then ligated into the pETRA plasmid containing the CTXM promoter [58] and a kanamycin resistance cassette previously introduced into the *Pst*I restriction site using primers listed in S1 Table. Cloning procedures were performed in *E*. *coli* TG1. Finally, an insert-containing pETRA derivative was used to transform *A*. *baumannii* ATCC 17978 as described by Aranda *et al*. [58].

### Constructions of knock-out strain

Plasmid pMO130 [Genbank: EU862243], a suicide vector containing *xylE*, *sacB* and a kanamycin resistance marker, was used as described by Hamad *et al*. [59]. The sRNA 13573 gene was targeted for deletion. Briefly, 900–1,000 bp upstream and downstream fragments flanking the sRNA 13573 gene were cloned into the pMO130 vector using primers listed in S1 Table. The resulting plasmid was used to transform *A*. *baumannii* ATCC 17978 by electroporation. Recombinant colonies representing the first crossover event were obtained using a combination of kanamycin selection and visual detection of XylE activity following the catechol-based method. Bright yellow kanamycin resistant colonies were grown overnight in LB supplemented with 15% sucrose and then plated on the same agar medium. Second crossover events were then confirmed by PCR using primers listed in S1 Table.

### Complementation of stable knockout mutant

To complement the isogenic Δ13573 mutant derivative, the cognate wild type allele was amplified from *A*. *baumannii* ATCC 17978 genomic DNA using the primers listed in S1 Table and then cloned into the pETRA plasmid as described above [58]. The construction was used to transform the mutant strain. Transformants were selected on kanamycin-containing plates and confirmed by PCR using primers listed in S1 Table. A strain containing the empty pETRA vector was used as control.

### Adhesion to and invasion of A549 human alveolar epithelial cells

Adhesion and invasion abilities were determined following the procedure described by Gaddy *et al*. with some modifications [22]. Briefly, A549 human alveolar epithelial cells were grown in 5% CO_2_ at 37°C in Dulbecco's Modified Eagle Medium (DMEM) (Sigma-Aldrich) supplemented with 10% heat-inactivated fetal bovine serum (FBS) and 1% of penicillin-streptomycin (Gibco). Confluent monolayers were washed twice with saline solution and once with modified Hank's balanced salt solution (mHBSS, same as HBSS but without glucose). Then, A549 cells were infected with 10^5^ bacteria *per* well and incubated for 24 h in mHBSS at 37°C. To determine bacterial adhesion, the infected monolayers were washed three times with saline solution and then lysed in 500 μL of 0.5% sodium deoxycholate. To determine bacterial invasion A549 cells were infected as described above for 24 h and each well was treated for 2 h with gentamicin (256 μg/mL) before washing. Dilutions of the cell lysates were plated onto LB agar and incubated at 37°C for 24 h. Colony forming units were counted to determine the % of bacteria that had attached to or invaded A549 cells at 24 h compared to the growth control, this being static conditions and same medium without cells as previously described by Álvarez-Fraga *et al*. [60]. Four independent replicates were done. Student's t-test was performed to evaluate the statistical significance of the observed differences.

### Scanning electron microscopy (SEM) of bacterial biofilms on plastic coverslips

Overnight cultures of *A*. *baumannii* were used to inoculate 5 ml of LB in 50-ml conical tubes at a 1:100 dilution. Sterile polystyrene coverslips were placed in the inoculated 50-mL conical tubes and the tubes were incubated for 48 h at 37°C without shaking as previously described [22]. Coverslips were washed, dehydrated in ethanol, processed with a critical point drier, and sputter coated as described previously [18]. Biofilms formed above, at and below the liquid-air interface were viewed using a Zeiss Supra Gemini Series 35V scanning electron microscope as described previously [18].

### Analysis of biofilms formed on polarized A549 human alveolar cells

A549 human alveolar epithelial cells were routinely maintained as previously described [61, 62]. Costar transwell permeable support polycarbonate membrane 24 well plates (Costar Transwell Polyester Supports, Corning Inc, Corning, New York) were preconditioned 24 h prior to seeding with DMEM on both sides of the membrane and incubated at 37°C and 5% CO_2_. DMEM was removed from the conditioned transwell plates and the membranes were seeded with 10^5^ A549 cells per membrane. A549 cells were maintained submerged (DMEM on top and bottom) on the transwell membranes for one week. Following the initial week of submerged growth, DMEM was removed from the top of the membrane to allow the A549 cells to polarize and begin secreting surfactant. Cells were polarized for 2 weeks. One day prior to and for the duration of infection, A549 cells were fed DMEM supplemented with 10% heat inactivated FBS without penicillin-streptomycin (DMEM-). Bacteria, previously grown in LB at 37°C for 24 h in a shaking incubator at 180 rpm, were washed and resuspended in Hank’s Buffered Salt Solution (HBSS) (Hyclone Laboratories, Inc, Logan, Utah). A concentration of 10^2^ bacteria was applied to the apical surface of A549 cells by pipetting 1 μL of suspension onto the center of each membrane. The transwell plate was then incubated and maintained for 72 h at 37°C and 5% CO_2_. After 72 h, the membranes were washed with HBSS to remove secretions and unattached bacterial cells. The membranes were then fixed for 24 h in 4% formaldehyde-HBSS at 4°C. The membranes were then prepared for SEM using the previously described [18].

### Quantitative biofilm assay

Biofilm formation was quantified following the procedure described by Tomaras *et al*. [18], with some modifications. *A*. *baumannii* was grown on LB agar for 18 h at 37°C and used to inoculate 5 mL of LB broth. Cultures were grown at 37°C with shaking. Overnight cultures were pelleted, washed and resuspended in 5 mL of LB. A 1:100 dilution of each strain was incubated at 37°C for 12, 24 and 48 h in 15 mL polyethylene tubes. Growth culture was measured at OD_600_ to estimate total cell biomass. Biofilm formation was quantified by staining with crystal violet and solubilisation with ethanol-acetone. The OD_580_/OD_600_ ratio was used to normalize the amount of biofilm formed to the total cell content of each sample tested to avoid variations due to differences in bacterial growth under different experimental conditions. Eight independent replicates were performed. Student's t-test was performed to evaluate the statistical significance of observed differences.

## Results and discussion

### Processing of sRNA 454 sequencing reads

sRNA fractions, obtained from *A*. *baumannii* ATCC 17978 planktonic cultures in the exponential and stationary phases of growth, and biofilms were pyrosequenced. The total number of reads was 689,097, 502,152 and 627,209 for the exponential, stationary and biofilm samples, respectively (S2 Table). Between 32% and 43% of pre-processed reads could be aligned for each sample (S2 Table). The total number of filtered alignments was around twice the number of aligned reads, indicating that a significant number of reads could align to more than one location with the same score. Additional information can be found in Supplementary material (S1 Text, S1 and S2 Figs, S2, S3 and S4 Tables and S1 Dataset).

### Quantification of the expression level for known sRNA genes

Filtered alignments were processed to estimate the expression level for known sRNA genes described for *A*. *baumannii* ATCC 17978. Relative abundance (RA) values were calculated for known protein coding genes and for 16S and 23S rRNA genes (S3 Table). tRNA and 5S rRNA genes had RA average values of 1,092 to 1,338 for the three samples, which were more than 1,000-fold and about 20-fold higher than RA values calculated for protein coding genes and 16S and 23S rRNA genes, respectively (S4 Table and S1 Fig), suggesting that sRNA fractions isolated as described in Methods were significantly depleted of mRNA and rRNA. A1S_2909, coding for Leu tRNA, was the known sRNA gene expressed at the highest level, with a RA value of 18,693 in the stationary phase sample. In contrast, A1S_2764, coding for Arg tRNA, was the known sRNA gene expressed at the lowest level, with a RA value of 7.6 in the exponential phase sample. For more details see S1 Text in Supplementary files.

### Identification of new sRNA gene candidates

A total of 26,956 read-covered regions (exprRegs) were detected. To identify read-covered regions overlapping with known genes, the *ad-hoc* script FindOlappingFeatures was used to compare their coordinates with the coordinates of 3,451 known protein, tRNA and rRNA coding genes. The minimal overlap required to consider two features as overlapping was set to 0.001% of the length of any of the features. A total of 21,392 exprRegs were found to overlap with already described genes. The remaining 5,564 exprRegs (S1 Dataset) were considered as new potential sRNA genes. New sRNA gene candidates were identified both in chromosome and plasmids (5,505 and 59 genes, respectively). For more details see S1 Text in Supplementary files.

### Quantification of the expression level of sRNA gene candidates

**Fig 1:**
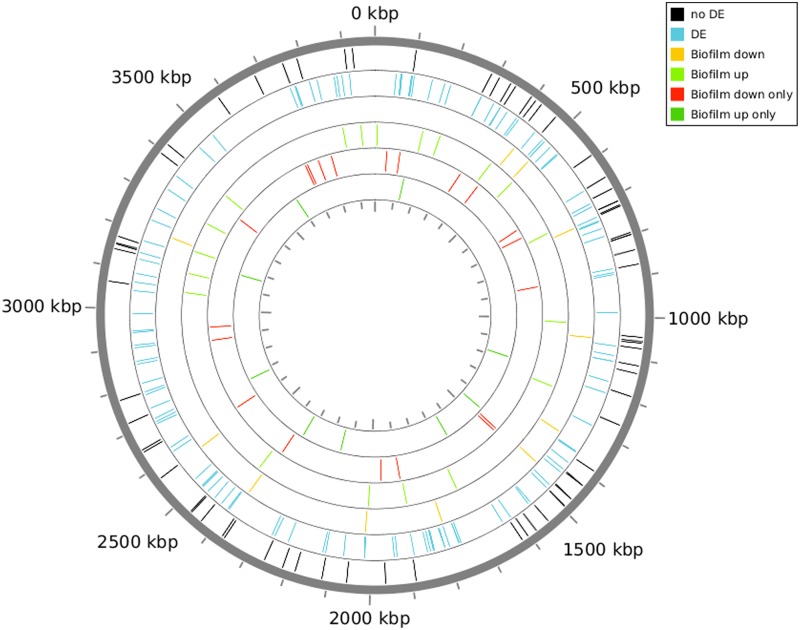
Representation of the 255 putative sRNA expression regions found by deep sequencing in the genome of the *A*. *baumannii* strain ATCC 17978. Bars indicate their location in the genome. *no DE* black bars indicate no expression differences of the sRNAs analyzed in the three growing conditions. Blue bars indicate sRNAs differentially expressed in stationary phase conditions related to exponential phase. Yellow bars indicate down regulated sRNAs in biofilm related to both types of planktonic cells. Light green bars indicate up regulated sRNAs in biofilm related to both types of planktonic cells. Red bars indicate sRNAs only expressed in planktonic cells and repressed in biofilm. Dark green bars indicate 9 sRNAs only expressed in biofilm and repressed in planktonic cells.

The set of 5,564 exprRegs that did not overlap with known protein, tRNA and rRNA coding genes had been defined by processing the combined filtered alignments of the reads from the three samples (exponential phase, stationary phase and biofilm) against the *A*. *baumannii* ATCC 17978 chromosome and plasmids. They provided a common set of candidate sRNA genes whose expression could now be quantified for each of the growing conditions. To quantify the expression of the 5,564 expRegs, the script mapAlignHits was used again in “expression mode”, using the filtered alignments obtained for each sample and the coordinate set for the 5,564 exprRegs as input. RA values were, in average, 500- to 1,000-fold lower than those calculated for known sRNAs (S5 Table). In fact, the distribution of RA values for the set of 5,564 expressed regions indicated that there were about 3,000 expRegs with RA values equal to zero for the biofilm and stationary phase samples and about 1,500 for the exponential phase sample (S2 Fig). The significant abundance of exprRegs that were not expressed in some of the three growing conditions suggested that many of them could be differentially expressed under the conditions used in this study. Since the aim of this study was to identify new sRNA genes in *A*. *baumannii* and compare their expression levels in planktonic exponential and stationary phase cells as well as in sessile bacteria, we decided to focus on the subset of sRNA gene candidates with RA values higher than that calculated for A1S_2764 (coding for tRNA-Arg), which was the lowest RA value previously observed for a known sRNA gene as described above. Accordingly, a total of 255 exprRegs were selected because they had RA values equal or higher than 7.6 in some of the three growing conditions (S2 Dataset). Fig 1 shows the genomic location of these 255 exprRegs. Out of them, 108 exprRegs fulfilled the 7.6 requirement in the RNA samples isolated from sessile bacteria, while this requirement was fulfilled in 140 and 121 exprRegs indentified in the exponential and stationary phase planktonic samples, respectively (S6 Table and S3 Fig). With the exception of exprReg_29, which was located on plasmid pAB2 (NC_009084), all of them were located in the chromosome.

This number of 255 exprReg selected varies significantly from those observed in a study done by Weiss *et al*., where 78 sRNAs were detected in *A*. *baumannii* AB5075 [63]. However, the authors evidenced that only a small number of sRNAs showed high levels of conservation amongs different *A*. *baumannii* strains, including ATCC 17978, and that many transcripts are only present either in a small subgroup of strains or exclusively in AB5075 [63]. This issue has been observed in other species such as *Pseudomonas aeruginosa*, where numerous sRNAs exhibit a strain-specific expression pattern [64, 65]. Furthermore, the set of sRNAs identified could depend on the library preparation strategy used, as it was demonstrated by Gómez-Lozano *et al*. using three different approaches to detect novel transcripts of *P*. *aeruginosa* PAO1 strain [66].

### Identification of differentially expressed sRNA genes

To identify differentially expressed regions among the set of 255 exprRegs, fold changes were calculated and an induction/repression ratio of 4 was chosen arbitrarily as threshold. A total of 185 exprRegs were classified as differentially expressed in biofilm or stationary phase conditions, relative to exponential phase (Table 1). Twenty-eight and 32 exprRegs were up or down-regulated in biofilm cells with respect to both types of planktonic cells, respectively (Fig 1). It is important to remark the high over-expression level of sRNA 13573; about 120-fold higher in biofilm with respect to planktonic cells. Nine sRNA gene candidates (expression regions 1004, 8014, 9803, 11065, 14414, 15677, 18098, 21533, and 24430) were only expressed in biofilm cells. In contrast, 21 putative sRNA genes (expression regions 29, 424, 788, 2345, 2892, 4493, 4750, 5958, 9947, 10022, 12757, 13281, 15861, 17548, 19511, 19890, 23002, 25126, 25193, 25499, and 25840) were only expressed in planktonic cells and repressed in biofilm (Table 1 and Fig 1). It is important to note that the expression region 29 was located in the plasmid pAB2 as mentioned above.

**Table 1:** List of the 185 differentially expressed coding regions.

Expression region No.	Biofilm	Exponential	Stationary
29**	1.00	14.20	3.39
165*	9.3	1.71	1.58
424**	1.00	1.97	20.67
497	1.00	11.91	1.00
598	4.24	16.09	1.00
601	4.25	12.83	1.00
602	6.37	12.42	1.00
611	3.86	16.89	1.00
786	13.85	1.42	25.22
788**	1.00	1.33	22.54
795	25.84	2.59	36.24
801	14.07	1.92	20.31
833	1.00	1.00	9.28
1004*	7.75	1.00	1.00
1136	7.52	7.68	1.69
1221*	7.78	6.59	1.00
1368	34.5	141.16	1.11
1435	2.53	1.00	12.49
1571*	13.75	1.54	1.73
2005	3.67	13.14	1.00
2006	1.56	9.58	1.00
2247	3.89	9.87	1.00
2345**	1.00	1.69	7.72
2350	36.03	60.87	5.13
2359	7.99	13.43	1.16
2492	1.00	7.71	1.00
2675	37.66	8.34	143.60
2711	1.22	8.06	1.00
2811*	8.3	1.00	2.58
2892**	1.00	8.52	1.69
2988**	1.65	8.89	3.41
3084	1.00	15.32	1.00
3275	1.00	1.00	21.31
3326	6.37	27.10	2.72
3333	4.00	7.90	1.00
3380**	1.74	8.66	29.98
3440*	15.43	2.94	1.00
3477	4.92	10.92	1.88
3641	4.65	10.11	1.00
3667	13.5	65.31	5.71
4493**	1.00	2.26	10.89
4505	1.00	8.20	1.00
4750**	1.00	1.50	9.92
4776	1.27	17.11	1.00
4846	1.00	1.00	15.24
4946*	9.91	1.38	1.00
5077	4.26	18.87	3.07
5098**	1.13	29.95	1.54
5148	1.00	1.00	7.84
5168	1.00	8.00	1.00
5286	1.00	20.55	1.00
5428	1.00	1.00	8.47
5881	298.42	484.14	15.87
5954	1.00	9.56	1.00
5958**	1.00	34.00	7.70
5985	8.67	1.00	10.16
6714	7.74	15.43	1.34
6904*	9.90	5.09	1.00
7203**	1.76	8.58	3.96
7297	1.00	1.00	7.93
7468	1.00	1.00	18.63
7562	3.82	10.67	1.00
7565	1.00	1.00	8.40
7923	1.00	1.00	31.41
8014*	8.72	1.00	1.00
8136	453.88	68.62	745.54
8382*	16.36	1.71	1.00
8776	1.00	7.65	1.00
9139**	1.32	8.64	1.39
9255	49.00	67.51	1.63
9276	2.07	1.00	8.95
9507	7.86	45.51	4.12
9656	11.25	9.21	43.47
9720	1.00	1.00	8.15
9803*	8.74	1.00	1.00
9878**	1.54	12.62	4.23
9947**	1.00	1.95	9.52
10021	1.37	1.00	17.98
10022**	1.00	1.79	9.67
10085	1.00	11.00	1.00
10453	5.99	16.68	3.05
10493	1.00	1.00	17.87
10859	1.00	1.00	18.08
11025	1.00	1.00	10.35
11065*	8.65	1.00	1.00
11473*	12.61	1.80	1.00
11798	1.00	12.00	1.00
11841	1.00	1.00	10.16
11854	3.78	7.81	1.16
12046	2.44	8.45	1.00
12048**	1.46	12.61	1.55
12150	3.07	15.33	1.00
12158	1.00	1.00	7.71
12361	1.00	1.00	13.55
12370	6.98	7.96	1.00
12371	4.40	10.40	1.34
12407	1.00	1.00	8.82
12460	1.00	1.00	22.09
12684*	10.17	4.62	1.00
12706	13.95	40.36	3.98
12757**	1.00	8.31	1.21
13053	5.16	16.31	1.00
13107	2.55	12.67	1.00
13281**	1.00	4.22	11.56
13573*	120.98	1.55	1.02
13621**	1.46	2.78	26.39
13625	1.00	1.00	7.90
13631	1.00	8.00	1.00
13953	1.00	1.00	8.47
14242	1.00	7.60	1.00
14286	1.00	9.00	1.00
14414*	17.52	1.00	1.00
14887	1.00	1.00	11.99
15147	1.00	1.00	10.16
15214	6.92	4.50	22.38
15677*	17.48	1.00	1.00
15861**	1.00	1.57	15.83
15955**	2.05	12.55	3.98
16106	2.52	11.71	1.00
16107*	10.66	2.50	1.00
16117	2.77	1.00	23.09
16257	1.00	1.00	13.55
16412	1.21	1.00	10.74
16574	1.00	11.68	1.00
16705	12.10	16.30	2.27
16723	12.16	19.32	3.89
16724	8.81	22.76	1.00
16912	1.00	1.00	10.16
17299**	1.13	9.73	1.31
17531	1.00	1.00	36.10
17548**	1.00	3.46	15.52
17642	1.00	1.00	37.26
18098*	9.58	1.00	1.00
18150	2.12	1.62	9.05
18205	19.44	35.13	1.00
18260	5.74	8.49	1.19
18451	6.23	11.35	1.54
18718	1.00	1.00	8.47
18763	1.00	1.00	8.31
18900	1.54	8.78	1.00
19276	1.00	1.00	11.62
19347	1.00	7.72	1.00
19511**	1.00	10.26	1.12
19604	6.55	17.70	1.00
19647	1.00	1.00	22.14
19890**	1.00	10.06	4.29
19898	6.21	22.87	1.00
19931	1.00	1.00	10.65
20262	1.00	11.00	1.00
20685	2.89	8.11	1.00
20762*	11.33	1.78	1.00
20837	1.00	1.00	10.49
21011	34.08	52.04	5.05
21223*	8.51	1.54	1.00
21275	1.00	1.00	8.47
21392	6.13	7.95	1.31
21533*	11.65	1.00	1.00
21637	4.54	10.68	1.00
21762*	8.08	7.45	1.00
21850**	3.14	20.00	3.20
22036	8.30	11.04	1.00
22275	1.00	1.00	15.24
22490*	7.75	1.88	1.00
22686	2.59	10.49	2.30
22981	1.00	1.00	11.08
23002**	1.00	1.14	8.00
23283*	12.19	9.52	1.00
23620	1.00	1.00	13.55
23869	1.00	11.00	1.00
24430*	8.30	1.00	1.00
25126**	1.00	1.21	11.90
25193**	1.00	9.98	1.18
25478	4.26	16.55	2.78
25499**	1.00	1.88	13.81
25551	18.69	3.92	46.59
25571	14.09	6.32	70.99
25811	1.96	1.00	10.16
25840**	1.00	6.00	8.36
25942	1.00	1.00	8.16
26235	1.00	1.00	8.47
26247*	17.86	16.66	3.39
26276	2.62	12.32	1.22
26388	1.88	9.77	1.38
26511	1.00	8.00	1.00
26627*	15.83	2.06	3.82

Differentially expressed coding regions assessed by 454 pyrosequencing corresponding to sRNA molecules and their normalized expression values in sessile (biofilm) and planktonic cells (in exponential and stationary phase of growth).

*sRNA molecules up-regulated in biofilmrelated to both planktonic cell types.

**sRNA molecules down-regulated in biofilm related to both planktonic cell types.

Coding regions only expressed or repressed in biofilm are shown in bold.

In addition, qRT-PCR analysis of sRNA 13573 and a group of six sRNA only expressed in biofilm cells using proper Taqman probes (S4 Fig) validated the differential production of these non-coding transcripts detected by pyrosequencing as described above.

Sharma *et al*. [56] described 31 predicted sRNAs coding regions in the genome of *A*. *baumannii* ATCC 17978 using computational approaches. A group of 18 of these 31 predicted sRNAs were also detected as candidate sRNAs in the present work as showed in S1 Dataset. Furthermore, these authors analyzed 10 of the 31 predicted sRNAs by northern blotting of total RNA isolated from *A*. *baumannii* MTCC1425 (ATCC 15308) cells at different stages of the growth cycle. This analysis showed that, although sRNAs AbsR11 and AbsR28 had almost the same expression level during all phases of planktonic growth, sRNA AbsR25 had no expression in the lag phase and a maximum expression in the exponential phase [56]. The AbsR25 and AbsR28 small transcripts were described in the present work as expression regions, as shown in S1 Dataset, which correspond to exprRegs 10452 and 22397. However, we did not find them as differentially expressed in the conditions analyzed. Moreover, the length of the sequences of these two regions in the ATCC 17978 strain described in the present work and in the MTCC1425 strain described by Sharma *et al*. [56] does not match completely. These variations could be due to genomic differences between these two strains or even to the different bioinformatic approaches used for prediction and analyses of these coding regions. This fact could partly explain the gene expression differences found between Sharma *et al*.’s work and the present study.

Taken together, all these observations strongly indicate that the production of a relatively large number of sRNA is significantly affected when *A*. *baumannii* cells grow as planktonic or sessile cells. Furthermore, the type and amount of particular sRNA are significantly affected by the growth stage of planktonic bacteria. Thus, sRNA transcripts could play a critical role in the physiology of *A*. *baumannii* by mechanisms that are poorly understood.

### Involvement of sRNA 13573 in biofilm formation

**Fig 2:**
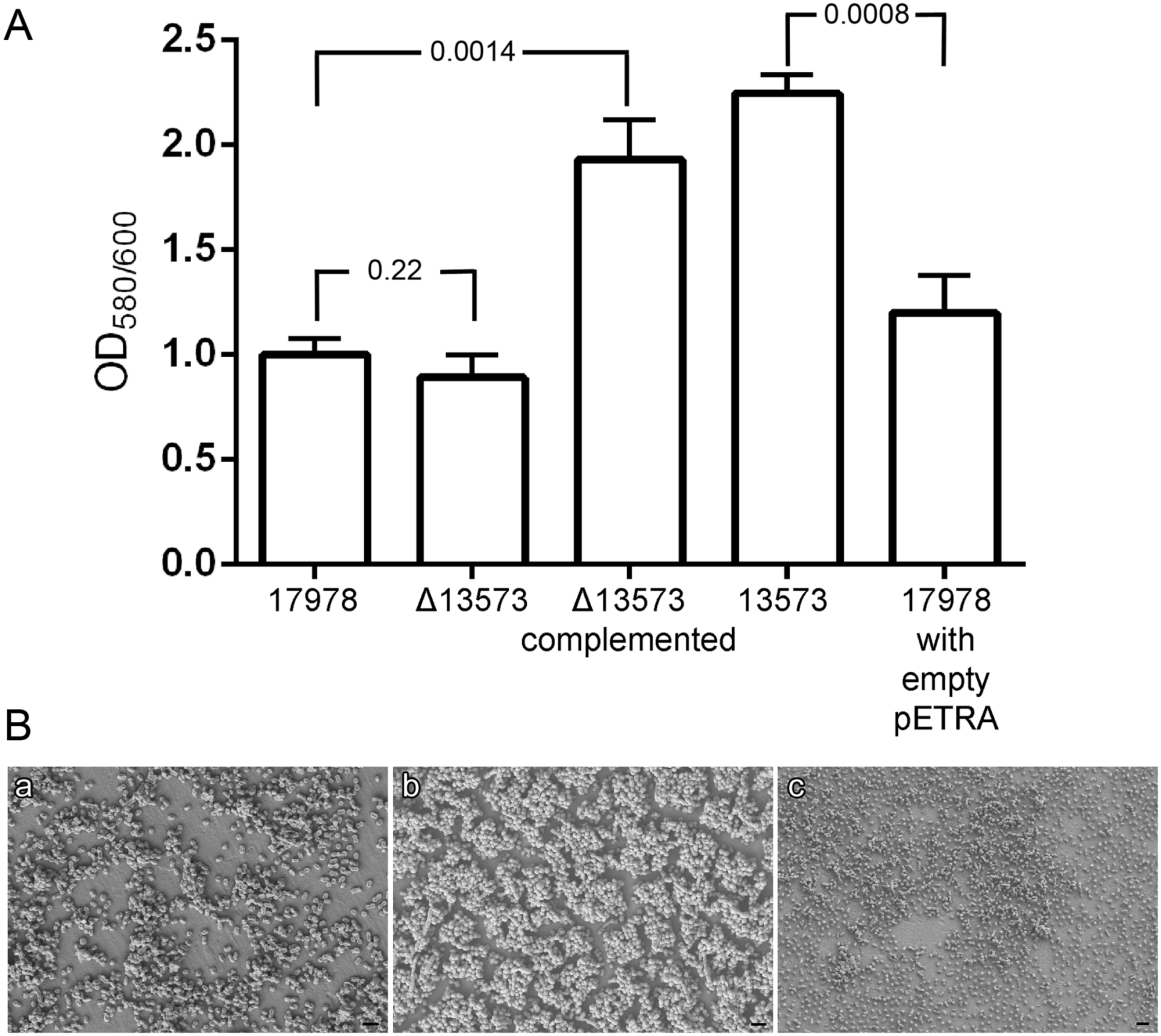
Biofilm formation assays. A) Quantification of biofilm formation by *A*. *baumannii* ATCC 17978 (17978), *A*. *baumannii* ATCC 17978 Δ13573 (Δ13573), *A*. *baumannii* ATCC 17978 harbouring pETRA (17978 with empty pETRA), *A*. *baumannii* ATCC 17978 harbouring pETRA with sRNA 13575 (13573), and *A*. *baumannii* ATCC Δ13573 harbouring pETRA with sRNA 13575 (Δ13573 complemented). B) SEM analysis of *A*. *baumannii* ATCC 17978 (a), *A*. *baumannii* sRNA 13573 over-producing strain (b) and *A*. *baumannii* ATCC 17978 Δ13573 (c). Micrographs were taken at 5,000x and bars indicate the scale marks (2 μm).

Special attention was paid to sRNA 13573 due to its high level of expression, *ca*. 120-fold in biofilm cells compared to planktonic cells as assessed by pyrosequencing. Thus, sRNA 13573 was first cloned into the pETRA vector under the control of the CTXM promoter using primers listed in S1 Table. This approach resulted in the over-expression of this coding region as confirmed by qRT-PCR (Table 2). The transformant colonies were grown and their ability to form biofilm was quantified using the wild type strain harbouring the empty pETRA vector as a control. Since the highest rate of biofilm formation was assessed at 48 h, the comparison between the different isogenic strains was perfomed at 48 h (Fig 2A, S5 Fig). Cells over-expressing sRNA 13573 (13573) showed the highest value of biofilm formation; about 2-fold compared to the strain containing the empty vector (*P* value 0.0008).

**Table 2:** Expression level of the sRNA 13573 coding region.

Strain	Expression level of sRNA 13573
17978	0.86 ± 0.19
Δ13573	0
17978 with empty pETRA	0.47 ± 0.23
13573	151.45 ± 50.84
Δ13573 complemented	151.70 ± 15.98

Expression level of the sRNA 1373 was determined by qRT-PCR using Taqman probes of the strains: wild type *A*. *baumannii* (17978), *A*. *baumannii* ATCC 17978 without sRNA 13573 (Δ13573), *A*. *baumannii* ATCC 17978 harbouring pETRA (ATCC with empty pETRA), *A*. *baumannii* ATCC 17978 harbouring pETRA with over-expressed sRNA 13575 (13573) and *A*. *baumannii* ATCC 17978 without sRNA 13573 harbouring pETRA with sRNA 13575 (Δ13573 complemented).

Also, the sRNA 13573 region was deleted from the ATCC 17978 genome and the biofilm formation ability was investigated for the corresponding knockout strain showing a no significant decrease (*P* value 0.22) in biofilm formation compared to the wild type strain as shown in Fig 2A. When a pETRA derivative containing sRNA 13573 was introduced into the knockout strain (Δ13573) the biofilm formation ability was higher (*P* value 0.0014) than that shown by the parental strain (17978). Expression level of the sRNA 13573 gene in the parental, the Δ13573, the 13573 and the control strains was confirmed by qRT-PCR (Table 2). Growth curves performed in parallel (S6 Fig) showed the same growing profile in all cases. SEM was performed for elucidating biofilm structure in *A*. *baumannii* ATCC 17978 and the strain over-expressing the sRNA 13573 or the strain lacking the 13573 sRNA (Fig 2B). SEM analysis revealed that cells over-expressing sRNA 13573 were able to develop tridimensional and organized biofilm structures on the surface of plastic coverslips while cells from both the parental and Δ13573 strains showed unorganized cells. However, cells from the Δ13573 strain showed a more disorganized pattern and more dispersion than cells from the parental strain (Fig 2B).

Little is known about the involvement of sRNA molecules in the regulation of biofilm formation and maintenance. To date, some sRNA molecules have been described as regulators involved in biofilm formation in *Pseudomonas*, *Vibrio*, *Salmonella* or *Erwinia* [67, 68]. Most of the sRNAs involved in biofilm have been described in *E*. *coli* [69] including RprA [70], McaS [71], GcvB [72] and OmrA-B [73], being OmrA-B sRNAs involved in the biosynthesis of the biofilm matrix by controlling the expression of σ^s^ or CsgD, which is in turn related to biofilm formation. Thomason *et al*. [71] described that under specific conditions *E*. *coli* over-expressing sRNA McaS increased its biofilm formation ability while it was reduced in the corresponding McaS knockout strain. Attending to the SEM analysis described in the present work, differences in biofilm formation can be appreciated between the wild type and the Δ13573 knockout strain although the crystal violet assay did not showed significant differences between both strains. This could be due to the existence of a complex regulation network where sRNA 13573 seems to play a role in biofilm formation although its deletion causes no remarkable effects. A similar effect has been shown for sRNAs RseX, CsrC or SgrS from *E*. *coli* whose over-expression increased biofilm formation while their deletion did not affect biofilm formation. Bak *et al*. [74] reported that after over-expressing and deleting 99 sRNAs in *E*. *coli*, in only a few cases the deletion of sRNA genes had effects on biofilm formation. This observation suggests that some sRNA may not be expressed highly enough to affect this cellular process under the experimental conditions used in this study. Alternatively, it is possible that there might be redundancy of sRNAs acting on the process. Furthermore, multiple sRNAs have been indentified that modulate the activity of transcriptional regulators important for biofilm formation. CsrA is a RNA-binding protein that represses biofilm formation and its activity is repressed by sRNAs CsrB and CsrC [75, 76]. Also, the transcriptional regulator CsgG, which has been shown to be required for attachment and biofilm formation in *E*. *coli* is regulated at the mRNA level by several sRNAs, including McaS, RprA or OmrA/OmrB, in response to different environmental cues [71, 73]. Thus, the effect caused by the deletion of a single sRNA could be counteracted by the action of other sRNAs.

### Involvement of the sRNA 13573 in attachment to human cells

**Fig 3:**
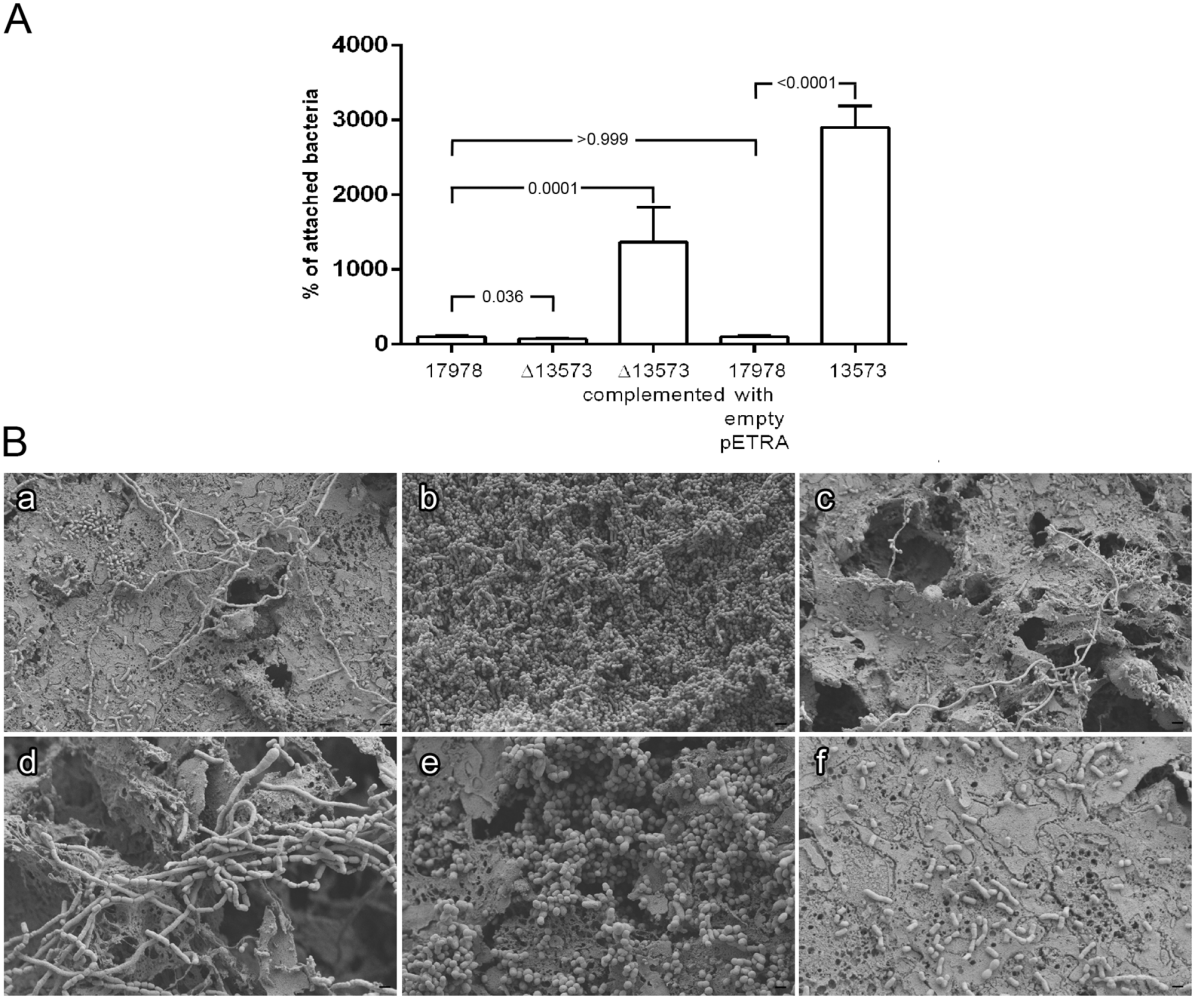
Adhesion assays. A) Attachment to A549 epithelial cells of *A*. *baumannii* wild type (17978), mutant derivative (Δ13573), mutant derivative over-expressing sRNA 13575 (Δ13573 complemented), harbouring empty pETRA vector (17978 with empty pETRA) and over-expressing sRNA 13575 (13573). The strain 17978 represents 100% of attached bacteria. B) SEM analysis of cells attached to A549 human alveolar cells of *A*. *baumannii* ATCC 17978 (a and d), *A*. *baumannii* ATCC 17978 over-expressing sRNA 13575 (b and e) and *A*. *baumannii* Δ13573 (c and f). Micrographs were taken at 5,000x (a, b and c) and 10,000x magnification (d, e and f). Bars indicate the scale marks (2 and 1 μm).

Bacterial adhesion to A549 human alveolar cells was measured for the sRNA Δ13573 strain compared with the strain over-expressing sRNA 13573 from pETRA and using i) the 17978 strain, ii) the 17978 strain containing the empty pETRA and iii) the Δ13573 strain harbouring pETRA containing sRNA 13573, as controls. As shown in Fig 3A and considering the 17978 as the sample representing 100% of attached cells, the 17978 strain over-expressing 13573 (13573) attached more (30-fold) to A549 alveolar epithelial cells than the wild type strain harbouring the empty pETRA plasmid (*P* value < 0.0001). Once more, the 13573 strain revealed a significant phenotypic change when over-expressed, showing an increase in attachment to human cells ability. Besides, an adhesion ability decrease in the Δ13573 strain was detected when compared to the wild type strain being this difference significant (*P* value 0.036). The Δ13573 complemented derivative reached a higher (13-fold) value than the wild type strain (*P* value 0.0001). Furthermore, no colonies were detected when invasiveness was checked for all the strains, indicating that all the bacterial counts obtained after a 24 h-incubation were due to attachment (data not shown). Overall data suggest that the deletion of the sRNA 13573 produces a slight but significant change in the adhesion to A549 human epithelial cells and that its over-expression clearly increases attachment abilities.

The SEM analysis of polarized A549 alveolar epithelial cells co-incubated with bacteria revealed that bacteria over-expressing sRNA 13573 were able to form a thick and tridimensional biofilm (micrographs b and e, Fig 3B) over the A549 cell layer while the wild type showed poor biofilm formation ability (micrographs a and d, Fig 3B). Accordingly to previous results, the capacity of the Δ13573 strain to attach to eukaryotic cells was remarkably lower than the wild type strain (micrographs c and f, Fig 3B).

Little is known about the role that sRNAs can play in the adhesion to eukaryotic cells. For example, sRNA FasX from *Streptococcus* inhibited the expression of a cell surface pilus, therefore reducing the ability of the bacteria to adhere to host cells [77]. Eijkelkamp *et al*. [78] compared the ability to form biofilms and to adhere to eukaryotic cells of different strains and reported that there is not a clear relationship between these two capacities. Furthermore, previous data revealed that while the truncation of a gene as *csuE* caused a decrease in biofilm formation, the resulting mutant strain was able to adhere more to bronchial epithelial cells [79]. Taken together, these results suggest that there is no direct correlation between biofilm formation on abiotic and biotic surfaces, and that there is wide variation in the cell-surface and cell-cell interactions that result in adherence and biofilm formation by different *A*. *baumannii* clinical isolates [80]. However, the 13573 sRNA here described showed to be involved in both adhesion and biofilm abilities, being both functions widely related with the pathogenesis of *A*. *baumannii* [60].

## Conclusions

The global analysis of the small RNA transcriptome of *A*. *baumannii* ATCC 17978 revealed different sRNA expression patterns in planktonic and biofilm associated cells, with some of the transcripts only expressed or repressed in sessile bacteria. *In vitro* and *in vivo* results demonstrated that the sRNA 13573 is involved in the control of the sessile lifestyle adoption and attachment to eukaryotic cells. The present work serves as a basis for future studies examining the complex regulatory network that regulate biofilm biogenesis and adhesion properties in *A*. *baumannii* ATCC 17978.

## Supporting information

S1 Text Additional details about 454 read data processing. (DOCX)

S1 Table Oligonucleotides and probes used in the present study. (DOCX)

S2 Table Number of initial reads, mapped reads and alignment hits for each sample. (DOCX)

S3 Table *A*. *baumannii* known sRNA and non-sRNA genes used as reference. (DOCX)

S4 Table Average, standard deviation, maximal and minimal values for the normalized expression scores calculated for protein coding genes, 16S and 23S rRNA genes and known sRNA genes, in each of the growing conditions. (DOCX)

S5 Table Average, standard deviation, maximal and minimal values for the normalized expression scores calculated for 5564 expressed regions not overlapping with known genes. (DOCX)

S6 Table Sets of expression regions not overlapping with known genes and having a normalized expression score equal or higher than 7.6 in some of the three growing conditions. (DOCX)

S1 Dataset Expressed regions. List of 5,564 expressed regions, their length and locations in the genome (NC_009085.1) or plasmids (NC_009083.1 and NC_009084.1) of *A*. *baumannii* ATCC 17978.
(DOCX)

S2 Dataset Coding regions. List of normalized expression of 255 coding regions (expression score should be equal or above 7.6 in some growing condition: Bio, Exp or Sta), their length and locations in the genome (NC_009085.1) or plasmid (NC_009084.1) of *A*. *baumannii* ATCC 17978.
(DOCX)

S1 Fig Distribution of normalized expression scores for known sRNA genes. Blue: biofilm samples. Orange: exponential phase samples. Yellow: stationary phase samples.
(DOCX)

S2 Fig Distribution of normalized expression scores for the 5564 expressed regions not overlapping with known genes. Blue: biofilm samples. Orange: exponential phase samples. Yellow: stationary phase samples.
(DOCX)

S3 Fig Distribution of normalized expression scores for the 255 expressed regions not overlapping with known genes and having a normalized expression score equal or higher than 7.6 in some of the growing conditions. Blue: biofilm samples. Orange: exponential phase samples. Yellow: stationary phase samples.
(DOCX)

S4 Fig qRT-PCR assays. Expression levels of 8 sRNA regions in planktonic and sessile cells determined by qRT-PCR using Taqman probes. Y axis represents the relative expression of the genes taking the housekeeping gene *gyrB* as value 1.
(DOCX)

S5 Fig Biofilm formation at different times. Staining at 12, 24 and 48 h of biofilm formation of *A*. *baumannii* ATCC 17978 (17978), *A*. *baumannii* ATCC 17978 Δ13573 (Δ13573), *A*. *baumannii* ATCC Δ13573 harbouring pETRA with sRNA 13575 (Δ13573 complemented), *A*. *baumannii* ATCC 17978 harbouring pETRA with sRNA 13575 (13573), and *A*. *baumannii* ATCC 17978 harbouring pETRA (17978 with empty pETRA).
(DOCX)

S6 Fig Growth curves. Growth curves of wild type *A*. *baumannii* (17978), *A*. *baumannii* ATCC 17978 lacking the sRNA 13573 (Δ13573), *A*. *baumannii* Δ13573 over-expressing sRNA 13575 (Δ13573 complemented), *A*. *baumannii* ATCC 17978 harbouring pETRA over-expressing sRNA 13575 (13573) and *A*. *baumannii* ATCC 17978 harbouring the empty pETRA vector (17978 with empty pETRA).
(DOCX)
