# Supplementary material for: Global assessment of small RNAs reveals a non-coding transcript involved in biofilm formation and attachment in Acinetobacter baumannii ATCC 17978
Source: PLoS One. 2017 Aug 1;12(8):e0182084. doi: 10.1371/journal.pone.0182084 (PMC5538643; doi:10.1371/journal.pone.0182084)
Supplement: S1 Text — (DOCX) [file pone.0182084.s001.docx]

**S1 Text. Additional details about 454 read data processing.**

**Processing of sRNA 454 sequencing reads**

sRNA fractions, obtained from liquid *A. baumannii* ATCC 17978 cultures in the exponential and stationary phases of growth, plus biofilms were pyrosequenced at the Roche 454 Sequencing Center (Connecticut, USA) using GS FLX Titanium chemistry. The total number of reads was 689,097, 502,152 and 627,209 for the exponential, stationary and biofilm samples, respectively. FASTA formatted sequences and quality values extracted from SFF files were used as input for cross-match (Phred-Phrap-Consed package) to mask the sequence of the adapters used with the SREK kit (Table S1). Masked segments were then removed with Trimseq (Emboss package). No procedure to eliminate identical reads was performed, given that the pyrosequencing libraries consisted of short cDNA sequences representing, in principle, full-length sRNAs and the presence of identical reads was expected.

Pre-processed reads were aligned with cross-match against the *A. baumannii* ATCC 17978 chromosome and plasmid nucleotide sequences (Genbank: NC_009085, NC_009083 and NC_009084). Alignments were filtered with an *ad-hoc* Perl script (crossMatchParser) to recover, for each read, those alignments with the highest score and fulfilling the minimal requirements of 90% identity and 15-base pair length. Between 32 % and 43 % of pre-processed reads could be aligned for each sample (Table S2). The total number of filtered alignments was around twice the number of aligned reads, indicating that a significant number of reads could align to more than one location with the same score.

**Quantification of the expression level for known sRNA genes**

To estimate the expression level for known sRNA genes described for *A. baumannii* ATCC 17978, which included 74 genes coding for 5S rRNAs and tRNA, filtered alignments were processed with the *ad-hoc* script mapAlignHits, which in “expression mode” calculated the average coverage for sets of target sequence segments defined by gene coordinates. Relative abundance (RA) values were also calculated for known protein coding genes and for 16S and 23S rRNA genes (Table S3). Reads aligning to multiple locations with identical score contributed to the coverage of each region with an amount that was inversely proportional to the number of locations. The resulting average coverage values were normalized to obtain relative abundance (RA) values, by dividing them by the total number of mapped reads for each sample, and multiplying them by the number of mapped reads for the exponential phase sample, which was defined as the reference condition. tRNA and 5S rRNA genes had RA values of 1,092 to 1,338, in average, for the three samples, which were more than 1,000-fold and about 20-fold higher than RA values calculated for protein coding genes and 16S and 23S rRNA genes, respectively (Table S4 and Figure S1), suggesting that sRNA fractions isolated as described in Methods were significantly depleted of mRNA and rRNA. A1S_2909, coding for Leu tRNA, was the known sRNA gene expressed at the highest level, with a RA value of 18,693 in the stationary phase sample. In contrast, A1S_2764, coding for Arg tRNA, was the known sRNA gene expressed at the lowest level, with a RA value of 7.6 in the exponential phase sample.

**Identification of new sRNA gene candidates**

To identify new sRNA gene candidates, filtered alignments were combined into a single file and processed with script mapAlignHits, which, in “detection mode” identified continuous read-covered regions, with a minimal average coverage of 1, in target sequences (chromosome and plasmids). A total of 26,956 read-covered regions (exprRegs) were detected. To identify read-covered regions overlapping with known genes, the *ad-hoc* script FindOlappingFeatures was used to compare their coordinates with the coordinates of 3,451 known protein, tRNA and rRNA coding genes. The minimal overlap required to consider two features as overlapping was set to 0.001 % of the length of any of the features. A total of 21,392 exprRegs were found to overlap with already described genes. The remaining 5,564 exprRegs (Dataset S1) were considered as new potential sRNA genes. However, since the coordinate set used to define known protein coding genes referred exclusively to coding regions, some of the 5,564 exprRegs could correspond to 5' or 3' UTRs of genes that had not been covered by a single, continuous alignment, spanning the whole length of the transcribed region. New sRNA gene candidates were identified both in chromosome and plasmids (5,505 and 59 genes, respectively). Their average length was 63.39 base pairs (Figure S2).
