# Supplementary material for: Global assessment of small RNAs reveals a non-coding transcript involved in biofilm formation and attachment in Acinetobacter baumannii ATCC 17978
Source: PLoS One. 2017 Aug 1;12(8):e0182084. doi: 10.1371/journal.pone.0182084 (PMC5538643; doi:10.1371/journal.pone.0182084)
Supplement: S1 Table — (DOCX) [file pone.0182084.s002.docx]

**S1 Table. Oligonucleotides and probes used in the present study.**

| Oligonucleotide name | Oligonucleotide sequence | Use of the oligonucleotides |
| --- | --- | --- |
| 13573 F | ccctctagagggattattatgactgcttaaatc | Over-expression of sRNA 13573 in pETRA |
| 13573 R | gggccatggcccaataggttagtaaggtaataa | Over-expression of sRNA 13573 in pETRA |
| KM F | cccctgcaggggccggaattgccagctggggcg | Cloning kanamycin resistance cassette in pETRA |
| KM R | gggctgcagccctcagaagaactcgtcaagaag | Cloning kanamycin resistance cassette in pETRA |
| SREK 1 | ctgccccgggttcctcattctctgcggtcctgctgtacggccaaggcg | Construction of cDNA libraries  SREK (Ambion) |
| SREK 2 | ccactacgcctccgctttcctctctatgggcagtcggtgat | Construction of cDNA libraries  SREK (Ambion) |
| 13573BUpFNotI | cccgcggccgcgggttggtcaaaggtgtgaaaatgt | Construction of the knockout mutant of sRNA 13573 |
| 13573 UpR3 BamHI | gggggatcccccactaaagtatctatttgggtgtacg | Construction of the knockout mutant of sRNA 13573 |
| 13573 DownF3 BamHI | cccggatccgggaggatcggttattgaatcag | Construction of the knockout mutant of sRNA 13573 |
| 13573DownRSphI | ggggcatgcccccaagtcacaagcaccttcttt | Construction of the knockout mutant of sRNA 13573 |
| 13573extF2 | tataaggtgtaagcacgctg | Checking of the knock-out mutant of sRNA 13573 |
| 13573extR2 | tcacgagacaagagatgaac | Checking of the knockout mutant of sRNA 13573 |
