# Supplementary material for: Global assessment of small RNAs reveals a non-coding transcript involved in biofilm formation and attachment in Acinetobacter baumannii ATCC 17978
Source: PLoS One. 2017 Aug 1;12(8):e0182084. doi: 10.1371/journal.pone.0182084 (PMC5538643; doi:10.1371/journal.pone.0182084)
Supplement: S2 Table — (DOCX) [file pone.0182084.s003.docx]

**S2 Table. Number of initial reads, mapped reads and alignment hits for each sample.**

| Sample | Number of Reads | Number of Mapped Reads | Number of Hits |
| --- | --- | --- | --- |
| Exp | 689,097 | 298,048 (43%) | 691,710 |
| Sta | 502,152 | 176,003 (35%) | 429,806 |
| Bio | 627,209 | 204,637 (32%) | 505,760 |

Biofilm: Bio. Exponential phase of growth: Exp. Stationary phase of growth: Sta.
