# Supplementary material for: Global assessment of small RNAs reveals a non-coding transcript involved in biofilm formation and attachment in Acinetobacter baumannii ATCC 17978
Source: PLoS One. 2017 Aug 1;12(8):e0182084. doi: 10.1371/journal.pone.0182084 (PMC5538643; doi:10.1371/journal.pone.0182084)
Supplement: S3 Table — (DOCX) [file pone.0182084.s004.docx]

**S3 Table. *A. baumannii* known sRNA and non-sRNA genes used as reference.**

| Gene set | Subset | Number of genes |
| --- | --- | --- |
| rRNA 5S | Known sRNA | 5 |
| tRNA | Known sRNA | 69 |
| rRNA 16S and 23 S | Known, non sRNA | 10 |
| Protein coding | Known, non sRNA | 3,367 |
