# Supplementary material for: Global assessment of small RNAs reveals a non-coding transcript involved in biofilm formation and attachment in Acinetobacter baumannii ATCC 17978
Source: PLoS One. 2017 Aug 1;12(8):e0182084. doi: 10.1371/journal.pone.0182084 (PMC5538643; doi:10.1371/journal.pone.0182084)
Supplement: S4 Table — (DOCX) [file pone.0182084.s005.docx]

**S4 Table. Average, standard deviation, maximal and minimal values for the normalized expression scores calculated for protein coding genes, 16S and 23S rRNA genes and known sRNA genes, in each of the growing conditions.**

|  | Protein Coding | | | 16S, 23S | | | tRNA, 5S | | |
| --- | --- | --- | --- | --- | --- | --- | --- | --- | --- |
|  | BioN | ExpN | StaN | BioN | ExpN | StaN | BioN | ExpN | StaN |
| Ave | 0.62 | 1.05 | 1.15 | 57.27 | 35.41 | 61.07 | 1,260.37 | 1,092.71 | 1,338.55 |
| Std | 1.03 | 1.27 | 1.32 | 1.72 | 3.09 | 16.6 | 2,719.95 | 1,797.35 | 2,689.99 |
| Max | 20.34 | 26.49 | 27.48 | 59.15 | 38.44 | 77.3 | 18,375.78 | 9,944.99 | 18,693.1 |
| Min | 0 | 0 | 0 | 55.65 | 32.08 | 45.3 | 15.9 | 7.6 | 14.93 |

Biofilm: BioN. Exponential phase of growth: ExpN. Stationary phase of growth: StaN. Average: Ave. Standard deviation: Std. Maximal values: Max. Minimal values: Min.
