# Supplementary material for: Global assessment of small RNAs reveals a non-coding transcript involved in biofilm formation and attachment in Acinetobacter baumannii ATCC 17978
Source: PLoS One. 2017 Aug 1;12(8):e0182084. doi: 10.1371/journal.pone.0182084 (PMC5538643; doi:10.1371/journal.pone.0182084)
Supplement: S5 Table — (DOCX) [file pone.0182084.s006.docx]

**S5 Table. Average, standard deviation, maximal and minimal values for the normalized expression scores calculated for 5564 expressed regions not overlapping with known genes.**

|  | Bio | Exp | Sta |
| --- | --- | --- | --- |
| Ave | 1.83 | 2.01 | 2.06 |
| Std | 20.62 | 12.96 | 27.64 |
| Max | 826.28 | 484.14 | 1,728.21 |
| Min | 0 | 0 | 0 |

Biofilm samples: Bio. Exponential phase of growth samples: Exp. Stationary phase of growth samples: Sta. Average: Ave. Standard deviation: Std.
