# Supplementary material for: Global assessment of small RNAs reveals a non-coding transcript involved in biofilm formation and attachment in Acinetobacter baumannii ATCC 17978
Source: PLoS One. 2017 Aug 1;12(8):e0182084. doi: 10.1371/journal.pone.0182084 (PMC5538643; doi:10.1371/journal.pone.0182084)
Supplement: S6 Table — (DOCX) [file pone.0182084.s007.docx]

**S6 Table. Sets of expression regions not overlapping with known genes and having a normalized expression score equal or higher than 7.6 in some of the three growing conditions.**

|  | Bio | Exp | Sta |
| --- | --- | --- | --- |
| Number of expression regions | 108 | 140 | 121 |
| Average normalized score | 57.88 | 35.64 | 54.53 |
| Standard deviation | 137.16 | 74.12 | 180.27 |
| Maximal normalized score | 826.28 | 484.14 | 1,728.21 |
| Minimal normalized score | 7.74 | 7.6 | 7.7 |
