# Supplementary material for: Global assessment of small RNAs reveals a non-coding transcript involved in biofilm formation and attachment in Acinetobacter baumannii ATCC 17978
Source: PLoS One. 2017 Aug 1;12(8):e0182084. doi: 10.1371/journal.pone.0182084 (PMC5538643; doi:10.1371/journal.pone.0182084)
Supplement: S1 Dataset — List of 5,564 expressed regions, their length and locations in the genome (NC_009085.1) or plasmids (NC_009083.1 and NC_009084.1) of A. baumannii ATCC 17978. (DOCX) [file pone.0182084.s008.docx]

**S1 Dataset. Expressed regions.** List of 5,564 expressed regions, their length and locations in the genome (NC_009085.1) or plasmids (NC_009083.1 and NC_009084.1) of *A. baumannii* ATCC 17978.

| **Expressed region** | **Genetic element** | **Start** | **End** | **Length** |
| --- | --- | --- | --- | --- |
| exprReg_4 | gi|126640109|ref|NC_009084.1| | 1632 | 2146 | 515 |
| exprReg_5 | gi|126640109|ref|NC_009084.1| | 2149 | 2777 | 629 |
| exprReg_7 | gi|126640109|ref|NC_009084.1| | 5300 | 5325 | 26 |
| exprReg_20 | gi|126640109|ref|NC_009084.1| | 9464 | 9960 | 497 |
| exprReg_21 | gi|126640109|ref|NC_009084.1| | 9994 | 10010 | 17 |
| exprReg_22 | gi|126640109|ref|NC_009084.1| | 10012 | 10071 | 60 |
| exprReg_23 | gi|126640109|ref|NC_009084.1| | 10171 | 10190 | 20 |
| exprReg_24 | gi|126640109|ref|NC_009084.1| | 10331 | 10551 | 221 |
| exprReg_25 | gi|126640109|ref|NC_009084.1| | 10746 | 10764 | 19 |
| exprReg_26 | gi|126640109|ref|NC_009084.1| | 10870 | 10896 | 27 |
| exprReg_27 | gi|126640109|ref|NC_009084.1| | 10920 | 10947 | 28 |
| exprReg_28 | gi|126640109|ref|NC_009084.1| | 11008 | 11244 | 237 |
| exprReg_29 | gi|126640109|ref|NC_009084.1| | 11279 | 11302 | 25 |
| exprReg_38 | gi|126640097|ref|NC_009083.1| | 1525 | 1705 | 181 |
| exprReg_39 | gi|126640097|ref|NC_009083.1| | 1708 | 1901 | 194 |
| exprReg_40 | gi|126640097|ref|NC_009083.1| | 1922 | 1941 | 20 |
| exprReg_41 | gi|126640097|ref|NC_009083.1| | 1945 | 2009 | 65 |
| exprReg_42 | gi|126640097|ref|NC_009083.1| | 2035 | 2098 | 64 |
| exprReg_43 | gi|126640097|ref|NC_009083.1| | 2124 | 2175 | 52 |
| exprReg_44 | gi|126640097|ref|NC_009083.1| | 2192 | 2210 | 19 |
| exprReg_45 | gi|126640097|ref|NC_009083.1| | 2243 | 2288 | 46 |
| exprReg_46 | gi|126640097|ref|NC_009083.1| | 2337 | 2405 | 69 |
| exprReg_49 | gi|126640097|ref|NC_009083.1| | 4885 | 4983 | 99 |
| exprReg_52 | gi|126640097|ref|NC_009083.1| | 5697 | 6017 | 321 |
| exprReg_53 | gi|126640097|ref|NC_009083.1| | 6031 | 6047 | 17 |
| exprReg_54 | gi|126640097|ref|NC_009083.1| | 6073 | 6113 | 41 |
| exprReg_55 | gi|126640097|ref|NC_009083.1| | 6122 | 6184 | 63 |
| exprReg_56 | gi|126640097|ref|NC_009083.1| | 6190 | 6227 | 38 |
| exprReg_57 | gi|126640097|ref|NC_009083.1| | 6229 | 6263 | 35 |
| exprReg_61 | gi|126640097|ref|NC_009083.1| | 6933 | 6957 | 25 |
| exprReg_62 | gi|126640097|ref|NC_009083.1| | 6976 | 6995 | 20 |
| exprReg_65 | gi|126640097|ref|NC_009083.1| | 7704 | 7762 | 59 |
| exprReg_66 | gi|126640097|ref|NC_009083.1| | 7769 | 7846 | 78 |
| exprReg_67 | gi|126640097|ref|NC_009083.1| | 7848 | 7946 | 99 |
| exprReg_68 | gi|126640097|ref|NC_009083.1| | 8012 | 8118 | 107 |
| exprReg_69 | gi|126640097|ref|NC_009083.1| | 8152 | 8329 | 178 |
| exprReg_70 | gi|126640097|ref|NC_009083.1| | 8353 | 8399 | 47 |
| exprReg_81 | gi|126640097|ref|NC_009083.1| | 10807 | 10939 | 133 |
| exprReg_82 | gi|126640097|ref|NC_009083.1| | 10972 | 10992 | 21 |
| exprReg_83 | gi|126640097|ref|NC_009083.1| | 10999 | 11083 | 85 |
| exprReg_84 | gi|126640097|ref|NC_009083.1| | 11115 | 11220 | 106 |
| exprReg_85 | gi|126640097|ref|NC_009083.1| | 11223 | 11283 | 61 |
| exprReg_86 | gi|126640097|ref|NC_009083.1| | 11328 | 11361 | 34 |
| exprReg_87 | gi|126640097|ref|NC_009083.1| | 11414 | 11449 | 36 |
| exprReg_88 | gi|126640097|ref|NC_009083.1| | 11585 | 11613 | 29 |
| exprReg_89 | gi|126640097|ref|NC_009083.1| | 11635 | 11697 | 63 |
| exprReg_90 | gi|126640097|ref|NC_009083.1| | 11728 | 11954 | 227 |
| exprReg_91 | gi|126640097|ref|NC_009083.1| | 11981 | 12026 | 46 |
| exprReg_92 | gi|126640097|ref|NC_009083.1| | 12029 | 12073 | 45 |
| exprReg_95 | gi|126640097|ref|NC_009083.1| | 12456 | 12601 | 146 |
| exprReg_96 | gi|126640097|ref|NC_009083.1| | 12642 | 12829 | 188 |
| exprReg_97 | gi|126640097|ref|NC_009083.1| | 12845 | 12866 | 22 |
| exprReg_98 | gi|126640097|ref|NC_009083.1| | 12888 | 12907 | 20 |
| exprReg_99 | gi|126640097|ref|NC_009083.1| | 12924 | 12953 | 30 |
| exprReg_100 | gi|126640097|ref|NC_009083.1| | 12991 | 13010 | 20 |
| exprReg_101 | gi|126640097|ref|NC_009083.1| | 13013 | 13061 | 49 |
| exprReg_102 | gi|126640097|ref|NC_009083.1| | 13090 | 13114 | 25 |
| exprReg_103 | gi|126640097|ref|NC_009083.1| | 13174 | 13195 | 22 |
| exprReg_104 | gi|126640097|ref|NC_009083.1| | 13257 | 13297 | 41 |
| exprReg_105 | gi|126640115|ref|NC_009085.1| | 1 | 52 | 52 |
| exprReg_106 | gi|126640115|ref|NC_009085.1| | 61 | 263 | 203 |
| exprReg_119 | gi|126640115|ref|NC_009085.1| | 1531 | 1671 | 141 |
| exprReg_132 | gi|126640115|ref|NC_009085.1| | 2855 | 2920 | 66 |
| exprReg_140 | gi|126640115|ref|NC_009085.1| | 3879 | 3901 | 23 |
| exprReg_154 | gi|126640115|ref|NC_009085.1| | 6374 | 6390 | 17 |
| exprReg_162 | gi|126640115|ref|NC_009085.1| | 7313 | 7388 | 76 |
| exprReg_163 | gi|126640115|ref|NC_009085.1| | 7504 | 7529 | 26 |
| exprReg_164 | gi|126640115|ref|NC_009085.1| | 7577 | 7593 | 17 |
| exprReg_165 | gi|126640115|ref|NC_009085.1| | 7618 | 7679 | 62 |
| exprReg_188 | gi|126640115|ref|NC_009085.1| | 10886 | 10964 | 79 |
| exprReg_189 | gi|126640115|ref|NC_009085.1| | 11006 | 11087 | 82 |
| exprReg_196 | gi|126640115|ref|NC_009085.1| | 12239 | 12297 | 59 |
| exprReg_197 | gi|126640115|ref|NC_009085.1| | 12356 | 12398 | 43 |
| exprReg_198 | gi|126640115|ref|NC_009085.1| | 12455 | 12475 | 21 |
| exprReg_211 | gi|126640115|ref|NC_009085.1| | 14916 | 14932 | 17 |
| exprReg_221 | gi|126640115|ref|NC_009085.1| | 15983 | 16111 | 129 |
| exprReg_222 | gi|126640115|ref|NC_009085.1| | 16177 | 16222 | 46 |
| exprReg_223 | gi|126640115|ref|NC_009085.1| | 16265 | 16285 | 21 |
| exprReg_224 | gi|126640115|ref|NC_009085.1| | 16303 | 16323 | 21 |
| exprReg_231 | gi|126640115|ref|NC_009085.1| | 17813 | 17840 | 28 |
| exprReg_232 | gi|126640115|ref|NC_009085.1| | 17940 | 17960 | 21 |
| exprReg_233 | gi|126640115|ref|NC_009085.1| | 18020 | 18329 | 310 |
| exprReg_234 | gi|126640115|ref|NC_009085.1| | 18575 | 18591 | 17 |
| exprReg_235 | gi|126640115|ref|NC_009085.1| | 18677 | 18703 | 27 |
| exprReg_236 | gi|126640115|ref|NC_009085.1| | 18705 | 18827 | 123 |
| exprReg_237 | gi|126640115|ref|NC_009085.1| | 19053 | 19133 | 81 |
| exprReg_238 | gi|126640115|ref|NC_009085.1| | 19152 | 19282 | 131 |
| exprReg_239 | gi|126640115|ref|NC_009085.1| | 19419 | 19453 | 35 |
| exprReg_240 | gi|126640115|ref|NC_009085.1| | 19474 | 19597 | 124 |
| exprReg_241 | gi|126640115|ref|NC_009085.1| | 19626 | 19794 | 169 |
| exprReg_242 | gi|126640115|ref|NC_009085.1| | 19894 | 19987 | 94 |
| exprReg_243 | gi|126640115|ref|NC_009085.1| | 20053 | 20148 | 96 |
| exprReg_244 | gi|126640115|ref|NC_009085.1| | 20235 | 20304 | 70 |
| exprReg_254 | gi|126640115|ref|NC_009085.1| | 22041 | 22157 | 117 |
| exprReg_264 | gi|126640115|ref|NC_009085.1| | 23240 | 23307 | 68 |
| exprReg_265 | gi|126640115|ref|NC_009085.1| | 23376 | 23392 | 17 |
| exprReg_266 | gi|126640115|ref|NC_009085.1| | 23395 | 23458 | 64 |
| exprReg_267 | gi|126640115|ref|NC_009085.1| | 23480 | 23554 | 75 |
| exprReg_268 | gi|126640115|ref|NC_009085.1| | 23558 | 23618 | 61 |
| exprReg_269 | gi|126640115|ref|NC_009085.1| | 23634 | 23707 | 74 |
| exprReg_270 | gi|126640115|ref|NC_009085.1| | 23787 | 23808 | 22 |
| exprReg_271 | gi|126640115|ref|NC_009085.1| | 23872 | 23888 | 17 |
| exprReg_272 | gi|126640115|ref|NC_009085.1| | 23901 | 24001 | 101 |
| exprReg_273 | gi|126640115|ref|NC_009085.1| | 24010 | 24185 | 176 |
| exprReg_274 | gi|126640115|ref|NC_009085.1| | 24201 | 24218 | 18 |
| exprReg_275 | gi|126640115|ref|NC_009085.1| | 24268 | 24285 | 18 |
| exprReg_276 | gi|126640115|ref|NC_009085.1| | 24322 | 24339 | 18 |
| exprReg_277 | gi|126640115|ref|NC_009085.1| | 24424 | 24442 | 19 |
| exprReg_278 | gi|126640115|ref|NC_009085.1| | 24527 | 24555 | 29 |
| exprReg_279 | gi|126640115|ref|NC_009085.1| | 24644 | 24661 | 18 |
| exprReg_280 | gi|126640115|ref|NC_009085.1| | 24691 | 24719 | 29 |
| exprReg_281 | gi|126640115|ref|NC_009085.1| | 24730 | 24760 | 31 |
| exprReg_282 | gi|126640115|ref|NC_009085.1| | 24776 | 24837 | 62 |
| exprReg_283 | gi|126640115|ref|NC_009085.1| | 24872 | 24915 | 44 |
| exprReg_284 | gi|126640115|ref|NC_009085.1| | 24928 | 25002 | 75 |
| exprReg_316 | gi|126640115|ref|NC_009085.1| | 29619 | 29662 | 44 |
| exprReg_317 | gi|126640115|ref|NC_009085.1| | 29667 | 29732 | 66 |
| exprReg_318 | gi|126640115|ref|NC_009085.1| | 29803 | 29922 | 120 |
| exprReg_319 | gi|126640115|ref|NC_009085.1| | 29968 | 30098 | 131 |
| exprReg_320 | gi|126640115|ref|NC_009085.1| | 30149 | 30171 | 23 |
| exprReg_321 | gi|126640115|ref|NC_009085.1| | 30275 | 30295 | 21 |
| exprReg_322 | gi|126640115|ref|NC_009085.1| | 30328 | 30349 | 22 |
| exprReg_323 | gi|126640115|ref|NC_009085.1| | 30351 | 30598 | 248 |
| exprReg_324 | gi|126640115|ref|NC_009085.1| | 30609 | 30685 | 77 |
| exprReg_353 | gi|126640115|ref|NC_009085.1| | 34897 | 35046 | 150 |
| exprReg_367 | gi|126640115|ref|NC_009085.1| | 37268 | 37288 | 21 |
| exprReg_368 | gi|126640115|ref|NC_009085.1| | 37309 | 37362 | 54 |
| exprReg_383 | gi|126640115|ref|NC_009085.1| | 39945 | 39992 | 48 |
| exprReg_384 | gi|126640115|ref|NC_009085.1| | 40028 | 40045 | 18 |
| exprReg_389 | gi|126640115|ref|NC_009085.1| | 41157 | 41312 | 156 |
| exprReg_407 | gi|126640115|ref|NC_009085.1| | 43454 | 43493 | 40 |
| exprReg_408 | gi|126640115|ref|NC_009085.1| | 43536 | 43723 | 188 |
| exprReg_417 | gi|126640115|ref|NC_009085.1| | 45012 | 45163 | 152 |
| exprReg_418 | gi|126640115|ref|NC_009085.1| | 45168 | 45187 | 20 |
| exprReg_423 | gi|126640115|ref|NC_009085.1| | 45726 | 45834 | 109 |
| exprReg_424 | gi|126640115|ref|NC_009085.1| | 45985 | 46023 | 39 |
| exprReg_425 | gi|126640115|ref|NC_009085.1| | 46029 | 46045 | 17 |
| exprReg_426 | gi|126640115|ref|NC_009085.1| | 46089 | 46140 | 52 |
| exprReg_438 | gi|126640115|ref|NC_009085.1| | 47056 | 47113 | 58 |
| exprReg_447 | gi|126640115|ref|NC_009085.1| | 48336 | 48388 | 53 |
| exprReg_448 | gi|126640115|ref|NC_009085.1| | 48421 | 48438 | 18 |
| exprReg_454 | gi|126640115|ref|NC_009085.1| | 49165 | 49191 | 27 |
| exprReg_455 | gi|126640115|ref|NC_009085.1| | 49267 | 49314 | 48 |
| exprReg_456 | gi|126640115|ref|NC_009085.1| | 49360 | 49426 | 67 |
| exprReg_469 | gi|126640115|ref|NC_009085.1| | 51609 | 51642 | 34 |
| exprReg_470 | gi|126640115|ref|NC_009085.1| | 51651 | 51667 | 17 |
| exprReg_471 | gi|126640115|ref|NC_009085.1| | 51757 | 51827 | 71 |
| exprReg_475 | gi|126640115|ref|NC_009085.1| | 52971 | 53007 | 37 |
| exprReg_476 | gi|126640115|ref|NC_009085.1| | 53104 | 53124 | 21 |
| exprReg_477 | gi|126640115|ref|NC_009085.1| | 53128 | 53144 | 17 |
| exprReg_481 | gi|126640115|ref|NC_009085.1| | 53775 | 53954 | 180 |
| exprReg_490 | gi|126640115|ref|NC_009085.1| | 55345 | 55364 | 20 |
| exprReg_497 | gi|126640115|ref|NC_009085.1| | 55988 | 56010 | 23 |
| exprReg_498 | gi|126640115|ref|NC_009085.1| | 56016 | 56048 | 33 |
| exprReg_522 | gi|126640115|ref|NC_009085.1| | 59044 | 59126 | 83 |
| exprReg_523 | gi|126640115|ref|NC_009085.1| | 59129 | 59229 | 101 |
| exprReg_535 | gi|126640115|ref|NC_009085.1| | 60996 | 61016 | 21 |
| exprReg_536 | gi|126640115|ref|NC_009085.1| | 61020 | 61311 | 292 |
| exprReg_584 | gi|126640115|ref|NC_009085.1| | 67031 | 67121 | 91 |
| exprReg_585 | gi|126640115|ref|NC_009085.1| | 67214 | 67251 | 38 |
| exprReg_598 | gi|126640115|ref|NC_009085.1| | 68496 | 68582 | 87 |
| exprReg_599 | gi|126640115|ref|NC_009085.1| | 68600 | 68647 | 48 |
| exprReg_600 | gi|126640115|ref|NC_009085.1| | 68651 | 68796 | 146 |
| exprReg_601 | gi|126640115|ref|NC_009085.1| | 68862 | 68924 | 63 |
| exprReg_602 | gi|126640115|ref|NC_009085.1| | 68993 | 69200 | 208 |
| exprReg_603 | gi|126640115|ref|NC_009085.1| | 69269 | 69294 | 26 |
| exprReg_604 | gi|126640115|ref|NC_009085.1| | 69385 | 69418 | 34 |
| exprReg_605 | gi|126640115|ref|NC_009085.1| | 69509 | 69600 | 92 |
| exprReg_606 | gi|126640115|ref|NC_009085.1| | 69622 | 69648 | 27 |
| exprReg_607 | gi|126640115|ref|NC_009085.1| | 69675 | 69713 | 39 |
| exprReg_608 | gi|126640115|ref|NC_009085.1| | 69750 | 69852 | 103 |
| exprReg_609 | gi|126640115|ref|NC_009085.1| | 69931 | 70041 | 111 |
| exprReg_610 | gi|126640115|ref|NC_009085.1| | 70060 | 70095 | 36 |
| exprReg_611 | gi|126640115|ref|NC_009085.1| | 70158 | 70322 | 165 |
| exprReg_612 | gi|126640115|ref|NC_009085.1| | 70399 | 70456 | 58 |
| exprReg_613 | gi|126640115|ref|NC_009085.1| | 70459 | 70488 | 30 |
| exprReg_614 | gi|126640115|ref|NC_009085.1| | 70511 | 70529 | 19 |
| exprReg_615 | gi|126640115|ref|NC_009085.1| | 70595 | 70613 | 19 |
| exprReg_643 | gi|126640115|ref|NC_009085.1| | 74109 | 74136 | 28 |
| exprReg_644 | gi|126640115|ref|NC_009085.1| | 74164 | 74230 | 67 |
| exprReg_645 | gi|126640115|ref|NC_009085.1| | 74423 | 74494 | 72 |
| exprReg_651 | gi|126640115|ref|NC_009085.1| | 75498 | 75567 | 70 |
| exprReg_652 | gi|126640115|ref|NC_009085.1| | 75571 | 75595 | 25 |
| exprReg_653 | gi|126640115|ref|NC_009085.1| | 75648 | 75673 | 26 |
| exprReg_654 | gi|126640115|ref|NC_009085.1| | 75677 | 75694 | 18 |
| exprReg_669 | gi|126640115|ref|NC_009085.1| | 77133 | 77181 | 49 |
| exprReg_670 | gi|126640115|ref|NC_009085.1| | 77239 | 77262 | 24 |
| exprReg_684 | gi|126640115|ref|NC_009085.1| | 79421 | 79437 | 17 |
| exprReg_685 | gi|126640115|ref|NC_009085.1| | 79476 | 79504 | 29 |
| exprReg_686 | gi|126640115|ref|NC_009085.1| | 79548 | 79615 | 68 |
| exprReg_687 | gi|126640115|ref|NC_009085.1| | 79796 | 79815 | 20 |
| exprReg_706 | gi|126640115|ref|NC_009085.1| | 82356 | 82374 | 19 |
| exprReg_709 | gi|126640115|ref|NC_009085.1| | 83673 | 83918 | 246 |
| exprReg_727 | gi|126640115|ref|NC_009085.1| | 86803 | 86830 | 28 |
| exprReg_728 | gi|126640115|ref|NC_009085.1| | 86849 | 87030 | 182 |
| exprReg_729 | gi|126640115|ref|NC_009085.1| | 87147 | 87195 | 49 |
| exprReg_730 | gi|126640115|ref|NC_009085.1| | 87255 | 87361 | 107 |
| exprReg_731 | gi|126640115|ref|NC_009085.1| | 87387 | 87484 | 98 |
| exprReg_739 | gi|126640115|ref|NC_009085.1| | 88885 | 88999 | 115 |
| exprReg_740 | gi|126640115|ref|NC_009085.1| | 89011 | 89044 | 34 |
| exprReg_741 | gi|126640115|ref|NC_009085.1| | 89065 | 89087 | 23 |
| exprReg_742 | gi|126640115|ref|NC_009085.1| | 89096 | 89114 | 19 |
| exprReg_743 | gi|126640115|ref|NC_009085.1| | 89145 | 89186 | 42 |
| exprReg_744 | gi|126640115|ref|NC_009085.1| | 89238 | 89441 | 204 |
| exprReg_745 | gi|126640115|ref|NC_009085.1| | 89506 | 89569 | 64 |
| exprReg_746 | gi|126640115|ref|NC_009085.1| | 89780 | 90122 | 343 |
| exprReg_747 | gi|126640115|ref|NC_009085.1| | 90128 | 90159 | 32 |
| exprReg_786 | gi|126640115|ref|NC_009085.1| | 95494 | 95609 | 116 |
| exprReg_787 | gi|126640115|ref|NC_009085.1| | 95700 | 95718 | 19 |
| exprReg_788 | gi|126640115|ref|NC_009085.1| | 95795 | 95833 | 39 |
| exprReg_789 | gi|126640115|ref|NC_009085.1| | 95849 | 95865 | 17 |
| exprReg_790 | gi|126640115|ref|NC_009085.1| | 95905 | 95955 | 51 |
| exprReg_791 | gi|126640115|ref|NC_009085.1| | 96003 | 96049 | 47 |
| exprReg_793 | gi|126640115|ref|NC_009085.1| | 96389 | 96478 | 90 |
| exprReg_794 | gi|126640115|ref|NC_009085.1| | 96483 | 96634 | 152 |
| exprReg_795 | gi|126640115|ref|NC_009085.1| | 96670 | 96783 | 114 |
| exprReg_796 | gi|126640115|ref|NC_009085.1| | 96822 | 96901 | 80 |
| exprReg_797 | gi|126640115|ref|NC_009085.1| | 96903 | 96919 | 17 |
| exprReg_798 | gi|126640115|ref|NC_009085.1| | 97070 | 97107 | 38 |
| exprReg_801 | gi|126640115|ref|NC_009085.1| | 97585 | 97700 | 116 |
| exprReg_802 | gi|126640115|ref|NC_009085.1| | 97739 | 97920 | 182 |
| exprReg_803 | gi|126640115|ref|NC_009085.1| | 97940 | 97984 | 45 |
| exprReg_804 | gi|126640115|ref|NC_009085.1| | 97998 | 98111 | 114 |
| exprReg_805 | gi|126640115|ref|NC_009085.1| | 98405 | 98649 | 245 |
| exprReg_816 | gi|126640115|ref|NC_009085.1| | 100927 | 100945 | 19 |
| exprReg_817 | gi|126640115|ref|NC_009085.1| | 100953 | 100990 | 38 |
| exprReg_818 | gi|126640115|ref|NC_009085.1| | 101044 | 101139 | 96 |
| exprReg_819 | gi|126640115|ref|NC_009085.1| | 101155 | 101186 | 32 |
| exprReg_820 | gi|126640115|ref|NC_009085.1| | 101190 | 101647 | 458 |
| exprReg_821 | gi|126640115|ref|NC_009085.1| | 101707 | 101734 | 28 |
| exprReg_822 | gi|126640115|ref|NC_009085.1| | 101797 | 102022 | 226 |
| exprReg_823 | gi|126640115|ref|NC_009085.1| | 102039 | 102130 | 92 |
| exprReg_824 | gi|126640115|ref|NC_009085.1| | 102139 | 102252 | 114 |
| exprReg_825 | gi|126640115|ref|NC_009085.1| | 102259 | 102391 | 133 |
| exprReg_826 | gi|126640115|ref|NC_009085.1| | 102456 | 102638 | 183 |
| exprReg_827 | gi|126640115|ref|NC_009085.1| | 102645 | 102704 | 60 |
| exprReg_828 | gi|126640115|ref|NC_009085.1| | 102754 | 102787 | 34 |
| exprReg_829 | gi|126640115|ref|NC_009085.1| | 102800 | 102823 | 24 |
| exprReg_830 | gi|126640115|ref|NC_009085.1| | 102878 | 103125 | 248 |
| exprReg_831 | gi|126640115|ref|NC_009085.1| | 103137 | 103188 | 52 |
| exprReg_832 | gi|126640115|ref|NC_009085.1| | 103343 | 103537 | 195 |
| exprReg_833 | gi|126640115|ref|NC_009085.1| | 103545 | 103589 | 45 |
| exprReg_834 | gi|126640115|ref|NC_009085.1| | 103729 | 103760 | 32 |
| exprReg_835 | gi|126640115|ref|NC_009085.1| | 103771 | 103789 | 19 |
| exprReg_843 | gi|126640115|ref|NC_009085.1| | 104797 | 104839 | 43 |
| exprReg_844 | gi|126640115|ref|NC_009085.1| | 104911 | 105184 | 274 |
| exprReg_845 | gi|126640115|ref|NC_009085.1| | 105573 | 105595 | 23 |
| exprReg_846 | gi|126640115|ref|NC_009085.1| | 105612 | 105686 | 75 |
| exprReg_847 | gi|126640115|ref|NC_009085.1| | 105769 | 105875 | 107 |
| exprReg_848 | gi|126640115|ref|NC_009085.1| | 105881 | 105907 | 27 |
| exprReg_849 | gi|126640115|ref|NC_009085.1| | 106273 | 106379 | 107 |
| exprReg_853 | gi|126640115|ref|NC_009085.1| | 107184 | 107274 | 91 |
| exprReg_854 | gi|126640115|ref|NC_009085.1| | 107351 | 107406 | 56 |
| exprReg_855 | gi|126640115|ref|NC_009085.1| | 107458 | 107480 | 23 |
| exprReg_856 | gi|126640115|ref|NC_009085.1| | 107498 | 107538 | 41 |
| exprReg_857 | gi|126640115|ref|NC_009085.1| | 107585 | 107624 | 40 |
| exprReg_858 | gi|126640115|ref|NC_009085.1| | 107646 | 107703 | 58 |
| exprReg_859 | gi|126640115|ref|NC_009085.1| | 107736 | 107753 | 18 |
| exprReg_860 | gi|126640115|ref|NC_009085.1| | 107852 | 107888 | 37 |
| exprReg_861 | gi|126640115|ref|NC_009085.1| | 107983 | 108024 | 42 |
| exprReg_887 | gi|126640115|ref|NC_009085.1| | 112510 | 112569 | 60 |
| exprReg_900 | gi|126640115|ref|NC_009085.1| | 114156 | 114263 | 108 |
| exprReg_910 | gi|126640115|ref|NC_009085.1| | 117323 | 117340 | 18 |
| exprReg_911 | gi|126640115|ref|NC_009085.1| | 117379 | 117396 | 18 |
| exprReg_924 | gi|126640115|ref|NC_009085.1| | 118869 | 118933 | 65 |
| exprReg_925 | gi|126640115|ref|NC_009085.1| | 118937 | 118980 | 44 |
| exprReg_926 | gi|126640115|ref|NC_009085.1| | 119037 | 119096 | 60 |
| exprReg_927 | gi|126640115|ref|NC_009085.1| | 119158 | 119239 | 82 |
| exprReg_943 | gi|126640115|ref|NC_009085.1| | 121535 | 121552 | 18 |
| exprReg_944 | gi|126640115|ref|NC_009085.1| | 121655 | 121728 | 74 |
| exprReg_945 | gi|126640115|ref|NC_009085.1| | 121755 | 121966 | 212 |
| exprReg_946 | gi|126640115|ref|NC_009085.1| | 121984 | 122179 | 196 |
| exprReg_947 | gi|126640115|ref|NC_009085.1| | 122188 | 122466 | 279 |
| exprReg_948 | gi|126640115|ref|NC_009085.1| | 122473 | 122507 | 35 |
| exprReg_949 | gi|126640115|ref|NC_009085.1| | 122542 | 122666 | 125 |
| exprReg_950 | gi|126640115|ref|NC_009085.1| | 122698 | 122840 | 143 |
| exprReg_956 | gi|126640115|ref|NC_009085.1| | 124156 | 124220 | 65 |
| exprReg_957 | gi|126640115|ref|NC_009085.1| | 124224 | 124253 | 30 |
| exprReg_980 | gi|126640115|ref|NC_009085.1| | 127793 | 127873 | 81 |
| exprReg_1001 | gi|126640115|ref|NC_009085.1| | 131421 | 131444 | 24 |
| exprReg_1002 | gi|126640115|ref|NC_009085.1| | 131833 | 131882 | 50 |
| exprReg_1003 | gi|126640115|ref|NC_009085.1| | 131884 | 131901 | 18 |
| exprReg_1004 | gi|126640115|ref|NC_009085.1| | 131905 | 131929 | 25 |
| exprReg_1005 | gi|126640115|ref|NC_009085.1| | 131950 | 131966 | 17 |
| exprReg_1006 | gi|126640115|ref|NC_009085.1| | 131990 | 132007 | 18 |
| exprReg_1007 | gi|126640115|ref|NC_009085.1| | 132081 | 132103 | 23 |
| exprReg_1013 | gi|126640115|ref|NC_009085.1| | 133027 | 133090 | 64 |
| exprReg_1014 | gi|126640115|ref|NC_009085.1| | 133293 | 133314 | 22 |
| exprReg_1015 | gi|126640115|ref|NC_009085.1| | 133444 | 133463 | 20 |
| exprReg_1016 | gi|126640115|ref|NC_009085.1| | 133505 | 133565 | 61 |
| exprReg_1017 | gi|126640115|ref|NC_009085.1| | 133593 | 133611 | 19 |
| exprReg_1033 | gi|126640115|ref|NC_009085.1| | 135446 | 135483 | 38 |
| exprReg_1106 | gi|126640115|ref|NC_009085.1| | 146456 | 146529 | 74 |
| exprReg_1119 | gi|126640115|ref|NC_009085.1| | 148432 | 148451 | 20 |
| exprReg_1128 | gi|126640115|ref|NC_009085.1| | 150369 | 150527 | 159 |
| exprReg_1136 | gi|126640115|ref|NC_009085.1| | 151616 | 151683 | 68 |
| exprReg_1137 | gi|126640115|ref|NC_009085.1| | 151725 | 151807 | 83 |
| exprReg_1145 | gi|126640115|ref|NC_009085.1| | 153130 | 153176 | 47 |
| exprReg_1146 | gi|126640115|ref|NC_009085.1| | 153231 | 153342 | 112 |
| exprReg_1147 | gi|126640115|ref|NC_009085.1| | 153363 | 153409 | 47 |
| exprReg_1163 | gi|126640115|ref|NC_009085.1| | 154928 | 154949 | 22 |
| exprReg_1164 | gi|126640115|ref|NC_009085.1| | 154968 | 154987 | 20 |
| exprReg_1165 | gi|126640115|ref|NC_009085.1| | 155014 | 155032 | 19 |
| exprReg_1166 | gi|126640115|ref|NC_009085.1| | 155059 | 155115 | 57 |
| exprReg_1167 | gi|126640115|ref|NC_009085.1| | 155287 | 155366 | 80 |
| exprReg_1168 | gi|126640115|ref|NC_009085.1| | 155453 | 155469 | 17 |
| exprReg_1184 | gi|126640115|ref|NC_009085.1| | 157971 | 157992 | 22 |
| exprReg_1185 | gi|126640115|ref|NC_009085.1| | 158008 | 158027 | 20 |
| exprReg_1186 | gi|126640115|ref|NC_009085.1| | 158033 | 158099 | 67 |
| exprReg_1194 | gi|126640115|ref|NC_009085.1| | 158943 | 159072 | 130 |
| exprReg_1195 | gi|126640115|ref|NC_009085.1| | 159130 | 159154 | 25 |
| exprReg_1196 | gi|126640115|ref|NC_009085.1| | 159158 | 159187 | 30 |
| exprReg_1220 | gi|126640115|ref|NC_009085.1| | 161840 | 161861 | 22 |
| exprReg_1221 | gi|126640115|ref|NC_009085.1| | 161963 | 162062 | 100 |
| exprReg_1222 | gi|126640115|ref|NC_009085.1| | 162067 | 162289 | 223 |
| exprReg_1223 | gi|126640115|ref|NC_009085.1| | 162292 | 162568 | 277 |
| exprReg_1224 | gi|126640115|ref|NC_009085.1| | 162599 | 162634 | 36 |
| exprReg_1225 | gi|126640115|ref|NC_009085.1| | 162646 | 162677 | 32 |
| exprReg_1226 | gi|126640115|ref|NC_009085.1| | 162732 | 163115 | 384 |
| exprReg_1227 | gi|126640115|ref|NC_009085.1| | 163144 | 163199 | 56 |
| exprReg_1228 | gi|126640115|ref|NC_009085.1| | 163314 | 163526 | 213 |
| exprReg_1229 | gi|126640115|ref|NC_009085.1| | 163578 | 163598 | 21 |
| exprReg_1230 | gi|126640115|ref|NC_009085.1| | 163606 | 163800 | 195 |
| exprReg_1231 | gi|126640115|ref|NC_009085.1| | 164224 | 164259 | 36 |
| exprReg_1245 | gi|126640115|ref|NC_009085.1| | 166819 | 166998 | 180 |
| exprReg_1246 | gi|126640115|ref|NC_009085.1| | 167017 | 167035 | 19 |
| exprReg_1247 | gi|126640115|ref|NC_009085.1| | 167078 | 167096 | 19 |
| exprReg_1259 | gi|126640115|ref|NC_009085.1| | 169821 | 169896 | 76 |
| exprReg_1260 | gi|126640115|ref|NC_009085.1| | 169908 | 169950 | 43 |
| exprReg_1261 | gi|126640115|ref|NC_009085.1| | 169983 | 170022 | 40 |
| exprReg_1262 | gi|126640115|ref|NC_009085.1| | 170077 | 170125 | 49 |
| exprReg_1271 | gi|126640115|ref|NC_009085.1| | 170792 | 170833 | 42 |
| exprReg_1272 | gi|126640115|ref|NC_009085.1| | 170846 | 170866 | 21 |
| exprReg_1273 | gi|126640115|ref|NC_009085.1| | 170873 | 170891 | 19 |
| exprReg_1287 | gi|126640115|ref|NC_009085.1| | 173248 | 173266 | 19 |
| exprReg_1323 | gi|126640115|ref|NC_009085.1| | 179355 | 179372 | 18 |
| exprReg_1324 | gi|126640115|ref|NC_009085.1| | 179396 | 179426 | 31 |
| exprReg_1337 | gi|126640115|ref|NC_009085.1| | 180859 | 180974 | 116 |
| exprReg_1348 | gi|126640115|ref|NC_009085.1| | 182047 | 182066 | 20 |
| exprReg_1349 | gi|126640115|ref|NC_009085.1| | 182079 | 182201 | 123 |
| exprReg_1350 | gi|126640115|ref|NC_009085.1| | 182321 | 182347 | 27 |
| exprReg_1360 | gi|126640115|ref|NC_009085.1| | 183102 | 183119 | 18 |
| exprReg_1361 | gi|126640115|ref|NC_009085.1| | 183209 | 183228 | 20 |
| exprReg_1366 | gi|126640115|ref|NC_009085.1| | 184085 | 184185 | 101 |
| exprReg_1367 | gi|126640115|ref|NC_009085.1| | 184194 | 184270 | 77 |
| exprReg_1368 | gi|126640115|ref|NC_009085.1| | 184272 | 184399 | 128 |
| exprReg_1383 | gi|126640115|ref|NC_009085.1| | 186120 | 186139 | 20 |
| exprReg_1393 | gi|126640115|ref|NC_009085.1| | 188523 | 188573 | 51 |
| exprReg_1394 | gi|126640115|ref|NC_009085.1| | 188617 | 188640 | 24 |
| exprReg_1395 | gi|126640115|ref|NC_009085.1| | 188646 | 188664 | 19 |
| exprReg_1396 | gi|126640115|ref|NC_009085.1| | 188679 | 188749 | 71 |
| exprReg_1413 | gi|126640115|ref|NC_009085.1| | 192122 | 192199 | 78 |
| exprReg_1414 | gi|126640115|ref|NC_009085.1| | 192298 | 192418 | 121 |
| exprReg_1417 | gi|126640115|ref|NC_009085.1| | 198813 | 198829 | 17 |
| exprReg_1421 | gi|126640115|ref|NC_009085.1| | 199524 | 199553 | 30 |
| exprReg_1422 | gi|126640115|ref|NC_009085.1| | 199614 | 199636 | 23 |
| exprReg_1423 | gi|126640115|ref|NC_009085.1| | 199691 | 199722 | 32 |
| exprReg_1434 | gi|126640115|ref|NC_009085.1| | 201308 | 201345 | 38 |
| exprReg_1435 | gi|126640115|ref|NC_009085.1| | 201350 | 201426 | 77 |
| exprReg_1436 | gi|126640115|ref|NC_009085.1| | 201460 | 201506 | 47 |
| exprReg_1443 | gi|126640115|ref|NC_009085.1| | 203105 | 203126 | 22 |
| exprReg_1444 | gi|126640115|ref|NC_009085.1| | 203232 | 203249 | 18 |
| exprReg_1445 | gi|126640115|ref|NC_009085.1| | 203393 | 203461 | 69 |
| exprReg_1449 | gi|126640115|ref|NC_009085.1| | 204506 | 204530 | 25 |
| exprReg_1469 | gi|126640115|ref|NC_009085.1| | 207201 | 207218 | 18 |
| exprReg_1470 | gi|126640115|ref|NC_009085.1| | 207249 | 207315 | 67 |
| exprReg_1475 | gi|126640115|ref|NC_009085.1| | 207986 | 208002 | 17 |
| exprReg_1482 | gi|126640115|ref|NC_009085.1| | 208428 | 208444 | 17 |
| exprReg_1511 | gi|126640115|ref|NC_009085.1| | 211931 | 211955 | 25 |
| exprReg_1512 | gi|126640115|ref|NC_009085.1| | 211972 | 211990 | 19 |
| exprReg_1513 | gi|126640115|ref|NC_009085.1| | 212128 | 212185 | 58 |
| exprReg_1519 | gi|126640115|ref|NC_009085.1| | 212748 | 212821 | 74 |
| exprReg_1520 | gi|126640115|ref|NC_009085.1| | 212988 | 213040 | 53 |
| exprReg_1521 | gi|126640115|ref|NC_009085.1| | 213076 | 213230 | 155 |
| exprReg_1522 | gi|126640115|ref|NC_009085.1| | 213238 | 213278 | 41 |
| exprReg_1544 | gi|126640115|ref|NC_009085.1| | 215973 | 216023 | 51 |
| exprReg_1545 | gi|126640115|ref|NC_009085.1| | 216030 | 216047 | 18 |
| exprReg_1554 | gi|126640115|ref|NC_009085.1| | 217419 | 217537 | 119 |
| exprReg_1555 | gi|126640115|ref|NC_009085.1| | 217539 | 217565 | 27 |
| exprReg_1556 | gi|126640115|ref|NC_009085.1| | 217589 | 217661 | 73 |
| exprReg_1557 | gi|126640115|ref|NC_009085.1| | 217682 | 217704 | 23 |
| exprReg_1558 | gi|126640115|ref|NC_009085.1| | 217716 | 217807 | 92 |
| exprReg_1559 | gi|126640115|ref|NC_009085.1| | 217832 | 217952 | 121 |
| exprReg_1560 | gi|126640115|ref|NC_009085.1| | 217955 | 218133 | 179 |
| exprReg_1561 | gi|126640115|ref|NC_009085.1| | 218158 | 218273 | 116 |
| exprReg_1562 | gi|126640115|ref|NC_009085.1| | 218289 | 218479 | 191 |
| exprReg_1563 | gi|126640115|ref|NC_009085.1| | 218483 | 218500 | 18 |
| exprReg_1564 | gi|126640115|ref|NC_009085.1| | 218525 | 218646 | 122 |
| exprReg_1565 | gi|126640115|ref|NC_009085.1| | 218681 | 218700 | 20 |
| exprReg_1566 | gi|126640115|ref|NC_009085.1| | 218716 | 218892 | 177 |
| exprReg_1567 | gi|126640115|ref|NC_009085.1| | 218944 | 219449 | 506 |
| exprReg_1568 | gi|126640115|ref|NC_009085.1| | 219612 | 219882 | 271 |
| exprReg_1569 | gi|126640115|ref|NC_009085.1| | 219949 | 219972 | 24 |
| exprReg_1570 | gi|126640115|ref|NC_009085.1| | 219979 | 220068 | 90 |
| exprReg_1571 | gi|126640115|ref|NC_009085.1| | 220077 | 220117 | 41 |
| exprReg_1572 | gi|126640115|ref|NC_009085.1| | 220149 | 220537 | 389 |
| exprReg_1573 | gi|126640115|ref|NC_009085.1| | 220563 | 220639 | 77 |
| exprReg_1574 | gi|126640115|ref|NC_009085.1| | 220658 | 220744 | 87 |
| exprReg_1575 | gi|126640115|ref|NC_009085.1| | 220750 | 220826 | 77 |
| exprReg_1576 | gi|126640115|ref|NC_009085.1| | 220846 | 220933 | 88 |
| exprReg_1577 | gi|126640115|ref|NC_009085.1| | 220968 | 221126 | 159 |
| exprReg_1578 | gi|126640115|ref|NC_009085.1| | 221149 | 221281 | 133 |
| exprReg_1579 | gi|126640115|ref|NC_009085.1| | 221342 | 221425 | 84 |
| exprReg_1580 | gi|126640115|ref|NC_009085.1| | 221437 | 221614 | 178 |
| exprReg_1581 | gi|126640115|ref|NC_009085.1| | 221662 | 221719 | 58 |
| exprReg_1602 | gi|126640115|ref|NC_009085.1| | 224713 | 224734 | 22 |
| exprReg_1603 | gi|126640115|ref|NC_009085.1| | 225309 | 225360 | 52 |
| exprReg_1635 | gi|126640115|ref|NC_009085.1| | 229764 | 229978 | 215 |
| exprReg_1636 | gi|126640115|ref|NC_009085.1| | 230007 | 230135 | 129 |
| exprReg_1637 | gi|126640115|ref|NC_009085.1| | 230198 | 230264 | 67 |
| exprReg_1648 | gi|126640115|ref|NC_009085.1| | 232035 | 232062 | 28 |
| exprReg_1669 | gi|126640115|ref|NC_009085.1| | 236370 | 236423 | 54 |
| exprReg_1678 | gi|126640115|ref|NC_009085.1| | 237752 | 237782 | 31 |
| exprReg_1679 | gi|126640115|ref|NC_009085.1| | 237844 | 237891 | 48 |
| exprReg_1680 | gi|126640115|ref|NC_009085.1| | 238029 | 238112 | 84 |
| exprReg_1681 | gi|126640115|ref|NC_009085.1| | 238126 | 238282 | 157 |
| exprReg_1682 | gi|126640115|ref|NC_009085.1| | 238287 | 238334 | 48 |
| exprReg_1683 | gi|126640115|ref|NC_009085.1| | 238401 | 238468 | 68 |
| exprReg_1684 | gi|126640115|ref|NC_009085.1| | 238471 | 238489 | 19 |
| exprReg_1685 | gi|126640115|ref|NC_009085.1| | 238530 | 238600 | 71 |
| exprReg_1686 | gi|126640115|ref|NC_009085.1| | 238606 | 238627 | 22 |
| exprReg_1687 | gi|126640115|ref|NC_009085.1| | 238689 | 238705 | 17 |
| exprReg_1688 | gi|126640115|ref|NC_009085.1| | 238733 | 238764 | 32 |
| exprReg_1689 | gi|126640115|ref|NC_009085.1| | 238774 | 238807 | 34 |
| exprReg_1703 | gi|126640115|ref|NC_009085.1| | 241565 | 241591 | 27 |
| exprReg_1704 | gi|126640115|ref|NC_009085.1| | 241704 | 241730 | 27 |
| exprReg_1705 | gi|126640115|ref|NC_009085.1| | 241742 | 241760 | 19 |
| exprReg_1706 | gi|126640115|ref|NC_009085.1| | 241779 | 241841 | 63 |
| exprReg_1707 | gi|126640115|ref|NC_009085.1| | 241862 | 241950 | 89 |
| exprReg_1708 | gi|126640115|ref|NC_009085.1| | 241973 | 242007 | 35 |
| exprReg_1709 | gi|126640115|ref|NC_009085.1| | 242067 | 242084 | 18 |
| exprReg_1710 | gi|126640115|ref|NC_009085.1| | 242127 | 242330 | 204 |
| exprReg_1711 | gi|126640115|ref|NC_009085.1| | 242350 | 242409 | 60 |
| exprReg_1712 | gi|126640115|ref|NC_009085.1| | 242428 | 242449 | 22 |
| exprReg_1713 | gi|126640115|ref|NC_009085.1| | 242501 | 242560 | 60 |
| exprReg_1714 | gi|126640115|ref|NC_009085.1| | 242573 | 242720 | 148 |
| exprReg_1715 | gi|126640115|ref|NC_009085.1| | 242763 | 242818 | 56 |
| exprReg_1716 | gi|126640115|ref|NC_009085.1| | 242835 | 242873 | 39 |
| exprReg_1717 | gi|126640115|ref|NC_009085.1| | 242878 | 242968 | 91 |
| exprReg_1718 | gi|126640115|ref|NC_009085.1| | 242978 | 243069 | 92 |
| exprReg_1719 | gi|126640115|ref|NC_009085.1| | 243081 | 243438 | 358 |
| exprReg_1720 | gi|126640115|ref|NC_009085.1| | 243455 | 243541 | 87 |
| exprReg_1721 | gi|126640115|ref|NC_009085.1| | 243665 | 243784 | 120 |
| exprReg_1722 | gi|126640115|ref|NC_009085.1| | 243794 | 243817 | 24 |
| exprReg_1723 | gi|126640115|ref|NC_009085.1| | 243881 | 243953 | 73 |
| exprReg_1724 | gi|126640115|ref|NC_009085.1| | 243998 | 244024 | 27 |
| exprReg_1725 | gi|126640115|ref|NC_009085.1| | 244048 | 244065 | 18 |
| exprReg_1726 | gi|126640115|ref|NC_009085.1| | 244095 | 244126 | 32 |
| exprReg_1727 | gi|126640115|ref|NC_009085.1| | 244148 | 244303 | 156 |
| exprReg_1731 | gi|126640115|ref|NC_009085.1| | 244623 | 244661 | 39 |
| exprReg_1732 | gi|126640115|ref|NC_009085.1| | 244737 | 244755 | 19 |
| exprReg_1733 | gi|126640115|ref|NC_009085.1| | 244777 | 244820 | 44 |
| exprReg_1734 | gi|126640115|ref|NC_009085.1| | 244834 | 244860 | 27 |
| exprReg_1735 | gi|126640115|ref|NC_009085.1| | 244867 | 244906 | 40 |
| exprReg_1747 | gi|126640115|ref|NC_009085.1| | 247317 | 247420 | 104 |
| exprReg_1753 | gi|126640115|ref|NC_009085.1| | 248415 | 248556 | 142 |
| exprReg_1765 | gi|126640115|ref|NC_009085.1| | 250386 | 250412 | 27 |
| exprReg_1768 | gi|126640115|ref|NC_009085.1| | 250729 | 250775 | 47 |
| exprReg_1774 | gi|126640115|ref|NC_009085.1| | 251375 | 251459 | 85 |
| exprReg_1805 | gi|126640115|ref|NC_009085.1| | 255844 | 255868 | 25 |
| exprReg_1843 | gi|126640115|ref|NC_009085.1| | 261307 | 261323 | 17 |
| exprReg_1844 | gi|126640115|ref|NC_009085.1| | 261352 | 261450 | 99 |
| exprReg_1845 | gi|126640115|ref|NC_009085.1| | 261455 | 261474 | 20 |
| exprReg_1872 | gi|126640115|ref|NC_009085.1| | 266042 | 266059 | 18 |
| exprReg_1873 | gi|126640115|ref|NC_009085.1| | 266216 | 266232 | 17 |
| exprReg_1878 | gi|126640115|ref|NC_009085.1| | 266941 | 266964 | 24 |
| exprReg_1879 | gi|126640115|ref|NC_009085.1| | 267103 | 267124 | 22 |
| exprReg_1884 | gi|126640115|ref|NC_009085.1| | 268025 | 268160 | 136 |
| exprReg_1885 | gi|126640115|ref|NC_009085.1| | 268219 | 268253 | 35 |
| exprReg_1913 | gi|126640115|ref|NC_009085.1| | 273498 | 273521 | 24 |
| exprReg_1921 | gi|126640115|ref|NC_009085.1| | 275078 | 275099 | 22 |
| exprReg_1948 | gi|126640115|ref|NC_009085.1| | 278499 | 278534 | 36 |
| exprReg_1949 | gi|126640115|ref|NC_009085.1| | 278589 | 278682 | 94 |
| exprReg_1959 | gi|126640115|ref|NC_009085.1| | 279810 | 279829 | 20 |
| exprReg_1960 | gi|126640115|ref|NC_009085.1| | 280080 | 280115 | 36 |
| exprReg_1965 | gi|126640115|ref|NC_009085.1| | 280641 | 280728 | 88 |
| exprReg_1988 | gi|126640115|ref|NC_009085.1| | 282952 | 283004 | 53 |
| exprReg_1994 | gi|126640115|ref|NC_009085.1| | 283843 | 283983 | 141 |
| exprReg_2005 | gi|126640115|ref|NC_009085.1| | 285684 | 285752 | 69 |
| exprReg_2006 | gi|126640115|ref|NC_009085.1| | 285754 | 285836 | 83 |
| exprReg_2020 | gi|126640115|ref|NC_009085.1| | 288898 | 288919 | 22 |
| exprReg_2046 | gi|126640115|ref|NC_009085.1| | 292083 | 292142 | 60 |
| exprReg_2050 | gi|126640115|ref|NC_009085.1| | 292642 | 292724 | 83 |
| exprReg_2051 | gi|126640115|ref|NC_009085.1| | 292820 | 292862 | 43 |
| exprReg_2058 | gi|126640115|ref|NC_009085.1| | 293650 | 293752 | 103 |
| exprReg_2067 | gi|126640115|ref|NC_009085.1| | 294489 | 294624 | 136 |
| exprReg_2068 | gi|126640115|ref|NC_009085.1| | 294671 | 294703 | 33 |
| exprReg_2089 | gi|126640115|ref|NC_009085.1| | 298394 | 298442 | 49 |
| exprReg_2103 | gi|126640115|ref|NC_009085.1| | 300323 | 300358 | 36 |
| exprReg_2113 | gi|126640115|ref|NC_009085.1| | 302945 | 303026 | 82 |
| exprReg_2118 | gi|126640115|ref|NC_009085.1| | 304096 | 304195 | 100 |
| exprReg_2119 | gi|126640115|ref|NC_009085.1| | 304207 | 304365 | 159 |
| exprReg_2125 | gi|126640115|ref|NC_009085.1| | 305396 | 305416 | 21 |
| exprReg_2181 | gi|126640115|ref|NC_009085.1| | 314123 | 314235 | 113 |
| exprReg_2182 | gi|126640115|ref|NC_009085.1| | 314255 | 314481 | 227 |
| exprReg_2183 | gi|126640115|ref|NC_009085.1| | 314696 | 315048 | 353 |
| exprReg_2189 | gi|126640115|ref|NC_009085.1| | 315551 | 315588 | 38 |
| exprReg_2190 | gi|126640115|ref|NC_009085.1| | 315614 | 315651 | 38 |
| exprReg_2191 | gi|126640115|ref|NC_009085.1| | 315693 | 315791 | 99 |
| exprReg_2192 | gi|126640115|ref|NC_009085.1| | 315798 | 315916 | 119 |
| exprReg_2197 | gi|126640115|ref|NC_009085.1| | 316677 | 316763 | 87 |
| exprReg_2198 | gi|126640115|ref|NC_009085.1| | 316812 | 316884 | 73 |
| exprReg_2199 | gi|126640115|ref|NC_009085.1| | 316904 | 316925 | 22 |
| exprReg_2211 | gi|126640115|ref|NC_009085.1| | 318159 | 318202 | 44 |
| exprReg_2213 | gi|126640115|ref|NC_009085.1| | 319207 | 319224 | 18 |
| exprReg_2214 | gi|126640115|ref|NC_009085.1| | 319235 | 319260 | 26 |
| exprReg_2217 | gi|126640115|ref|NC_009085.1| | 319612 | 319630 | 19 |
| exprReg_2218 | gi|126640115|ref|NC_009085.1| | 319675 | 319702 | 28 |
| exprReg_2219 | gi|126640115|ref|NC_009085.1| | 319723 | 319739 | 17 |
| exprReg_2220 | gi|126640115|ref|NC_009085.1| | 319750 | 319772 | 23 |
| exprReg_2221 | gi|126640115|ref|NC_009085.1| | 319827 | 319849 | 23 |
| exprReg_2222 | gi|126640115|ref|NC_009085.1| | 319956 | 320113 | 158 |
| exprReg_2223 | gi|126640115|ref|NC_009085.1| | 320119 | 320211 | 93 |
| exprReg_2224 | gi|126640115|ref|NC_009085.1| | 320343 | 320430 | 88 |
| exprReg_2225 | gi|126640115|ref|NC_009085.1| | 320432 | 320584 | 153 |
| exprReg_2226 | gi|126640115|ref|NC_009085.1| | 320633 | 320656 | 24 |
| exprReg_2227 | gi|126640115|ref|NC_009085.1| | 320698 | 320814 | 117 |
| exprReg_2228 | gi|126640115|ref|NC_009085.1| | 320906 | 320926 | 21 |
| exprReg_2229 | gi|126640115|ref|NC_009085.1| | 320979 | 320998 | 20 |
| exprReg_2230 | gi|126640115|ref|NC_009085.1| | 321118 | 321237 | 120 |
| exprReg_2231 | gi|126640115|ref|NC_009085.1| | 321314 | 321342 | 29 |
| exprReg_2232 | gi|126640115|ref|NC_009085.1| | 321408 | 321575 | 168 |
| exprReg_2247 | gi|126640115|ref|NC_009085.1| | 323784 | 323872 | 89 |
| exprReg_2248 | gi|126640115|ref|NC_009085.1| | 323999 | 324109 | 111 |
| exprReg_2251 | gi|126640115|ref|NC_009085.1| | 324748 | 325159 | 412 |
| exprReg_2252 | gi|126640115|ref|NC_009085.1| | 325167 | 325190 | 24 |
| exprReg_2263 | gi|126640115|ref|NC_009085.1| | 326370 | 326389 | 20 |
| exprReg_2292 | gi|126640115|ref|NC_009085.1| | 332796 | 332893 | 98 |
| exprReg_2293 | gi|126640115|ref|NC_009085.1| | 332895 | 333480 | 586 |
| exprReg_2294 | gi|126640115|ref|NC_009085.1| | 333554 | 333579 | 26 |
| exprReg_2295 | gi|126640115|ref|NC_009085.1| | 333694 | 333714 | 21 |
| exprReg_2296 | gi|126640115|ref|NC_009085.1| | 333719 | 333739 | 21 |
| exprReg_2297 | gi|126640115|ref|NC_009085.1| | 333777 | 333858 | 82 |
| exprReg_2298 | gi|126640115|ref|NC_009085.1| | 333881 | 334014 | 134 |
| exprReg_2299 | gi|126640115|ref|NC_009085.1| | 334066 | 334126 | 61 |
| exprReg_2300 | gi|126640115|ref|NC_009085.1| | 334139 | 334233 | 95 |
| exprReg_2301 | gi|126640115|ref|NC_009085.1| | 334303 | 334583 | 281 |
| exprReg_2302 | gi|126640115|ref|NC_009085.1| | 334585 | 334608 | 24 |
| exprReg_2303 | gi|126640115|ref|NC_009085.1| | 334645 | 334855 | 211 |
| exprReg_2304 | gi|126640115|ref|NC_009085.1| | 334867 | 335065 | 199 |
| exprReg_2305 | gi|126640115|ref|NC_009085.1| | 335128 | 335183 | 56 |
| exprReg_2306 | gi|126640115|ref|NC_009085.1| | 335490 | 335606 | 117 |
| exprReg_2313 | gi|126640115|ref|NC_009085.1| | 336567 | 336603 | 37 |
| exprReg_2339 | gi|126640115|ref|NC_009085.1| | 340772 | 340933 | 162 |
| exprReg_2340 | gi|126640115|ref|NC_009085.1| | 340942 | 341014 | 73 |
| exprReg_2341 | gi|126640115|ref|NC_009085.1| | 341054 | 341106 | 53 |
| exprReg_2342 | gi|126640115|ref|NC_009085.1| | 341159 | 341182 | 24 |
| exprReg_2343 | gi|126640115|ref|NC_009085.1| | 341302 | 341451 | 150 |
| exprReg_2344 | gi|126640115|ref|NC_009085.1| | 341477 | 341583 | 107 |
| exprReg_2345 | gi|126640115|ref|NC_009085.1| | 341597 | 341666 | 70 |
| exprReg_2346 | gi|126640115|ref|NC_009085.1| | 341720 | 341749 | 30 |
| exprReg_2350 | gi|126640115|ref|NC_009085.1| | 342271 | 342369 | 99 |
| exprReg_2355 | gi|126640115|ref|NC_009085.1| | 342972 | 342993 | 22 |
| exprReg_2356 | gi|126640115|ref|NC_009085.1| | 343024 | 343044 | 21 |
| exprReg_2357 | gi|126640115|ref|NC_009085.1| | 343071 | 343316 | 246 |
| exprReg_2358 | gi|126640115|ref|NC_009085.1| | 343331 | 343365 | 35 |
| exprReg_2359 | gi|126640115|ref|NC_009085.1| | 343455 | 343514 | 60 |
| exprReg_2360 | gi|126640115|ref|NC_009085.1| | 343550 | 343598 | 49 |
| exprReg_2361 | gi|126640115|ref|NC_009085.1| | 343607 | 343641 | 35 |
| exprReg_2362 | gi|126640115|ref|NC_009085.1| | 343732 | 343763 | 32 |
| exprReg_2374 | gi|126640115|ref|NC_009085.1| | 345864 | 345894 | 31 |
| exprReg_2384 | gi|126640115|ref|NC_009085.1| | 348114 | 348174 | 61 |
| exprReg_2396 | gi|126640115|ref|NC_009085.1| | 349981 | 350018 | 38 |
| exprReg_2408 | gi|126640115|ref|NC_009085.1| | 351371 | 351432 | 62 |
| exprReg_2409 | gi|126640115|ref|NC_009085.1| | 351462 | 351478 | 17 |
| exprReg_2441 | gi|126640115|ref|NC_009085.1| | 356174 | 356191 | 18 |
| exprReg_2450 | gi|126640115|ref|NC_009085.1| | 357916 | 357966 | 51 |
| exprReg_2451 | gi|126640115|ref|NC_009085.1| | 358034 | 358053 | 20 |
| exprReg_2461 | gi|126640115|ref|NC_009085.1| | 359739 | 359888 | 150 |
| exprReg_2468 | gi|126640115|ref|NC_009085.1| | 360513 | 360544 | 32 |
| exprReg_2479 | gi|126640115|ref|NC_009085.1| | 362270 | 362525 | 256 |
| exprReg_2480 | gi|126640115|ref|NC_009085.1| | 362543 | 362793 | 251 |
| exprReg_2481 | gi|126640115|ref|NC_009085.1| | 362800 | 362821 | 22 |
| exprReg_2482 | gi|126640115|ref|NC_009085.1| | 362928 | 362946 | 19 |
| exprReg_2483 | gi|126640115|ref|NC_009085.1| | 362948 | 363017 | 70 |
| exprReg_2484 | gi|126640115|ref|NC_009085.1| | 363052 | 363288 | 237 |
| exprReg_2485 | gi|126640115|ref|NC_009085.1| | 363310 | 363417 | 108 |
| exprReg_2486 | gi|126640115|ref|NC_009085.1| | 363454 | 363474 | 21 |
| exprReg_2487 | gi|126640115|ref|NC_009085.1| | 363541 | 363558 | 18 |
| exprReg_2488 | gi|126640115|ref|NC_009085.1| | 363567 | 363732 | 166 |
| exprReg_2489 | gi|126640115|ref|NC_009085.1| | 363793 | 363823 | 31 |
| exprReg_2490 | gi|126640115|ref|NC_009085.1| | 363945 | 363961 | 17 |
| exprReg_2491 | gi|126640115|ref|NC_009085.1| | 363982 | 364056 | 75 |
| exprReg_2492 | gi|126640115|ref|NC_009085.1| | 364098 | 364184 | 87 |
| exprReg_2493 | gi|126640115|ref|NC_009085.1| | 364228 | 364333 | 106 |
| exprReg_2494 | gi|126640115|ref|NC_009085.1| | 364382 | 364527 | 146 |
| exprReg_2495 | gi|126640115|ref|NC_009085.1| | 364598 | 364714 | 117 |
| exprReg_2498 | gi|126640115|ref|NC_009085.1| | 365028 | 365048 | 21 |
| exprReg_2501 | gi|126640115|ref|NC_009085.1| | 365545 | 365574 | 30 |
| exprReg_2522 | gi|126640115|ref|NC_009085.1| | 368384 | 368401 | 18 |
| exprReg_2535 | gi|126640115|ref|NC_009085.1| | 371342 | 371416 | 75 |
| exprReg_2536 | gi|126640115|ref|NC_009085.1| | 371424 | 371479 | 56 |
| exprReg_2537 | gi|126640115|ref|NC_009085.1| | 371533 | 371552 | 20 |
| exprReg_2564 | gi|126640115|ref|NC_009085.1| | 375392 | 375417 | 26 |
| exprReg_2565 | gi|126640115|ref|NC_009085.1| | 375438 | 375473 | 36 |
| exprReg_2574 | gi|126640115|ref|NC_009085.1| | 376696 | 376722 | 27 |
| exprReg_2575 | gi|126640115|ref|NC_009085.1| | 376735 | 376761 | 27 |
| exprReg_2576 | gi|126640115|ref|NC_009085.1| | 376777 | 376852 | 76 |
| exprReg_2596 | gi|126640115|ref|NC_009085.1| | 380197 | 380277 | 81 |
| exprReg_2603 | gi|126640115|ref|NC_009085.1| | 381163 | 381179 | 17 |
| exprReg_2635 | gi|126640115|ref|NC_009085.1| | 386294 | 386365 | 72 |
| exprReg_2674 | gi|126640115|ref|NC_009085.1| | 392610 | 392627 | 18 |
| exprReg_2675 | gi|126640115|ref|NC_009085.1| | 392646 | 392809 | 164 |
| exprReg_2676 | gi|126640115|ref|NC_009085.1| | 393029 | 393047 | 19 |
| exprReg_2677 | gi|126640115|ref|NC_009085.1| | 393097 | 393243 | 147 |
| exprReg_2686 | gi|126640115|ref|NC_009085.1| | 394497 | 394577 | 81 |
| exprReg_2687 | gi|126640115|ref|NC_009085.1| | 394609 | 394742 | 134 |
| exprReg_2690 | gi|126640115|ref|NC_009085.1| | 395251 | 395280 | 30 |
| exprReg_2691 | gi|126640115|ref|NC_009085.1| | 395330 | 395357 | 28 |
| exprReg_2710 | gi|126640115|ref|NC_009085.1| | 398702 | 398720 | 19 |
| exprReg_2711 | gi|126640115|ref|NC_009085.1| | 398734 | 398921 | 188 |
| exprReg_2713 | gi|126640115|ref|NC_009085.1| | 399212 | 399232 | 21 |
| exprReg_2717 | gi|126640115|ref|NC_009085.1| | 400149 | 400168 | 20 |
| exprReg_2718 | gi|126640115|ref|NC_009085.1| | 400173 | 400211 | 39 |
| exprReg_2719 | gi|126640115|ref|NC_009085.1| | 400213 | 400320 | 108 |
| exprReg_2720 | gi|126640115|ref|NC_009085.1| | 400361 | 400384 | 24 |
| exprReg_2725 | gi|126640115|ref|NC_009085.1| | 401071 | 401120 | 50 |
| exprReg_2765 | gi|126640115|ref|NC_009085.1| | 406153 | 406228 | 76 |
| exprReg_2766 | gi|126640115|ref|NC_009085.1| | 406231 | 406340 | 110 |
| exprReg_2767 | gi|126640115|ref|NC_009085.1| | 406356 | 406483 | 128 |
| exprReg_2778 | gi|126640115|ref|NC_009085.1| | 408291 | 408307 | 17 |
| exprReg_2779 | gi|126640115|ref|NC_009085.1| | 408329 | 408352 | 24 |
| exprReg_2805 | gi|126640115|ref|NC_009085.1| | 411659 | 411684 | 26 |
| exprReg_2806 | gi|126640115|ref|NC_009085.1| | 411692 | 411804 | 113 |
| exprReg_2810 | gi|126640115|ref|NC_009085.1| | 412516 | 412538 | 23 |
| exprReg_2811 | gi|126640115|ref|NC_009085.1| | 412784 | 412829 | 46 |
| exprReg_2812 | gi|126640115|ref|NC_009085.1| | 412863 | 413073 | 211 |
| exprReg_2813 | gi|126640115|ref|NC_009085.1| | 413144 | 413164 | 21 |
| exprReg_2814 | gi|126640115|ref|NC_009085.1| | 413180 | 413196 | 17 |
| exprReg_2815 | gi|126640115|ref|NC_009085.1| | 413216 | 413235 | 20 |
| exprReg_2816 | gi|126640115|ref|NC_009085.1| | 413264 | 413287 | 24 |
| exprReg_2817 | gi|126640115|ref|NC_009085.1| | 413300 | 413341 | 42 |
| exprReg_2818 | gi|126640115|ref|NC_009085.1| | 413390 | 413429 | 40 |
| exprReg_2829 | gi|126640115|ref|NC_009085.1| | 415429 | 415445 | 17 |
| exprReg_2839 | gi|126640115|ref|NC_009085.1| | 417566 | 417653 | 88 |
| exprReg_2844 | gi|126640115|ref|NC_009085.1| | 418505 | 418522 | 18 |
| exprReg_2849 | gi|126640115|ref|NC_009085.1| | 419352 | 419398 | 47 |
| exprReg_2850 | gi|126640115|ref|NC_009085.1| | 419403 | 419422 | 20 |
| exprReg_2851 | gi|126640115|ref|NC_009085.1| | 419424 | 419444 | 21 |
| exprReg_2859 | gi|126640115|ref|NC_009085.1| | 421251 | 421269 | 19 |
| exprReg_2871 | gi|126640115|ref|NC_009085.1| | 422457 | 422598 | 142 |
| exprReg_2872 | gi|126640115|ref|NC_009085.1| | 422652 | 422726 | 75 |
| exprReg_2875 | gi|126640115|ref|NC_009085.1| | 423349 | 423401 | 53 |
| exprReg_2888 | gi|126640115|ref|NC_009085.1| | 424960 | 424980 | 21 |
| exprReg_2889 | gi|126640115|ref|NC_009085.1| | 424994 | 425046 | 53 |
| exprReg_2890 | gi|126640115|ref|NC_009085.1| | 425075 | 425095 | 21 |
| exprReg_2891 | gi|126640115|ref|NC_009085.1| | 425304 | 425552 | 249 |
| exprReg_2892 | gi|126640115|ref|NC_009085.1| | 425564 | 425594 | 31 |
| exprReg_2897 | gi|126640115|ref|NC_009085.1| | 426800 | 426834 | 35 |
| exprReg_2919 | gi|126640115|ref|NC_009085.1| | 429814 | 429935 | 122 |
| exprReg_2920 | gi|126640115|ref|NC_009085.1| | 429975 | 430035 | 61 |
| exprReg_2933 | gi|126640115|ref|NC_009085.1| | 432047 | 432095 | 49 |
| exprReg_2934 | gi|126640115|ref|NC_009085.1| | 432111 | 432134 | 24 |
| exprReg_2987 | gi|126640115|ref|NC_009085.1| | 438574 | 438608 | 35 |
| exprReg_2988 | gi|126640115|ref|NC_009085.1| | 438612 | 439314 | 703 |
| exprReg_2989 | gi|126640115|ref|NC_009085.1| | 439398 | 439418 | 21 |
| exprReg_2990 | gi|126640115|ref|NC_009085.1| | 439426 | 439456 | 31 |
| exprReg_2991 | gi|126640115|ref|NC_009085.1| | 439493 | 439546 | 54 |
| exprReg_2992 | gi|126640115|ref|NC_009085.1| | 439548 | 439749 | 202 |
| exprReg_2993 | gi|126640115|ref|NC_009085.1| | 439751 | 439821 | 71 |
| exprReg_2994 | gi|126640115|ref|NC_009085.1| | 439827 | 439845 | 19 |
| exprReg_2995 | gi|126640115|ref|NC_009085.1| | 439867 | 439901 | 35 |
| exprReg_2996 | gi|126640115|ref|NC_009085.1| | 439915 | 439943 | 29 |
| exprReg_2997 | gi|126640115|ref|NC_009085.1| | 440009 | 440074 | 66 |
| exprReg_2998 | gi|126640115|ref|NC_009085.1| | 440151 | 440335 | 185 |
| exprReg_3004 | gi|126640115|ref|NC_009085.1| | 441048 | 441073 | 26 |
| exprReg_3005 | gi|126640115|ref|NC_009085.1| | 441077 | 441146 | 70 |
| exprReg_3011 | gi|126640115|ref|NC_009085.1| | 441789 | 441806 | 18 |
| exprReg_3012 | gi|126640115|ref|NC_009085.1| | 441811 | 441827 | 17 |
| exprReg_3020 | gi|126640115|ref|NC_009085.1| | 443110 | 443173 | 64 |
| exprReg_3027 | gi|126640115|ref|NC_009085.1| | 444233 | 444359 | 127 |
| exprReg_3028 | gi|126640115|ref|NC_009085.1| | 444588 | 444770 | 183 |
| exprReg_3029 | gi|126640115|ref|NC_009085.1| | 444797 | 444813 | 17 |
| exprReg_3030 | gi|126640115|ref|NC_009085.1| | 444886 | 444910 | 25 |
| exprReg_3031 | gi|126640115|ref|NC_009085.1| | 444932 | 444983 | 52 |
| exprReg_3032 | gi|126640115|ref|NC_009085.1| | 445017 | 445034 | 18 |
| exprReg_3033 | gi|126640115|ref|NC_009085.1| | 445042 | 445136 | 95 |
| exprReg_3034 | gi|126640115|ref|NC_009085.1| | 445173 | 445198 | 26 |
| exprReg_3051 | gi|126640115|ref|NC_009085.1| | 448232 | 448352 | 121 |
| exprReg_3052 | gi|126640115|ref|NC_009085.1| | 448355 | 448372 | 18 |
| exprReg_3053 | gi|126640115|ref|NC_009085.1| | 448384 | 448431 | 48 |
| exprReg_3054 | gi|126640115|ref|NC_009085.1| | 448465 | 448525 | 61 |
| exprReg_3082 | gi|126640115|ref|NC_009085.1| | 453451 | 453499 | 49 |
| exprReg_3083 | gi|126640115|ref|NC_009085.1| | 453668 | 453701 | 34 |
| exprReg_3084 | gi|126640115|ref|NC_009085.1| | 453718 | 453748 | 31 |
| exprReg_3085 | gi|126640115|ref|NC_009085.1| | 453775 | 453794 | 20 |
| exprReg_3113 | gi|126640115|ref|NC_009085.1| | 458098 | 458114 | 17 |
| exprReg_3114 | gi|126640115|ref|NC_009085.1| | 458116 | 458358 | 243 |
| exprReg_3115 | gi|126640115|ref|NC_009085.1| | 458527 | 458551 | 25 |
| exprReg_3116 | gi|126640115|ref|NC_009085.1| | 458583 | 458600 | 18 |
| exprReg_3117 | gi|126640115|ref|NC_009085.1| | 458616 | 458693 | 78 |
| exprReg_3118 | gi|126640115|ref|NC_009085.1| | 458749 | 458766 | 18 |
| exprReg_3119 | gi|126640115|ref|NC_009085.1| | 458768 | 458791 | 24 |
| exprReg_3123 | gi|126640115|ref|NC_009085.1| | 459311 | 459535 | 225 |
| exprReg_3124 | gi|126640115|ref|NC_009085.1| | 459573 | 459694 | 122 |
| exprReg_3130 | gi|126640115|ref|NC_009085.1| | 461206 | 461240 | 35 |
| exprReg_3139 | gi|126640115|ref|NC_009085.1| | 462302 | 462318 | 17 |
| exprReg_3150 | gi|126640115|ref|NC_009085.1| | 463369 | 463466 | 98 |
| exprReg_3159 | gi|126640115|ref|NC_009085.1| | 464933 | 464954 | 22 |
| exprReg_3160 | gi|126640115|ref|NC_009085.1| | 464968 | 465049 | 82 |
| exprReg_3167 | gi|126640115|ref|NC_009085.1| | 466420 | 466467 | 48 |
| exprReg_3168 | gi|126640115|ref|NC_009085.1| | 466477 | 466538 | 62 |
| exprReg_3176 | gi|126640115|ref|NC_009085.1| | 467676 | 467846 | 171 |
| exprReg_3177 | gi|126640115|ref|NC_009085.1| | 468133 | 468175 | 43 |
| exprReg_3178 | gi|126640115|ref|NC_009085.1| | 468226 | 468244 | 19 |
| exprReg_3179 | gi|126640115|ref|NC_009085.1| | 468258 | 468282 | 25 |
| exprReg_3180 | gi|126640115|ref|NC_009085.1| | 468339 | 468355 | 17 |
| exprReg_3181 | gi|126640115|ref|NC_009085.1| | 468389 | 468415 | 27 |
| exprReg_3206 | gi|126640115|ref|NC_009085.1| | 470749 | 470877 | 129 |
| exprReg_3218 | gi|126640115|ref|NC_009085.1| | 472678 | 472710 | 33 |
| exprReg_3230 | gi|126640115|ref|NC_009085.1| | 474553 | 474593 | 41 |
| exprReg_3231 | gi|126640115|ref|NC_009085.1| | 474606 | 474727 | 122 |
| exprReg_3237 | gi|126640115|ref|NC_009085.1| | 475988 | 476089 | 102 |
| exprReg_3238 | gi|126640115|ref|NC_009085.1| | 476155 | 476195 | 41 |
| exprReg_3244 | gi|126640115|ref|NC_009085.1| | 477196 | 477276 | 81 |
| exprReg_3264 | gi|126640115|ref|NC_009085.1| | 479830 | 479866 | 37 |
| exprReg_3269 | gi|126640115|ref|NC_009085.1| | 480702 | 480759 | 58 |
| exprReg_3270 | gi|126640115|ref|NC_009085.1| | 480764 | 480864 | 101 |
| exprReg_3275 | gi|126640115|ref|NC_009085.1| | 481382 | 481405 | 24 |
| exprReg_3289 | gi|126640115|ref|NC_009085.1| | 483490 | 483507 | 18 |
| exprReg_3291 | gi|126640115|ref|NC_009085.1| | 483839 | 483903 | 65 |
| exprReg_3296 | gi|126640115|ref|NC_009085.1| | 484993 | 485009 | 17 |
| exprReg_3313 | gi|126640115|ref|NC_009085.1| | 486609 | 486653 | 45 |
| exprReg_3314 | gi|126640115|ref|NC_009085.1| | 486658 | 486758 | 101 |
| exprReg_3326 | gi|126640115|ref|NC_009085.1| | 488732 | 488918 | 187 |
| exprReg_3327 | gi|126640115|ref|NC_009085.1| | 488941 | 488958 | 18 |
| exprReg_3333 | gi|126640115|ref|NC_009085.1| | 489769 | 489878 | 110 |
| exprReg_3342 | gi|126640115|ref|NC_009085.1| | 490714 | 490782 | 69 |
| exprReg_3343 | gi|126640115|ref|NC_009085.1| | 490863 | 490886 | 24 |
| exprReg_3344 | gi|126640115|ref|NC_009085.1| | 490955 | 491009 | 55 |
| exprReg_3345 | gi|126640115|ref|NC_009085.1| | 491018 | 491226 | 209 |
| exprReg_3348 | gi|126640115|ref|NC_009085.1| | 491847 | 491874 | 28 |
| exprReg_3358 | gi|126640115|ref|NC_009085.1| | 493386 | 493408 | 23 |
| exprReg_3375 | gi|126640115|ref|NC_009085.1| | 495971 | 496017 | 47 |
| exprReg_3376 | gi|126640115|ref|NC_009085.1| | 496036 | 496073 | 38 |
| exprReg_3377 | gi|126640115|ref|NC_009085.1| | 496077 | 496116 | 40 |
| exprReg_3378 | gi|126640115|ref|NC_009085.1| | 496162 | 496227 | 66 |
| exprReg_3379 | gi|126640115|ref|NC_009085.1| | 496334 | 496351 | 18 |
| exprReg_3380 | gi|126640115|ref|NC_009085.1| | 496363 | 496466 | 104 |
| exprReg_3382 | gi|126640115|ref|NC_009085.1| | 496767 | 496790 | 24 |
| exprReg_3407 | gi|126640115|ref|NC_009085.1| | 500859 | 500879 | 21 |
| exprReg_3408 | gi|126640115|ref|NC_009085.1| | 500899 | 500945 | 47 |
| exprReg_3409 | gi|126640115|ref|NC_009085.1| | 500964 | 500987 | 24 |
| exprReg_3440 | gi|126640115|ref|NC_009085.1| | 506150 | 506259 | 110 |
| exprReg_3446 | gi|126640115|ref|NC_009085.1| | 506866 | 506884 | 19 |
| exprReg_3447 | gi|126640115|ref|NC_009085.1| | 506940 | 506965 | 26 |
| exprReg_3448 | gi|126640115|ref|NC_009085.1| | 506998 | 507034 | 37 |
| exprReg_3455 | gi|126640115|ref|NC_009085.1| | 508204 | 508227 | 24 |
| exprReg_3462 | gi|126640115|ref|NC_009085.1| | 509403 | 509430 | 28 |
| exprReg_3463 | gi|126640115|ref|NC_009085.1| | 509477 | 509495 | 19 |
| exprReg_3477 | gi|126640115|ref|NC_009085.1| | 511249 | 511338 | 90 |
| exprReg_3478 | gi|126640115|ref|NC_009085.1| | 511355 | 511415 | 61 |
| exprReg_3479 | gi|126640115|ref|NC_009085.1| | 511428 | 511595 | 168 |
| exprReg_3505 | gi|126640115|ref|NC_009085.1| | 514720 | 514740 | 21 |
| exprReg_3506 | gi|126640115|ref|NC_009085.1| | 514748 | 514781 | 34 |
| exprReg_3507 | gi|126640115|ref|NC_009085.1| | 514802 | 514828 | 27 |
| exprReg_3508 | gi|126640115|ref|NC_009085.1| | 514857 | 514920 | 64 |
| exprReg_3509 | gi|126640115|ref|NC_009085.1| | 514971 | 515045 | 75 |
| exprReg_3510 | gi|126640115|ref|NC_009085.1| | 515056 | 515079 | 24 |
| exprReg_3511 | gi|126640115|ref|NC_009085.1| | 515098 | 515198 | 101 |
| exprReg_3512 | gi|126640115|ref|NC_009085.1| | 515204 | 515237 | 34 |
| exprReg_3513 | gi|126640115|ref|NC_009085.1| | 515291 | 515407 | 117 |
| exprReg_3520 | gi|126640115|ref|NC_009085.1| | 516659 | 516812 | 154 |
| exprReg_3534 | gi|126640115|ref|NC_009085.1| | 518780 | 518799 | 20 |
| exprReg_3535 | gi|126640115|ref|NC_009085.1| | 518804 | 518863 | 60 |
| exprReg_3536 | gi|126640115|ref|NC_009085.1| | 518906 | 518931 | 26 |
| exprReg_3547 | gi|126640115|ref|NC_009085.1| | 519862 | 519886 | 25 |
| exprReg_3548 | gi|126640115|ref|NC_009085.1| | 520046 | 520082 | 37 |
| exprReg_3549 | gi|126640115|ref|NC_009085.1| | 520144 | 520160 | 17 |
| exprReg_3550 | gi|126640115|ref|NC_009085.1| | 520171 | 520220 | 50 |
| exprReg_3585 | gi|126640115|ref|NC_009085.1| | 525518 | 525615 | 98 |
| exprReg_3586 | gi|126640115|ref|NC_009085.1| | 525872 | 525907 | 36 |
| exprReg_3601 | gi|126640115|ref|NC_009085.1| | 528808 | 528829 | 22 |
| exprReg_3618 | gi|126640115|ref|NC_009085.1| | 531208 | 531581 | 374 |
| exprReg_3619 | gi|126640115|ref|NC_009085.1| | 531599 | 531633 | 35 |
| exprReg_3620 | gi|126640115|ref|NC_009085.1| | 531659 | 531676 | 18 |
| exprReg_3621 | gi|126640115|ref|NC_009085.1| | 531697 | 531746 | 50 |
| exprReg_3622 | gi|126640115|ref|NC_009085.1| | 531799 | 531896 | 98 |
| exprReg_3623 | gi|126640115|ref|NC_009085.1| | 531916 | 532155 | 240 |
| exprReg_3624 | gi|126640115|ref|NC_009085.1| | 532158 | 532220 | 63 |
| exprReg_3625 | gi|126640115|ref|NC_009085.1| | 532238 | 532331 | 94 |
| exprReg_3626 | gi|126640115|ref|NC_009085.1| | 532366 | 532483 | 118 |
| exprReg_3627 | gi|126640115|ref|NC_009085.1| | 532486 | 532560 | 75 |
| exprReg_3628 | gi|126640115|ref|NC_009085.1| | 532642 | 532666 | 25 |
| exprReg_3638 | gi|126640115|ref|NC_009085.1| | 533988 | 534013 | 26 |
| exprReg_3639 | gi|126640115|ref|NC_009085.1| | 534140 | 534217 | 78 |
| exprReg_3640 | gi|126640115|ref|NC_009085.1| | 534241 | 534308 | 68 |
| exprReg_3641 | gi|126640115|ref|NC_009085.1| | 534346 | 534381 | 36 |
| exprReg_3658 | gi|126640115|ref|NC_009085.1| | 536494 | 536527 | 34 |
| exprReg_3659 | gi|126640115|ref|NC_009085.1| | 536548 | 536584 | 37 |
| exprReg_3660 | gi|126640115|ref|NC_009085.1| | 536607 | 536625 | 19 |
| exprReg_3661 | gi|126640115|ref|NC_009085.1| | 536660 | 536747 | 88 |
| exprReg_3667 | gi|126640115|ref|NC_009085.1| | 537765 | 537823 | 59 |
| exprReg_3668 | gi|126640115|ref|NC_009085.1| | 537877 | 537893 | 17 |
| exprReg_3669 | gi|126640115|ref|NC_009085.1| | 537928 | 537989 | 62 |
| exprReg_3670 | gi|126640115|ref|NC_009085.1| | 538003 | 538020 | 18 |
| exprReg_3687 | gi|126640115|ref|NC_009085.1| | 540335 | 540394 | 60 |
| exprReg_3697 | gi|126640115|ref|NC_009085.1| | 542202 | 542218 | 17 |
| exprReg_3698 | gi|126640115|ref|NC_009085.1| | 542253 | 542293 | 41 |
| exprReg_3704 | gi|126640115|ref|NC_009085.1| | 542598 | 542666 | 69 |
| exprReg_3708 | gi|126640115|ref|NC_009085.1| | 543069 | 543205 | 137 |
| exprReg_3709 | gi|126640115|ref|NC_009085.1| | 543209 | 543227 | 19 |
| exprReg_3718 | gi|126640115|ref|NC_009085.1| | 544444 | 544466 | 23 |
| exprReg_3756 | gi|126640115|ref|NC_009085.1| | 550459 | 550477 | 19 |
| exprReg_3757 | gi|126640115|ref|NC_009085.1| | 550481 | 550500 | 20 |
| exprReg_3758 | gi|126640115|ref|NC_009085.1| | 550513 | 550531 | 19 |
| exprReg_3759 | gi|126640115|ref|NC_009085.1| | 550541 | 550586 | 46 |
| exprReg_3770 | gi|126640115|ref|NC_009085.1| | 552046 | 552120 | 75 |
| exprReg_3786 | gi|126640115|ref|NC_009085.1| | 553754 | 553796 | 43 |
| exprReg_3792 | gi|126640115|ref|NC_009085.1| | 554620 | 554638 | 19 |
| exprReg_3793 | gi|126640115|ref|NC_009085.1| | 554662 | 554683 | 22 |
| exprReg_3806 | gi|126640115|ref|NC_009085.1| | 556234 | 556255 | 22 |
| exprReg_3836 | gi|126640115|ref|NC_009085.1| | 560513 | 560550 | 38 |
| exprReg_3837 | gi|126640115|ref|NC_009085.1| | 560560 | 560654 | 95 |
| exprReg_3856 | gi|126640115|ref|NC_009085.1| | 562856 | 562982 | 127 |
| exprReg_3872 | gi|126640115|ref|NC_009085.1| | 566353 | 566428 | 76 |
| exprReg_3873 | gi|126640115|ref|NC_009085.1| | 566436 | 566452 | 17 |
| exprReg_3891 | gi|126640115|ref|NC_009085.1| | 568719 | 568793 | 75 |
| exprReg_3892 | gi|126640115|ref|NC_009085.1| | 568801 | 568817 | 17 |
| exprReg_3907 | gi|126640115|ref|NC_009085.1| | 571661 | 571728 | 68 |
| exprReg_3908 | gi|126640115|ref|NC_009085.1| | 571772 | 571848 | 77 |
| exprReg_3920 | gi|126640115|ref|NC_009085.1| | 573375 | 573391 | 17 |
| exprReg_3927 | gi|126640115|ref|NC_009085.1| | 574325 | 574354 | 30 |
| exprReg_3962 | gi|126640115|ref|NC_009085.1| | 579023 | 579047 | 25 |
| exprReg_3963 | gi|126640115|ref|NC_009085.1| | 579050 | 579067 | 18 |
| exprReg_3984 | gi|126640115|ref|NC_009085.1| | 581565 | 581667 | 103 |
| exprReg_3985 | gi|126640115|ref|NC_009085.1| | 581718 | 581766 | 49 |
| exprReg_3993 | gi|126640115|ref|NC_009085.1| | 583412 | 583431 | 20 |
| exprReg_3994 | gi|126640115|ref|NC_009085.1| | 583447 | 583463 | 17 |
| exprReg_3995 | gi|126640115|ref|NC_009085.1| | 583512 | 583531 | 20 |
| exprReg_3996 | gi|126640115|ref|NC_009085.1| | 583557 | 583686 | 130 |
| exprReg_3997 | gi|126640115|ref|NC_009085.1| | 583801 | 583836 | 36 |
| exprReg_3998 | gi|126640115|ref|NC_009085.1| | 583852 | 583904 | 53 |
| exprReg_3999 | gi|126640115|ref|NC_009085.1| | 583938 | 583960 | 23 |
| exprReg_4000 | gi|126640115|ref|NC_009085.1| | 584109 | 584310 | 202 |
| exprReg_4001 | gi|126640115|ref|NC_009085.1| | 584324 | 584350 | 27 |
| exprReg_4021 | gi|126640115|ref|NC_009085.1| | 586747 | 586784 | 38 |
| exprReg_4022 | gi|126640115|ref|NC_009085.1| | 586880 | 586899 | 20 |
| exprReg_4045 | gi|126640115|ref|NC_009085.1| | 590214 | 590424 | 211 |
| exprReg_4046 | gi|126640115|ref|NC_009085.1| | 590458 | 590477 | 20 |
| exprReg_4047 | gi|126640115|ref|NC_009085.1| | 590562 | 590606 | 45 |
| exprReg_4048 | gi|126640115|ref|NC_009085.1| | 590675 | 590796 | 122 |
| exprReg_4051 | gi|126640115|ref|NC_009085.1| | 591376 | 591414 | 39 |
| exprReg_4061 | gi|126640115|ref|NC_009085.1| | 592264 | 592294 | 31 |
| exprReg_4062 | gi|126640115|ref|NC_009085.1| | 592314 | 592348 | 35 |
| exprReg_4072 | gi|126640115|ref|NC_009085.1| | 593270 | 593286 | 17 |
| exprReg_4073 | gi|126640115|ref|NC_009085.1| | 593350 | 593547 | 198 |
| exprReg_4074 | gi|126640115|ref|NC_009085.1| | 593566 | 593718 | 153 |
| exprReg_4110 | gi|126640115|ref|NC_009085.1| | 598962 | 598992 | 31 |
| exprReg_4111 | gi|126640115|ref|NC_009085.1| | 599092 | 599116 | 25 |
| exprReg_4112 | gi|126640115|ref|NC_009085.1| | 599185 | 599262 | 78 |
| exprReg_4119 | gi|126640115|ref|NC_009085.1| | 600147 | 600349 | 203 |
| exprReg_4129 | gi|126640115|ref|NC_009085.1| | 602018 | 602080 | 63 |
| exprReg_4136 | gi|126640115|ref|NC_009085.1| | 602850 | 602937 | 88 |
| exprReg_4137 | gi|126640115|ref|NC_009085.1| | 602956 | 602974 | 19 |
| exprReg_4149 | gi|126640115|ref|NC_009085.1| | 604671 | 604689 | 19 |
| exprReg_4150 | gi|126640115|ref|NC_009085.1| | 604698 | 604839 | 142 |
| exprReg_4151 | gi|126640115|ref|NC_009085.1| | 604854 | 604883 | 30 |
| exprReg_4152 | gi|126640115|ref|NC_009085.1| | 604912 | 604928 | 17 |
| exprReg_4170 | gi|126640115|ref|NC_009085.1| | 607813 | 607886 | 74 |
| exprReg_4176 | gi|126640115|ref|NC_009085.1| | 608864 | 608975 | 112 |
| exprReg_4188 | gi|126640115|ref|NC_009085.1| | 610419 | 610465 | 47 |
| exprReg_4189 | gi|126640115|ref|NC_009085.1| | 610541 | 610562 | 22 |
| exprReg_4197 | gi|126640115|ref|NC_009085.1| | 611683 | 611712 | 30 |
| exprReg_4204 | gi|126640115|ref|NC_009085.1| | 612778 | 612795 | 18 |
| exprReg_4205 | gi|126640115|ref|NC_009085.1| | 612799 | 612826 | 28 |
| exprReg_4206 | gi|126640115|ref|NC_009085.1| | 612835 | 612968 | 134 |
| exprReg_4217 | gi|126640115|ref|NC_009085.1| | 614440 | 614462 | 23 |
| exprReg_4218 | gi|126640115|ref|NC_009085.1| | 614466 | 614486 | 21 |
| exprReg_4219 | gi|126640115|ref|NC_009085.1| | 614614 | 614663 | 50 |
| exprReg_4220 | gi|126640115|ref|NC_009085.1| | 614690 | 614822 | 133 |
| exprReg_4289 | gi|126640115|ref|NC_009085.1| | 624679 | 624840 | 162 |
| exprReg_4296 | gi|126640115|ref|NC_009085.1| | 625739 | 625759 | 21 |
| exprReg_4297 | gi|126640115|ref|NC_009085.1| | 625764 | 625781 | 18 |
| exprReg_4303 | gi|126640115|ref|NC_009085.1| | 626484 | 626834 | 351 |
| exprReg_4304 | gi|126640115|ref|NC_009085.1| | 626841 | 626861 | 21 |
| exprReg_4305 | gi|126640115|ref|NC_009085.1| | 626911 | 627083 | 173 |
| exprReg_4306 | gi|126640115|ref|NC_009085.1| | 627096 | 627146 | 51 |
| exprReg_4307 | gi|126640115|ref|NC_009085.1| | 627172 | 627199 | 28 |
| exprReg_4308 | gi|126640115|ref|NC_009085.1| | 627204 | 627280 | 77 |
| exprReg_4309 | gi|126640115|ref|NC_009085.1| | 627345 | 627452 | 108 |
| exprReg_4310 | gi|126640115|ref|NC_009085.1| | 627455 | 627475 | 21 |
| exprReg_4311 | gi|126640115|ref|NC_009085.1| | 627495 | 627664 | 170 |
| exprReg_4312 | gi|126640115|ref|NC_009085.1| | 627681 | 627697 | 17 |
| exprReg_4313 | gi|126640115|ref|NC_009085.1| | 627704 | 627751 | 48 |
| exprReg_4314 | gi|126640115|ref|NC_009085.1| | 627754 | 627808 | 55 |
| exprReg_4326 | gi|126640115|ref|NC_009085.1| | 630916 | 630969 | 54 |
| exprReg_4327 | gi|126640115|ref|NC_009085.1| | 631012 | 631065 | 54 |
| exprReg_4334 | gi|126640115|ref|NC_009085.1| | 632398 | 632455 | 58 |
| exprReg_4335 | gi|126640115|ref|NC_009085.1| | 632460 | 632568 | 109 |
| exprReg_4353 | gi|126640115|ref|NC_009085.1| | 634469 | 634489 | 21 |
| exprReg_4362 | gi|126640115|ref|NC_009085.1| | 635290 | 635330 | 41 |
| exprReg_4390 | gi|126640115|ref|NC_009085.1| | 639350 | 639464 | 115 |
| exprReg_4391 | gi|126640115|ref|NC_009085.1| | 639569 | 639585 | 17 |
| exprReg_4392 | gi|126640115|ref|NC_009085.1| | 639745 | 639762 | 18 |
| exprReg_4393 | gi|126640115|ref|NC_009085.1| | 639773 | 639861 | 89 |
| exprReg_4394 | gi|126640115|ref|NC_009085.1| | 639911 | 639954 | 44 |
| exprReg_4404 | gi|126640115|ref|NC_009085.1| | 641806 | 642004 | 199 |
| exprReg_4410 | gi|126640115|ref|NC_009085.1| | 642375 | 642393 | 19 |
| exprReg_4411 | gi|126640115|ref|NC_009085.1| | 642499 | 642532 | 34 |
| exprReg_4412 | gi|126640115|ref|NC_009085.1| | 642545 | 642594 | 50 |
| exprReg_4416 | gi|126640115|ref|NC_009085.1| | 643290 | 643310 | 21 |
| exprReg_4426 | gi|126640115|ref|NC_009085.1| | 644840 | 644900 | 61 |
| exprReg_4427 | gi|126640115|ref|NC_009085.1| | 644920 | 644976 | 57 |
| exprReg_4428 | gi|126640115|ref|NC_009085.1| | 645004 | 645031 | 28 |
| exprReg_4429 | gi|126640115|ref|NC_009085.1| | 645111 | 645263 | 153 |
| exprReg_4430 | gi|126640115|ref|NC_009085.1| | 645266 | 645282 | 17 |
| exprReg_4431 | gi|126640115|ref|NC_009085.1| | 645302 | 645346 | 45 |
| exprReg_4432 | gi|126640115|ref|NC_009085.1| | 645348 | 645364 | 17 |
| exprReg_4438 | gi|126640115|ref|NC_009085.1| | 645859 | 645973 | 115 |
| exprReg_4439 | gi|126640115|ref|NC_009085.1| | 645976 | 646015 | 40 |
| exprReg_4450 | gi|126640115|ref|NC_009085.1| | 647555 | 647571 | 17 |
| exprReg_4451 | gi|126640115|ref|NC_009085.1| | 647578 | 647668 | 91 |
| exprReg_4452 | gi|126640115|ref|NC_009085.1| | 647707 | 647742 | 36 |
| exprReg_4453 | gi|126640115|ref|NC_009085.1| | 647754 | 647784 | 31 |
| exprReg_4454 | gi|126640115|ref|NC_009085.1| | 647788 | 647806 | 19 |
| exprReg_4455 | gi|126640115|ref|NC_009085.1| | 647815 | 647832 | 18 |
| exprReg_4456 | gi|126640115|ref|NC_009085.1| | 647921 | 647973 | 53 |
| exprReg_4457 | gi|126640115|ref|NC_009085.1| | 648105 | 648187 | 83 |
| exprReg_4458 | gi|126640115|ref|NC_009085.1| | 648207 | 648224 | 18 |
| exprReg_4459 | gi|126640115|ref|NC_009085.1| | 648228 | 648244 | 17 |
| exprReg_4486 | gi|126640115|ref|NC_009085.1| | 651955 | 652382 | 428 |
| exprReg_4487 | gi|126640115|ref|NC_009085.1| | 652418 | 652439 | 22 |
| exprReg_4488 | gi|126640115|ref|NC_009085.1| | 652678 | 652745 | 68 |
| exprReg_4489 | gi|126640115|ref|NC_009085.1| | 652750 | 652930 | 181 |
| exprReg_4490 | gi|126640115|ref|NC_009085.1| | 652942 | 653134 | 193 |
| exprReg_4491 | gi|126640115|ref|NC_009085.1| | 653154 | 653279 | 126 |
| exprReg_4492 | gi|126640115|ref|NC_009085.1| | 653366 | 653383 | 18 |
| exprReg_4493 | gi|126640115|ref|NC_009085.1| | 653394 | 653451 | 58 |
| exprReg_4494 | gi|126640115|ref|NC_009085.1| | 653456 | 653472 | 17 |
| exprReg_4495 | gi|126640115|ref|NC_009085.1| | 653507 | 653533 | 27 |
| exprReg_4496 | gi|126640115|ref|NC_009085.1| | 653559 | 653576 | 18 |
| exprReg_4497 | gi|126640115|ref|NC_009085.1| | 653618 | 653880 | 263 |
| exprReg_4498 | gi|126640115|ref|NC_009085.1| | 653952 | 654010 | 59 |
| exprReg_4505 | gi|126640115|ref|NC_009085.1| | 654771 | 654826 | 56 |
| exprReg_4506 | gi|126640115|ref|NC_009085.1| | 654829 | 654890 | 62 |
| exprReg_4519 | gi|126640115|ref|NC_009085.1| | 656403 | 656531 | 129 |
| exprReg_4569 | gi|126640115|ref|NC_009085.1| | 662750 | 662769 | 20 |
| exprReg_4570 | gi|126640115|ref|NC_009085.1| | 662776 | 662795 | 20 |
| exprReg_4571 | gi|126640115|ref|NC_009085.1| | 662887 | 663094 | 208 |
| exprReg_4572 | gi|126640115|ref|NC_009085.1| | 663109 | 663150 | 42 |
| exprReg_4599 | gi|126640115|ref|NC_009085.1| | 666444 | 666527 | 84 |
| exprReg_4600 | gi|126640115|ref|NC_009085.1| | 666536 | 666561 | 26 |
| exprReg_4601 | gi|126640115|ref|NC_009085.1| | 666615 | 666633 | 19 |
| exprReg_4605 | gi|126640115|ref|NC_009085.1| | 667442 | 667473 | 32 |
| exprReg_4606 | gi|126640115|ref|NC_009085.1| | 667503 | 667568 | 66 |
| exprReg_4607 | gi|126640115|ref|NC_009085.1| | 667573 | 667597 | 25 |
| exprReg_4608 | gi|126640115|ref|NC_009085.1| | 667629 | 667709 | 81 |
| exprReg_4609 | gi|126640115|ref|NC_009085.1| | 667788 | 667813 | 26 |
| exprReg_4627 | gi|126640115|ref|NC_009085.1| | 670048 | 670506 | 459 |
| exprReg_4628 | gi|126640115|ref|NC_009085.1| | 670526 | 670628 | 103 |
| exprReg_4636 | gi|126640115|ref|NC_009085.1| | 672056 | 672091 | 36 |
| exprReg_4644 | gi|126640115|ref|NC_009085.1| | 673012 | 673029 | 18 |
| exprReg_4645 | gi|126640115|ref|NC_009085.1| | 673044 | 673077 | 34 |
| exprReg_4651 | gi|126640115|ref|NC_009085.1| | 673948 | 674146 | 199 |
| exprReg_4652 | gi|126640115|ref|NC_009085.1| | 674159 | 674210 | 52 |
| exprReg_4653 | gi|126640115|ref|NC_009085.1| | 674344 | 674444 | 101 |
| exprReg_4654 | gi|126640115|ref|NC_009085.1| | 674461 | 674597 | 137 |
| exprReg_4655 | gi|126640115|ref|NC_009085.1| | 674602 | 674807 | 206 |
| exprReg_4672 | gi|126640115|ref|NC_009085.1| | 676849 | 676870 | 22 |
| exprReg_4673 | gi|126640115|ref|NC_009085.1| | 676875 | 676891 | 17 |
| exprReg_4674 | gi|126640115|ref|NC_009085.1| | 676989 | 677005 | 17 |
| exprReg_4681 | gi|126640115|ref|NC_009085.1| | 677882 | 677936 | 55 |
| exprReg_4682 | gi|126640115|ref|NC_009085.1| | 678049 | 678105 | 57 |
| exprReg_4683 | gi|126640115|ref|NC_009085.1| | 678257 | 678330 | 74 |
| exprReg_4684 | gi|126640115|ref|NC_009085.1| | 678401 | 678420 | 20 |
| exprReg_4685 | gi|126640115|ref|NC_009085.1| | 678448 | 678465 | 18 |
| exprReg_4690 | gi|126640115|ref|NC_009085.1| | 679059 | 679075 | 17 |
| exprReg_4691 | gi|126640115|ref|NC_009085.1| | 679107 | 679189 | 83 |
| exprReg_4692 | gi|126640115|ref|NC_009085.1| | 679234 | 679251 | 18 |
| exprReg_4693 | gi|126640115|ref|NC_009085.1| | 679279 | 679297 | 19 |
| exprReg_4694 | gi|126640115|ref|NC_009085.1| | 679310 | 679328 | 19 |
| exprReg_4695 | gi|126640115|ref|NC_009085.1| | 679346 | 679365 | 20 |
| exprReg_4696 | gi|126640115|ref|NC_009085.1| | 679483 | 679516 | 34 |
| exprReg_4697 | gi|126640115|ref|NC_009085.1| | 679621 | 679646 | 26 |
| exprReg_4703 | gi|126640115|ref|NC_009085.1| | 680399 | 680430 | 32 |
| exprReg_4704 | gi|126640115|ref|NC_009085.1| | 680529 | 680556 | 28 |
| exprReg_4705 | gi|126640115|ref|NC_009085.1| | 680574 | 680607 | 34 |
| exprReg_4706 | gi|126640115|ref|NC_009085.1| | 680720 | 680740 | 21 |
| exprReg_4707 | gi|126640115|ref|NC_009085.1| | 680759 | 680810 | 52 |
| exprReg_4708 | gi|126640115|ref|NC_009085.1| | 680891 | 680911 | 21 |
| exprReg_4709 | gi|126640115|ref|NC_009085.1| | 681110 | 681126 | 17 |
| exprReg_4710 | gi|126640115|ref|NC_009085.1| | 681151 | 681220 | 70 |
| exprReg_4711 | gi|126640115|ref|NC_009085.1| | 681285 | 681306 | 22 |
| exprReg_4712 | gi|126640115|ref|NC_009085.1| | 681467 | 681489 | 23 |
| exprReg_4713 | gi|126640115|ref|NC_009085.1| | 681491 | 681508 | 18 |
| exprReg_4714 | gi|126640115|ref|NC_009085.1| | 681557 | 681659 | 103 |
| exprReg_4715 | gi|126640115|ref|NC_009085.1| | 681767 | 681784 | 18 |
| exprReg_4716 | gi|126640115|ref|NC_009085.1| | 681811 | 681827 | 17 |
| exprReg_4717 | gi|126640115|ref|NC_009085.1| | 681831 | 681852 | 22 |
| exprReg_4718 | gi|126640115|ref|NC_009085.1| | 682036 | 682055 | 20 |
| exprReg_4719 | gi|126640115|ref|NC_009085.1| | 682111 | 682135 | 25 |
| exprReg_4720 | gi|126640115|ref|NC_009085.1| | 682169 | 682185 | 17 |
| exprReg_4721 | gi|126640115|ref|NC_009085.1| | 682229 | 682247 | 19 |
| exprReg_4722 | gi|126640115|ref|NC_009085.1| | 682328 | 682391 | 64 |
| exprReg_4723 | gi|126640115|ref|NC_009085.1| | 682396 | 682470 | 75 |
| exprReg_4724 | gi|126640115|ref|NC_009085.1| | 682472 | 682771 | 300 |
| exprReg_4725 | gi|126640115|ref|NC_009085.1| | 682787 | 682970 | 184 |
| exprReg_4726 | gi|126640115|ref|NC_009085.1| | 683017 | 683189 | 173 |
| exprReg_4727 | gi|126640115|ref|NC_009085.1| | 683191 | 683255 | 65 |
| exprReg_4728 | gi|126640115|ref|NC_009085.1| | 683294 | 683315 | 22 |
| exprReg_4729 | gi|126640115|ref|NC_009085.1| | 683342 | 683359 | 18 |
| exprReg_4730 | gi|126640115|ref|NC_009085.1| | 683364 | 683454 | 91 |
| exprReg_4738 | gi|126640115|ref|NC_009085.1| | 684804 | 684825 | 22 |
| exprReg_4739 | gi|126640115|ref|NC_009085.1| | 684884 | 684900 | 17 |
| exprReg_4740 | gi|126640115|ref|NC_009085.1| | 685004 | 685035 | 32 |
| exprReg_4741 | gi|126640115|ref|NC_009085.1| | 685074 | 685090 | 17 |
| exprReg_4742 | gi|126640115|ref|NC_009085.1| | 685135 | 685374 | 240 |
| exprReg_4743 | gi|126640115|ref|NC_009085.1| | 685421 | 685437 | 17 |
| exprReg_4744 | gi|126640115|ref|NC_009085.1| | 685476 | 685511 | 36 |
| exprReg_4745 | gi|126640115|ref|NC_009085.1| | 685573 | 685591 | 19 |
| exprReg_4746 | gi|126640115|ref|NC_009085.1| | 685674 | 685694 | 21 |
| exprReg_4747 | gi|126640115|ref|NC_009085.1| | 685766 | 685785 | 20 |
| exprReg_4748 | gi|126640115|ref|NC_009085.1| | 685802 | 685894 | 93 |
| exprReg_4749 | gi|126640115|ref|NC_009085.1| | 685972 | 686013 | 42 |
| exprReg_4750 | gi|126640115|ref|NC_009085.1| | 686048 | 686151 | 104 |
| exprReg_4751 | gi|126640115|ref|NC_009085.1| | 686154 | 686338 | 185 |
| exprReg_4758 | gi|126640115|ref|NC_009085.1| | 687412 | 687441 | 30 |
| exprReg_4759 | gi|126640115|ref|NC_009085.1| | 687592 | 687644 | 53 |
| exprReg_4760 | gi|126640115|ref|NC_009085.1| | 687831 | 687959 | 129 |
| exprReg_4761 | gi|126640115|ref|NC_009085.1| | 688084 | 688158 | 75 |
| exprReg_4762 | gi|126640115|ref|NC_009085.1| | 688252 | 688358 | 107 |
| exprReg_4763 | gi|126640115|ref|NC_009085.1| | 688609 | 688835 | 227 |
| exprReg_4764 | gi|126640115|ref|NC_009085.1| | 688902 | 689108 | 207 |
| exprReg_4765 | gi|126640115|ref|NC_009085.1| | 689123 | 689139 | 17 |
| exprReg_4766 | gi|126640115|ref|NC_009085.1| | 689155 | 689230 | 76 |
| exprReg_4767 | gi|126640115|ref|NC_009085.1| | 689235 | 689261 | 27 |
| exprReg_4768 | gi|126640115|ref|NC_009085.1| | 689362 | 689383 | 22 |
| exprReg_4769 | gi|126640115|ref|NC_009085.1| | 689437 | 689453 | 17 |
| exprReg_4770 | gi|126640115|ref|NC_009085.1| | 689518 | 689573 | 56 |
| exprReg_4771 | gi|126640115|ref|NC_009085.1| | 689599 | 689654 | 56 |
| exprReg_4772 | gi|126640115|ref|NC_009085.1| | 689663 | 689768 | 106 |
| exprReg_4773 | gi|126640115|ref|NC_009085.1| | 689817 | 690030 | 214 |
| exprReg_4774 | gi|126640115|ref|NC_009085.1| | 690046 | 690136 | 91 |
| exprReg_4775 | gi|126640115|ref|NC_009085.1| | 690162 | 690248 | 87 |
| exprReg_4776 | gi|126640115|ref|NC_009085.1| | 690255 | 690310 | 56 |
| exprReg_4777 | gi|126640115|ref|NC_009085.1| | 690346 | 690386 | 41 |
| exprReg_4778 | gi|126640115|ref|NC_009085.1| | 690478 | 690811 | 334 |
| exprReg_4779 | gi|126640115|ref|NC_009085.1| | 690839 | 690881 | 43 |
| exprReg_4780 | gi|126640115|ref|NC_009085.1| | 690971 | 690999 | 29 |
| exprReg_4781 | gi|126640115|ref|NC_009085.1| | 691460 | 691501 | 42 |
| exprReg_4782 | gi|126640115|ref|NC_009085.1| | 691506 | 691523 | 18 |
| exprReg_4783 | gi|126640115|ref|NC_009085.1| | 691596 | 691616 | 21 |
| exprReg_4784 | gi|126640115|ref|NC_009085.1| | 691625 | 691643 | 19 |
| exprReg_4785 | gi|126640115|ref|NC_009085.1| | 691654 | 691694 | 41 |
| exprReg_4786 | gi|126640115|ref|NC_009085.1| | 691771 | 691793 | 23 |
| exprReg_4787 | gi|126640115|ref|NC_009085.1| | 691801 | 691845 | 45 |
| exprReg_4788 | gi|126640115|ref|NC_009085.1| | 691859 | 691924 | 66 |
| exprReg_4789 | gi|126640115|ref|NC_009085.1| | 691933 | 691953 | 21 |
| exprReg_4790 | gi|126640115|ref|NC_009085.1| | 691964 | 691982 | 19 |
| exprReg_4791 | gi|126640115|ref|NC_009085.1| | 692050 | 692205 | 156 |
| exprReg_4792 | gi|126640115|ref|NC_009085.1| | 692215 | 692240 | 26 |
| exprReg_4793 | gi|126640115|ref|NC_009085.1| | 692259 | 692338 | 80 |
| exprReg_4794 | gi|126640115|ref|NC_009085.1| | 692524 | 692560 | 37 |
| exprReg_4795 | gi|126640115|ref|NC_009085.1| | 692624 | 692651 | 28 |
| exprReg_4796 | gi|126640115|ref|NC_009085.1| | 692798 | 692815 | 18 |
| exprReg_4831 | gi|126640115|ref|NC_009085.1| | 697494 | 697511 | 18 |
| exprReg_4832 | gi|126640115|ref|NC_009085.1| | 697956 | 698203 | 248 |
| exprReg_4833 | gi|126640115|ref|NC_009085.1| | 698213 | 698232 | 20 |
| exprReg_4834 | gi|126640115|ref|NC_009085.1| | 698237 | 698281 | 45 |
| exprReg_4835 | gi|126640115|ref|NC_009085.1| | 698356 | 698375 | 20 |
| exprReg_4836 | gi|126640115|ref|NC_009085.1| | 698437 | 698455 | 19 |
| exprReg_4837 | gi|126640115|ref|NC_009085.1| | 698553 | 698572 | 20 |
| exprReg_4838 | gi|126640115|ref|NC_009085.1| | 698639 | 698655 | 17 |
| exprReg_4839 | gi|126640115|ref|NC_009085.1| | 698839 | 698923 | 85 |
| exprReg_4840 | gi|126640115|ref|NC_009085.1| | 699039 | 699305 | 267 |
| exprReg_4841 | gi|126640115|ref|NC_009085.1| | 699312 | 699331 | 20 |
| exprReg_4842 | gi|126640115|ref|NC_009085.1| | 699351 | 699394 | 44 |
| exprReg_4843 | gi|126640115|ref|NC_009085.1| | 699703 | 699743 | 41 |
| exprReg_4844 | gi|126640115|ref|NC_009085.1| | 699910 | 699931 | 22 |
| exprReg_4845 | gi|126640115|ref|NC_009085.1| | 699951 | 700030 | 80 |
| exprReg_4846 | gi|126640115|ref|NC_009085.1| | 700057 | 700126 | 70 |
| exprReg_4847 | gi|126640115|ref|NC_009085.1| | 700178 | 700376 | 199 |
| exprReg_4848 | gi|126640115|ref|NC_009085.1| | 700468 | 700552 | 85 |
| exprReg_4849 | gi|126640115|ref|NC_009085.1| | 700763 | 700811 | 49 |
| exprReg_4850 | gi|126640115|ref|NC_009085.1| | 700813 | 701025 | 213 |
| exprReg_4851 | gi|126640115|ref|NC_009085.1| | 701144 | 701184 | 41 |
| exprReg_4852 | gi|126640115|ref|NC_009085.1| | 701217 | 701234 | 18 |
| exprReg_4853 | gi|126640115|ref|NC_009085.1| | 701274 | 701309 | 36 |
| exprReg_4854 | gi|126640115|ref|NC_009085.1| | 701339 | 701356 | 18 |
| exprReg_4855 | gi|126640115|ref|NC_009085.1| | 701393 | 701575 | 183 |
| exprReg_4856 | gi|126640115|ref|NC_009085.1| | 701767 | 701786 | 20 |
| exprReg_4857 | gi|126640115|ref|NC_009085.1| | 701789 | 701806 | 18 |
| exprReg_4858 | gi|126640115|ref|NC_009085.1| | 701876 | 701915 | 40 |
| exprReg_4859 | gi|126640115|ref|NC_009085.1| | 701942 | 701969 | 28 |
| exprReg_4860 | gi|126640115|ref|NC_009085.1| | 701993 | 702015 | 23 |
| exprReg_4861 | gi|126640115|ref|NC_009085.1| | 702039 | 702134 | 96 |
| exprReg_4862 | gi|126640115|ref|NC_009085.1| | 702136 | 702182 | 47 |
| exprReg_4863 | gi|126640115|ref|NC_009085.1| | 702249 | 702276 | 28 |
| exprReg_4864 | gi|126640115|ref|NC_009085.1| | 702352 | 702407 | 56 |
| exprReg_4865 | gi|126640115|ref|NC_009085.1| | 702441 | 702476 | 36 |
| exprReg_4866 | gi|126640115|ref|NC_009085.1| | 702504 | 702548 | 45 |
| exprReg_4867 | gi|126640115|ref|NC_009085.1| | 702551 | 702610 | 60 |
| exprReg_4868 | gi|126640115|ref|NC_009085.1| | 702618 | 702636 | 19 |
| exprReg_4869 | gi|126640115|ref|NC_009085.1| | 702674 | 702728 | 55 |
| exprReg_4870 | gi|126640115|ref|NC_009085.1| | 702777 | 702896 | 120 |
| exprReg_4871 | gi|126640115|ref|NC_009085.1| | 702932 | 702960 | 29 |
| exprReg_4872 | gi|126640115|ref|NC_009085.1| | 702974 | 702992 | 19 |
| exprReg_4873 | gi|126640115|ref|NC_009085.1| | 703007 | 703024 | 18 |
| exprReg_4874 | gi|126640115|ref|NC_009085.1| | 703067 | 703085 | 19 |
| exprReg_4875 | gi|126640115|ref|NC_009085.1| | 703096 | 703114 | 19 |
| exprReg_4876 | gi|126640115|ref|NC_009085.1| | 703137 | 703155 | 19 |
| exprReg_4877 | gi|126640115|ref|NC_009085.1| | 703158 | 703331 | 174 |
| exprReg_4878 | gi|126640115|ref|NC_009085.1| | 703337 | 703485 | 149 |
| exprReg_4879 | gi|126640115|ref|NC_009085.1| | 703507 | 703565 | 59 |
| exprReg_4880 | gi|126640115|ref|NC_009085.1| | 703568 | 703849 | 282 |
| exprReg_4898 | gi|126640115|ref|NC_009085.1| | 706258 | 706274 | 17 |
| exprReg_4899 | gi|126640115|ref|NC_009085.1| | 706370 | 706386 | 17 |
| exprReg_4900 | gi|126640115|ref|NC_009085.1| | 706434 | 706453 | 20 |
| exprReg_4925 | gi|126640115|ref|NC_009085.1| | 710038 | 710091 | 54 |
| exprReg_4926 | gi|126640115|ref|NC_009085.1| | 710395 | 710524 | 130 |
| exprReg_4927 | gi|126640115|ref|NC_009085.1| | 710549 | 710565 | 17 |
| exprReg_4928 | gi|126640115|ref|NC_009085.1| | 710572 | 710610 | 39 |
| exprReg_4929 | gi|126640115|ref|NC_009085.1| | 710846 | 710866 | 21 |
| exprReg_4930 | gi|126640115|ref|NC_009085.1| | 710902 | 710938 | 37 |
| exprReg_4931 | gi|126640115|ref|NC_009085.1| | 711044 | 711061 | 18 |
| exprReg_4932 | gi|126640115|ref|NC_009085.1| | 711149 | 711246 | 98 |
| exprReg_4933 | gi|126640115|ref|NC_009085.1| | 711268 | 711301 | 34 |
| exprReg_4934 | gi|126640115|ref|NC_009085.1| | 711526 | 711556 | 31 |
| exprReg_4935 | gi|126640115|ref|NC_009085.1| | 711689 | 711784 | 96 |
| exprReg_4936 | gi|126640115|ref|NC_009085.1| | 711842 | 711859 | 18 |
| exprReg_4937 | gi|126640115|ref|NC_009085.1| | 711997 | 712082 | 86 |
| exprReg_4938 | gi|126640115|ref|NC_009085.1| | 712123 | 712530 | 408 |
| exprReg_4939 | gi|126640115|ref|NC_009085.1| | 712537 | 712556 | 20 |
| exprReg_4940 | gi|126640115|ref|NC_009085.1| | 712658 | 713049 | 392 |
| exprReg_4941 | gi|126640115|ref|NC_009085.1| | 713054 | 713098 | 45 |
| exprReg_4942 | gi|126640115|ref|NC_009085.1| | 713156 | 713267 | 112 |
| exprReg_4943 | gi|126640115|ref|NC_009085.1| | 713318 | 713346 | 29 |
| exprReg_4944 | gi|126640115|ref|NC_009085.1| | 713355 | 713456 | 102 |
| exprReg_4945 | gi|126640115|ref|NC_009085.1| | 713517 | 713728 | 212 |
| exprReg_4946 | gi|126640115|ref|NC_009085.1| | 713784 | 713875 | 92 |
| exprReg_4947 | gi|126640115|ref|NC_009085.1| | 713880 | 713947 | 68 |
| exprReg_4948 | gi|126640115|ref|NC_009085.1| | 713990 | 714006 | 17 |
| exprReg_4949 | gi|126640115|ref|NC_009085.1| | 714017 | 714311 | 295 |
| exprReg_4950 | gi|126640115|ref|NC_009085.1| | 714322 | 714384 | 63 |
| exprReg_4951 | gi|126640115|ref|NC_009085.1| | 714393 | 714491 | 99 |
| exprReg_4952 | gi|126640115|ref|NC_009085.1| | 714517 | 714539 | 23 |
| exprReg_4953 | gi|126640115|ref|NC_009085.1| | 714551 | 714600 | 50 |
| exprReg_4954 | gi|126640115|ref|NC_009085.1| | 714618 | 714967 | 350 |
| exprReg_4955 | gi|126640115|ref|NC_009085.1| | 714980 | 714998 | 19 |
| exprReg_4987 | gi|126640115|ref|NC_009085.1| | 718367 | 718383 | 17 |
| exprReg_4999 | gi|126640115|ref|NC_009085.1| | 720353 | 720369 | 17 |
| exprReg_5000 | gi|126640115|ref|NC_009085.1| | 720623 | 720657 | 35 |
| exprReg_5001 | gi|126640115|ref|NC_009085.1| | 720695 | 720739 | 45 |
| exprReg_5002 | gi|126640115|ref|NC_009085.1| | 720749 | 720765 | 17 |
| exprReg_5003 | gi|126640115|ref|NC_009085.1| | 720767 | 720993 | 227 |
| exprReg_5004 | gi|126640115|ref|NC_009085.1| | 721007 | 721040 | 34 |
| exprReg_5005 | gi|126640115|ref|NC_009085.1| | 721103 | 721353 | 251 |
| exprReg_5006 | gi|126640115|ref|NC_009085.1| | 721394 | 721510 | 117 |
| exprReg_5007 | gi|126640115|ref|NC_009085.1| | 721572 | 721672 | 101 |
| exprReg_5018 | gi|126640115|ref|NC_009085.1| | 723143 | 723170 | 28 |
| exprReg_5019 | gi|126640115|ref|NC_009085.1| | 723219 | 723261 | 43 |
| exprReg_5036 | gi|126640115|ref|NC_009085.1| | 725168 | 725281 | 114 |
| exprReg_5037 | gi|126640115|ref|NC_009085.1| | 725294 | 725312 | 19 |
| exprReg_5038 | gi|126640115|ref|NC_009085.1| | 725350 | 725372 | 23 |
| exprReg_5039 | gi|126640115|ref|NC_009085.1| | 725413 | 725430 | 18 |
| exprReg_5047 | gi|126640115|ref|NC_009085.1| | 726403 | 726685 | 283 |
| exprReg_5048 | gi|126640115|ref|NC_009085.1| | 726762 | 727096 | 335 |
| exprReg_5059 | gi|126640115|ref|NC_009085.1| | 728051 | 728270 | 220 |
| exprReg_5060 | gi|126640115|ref|NC_009085.1| | 728329 | 728428 | 100 |
| exprReg_5061 | gi|126640115|ref|NC_009085.1| | 728581 | 728717 | 137 |
| exprReg_5062 | gi|126640115|ref|NC_009085.1| | 728762 | 728778 | 17 |
| exprReg_5063 | gi|126640115|ref|NC_009085.1| | 728789 | 728810 | 22 |
| exprReg_5064 | gi|126640115|ref|NC_009085.1| | 728855 | 728872 | 18 |
| exprReg_5065 | gi|126640115|ref|NC_009085.1| | 728997 | 729051 | 55 |
| exprReg_5066 | gi|126640115|ref|NC_009085.1| | 729085 | 729138 | 54 |
| exprReg_5067 | gi|126640115|ref|NC_009085.1| | 729190 | 729210 | 21 |
| exprReg_5068 | gi|126640115|ref|NC_009085.1| | 729273 | 729292 | 20 |
| exprReg_5069 | gi|126640115|ref|NC_009085.1| | 729387 | 729410 | 24 |
| exprReg_5070 | gi|126640115|ref|NC_009085.1| | 729460 | 729478 | 19 |
| exprReg_5071 | gi|126640115|ref|NC_009085.1| | 729517 | 729576 | 60 |
| exprReg_5072 | gi|126640115|ref|NC_009085.1| | 729665 | 729934 | 270 |
| exprReg_5073 | gi|126640115|ref|NC_009085.1| | 729943 | 730174 | 232 |
| exprReg_5074 | gi|126640115|ref|NC_009085.1| | 730296 | 730313 | 18 |
| exprReg_5075 | gi|126640115|ref|NC_009085.1| | 730432 | 730448 | 17 |
| exprReg_5076 | gi|126640115|ref|NC_009085.1| | 730561 | 730584 | 24 |
| exprReg_5077 | gi|126640115|ref|NC_009085.1| | 730587 | 730639 | 53 |
| exprReg_5078 | gi|126640115|ref|NC_009085.1| | 730734 | 730979 | 246 |
| exprReg_5079 | gi|126640115|ref|NC_009085.1| | 731029 | 731046 | 18 |
| exprReg_5080 | gi|126640115|ref|NC_009085.1| | 731075 | 731261 | 187 |
| exprReg_5081 | gi|126640115|ref|NC_009085.1| | 731289 | 731314 | 26 |
| exprReg_5082 | gi|126640115|ref|NC_009085.1| | 731340 | 731420 | 81 |
| exprReg_5088 | gi|126640115|ref|NC_009085.1| | 732289 | 732319 | 31 |
| exprReg_5096 | gi|126640115|ref|NC_009085.1| | 733597 | 733680 | 84 |
| exprReg_5097 | gi|126640115|ref|NC_009085.1| | 733699 | 733841 | 143 |
| exprReg_5098 | gi|126640115|ref|NC_009085.1| | 733843 | 733864 | 22 |
| exprReg_5099 | gi|126640115|ref|NC_009085.1| | 733889 | 733910 | 22 |
| exprReg_5100 | gi|126640115|ref|NC_009085.1| | 733946 | 734118 | 173 |
| exprReg_5101 | gi|126640115|ref|NC_009085.1| | 734136 | 734192 | 57 |
| exprReg_5102 | gi|126640115|ref|NC_009085.1| | 734216 | 734242 | 27 |
| exprReg_5103 | gi|126640115|ref|NC_009085.1| | 734306 | 734389 | 84 |
| exprReg_5110 | gi|126640115|ref|NC_009085.1| | 735738 | 735761 | 24 |
| exprReg_5119 | gi|126640115|ref|NC_009085.1| | 737037 | 737072 | 36 |
| exprReg_5120 | gi|126640115|ref|NC_009085.1| | 737134 | 737164 | 31 |
| exprReg_5121 | gi|126640115|ref|NC_009085.1| | 737207 | 737238 | 32 |
| exprReg_5128 | gi|126640115|ref|NC_009085.1| | 737927 | 737949 | 23 |
| exprReg_5129 | gi|126640115|ref|NC_009085.1| | 737959 | 737975 | 17 |
| exprReg_5130 | gi|126640115|ref|NC_009085.1| | 738153 | 738342 | 190 |
| exprReg_5131 | gi|126640115|ref|NC_009085.1| | 738398 | 738418 | 21 |
| exprReg_5132 | gi|126640115|ref|NC_009085.1| | 738421 | 738437 | 17 |
| exprReg_5133 | gi|126640115|ref|NC_009085.1| | 738457 | 738658 | 202 |
| exprReg_5134 | gi|126640115|ref|NC_009085.1| | 738701 | 738799 | 99 |
| exprReg_5135 | gi|126640115|ref|NC_009085.1| | 738962 | 739055 | 94 |
| exprReg_5136 | gi|126640115|ref|NC_009085.1| | 739119 | 739197 | 79 |
| exprReg_5137 | gi|126640115|ref|NC_009085.1| | 739338 | 739531 | 194 |
| exprReg_5138 | gi|126640115|ref|NC_009085.1| | 739572 | 739588 | 17 |
| exprReg_5139 | gi|126640115|ref|NC_009085.1| | 739615 | 739632 | 18 |
| exprReg_5140 | gi|126640115|ref|NC_009085.1| | 739657 | 739882 | 226 |
| exprReg_5141 | gi|126640115|ref|NC_009085.1| | 739888 | 739939 | 52 |
| exprReg_5142 | gi|126640115|ref|NC_009085.1| | 740046 | 740141 | 96 |
| exprReg_5143 | gi|126640115|ref|NC_009085.1| | 740156 | 740302 | 147 |
| exprReg_5144 | gi|126640115|ref|NC_009085.1| | 740304 | 740325 | 22 |
| exprReg_5145 | gi|126640115|ref|NC_009085.1| | 740423 | 740595 | 173 |
| exprReg_5146 | gi|126640115|ref|NC_009085.1| | 740627 | 740645 | 19 |
| exprReg_5147 | gi|126640115|ref|NC_009085.1| | 740692 | 740898 | 207 |
| exprReg_5148 | gi|126640115|ref|NC_009085.1| | 740901 | 740938 | 38 |
| exprReg_5149 | gi|126640115|ref|NC_009085.1| | 740969 | 741036 | 68 |
| exprReg_5150 | gi|126640115|ref|NC_009085.1| | 741062 | 741157 | 96 |
| exprReg_5151 | gi|126640115|ref|NC_009085.1| | 741203 | 741411 | 209 |
| exprReg_5152 | gi|126640115|ref|NC_009085.1| | 741471 | 741593 | 123 |
| exprReg_5153 | gi|126640115|ref|NC_009085.1| | 741696 | 741832 | 137 |
| exprReg_5154 | gi|126640115|ref|NC_009085.1| | 742011 | 742177 | 167 |
| exprReg_5155 | gi|126640115|ref|NC_009085.1| | 742299 | 742316 | 18 |
| exprReg_5156 | gi|126640115|ref|NC_009085.1| | 742319 | 742427 | 109 |
| exprReg_5157 | gi|126640115|ref|NC_009085.1| | 742437 | 742454 | 18 |
| exprReg_5158 | gi|126640115|ref|NC_009085.1| | 742457 | 742543 | 87 |
| exprReg_5159 | gi|126640115|ref|NC_009085.1| | 742645 | 742662 | 18 |
| exprReg_5160 | gi|126640115|ref|NC_009085.1| | 742712 | 742730 | 19 |
| exprReg_5161 | gi|126640115|ref|NC_009085.1| | 742752 | 742772 | 21 |
| exprReg_5162 | gi|126640115|ref|NC_009085.1| | 742808 | 742902 | 95 |
| exprReg_5163 | gi|126640115|ref|NC_009085.1| | 743058 | 743125 | 68 |
| exprReg_5164 | gi|126640115|ref|NC_009085.1| | 743137 | 743164 | 28 |
| exprReg_5165 | gi|126640115|ref|NC_009085.1| | 743196 | 743433 | 238 |
| exprReg_5166 | gi|126640115|ref|NC_009085.1| | 743605 | 743622 | 18 |
| exprReg_5167 | gi|126640115|ref|NC_009085.1| | 743655 | 743871 | 217 |
| exprReg_5168 | gi|126640115|ref|NC_009085.1| | 743902 | 743921 | 20 |
| exprReg_5169 | gi|126640115|ref|NC_009085.1| | 743966 | 744059 | 94 |
| exprReg_5170 | gi|126640115|ref|NC_009085.1| | 744093 | 744112 | 20 |
| exprReg_5171 | gi|126640115|ref|NC_009085.1| | 744114 | 744226 | 113 |
| exprReg_5172 | gi|126640115|ref|NC_009085.1| | 744244 | 744508 | 265 |
| exprReg_5173 | gi|126640115|ref|NC_009085.1| | 744525 | 744552 | 28 |
| exprReg_5174 | gi|126640115|ref|NC_009085.1| | 744587 | 744662 | 76 |
| exprReg_5175 | gi|126640115|ref|NC_009085.1| | 744746 | 744764 | 19 |
| exprReg_5176 | gi|126640115|ref|NC_009085.1| | 744799 | 744818 | 20 |
| exprReg_5186 | gi|126640115|ref|NC_009085.1| | 746083 | 746112 | 30 |
| exprReg_5187 | gi|126640115|ref|NC_009085.1| | 746126 | 746190 | 65 |
| exprReg_5188 | gi|126640115|ref|NC_009085.1| | 746216 | 746256 | 41 |
| exprReg_5189 | gi|126640115|ref|NC_009085.1| | 746313 | 746585 | 273 |
| exprReg_5190 | gi|126640115|ref|NC_009085.1| | 746718 | 746785 | 68 |
| exprReg_5191 | gi|126640115|ref|NC_009085.1| | 746796 | 746975 | 180 |
| exprReg_5192 | gi|126640115|ref|NC_009085.1| | 747020 | 747091 | 72 |
| exprReg_5193 | gi|126640115|ref|NC_009085.1| | 747095 | 747116 | 22 |
| exprReg_5194 | gi|126640115|ref|NC_009085.1| | 747148 | 747346 | 199 |
| exprReg_5195 | gi|126640115|ref|NC_009085.1| | 747351 | 747387 | 37 |
| exprReg_5196 | gi|126640115|ref|NC_009085.1| | 747401 | 747450 | 50 |
| exprReg_5197 | gi|126640115|ref|NC_009085.1| | 747558 | 747598 | 41 |
| exprReg_5198 | gi|126640115|ref|NC_009085.1| | 747676 | 747891 | 216 |
| exprReg_5199 | gi|126640115|ref|NC_009085.1| | 747898 | 747991 | 94 |
| exprReg_5200 | gi|126640115|ref|NC_009085.1| | 748010 | 748038 | 29 |
| exprReg_5201 | gi|126640115|ref|NC_009085.1| | 748153 | 748446 | 294 |
| exprReg_5202 | gi|126640115|ref|NC_009085.1| | 748574 | 748628 | 55 |
| exprReg_5203 | gi|126640115|ref|NC_009085.1| | 748648 | 749027 | 380 |
| exprReg_5204 | gi|126640115|ref|NC_009085.1| | 749032 | 749075 | 44 |
| exprReg_5205 | gi|126640115|ref|NC_009085.1| | 749135 | 749191 | 57 |
| exprReg_5206 | gi|126640115|ref|NC_009085.1| | 749194 | 749222 | 29 |
| exprReg_5207 | gi|126640115|ref|NC_009085.1| | 749257 | 749551 | 295 |
| exprReg_5208 | gi|126640115|ref|NC_009085.1| | 749586 | 749634 | 49 |
| exprReg_5209 | gi|126640115|ref|NC_009085.1| | 749681 | 749825 | 145 |
| exprReg_5210 | gi|126640115|ref|NC_009085.1| | 749832 | 750115 | 284 |
| exprReg_5211 | gi|126640115|ref|NC_009085.1| | 750141 | 750193 | 53 |
| exprReg_5212 | gi|126640115|ref|NC_009085.1| | 750249 | 750296 | 48 |
| exprReg_5213 | gi|126640115|ref|NC_009085.1| | 750336 | 750355 | 20 |
| exprReg_5214 | gi|126640115|ref|NC_009085.1| | 750388 | 750424 | 37 |
| exprReg_5215 | gi|126640115|ref|NC_009085.1| | 750497 | 750525 | 29 |
| exprReg_5216 | gi|126640115|ref|NC_009085.1| | 750561 | 750633 | 73 |
| exprReg_5217 | gi|126640115|ref|NC_009085.1| | 750685 | 750742 | 58 |
| exprReg_5218 | gi|126640115|ref|NC_009085.1| | 750775 | 750812 | 38 |
| exprReg_5219 | gi|126640115|ref|NC_009085.1| | 751000 | 751028 | 29 |
| exprReg_5220 | gi|126640115|ref|NC_009085.1| | 751074 | 751142 | 69 |
| exprReg_5221 | gi|126640115|ref|NC_009085.1| | 751167 | 751189 | 23 |
| exprReg_5222 | gi|126640115|ref|NC_009085.1| | 751193 | 751230 | 38 |
| exprReg_5223 | gi|126640115|ref|NC_009085.1| | 751369 | 751395 | 27 |
| exprReg_5224 | gi|126640115|ref|NC_009085.1| | 751447 | 751510 | 64 |
| exprReg_5247 | gi|126640115|ref|NC_009085.1| | 754854 | 755011 | 158 |
| exprReg_5248 | gi|126640115|ref|NC_009085.1| | 755120 | 755161 | 42 |
| exprReg_5249 | gi|126640115|ref|NC_009085.1| | 755214 | 755234 | 21 |
| exprReg_5263 | gi|126640115|ref|NC_009085.1| | 756720 | 756776 | 57 |
| exprReg_5264 | gi|126640115|ref|NC_009085.1| | 756795 | 756848 | 54 |
| exprReg_5265 | gi|126640115|ref|NC_009085.1| | 756869 | 756921 | 53 |
| exprReg_5266 | gi|126640115|ref|NC_009085.1| | 757100 | 757190 | 91 |
| exprReg_5267 | gi|126640115|ref|NC_009085.1| | 757270 | 757287 | 18 |
| exprReg_5268 | gi|126640115|ref|NC_009085.1| | 757365 | 757387 | 23 |
| exprReg_5269 | gi|126640115|ref|NC_009085.1| | 757457 | 757501 | 45 |
| exprReg_5270 | gi|126640115|ref|NC_009085.1| | 757508 | 757645 | 138 |
| exprReg_5271 | gi|126640115|ref|NC_009085.1| | 757648 | 757726 | 79 |
| exprReg_5272 | gi|126640115|ref|NC_009085.1| | 757889 | 758082 | 194 |
| exprReg_5273 | gi|126640115|ref|NC_009085.1| | 758090 | 758107 | 18 |
| exprReg_5274 | gi|126640115|ref|NC_009085.1| | 758679 | 758820 | 142 |
| exprReg_5275 | gi|126640115|ref|NC_009085.1| | 758826 | 758920 | 95 |
| exprReg_5276 | gi|126640115|ref|NC_009085.1| | 759915 | 759944 | 30 |
| exprReg_5277 | gi|126640115|ref|NC_009085.1| | 759968 | 760016 | 49 |
| exprReg_5281 | gi|126640115|ref|NC_009085.1| | 760625 | 760656 | 32 |
| exprReg_5282 | gi|126640115|ref|NC_009085.1| | 760762 | 760827 | 66 |
| exprReg_5283 | gi|126640115|ref|NC_009085.1| | 761020 | 761037 | 18 |
| exprReg_5284 | gi|126640115|ref|NC_009085.1| | 761133 | 761151 | 19 |
| exprReg_5285 | gi|126640115|ref|NC_009085.1| | 761310 | 761336 | 27 |
| exprReg_5286 | gi|126640115|ref|NC_009085.1| | 761346 | 761400 | 55 |
| exprReg_5287 | gi|126640115|ref|NC_009085.1| | 761412 | 761428 | 17 |
| exprReg_5288 | gi|126640115|ref|NC_009085.1| | 761535 | 761554 | 20 |
| exprReg_5289 | gi|126640115|ref|NC_009085.1| | 761627 | 761649 | 23 |
| exprReg_5290 | gi|126640115|ref|NC_009085.1| | 761741 | 761758 | 18 |
| exprReg_5291 | gi|126640115|ref|NC_009085.1| | 761811 | 761987 | 177 |
| exprReg_5292 | gi|126640115|ref|NC_009085.1| | 762035 | 762051 | 17 |
| exprReg_5293 | gi|126640115|ref|NC_009085.1| | 762156 | 762176 | 21 |
| exprReg_5306 | gi|126640115|ref|NC_009085.1| | 763935 | 764133 | 199 |
| exprReg_5307 | gi|126640115|ref|NC_009085.1| | 764316 | 764647 | 332 |
| exprReg_5308 | gi|126640115|ref|NC_009085.1| | 764697 | 764767 | 71 |
| exprReg_5309 | gi|126640115|ref|NC_009085.1| | 764910 | 764948 | 39 |
| exprReg_5310 | gi|126640115|ref|NC_009085.1| | 764953 | 765008 | 56 |
| exprReg_5311 | gi|126640115|ref|NC_009085.1| | 765024 | 765041 | 18 |
| exprReg_5312 | gi|126640115|ref|NC_009085.1| | 765299 | 765316 | 18 |
| exprReg_5313 | gi|126640115|ref|NC_009085.1| | 765505 | 765528 | 24 |
| exprReg_5314 | gi|126640115|ref|NC_009085.1| | 765655 | 765737 | 83 |
| exprReg_5315 | gi|126640115|ref|NC_009085.1| | 765762 | 765780 | 19 |
| exprReg_5316 | gi|126640115|ref|NC_009085.1| | 765812 | 765866 | 55 |
| exprReg_5317 | gi|126640115|ref|NC_009085.1| | 765874 | 765890 | 17 |
| exprReg_5318 | gi|126640115|ref|NC_009085.1| | 766063 | 766082 | 20 |
| exprReg_5327 | gi|126640115|ref|NC_009085.1| | 767192 | 767318 | 127 |
| exprReg_5328 | gi|126640115|ref|NC_009085.1| | 767377 | 767405 | 29 |
| exprReg_5329 | gi|126640115|ref|NC_009085.1| | 767493 | 767509 | 17 |
| exprReg_5330 | gi|126640115|ref|NC_009085.1| | 767512 | 767591 | 80 |
| exprReg_5331 | gi|126640115|ref|NC_009085.1| | 767763 | 767780 | 18 |
| exprReg_5332 | gi|126640115|ref|NC_009085.1| | 767822 | 767996 | 175 |
| exprReg_5333 | gi|126640115|ref|NC_009085.1| | 768173 | 768190 | 18 |
| exprReg_5334 | gi|126640115|ref|NC_009085.1| | 768224 | 768253 | 30 |
| exprReg_5335 | gi|126640115|ref|NC_009085.1| | 768338 | 768381 | 44 |
| exprReg_5336 | gi|126640115|ref|NC_009085.1| | 768454 | 768540 | 87 |
| exprReg_5337 | gi|126640115|ref|NC_009085.1| | 768542 | 768659 | 118 |
| exprReg_5338 | gi|126640115|ref|NC_009085.1| | 768663 | 768688 | 26 |
| exprReg_5339 | gi|126640115|ref|NC_009085.1| | 768762 | 769138 | 377 |
| exprReg_5340 | gi|126640115|ref|NC_009085.1| | 769283 | 769538 | 256 |
| exprReg_5341 | gi|126640115|ref|NC_009085.1| | 769550 | 769594 | 45 |
| exprReg_5342 | gi|126640115|ref|NC_009085.1| | 769674 | 769752 | 79 |
| exprReg_5343 | gi|126640115|ref|NC_009085.1| | 769896 | 769917 | 22 |
| exprReg_5344 | gi|126640115|ref|NC_009085.1| | 769940 | 769956 | 17 |
| exprReg_5345 | gi|126640115|ref|NC_009085.1| | 769981 | 770073 | 93 |
| exprReg_5346 | gi|126640115|ref|NC_009085.1| | 770078 | 770094 | 17 |
| exprReg_5347 | gi|126640115|ref|NC_009085.1| | 770178 | 770200 | 23 |
| exprReg_5348 | gi|126640115|ref|NC_009085.1| | 770224 | 770240 | 17 |
| exprReg_5349 | gi|126640115|ref|NC_009085.1| | 770248 | 770266 | 19 |
| exprReg_5350 | gi|126640115|ref|NC_009085.1| | 770303 | 770322 | 20 |
| exprReg_5351 | gi|126640115|ref|NC_009085.1| | 770491 | 770550 | 60 |
| exprReg_5352 | gi|126640115|ref|NC_009085.1| | 770556 | 770596 | 41 |
| exprReg_5353 | gi|126640115|ref|NC_009085.1| | 770646 | 770667 | 22 |
| exprReg_5371 | gi|126640115|ref|NC_009085.1| | 772834 | 772860 | 27 |
| exprReg_5372 | gi|126640115|ref|NC_009085.1| | 772922 | 772941 | 20 |
| exprReg_5373 | gi|126640115|ref|NC_009085.1| | 772955 | 773010 | 56 |
| exprReg_5374 | gi|126640115|ref|NC_009085.1| | 773091 | 773126 | 36 |
| exprReg_5375 | gi|126640115|ref|NC_009085.1| | 773153 | 773187 | 35 |
| exprReg_5376 | gi|126640115|ref|NC_009085.1| | 773360 | 773456 | 97 |
| exprReg_5377 | gi|126640115|ref|NC_009085.1| | 773892 | 773913 | 22 |
| exprReg_5378 | gi|126640115|ref|NC_009085.1| | 773939 | 773961 | 23 |
| exprReg_5379 | gi|126640115|ref|NC_009085.1| | 773985 | 774128 | 144 |
| exprReg_5380 | gi|126640115|ref|NC_009085.1| | 774165 | 774185 | 21 |
| exprReg_5381 | gi|126640115|ref|NC_009085.1| | 774292 | 774479 | 188 |
| exprReg_5382 | gi|126640115|ref|NC_009085.1| | 774501 | 774517 | 17 |
| exprReg_5383 | gi|126640115|ref|NC_009085.1| | 774541 | 774806 | 266 |
| exprReg_5384 | gi|126640115|ref|NC_009085.1| | 774880 | 775007 | 128 |
| exprReg_5385 | gi|126640115|ref|NC_009085.1| | 775013 | 775052 | 40 |
| exprReg_5386 | gi|126640115|ref|NC_009085.1| | 775058 | 775178 | 121 |
| exprReg_5387 | gi|126640115|ref|NC_009085.1| | 775209 | 775252 | 44 |
| exprReg_5388 | gi|126640115|ref|NC_009085.1| | 775336 | 775356 | 21 |
| exprReg_5389 | gi|126640115|ref|NC_009085.1| | 775459 | 775476 | 18 |
| exprReg_5390 | gi|126640115|ref|NC_009085.1| | 775753 | 775794 | 42 |
| exprReg_5391 | gi|126640115|ref|NC_009085.1| | 775812 | 775828 | 17 |
| exprReg_5392 | gi|126640115|ref|NC_009085.1| | 775833 | 775852 | 20 |
| exprReg_5393 | gi|126640115|ref|NC_009085.1| | 775890 | 775968 | 79 |
| exprReg_5411 | gi|126640115|ref|NC_009085.1| | 779668 | 779752 | 85 |
| exprReg_5412 | gi|126640115|ref|NC_009085.1| | 779810 | 779853 | 44 |
| exprReg_5419 | gi|126640115|ref|NC_009085.1| | 781045 | 781108 | 64 |
| exprReg_5423 | gi|126640115|ref|NC_009085.1| | 781549 | 781655 | 107 |
| exprReg_5428 | gi|126640115|ref|NC_009085.1| | 782361 | 782378 | 18 |
| exprReg_5437 | gi|126640115|ref|NC_009085.1| | 783644 | 783743 | 100 |
| exprReg_5443 | gi|126640115|ref|NC_009085.1| | 784563 | 784612 | 50 |
| exprReg_5444 | gi|126640115|ref|NC_009085.1| | 784662 | 784696 | 35 |
| exprReg_5462 | gi|126640115|ref|NC_009085.1| | 788189 | 788297 | 109 |
| exprReg_5463 | gi|126640115|ref|NC_009085.1| | 788340 | 788538 | 199 |
| exprReg_5464 | gi|126640115|ref|NC_009085.1| | 788603 | 788800 | 198 |
| exprReg_5465 | gi|126640115|ref|NC_009085.1| | 788969 | 789351 | 383 |
| exprReg_5483 | gi|126640115|ref|NC_009085.1| | 795059 | 795568 | 510 |
| exprReg_5484 | gi|126640115|ref|NC_009085.1| | 795585 | 795666 | 82 |
| exprReg_5485 | gi|126640115|ref|NC_009085.1| | 795702 | 795753 | 52 |
| exprReg_5488 | gi|126640115|ref|NC_009085.1| | 796593 | 796669 | 77 |
| exprReg_5489 | gi|126640115|ref|NC_009085.1| | 796679 | 796700 | 22 |
| exprReg_5490 | gi|126640115|ref|NC_009085.1| | 796721 | 796933 | 213 |
| exprReg_5491 | gi|126640115|ref|NC_009085.1| | 796943 | 796959 | 17 |
| exprReg_5498 | gi|126640115|ref|NC_009085.1| | 799756 | 799861 | 106 |
| exprReg_5504 | gi|126640115|ref|NC_009085.1| | 800743 | 800821 | 79 |
| exprReg_5505 | gi|126640115|ref|NC_009085.1| | 800862 | 800919 | 58 |
| exprReg_5509 | gi|126640115|ref|NC_009085.1| | 812537 | 812579 | 43 |
| exprReg_5510 | gi|126640115|ref|NC_009085.1| | 812597 | 812613 | 17 |
| exprReg_5520 | gi|126640115|ref|NC_009085.1| | 814224 | 814240 | 17 |
| exprReg_5521 | gi|126640115|ref|NC_009085.1| | 814303 | 814331 | 29 |
| exprReg_5531 | gi|126640115|ref|NC_009085.1| | 816340 | 816360 | 21 |
| exprReg_5532 | gi|126640115|ref|NC_009085.1| | 816379 | 816468 | 90 |
| exprReg_5538 | gi|126640115|ref|NC_009085.1| | 817048 | 817100 | 53 |
| exprReg_5539 | gi|126640115|ref|NC_009085.1| | 817127 | 817257 | 131 |
| exprReg_5560 | gi|126640115|ref|NC_009085.1| | 820891 | 820909 | 19 |
| exprReg_5561 | gi|126640115|ref|NC_009085.1| | 821014 | 821094 | 81 |
| exprReg_5564 | gi|126640115|ref|NC_009085.1| | 822081 | 822132 | 52 |
| exprReg_5565 | gi|126640115|ref|NC_009085.1| | 822354 | 822376 | 23 |
| exprReg_5566 | gi|126640115|ref|NC_009085.1| | 822406 | 822453 | 48 |
| exprReg_5567 | gi|126640115|ref|NC_009085.1| | 822462 | 822628 | 167 |
| exprReg_5574 | gi|126640115|ref|NC_009085.1| | 823435 | 823531 | 97 |
| exprReg_5575 | gi|126640115|ref|NC_009085.1| | 823600 | 823621 | 22 |
| exprReg_5630 | gi|126640115|ref|NC_009085.1| | 831201 | 831236 | 36 |
| exprReg_5631 | gi|126640115|ref|NC_009085.1| | 831247 | 831358 | 112 |
| exprReg_5636 | gi|126640115|ref|NC_009085.1| | 831965 | 832012 | 48 |
| exprReg_5637 | gi|126640115|ref|NC_009085.1| | 832049 | 832126 | 78 |
| exprReg_5642 | gi|126640115|ref|NC_009085.1| | 832809 | 832887 | 79 |
| exprReg_5650 | gi|126640115|ref|NC_009085.1| | 834029 | 834055 | 27 |
| exprReg_5655 | gi|126640115|ref|NC_009085.1| | 834402 | 834445 | 44 |
| exprReg_5682 | gi|126640115|ref|NC_009085.1| | 837562 | 837776 | 215 |
| exprReg_5683 | gi|126640115|ref|NC_009085.1| | 837807 | 838056 | 250 |
| exprReg_5684 | gi|126640115|ref|NC_009085.1| | 838171 | 838197 | 27 |
| exprReg_5706 | gi|126640115|ref|NC_009085.1| | 840794 | 840863 | 70 |
| exprReg_5707 | gi|126640115|ref|NC_009085.1| | 840872 | 840936 | 65 |
| exprReg_5716 | gi|126640115|ref|NC_009085.1| | 841829 | 841863 | 35 |
| exprReg_5717 | gi|126640115|ref|NC_009085.1| | 841874 | 841894 | 21 |
| exprReg_5723 | gi|126640115|ref|NC_009085.1| | 842718 | 842774 | 57 |
| exprReg_5724 | gi|126640115|ref|NC_009085.1| | 842811 | 842854 | 44 |
| exprReg_5725 | gi|126640115|ref|NC_009085.1| | 842896 | 842925 | 30 |
| exprReg_5726 | gi|126640115|ref|NC_009085.1| | 842959 | 843041 | 83 |
| exprReg_5733 | gi|126640115|ref|NC_009085.1| | 844124 | 844180 | 57 |
| exprReg_5734 | gi|126640115|ref|NC_009085.1| | 844189 | 844210 | 22 |
| exprReg_5735 | gi|126640115|ref|NC_009085.1| | 844319 | 844344 | 26 |
| exprReg_5746 | gi|126640115|ref|NC_009085.1| | 847104 | 847122 | 19 |
| exprReg_5747 | gi|126640115|ref|NC_009085.1| | 847141 | 847329 | 189 |
| exprReg_5748 | gi|126640115|ref|NC_009085.1| | 847412 | 847429 | 18 |
| exprReg_5761 | gi|126640115|ref|NC_009085.1| | 849443 | 849568 | 126 |
| exprReg_5780 | gi|126640115|ref|NC_009085.1| | 852625 | 852641 | 17 |
| exprReg_5788 | gi|126640115|ref|NC_009085.1| | 853868 | 853887 | 20 |
| exprReg_5805 | gi|126640115|ref|NC_009085.1| | 856397 | 856433 | 37 |
| exprReg_5821 | gi|126640115|ref|NC_009085.1| | 859748 | 859877 | 130 |
| exprReg_5830 | gi|126640115|ref|NC_009085.1| | 861096 | 861133 | 38 |
| exprReg_5839 | gi|126640115|ref|NC_009085.1| | 862830 | 862887 | 58 |
| exprReg_5857 | gi|126640115|ref|NC_009085.1| | 866513 | 866553 | 41 |
| exprReg_5861 | gi|126640115|ref|NC_009085.1| | 867002 | 867209 | 208 |
| exprReg_5862 | gi|126640115|ref|NC_009085.1| | 867474 | 867562 | 89 |
| exprReg_5866 | gi|126640115|ref|NC_009085.1| | 868551 | 868587 | 37 |
| exprReg_5881 | gi|126640115|ref|NC_009085.1| | 871472 | 871833 | 362 |
| exprReg_5882 | gi|126640115|ref|NC_009085.1| | 872010 | 872197 | 188 |
| exprReg_5883 | gi|126640115|ref|NC_009085.1| | 872207 | 872271 | 65 |
| exprReg_5884 | gi|126640115|ref|NC_009085.1| | 872291 | 872348 | 58 |
| exprReg_5885 | gi|126640115|ref|NC_009085.1| | 872360 | 872383 | 24 |
| exprReg_5886 | gi|126640115|ref|NC_009085.1| | 872405 | 872465 | 61 |
| exprReg_5898 | gi|126640115|ref|NC_009085.1| | 874416 | 874444 | 29 |
| exprReg_5901 | gi|126640115|ref|NC_009085.1| | 874840 | 874859 | 20 |
| exprReg_5902 | gi|126640115|ref|NC_009085.1| | 874917 | 874947 | 31 |
| exprReg_5903 | gi|126640115|ref|NC_009085.1| | 874980 | 875006 | 27 |
| exprReg_5904 | gi|126640115|ref|NC_009085.1| | 875033 | 875067 | 35 |
| exprReg_5905 | gi|126640115|ref|NC_009085.1| | 875144 | 875246 | 103 |
| exprReg_5906 | gi|126640115|ref|NC_009085.1| | 875283 | 875331 | 49 |
| exprReg_5907 | gi|126640115|ref|NC_009085.1| | 875340 | 875382 | 43 |
| exprReg_5908 | gi|126640115|ref|NC_009085.1| | 875411 | 875432 | 22 |
| exprReg_5909 | gi|126640115|ref|NC_009085.1| | 875440 | 875461 | 22 |
| exprReg_5910 | gi|126640115|ref|NC_009085.1| | 875489 | 875596 | 108 |
| exprReg_5929 | gi|126640115|ref|NC_009085.1| | 878304 | 878330 | 27 |
| exprReg_5931 | gi|126640115|ref|NC_009085.1| | 878691 | 878713 | 23 |
| exprReg_5952 | gi|126640115|ref|NC_009085.1| | 882158 | 882178 | 21 |
| exprReg_5953 | gi|126640115|ref|NC_009085.1| | 882239 | 882329 | 91 |
| exprReg_5954 | gi|126640115|ref|NC_009085.1| | 882356 | 882380 | 25 |
| exprReg_5955 | gi|126640115|ref|NC_009085.1| | 882427 | 882446 | 20 |
| exprReg_5956 | gi|126640115|ref|NC_009085.1| | 882549 | 882576 | 28 |
| exprReg_5957 | gi|126640115|ref|NC_009085.1| | 882679 | 882729 | 51 |
| exprReg_5958 | gi|126640115|ref|NC_009085.1| | 882910 | 882973 | 64 |
| exprReg_5966 | gi|126640115|ref|NC_009085.1| | 884445 | 884535 | 91 |
| exprReg_5967 | gi|126640115|ref|NC_009085.1| | 884549 | 884611 | 63 |
| exprReg_5968 | gi|126640115|ref|NC_009085.1| | 884662 | 884683 | 22 |
| exprReg_5985 | gi|126640115|ref|NC_009085.1| | 887664 | 887684 | 21 |
| exprReg_5986 | gi|126640115|ref|NC_009085.1| | 887734 | 887792 | 59 |
| exprReg_5987 | gi|126640115|ref|NC_009085.1| | 887873 | 887889 | 17 |
| exprReg_5992 | gi|126640115|ref|NC_009085.1| | 888937 | 889162 | 226 |
| exprReg_6001 | gi|126640115|ref|NC_009085.1| | 890960 | 891015 | 56 |
| exprReg_6002 | gi|126640115|ref|NC_009085.1| | 891022 | 891081 | 60 |
| exprReg_6009 | gi|126640115|ref|NC_009085.1| | 892278 | 892321 | 44 |
| exprReg_6010 | gi|126640115|ref|NC_009085.1| | 892354 | 892377 | 24 |
| exprReg_6011 | gi|126640115|ref|NC_009085.1| | 892398 | 892434 | 37 |
| exprReg_6012 | gi|126640115|ref|NC_009085.1| | 892471 | 892544 | 74 |
| exprReg_6013 | gi|126640115|ref|NC_009085.1| | 892569 | 892588 | 20 |
| exprReg_6093 | gi|126640115|ref|NC_009085.1| | 904901 | 904938 | 38 |
| exprReg_6116 | gi|126640115|ref|NC_009085.1| | 908059 | 908113 | 55 |
| exprReg_6122 | gi|126640115|ref|NC_009085.1| | 909177 | 909195 | 19 |
| exprReg_6148 | gi|126640115|ref|NC_009085.1| | 911935 | 911965 | 31 |
| exprReg_6149 | gi|126640115|ref|NC_009085.1| | 912037 | 912054 | 18 |
| exprReg_6150 | gi|126640115|ref|NC_009085.1| | 912106 | 912155 | 50 |
| exprReg_6151 | gi|126640115|ref|NC_009085.1| | 912220 | 912305 | 86 |
| exprReg_6152 | gi|126640115|ref|NC_009085.1| | 912307 | 912534 | 228 |
| exprReg_6167 | gi|126640115|ref|NC_009085.1| | 914451 | 914499 | 49 |
| exprReg_6168 | gi|126640115|ref|NC_009085.1| | 914506 | 914523 | 18 |
| exprReg_6177 | gi|126640115|ref|NC_009085.1| | 915979 | 915999 | 21 |
| exprReg_6182 | gi|126640115|ref|NC_009085.1| | 916664 | 916721 | 58 |
| exprReg_6183 | gi|126640115|ref|NC_009085.1| | 916750 | 916767 | 18 |
| exprReg_6192 | gi|126640115|ref|NC_009085.1| | 917971 | 917988 | 18 |
| exprReg_6209 | gi|126640115|ref|NC_009085.1| | 920488 | 920507 | 20 |
| exprReg_6215 | gi|126640115|ref|NC_009085.1| | 921269 | 921290 | 22 |
| exprReg_6216 | gi|126640115|ref|NC_009085.1| | 921368 | 921385 | 18 |
| exprReg_6217 | gi|126640115|ref|NC_009085.1| | 921394 | 921417 | 24 |
| exprReg_6218 | gi|126640115|ref|NC_009085.1| | 921494 | 921541 | 48 |
| exprReg_6229 | gi|126640115|ref|NC_009085.1| | 923650 | 923683 | 34 |
| exprReg_6234 | gi|126640115|ref|NC_009085.1| | 924578 | 924610 | 33 |
| exprReg_6235 | gi|126640115|ref|NC_009085.1| | 924638 | 924655 | 18 |
| exprReg_6236 | gi|126640115|ref|NC_009085.1| | 924725 | 924744 | 20 |
| exprReg_6254 | gi|126640115|ref|NC_009085.1| | 927451 | 927511 | 61 |
| exprReg_6255 | gi|126640115|ref|NC_009085.1| | 927602 | 927636 | 35 |
| exprReg_6256 | gi|126640115|ref|NC_009085.1| | 927790 | 927826 | 37 |
| exprReg_6257 | gi|126640115|ref|NC_009085.1| | 927845 | 927869 | 25 |
| exprReg_6258 | gi|126640115|ref|NC_009085.1| | 927881 | 927904 | 24 |
| exprReg_6259 | gi|126640115|ref|NC_009085.1| | 927947 | 927970 | 24 |
| exprReg_6269 | gi|126640115|ref|NC_009085.1| | 929139 | 929167 | 29 |
| exprReg_6270 | gi|126640115|ref|NC_009085.1| | 929238 | 929256 | 19 |
| exprReg_6283 | gi|126640115|ref|NC_009085.1| | 930782 | 931005 | 224 |
| exprReg_6284 | gi|126640115|ref|NC_009085.1| | 931012 | 931031 | 20 |
| exprReg_6285 | gi|126640115|ref|NC_009085.1| | 931034 | 931069 | 36 |
| exprReg_6327 | gi|126640115|ref|NC_009085.1| | 936345 | 936552 | 208 |
| exprReg_6328 | gi|126640115|ref|NC_009085.1| | 936603 | 936629 | 27 |
| exprReg_6329 | gi|126640115|ref|NC_009085.1| | 936675 | 936751 | 77 |
| exprReg_6330 | gi|126640115|ref|NC_009085.1| | 936891 | 936977 | 87 |
| exprReg_6331 | gi|126640115|ref|NC_009085.1| | 937044 | 937073 | 30 |
| exprReg_6332 | gi|126640115|ref|NC_009085.1| | 937182 | 937265 | 84 |
| exprReg_6344 | gi|126640115|ref|NC_009085.1| | 939152 | 939201 | 50 |
| exprReg_6345 | gi|126640115|ref|NC_009085.1| | 939239 | 939460 | 222 |
| exprReg_6346 | gi|126640115|ref|NC_009085.1| | 939490 | 939544 | 55 |
| exprReg_6347 | gi|126640115|ref|NC_009085.1| | 939578 | 939657 | 80 |
| exprReg_6348 | gi|126640115|ref|NC_009085.1| | 939729 | 939768 | 40 |
| exprReg_6349 | gi|126640115|ref|NC_009085.1| | 939991 | 940021 | 31 |
| exprReg_6350 | gi|126640115|ref|NC_009085.1| | 940095 | 940205 | 111 |
| exprReg_6351 | gi|126640115|ref|NC_009085.1| | 940371 | 940606 | 236 |
| exprReg_6352 | gi|126640115|ref|NC_009085.1| | 940687 | 940747 | 61 |
| exprReg_6355 | gi|126640115|ref|NC_009085.1| | 941342 | 941414 | 73 |
| exprReg_6363 | gi|126640115|ref|NC_009085.1| | 942265 | 942456 | 192 |
| exprReg_6409 | gi|126640115|ref|NC_009085.1| | 948583 | 948654 | 72 |
| exprReg_6432 | gi|126640115|ref|NC_009085.1| | 951540 | 951638 | 99 |
| exprReg_6451 | gi|126640115|ref|NC_009085.1| | 954221 | 954241 | 21 |
| exprReg_6452 | gi|126640115|ref|NC_009085.1| | 954255 | 954336 | 82 |
| exprReg_6457 | gi|126640115|ref|NC_009085.1| | 955090 | 955258 | 169 |
| exprReg_6458 | gi|126640115|ref|NC_009085.1| | 955483 | 955503 | 21 |
| exprReg_6459 | gi|126640115|ref|NC_009085.1| | 955595 | 955645 | 51 |
| exprReg_6465 | gi|126640115|ref|NC_009085.1| | 956427 | 956467 | 41 |
| exprReg_6466 | gi|126640115|ref|NC_009085.1| | 956469 | 956486 | 18 |
| exprReg_6493 | gi|126640115|ref|NC_009085.1| | 959638 | 959678 | 41 |
| exprReg_6494 | gi|126640115|ref|NC_009085.1| | 959700 | 959736 | 37 |
| exprReg_6517 | gi|126640115|ref|NC_009085.1| | 962536 | 962553 | 18 |
| exprReg_6518 | gi|126640115|ref|NC_009085.1| | 962574 | 962808 | 235 |
| exprReg_6534 | gi|126640115|ref|NC_009085.1| | 965307 | 965335 | 29 |
| exprReg_6535 | gi|126640115|ref|NC_009085.1| | 965344 | 965382 | 39 |
| exprReg_6550 | gi|126640115|ref|NC_009085.1| | 966441 | 966480 | 40 |
| exprReg_6557 | gi|126640115|ref|NC_009085.1| | 967383 | 967438 | 56 |
| exprReg_6558 | gi|126640115|ref|NC_009085.1| | 967493 | 967681 | 189 |
| exprReg_6562 | gi|126640115|ref|NC_009085.1| | 968064 | 968082 | 19 |
| exprReg_6568 | gi|126640115|ref|NC_009085.1| | 968853 | 968872 | 20 |
| exprReg_6569 | gi|126640115|ref|NC_009085.1| | 968917 | 969004 | 88 |
| exprReg_6576 | gi|126640115|ref|NC_009085.1| | 969664 | 969681 | 18 |
| exprReg_6581 | gi|126640115|ref|NC_009085.1| | 970355 | 970374 | 20 |
| exprReg_6582 | gi|126640115|ref|NC_009085.1| | 970379 | 970595 | 217 |
| exprReg_6583 | gi|126640115|ref|NC_009085.1| | 970604 | 970622 | 19 |
| exprReg_6584 | gi|126640115|ref|NC_009085.1| | 970638 | 970657 | 20 |
| exprReg_6585 | gi|126640115|ref|NC_009085.1| | 970703 | 970719 | 17 |
| exprReg_6586 | gi|126640115|ref|NC_009085.1| | 970870 | 970896 | 27 |
| exprReg_6587 | gi|126640115|ref|NC_009085.1| | 970903 | 971041 | 139 |
| exprReg_6588 | gi|126640115|ref|NC_009085.1| | 971132 | 971164 | 33 |
| exprReg_6589 | gi|126640115|ref|NC_009085.1| | 971190 | 971206 | 17 |
| exprReg_6590 | gi|126640115|ref|NC_009085.1| | 971247 | 971277 | 31 |
| exprReg_6591 | gi|126640115|ref|NC_009085.1| | 971420 | 971460 | 41 |
| exprReg_6592 | gi|126640115|ref|NC_009085.1| | 971486 | 971545 | 60 |
| exprReg_6593 | gi|126640115|ref|NC_009085.1| | 971584 | 971655 | 72 |
| exprReg_6594 | gi|126640115|ref|NC_009085.1| | 971673 | 971692 | 20 |
| exprReg_6595 | gi|126640115|ref|NC_009085.1| | 971707 | 971726 | 20 |
| exprReg_6596 | gi|126640115|ref|NC_009085.1| | 971792 | 971810 | 19 |
| exprReg_6597 | gi|126640115|ref|NC_009085.1| | 971897 | 971996 | 100 |
| exprReg_6598 | gi|126640115|ref|NC_009085.1| | 972069 | 972110 | 42 |
| exprReg_6599 | gi|126640115|ref|NC_009085.1| | 972121 | 972207 | 87 |
| exprReg_6600 | gi|126640115|ref|NC_009085.1| | 972212 | 972258 | 47 |
| exprReg_6601 | gi|126640115|ref|NC_009085.1| | 972288 | 972308 | 21 |
| exprReg_6602 | gi|126640115|ref|NC_009085.1| | 972343 | 972432 | 90 |
| exprReg_6603 | gi|126640115|ref|NC_009085.1| | 972454 | 972475 | 22 |
| exprReg_6604 | gi|126640115|ref|NC_009085.1| | 972517 | 972543 | 27 |
| exprReg_6609 | gi|126640115|ref|NC_009085.1| | 973462 | 973521 | 60 |
| exprReg_6610 | gi|126640115|ref|NC_009085.1| | 973540 | 973644 | 105 |
| exprReg_6615 | gi|126640115|ref|NC_009085.1| | 974042 | 974066 | 25 |
| exprReg_6618 | gi|126640115|ref|NC_009085.1| | 974250 | 974273 | 24 |
| exprReg_6623 | gi|126640115|ref|NC_009085.1| | 974615 | 974667 | 53 |
| exprReg_6630 | gi|126640115|ref|NC_009085.1| | 975807 | 975828 | 22 |
| exprReg_6680 | gi|126640115|ref|NC_009085.1| | 982414 | 982500 | 87 |
| exprReg_6681 | gi|126640115|ref|NC_009085.1| | 982508 | 982557 | 50 |
| exprReg_6693 | gi|126640115|ref|NC_009085.1| | 983778 | 983957 | 180 |
| exprReg_6694 | gi|126640115|ref|NC_009085.1| | 983960 | 983977 | 18 |
| exprReg_6695 | gi|126640115|ref|NC_009085.1| | 983994 | 984011 | 18 |
| exprReg_6713 | gi|126640115|ref|NC_009085.1| | 986873 | 986937 | 65 |
| exprReg_6714 | gi|126640115|ref|NC_009085.1| | 987073 | 987168 | 96 |
| exprReg_6718 | gi|126640115|ref|NC_009085.1| | 987629 | 987791 | 163 |
| exprReg_6720 | gi|126640115|ref|NC_009085.1| | 988456 | 988490 | 35 |
| exprReg_6721 | gi|126640115|ref|NC_009085.1| | 988585 | 988657 | 73 |
| exprReg_6736 | gi|126640115|ref|NC_009085.1| | 990476 | 990547 | 72 |
| exprReg_6737 | gi|126640115|ref|NC_009085.1| | 990564 | 990632 | 69 |
| exprReg_6738 | gi|126640115|ref|NC_009085.1| | 990647 | 990668 | 22 |
| exprReg_6739 | gi|126640115|ref|NC_009085.1| | 990694 | 990809 | 116 |
| exprReg_6740 | gi|126640115|ref|NC_009085.1| | 990826 | 990891 | 66 |
| exprReg_6768 | gi|126640115|ref|NC_009085.1| | 995792 | 995808 | 17 |
| exprReg_6769 | gi|126640115|ref|NC_009085.1| | 995881 | 995962 | 82 |
| exprReg_6783 | gi|126640115|ref|NC_009085.1| | 997424 | 997446 | 23 |
| exprReg_6802 | gi|126640115|ref|NC_009085.1| | 1001228 | 1001244 | 17 |
| exprReg_6803 | gi|126640115|ref|NC_009085.1| | 1001258 | 1001342 | 85 |
| exprReg_6804 | gi|126640115|ref|NC_009085.1| | 1001412 | 1001444 | 33 |
| exprReg_6805 | gi|126640115|ref|NC_009085.1| | 1001552 | 1001568 | 17 |
| exprReg_6810 | gi|126640115|ref|NC_009085.1| | 1002032 | 1002068 | 37 |
| exprReg_6815 | gi|126640115|ref|NC_009085.1| | 1002993 | 1003090 | 98 |
| exprReg_6827 | gi|126640115|ref|NC_009085.1| | 1005010 | 1005108 | 99 |
| exprReg_6828 | gi|126640115|ref|NC_009085.1| | 1005170 | 1005188 | 19 |
| exprReg_6829 | gi|126640115|ref|NC_009085.1| | 1005216 | 1005351 | 136 |
| exprReg_6830 | gi|126640115|ref|NC_009085.1| | 1005373 | 1005393 | 21 |
| exprReg_6841 | gi|126640115|ref|NC_009085.1| | 1006667 | 1006771 | 105 |
| exprReg_6842 | gi|126640115|ref|NC_009085.1| | 1006789 | 1006806 | 18 |
| exprReg_6867 | gi|126640115|ref|NC_009085.1| | 1012554 | 1012597 | 44 |
| exprReg_6873 | gi|126640115|ref|NC_009085.1| | 1013575 | 1013640 | 66 |
| exprReg_6893 | gi|126640115|ref|NC_009085.1| | 1015541 | 1015676 | 136 |
| exprReg_6894 | gi|126640115|ref|NC_009085.1| | 1015701 | 1015742 | 42 |
| exprReg_6895 | gi|126640115|ref|NC_009085.1| | 1015760 | 1015803 | 44 |
| exprReg_6901 | gi|126640115|ref|NC_009085.1| | 1016732 | 1016778 | 47 |
| exprReg_6902 | gi|126640115|ref|NC_009085.1| | 1016803 | 1016819 | 17 |
| exprReg_6903 | gi|126640115|ref|NC_009085.1| | 1016868 | 1016886 | 19 |
| exprReg_6904 | gi|126640115|ref|NC_009085.1| | 1016932 | 1017048 | 117 |
| exprReg_6931 | gi|126640115|ref|NC_009085.1| | 1021246 | 1021266 | 21 |
| exprReg_6947 | gi|126640115|ref|NC_009085.1| | 1022998 | 1023068 | 71 |
| exprReg_6948 | gi|126640115|ref|NC_009085.1| | 1023093 | 1023219 | 127 |
| exprReg_6964 | gi|126640115|ref|NC_009085.1| | 1026366 | 1026384 | 19 |
| exprReg_6965 | gi|126640115|ref|NC_009085.1| | 1026387 | 1026405 | 19 |
| exprReg_6966 | gi|126640115|ref|NC_009085.1| | 1026448 | 1026496 | 49 |
| exprReg_6967 | gi|126640115|ref|NC_009085.1| | 1026504 | 1026523 | 20 |
| exprReg_6991 | gi|126640115|ref|NC_009085.1| | 1028936 | 1028961 | 26 |
| exprReg_6992 | gi|126640115|ref|NC_009085.1| | 1029011 | 1029035 | 25 |
| exprReg_6993 | gi|126640115|ref|NC_009085.1| | 1029088 | 1029116 | 29 |
| exprReg_6994 | gi|126640115|ref|NC_009085.1| | 1029126 | 1029142 | 17 |
| exprReg_6995 | gi|126640115|ref|NC_009085.1| | 1029160 | 1029186 | 27 |
| exprReg_6996 | gi|126640115|ref|NC_009085.1| | 1029221 | 1029238 | 18 |
| exprReg_6997 | gi|126640115|ref|NC_009085.1| | 1029250 | 1029276 | 27 |
| exprReg_6998 | gi|126640115|ref|NC_009085.1| | 1029304 | 1029345 | 42 |
| exprReg_7064 | gi|126640115|ref|NC_009085.1| | 1038840 | 1038859 | 20 |
| exprReg_7065 | gi|126640115|ref|NC_009085.1| | 1038864 | 1038999 | 136 |
| exprReg_7101 | gi|126640115|ref|NC_009085.1| | 1044621 | 1044654 | 34 |
| exprReg_7102 | gi|126640115|ref|NC_009085.1| | 1044680 | 1044716 | 37 |
| exprReg_7107 | gi|126640115|ref|NC_009085.1| | 1045343 | 1045361 | 19 |
| exprReg_7111 | gi|126640115|ref|NC_009085.1| | 1045732 | 1045766 | 35 |
| exprReg_7112 | gi|126640115|ref|NC_009085.1| | 1045778 | 1046079 | 302 |
| exprReg_7140 | gi|126640115|ref|NC_009085.1| | 1050493 | 1050555 | 63 |
| exprReg_7141 | gi|126640115|ref|NC_009085.1| | 1050572 | 1050627 | 56 |
| exprReg_7161 | gi|126640115|ref|NC_009085.1| | 1053505 | 1053523 | 19 |
| exprReg_7162 | gi|126640115|ref|NC_009085.1| | 1053594 | 1053658 | 65 |
| exprReg_7163 | gi|126640115|ref|NC_009085.1| | 1053705 | 1053758 | 54 |
| exprReg_7178 | gi|126640115|ref|NC_009085.1| | 1055718 | 1056190 | 473 |
| exprReg_7179 | gi|126640115|ref|NC_009085.1| | 1056238 | 1056283 | 46 |
| exprReg_7202 | gi|126640115|ref|NC_009085.1| | 1059386 | 1059412 | 27 |
| exprReg_7203 | gi|126640115|ref|NC_009085.1| | 1059464 | 1059648 | 185 |
| exprReg_7204 | gi|126640115|ref|NC_009085.1| | 1059658 | 1059816 | 159 |
| exprReg_7205 | gi|126640115|ref|NC_009085.1| | 1059852 | 1059873 | 22 |
| exprReg_7206 | gi|126640115|ref|NC_009085.1| | 1059893 | 1059955 | 63 |
| exprReg_7207 | gi|126640115|ref|NC_009085.1| | 1060072 | 1060095 | 24 |
| exprReg_7208 | gi|126640115|ref|NC_009085.1| | 1060103 | 1060362 | 260 |
| exprReg_7209 | gi|126640115|ref|NC_009085.1| | 1060614 | 1060639 | 26 |
| exprReg_7210 | gi|126640115|ref|NC_009085.1| | 1060768 | 1060836 | 69 |
| exprReg_7211 | gi|126640115|ref|NC_009085.1| | 1060839 | 1061008 | 170 |
| exprReg_7212 | gi|126640115|ref|NC_009085.1| | 1061023 | 1061193 | 171 |
| exprReg_7213 | gi|126640115|ref|NC_009085.1| | 1061378 | 1061471 | 94 |
| exprReg_7214 | gi|126640115|ref|NC_009085.1| | 1061612 | 1061628 | 17 |
| exprReg_7215 | gi|126640115|ref|NC_009085.1| | 1061676 | 1061800 | 125 |
| exprReg_7216 | gi|126640115|ref|NC_009085.1| | 1061868 | 1061903 | 36 |
| exprReg_7230 | gi|126640115|ref|NC_009085.1| | 1064358 | 1064572 | 215 |
| exprReg_7231 | gi|126640115|ref|NC_009085.1| | 1064577 | 1064653 | 77 |
| exprReg_7237 | gi|126640115|ref|NC_009085.1| | 1065515 | 1065575 | 61 |
| exprReg_7238 | gi|126640115|ref|NC_009085.1| | 1065589 | 1065626 | 38 |
| exprReg_7239 | gi|126640115|ref|NC_009085.1| | 1065669 | 1065687 | 19 |
| exprReg_7240 | gi|126640115|ref|NC_009085.1| | 1065735 | 1065757 | 23 |
| exprReg_7241 | gi|126640115|ref|NC_009085.1| | 1065850 | 1065982 | 133 |
| exprReg_7272 | gi|126640115|ref|NC_009085.1| | 1070242 | 1070270 | 29 |
| exprReg_7278 | gi|126640115|ref|NC_009085.1| | 1071134 | 1071194 | 61 |
| exprReg_7279 | gi|126640115|ref|NC_009085.1| | 1071283 | 1071377 | 95 |
| exprReg_7280 | gi|126640115|ref|NC_009085.1| | 1071612 | 1071628 | 17 |
| exprReg_7281 | gi|126640115|ref|NC_009085.1| | 1071735 | 1071791 | 57 |
| exprReg_7294 | gi|126640115|ref|NC_009085.1| | 1073607 | 1073634 | 28 |
| exprReg_7295 | gi|126640115|ref|NC_009085.1| | 1073647 | 1073724 | 78 |
| exprReg_7296 | gi|126640115|ref|NC_009085.1| | 1073741 | 1073849 | 109 |
| exprReg_7297 | gi|126640115|ref|NC_009085.1| | 1073971 | 1074008 | 38 |
| exprReg_7298 | gi|126640115|ref|NC_009085.1| | 1074051 | 1074071 | 21 |
| exprReg_7306 | gi|126640115|ref|NC_009085.1| | 1074942 | 1074958 | 17 |
| exprReg_7322 | gi|126640115|ref|NC_009085.1| | 1077914 | 1077933 | 20 |
| exprReg_7323 | gi|126640115|ref|NC_009085.1| | 1077978 | 1077995 | 18 |
| exprReg_7324 | gi|126640115|ref|NC_009085.1| | 1078082 | 1078158 | 77 |
| exprReg_7336 | gi|126640115|ref|NC_009085.1| | 1079841 | 1079893 | 53 |
| exprReg_7337 | gi|126640115|ref|NC_009085.1| | 1079945 | 1079967 | 23 |
| exprReg_7358 | gi|126640115|ref|NC_009085.1| | 1082624 | 1082643 | 20 |
| exprReg_7359 | gi|126640115|ref|NC_009085.1| | 1082695 | 1082714 | 20 |
| exprReg_7360 | gi|126640115|ref|NC_009085.1| | 1082856 | 1082877 | 22 |
| exprReg_7361 | gi|126640115|ref|NC_009085.1| | 1082892 | 1082909 | 18 |
| exprReg_7367 | gi|126640115|ref|NC_009085.1| | 1083399 | 1083421 | 23 |
| exprReg_7368 | gi|126640115|ref|NC_009085.1| | 1083428 | 1083584 | 157 |
| exprReg_7369 | gi|126640115|ref|NC_009085.1| | 1083595 | 1083664 | 70 |
| exprReg_7370 | gi|126640115|ref|NC_009085.1| | 1083675 | 1083779 | 105 |
| exprReg_7371 | gi|126640115|ref|NC_009085.1| | 1083784 | 1083890 | 107 |
| exprReg_7372 | gi|126640115|ref|NC_009085.1| | 1083988 | 1084011 | 24 |
| exprReg_7373 | gi|126640115|ref|NC_009085.1| | 1084149 | 1084192 | 44 |
| exprReg_7374 | gi|126640115|ref|NC_009085.1| | 1084204 | 1084224 | 21 |
| exprReg_7375 | gi|126640115|ref|NC_009085.1| | 1084304 | 1084321 | 18 |
| exprReg_7389 | gi|126640115|ref|NC_009085.1| | 1086463 | 1086532 | 70 |
| exprReg_7390 | gi|126640115|ref|NC_009085.1| | 1086659 | 1086681 | 23 |
| exprReg_7399 | gi|126640115|ref|NC_009085.1| | 1087662 | 1087678 | 17 |
| exprReg_7400 | gi|126640115|ref|NC_009085.1| | 1087878 | 1087900 | 23 |
| exprReg_7426 | gi|126640115|ref|NC_009085.1| | 1091184 | 1091227 | 44 |
| exprReg_7429 | gi|126640115|ref|NC_009085.1| | 1091684 | 1091742 | 59 |
| exprReg_7433 | gi|126640115|ref|NC_009085.1| | 1092530 | 1092575 | 46 |
| exprReg_7434 | gi|126640115|ref|NC_009085.1| | 1092762 | 1092778 | 17 |
| exprReg_7444 | gi|126640115|ref|NC_009085.1| | 1094454 | 1094586 | 133 |
| exprReg_7449 | gi|126640115|ref|NC_009085.1| | 1095484 | 1095604 | 121 |
| exprReg_7468 | gi|126640115|ref|NC_009085.1| | 1098721 | 1098759 | 39 |
| exprReg_7477 | gi|126640115|ref|NC_009085.1| | 1100813 | 1100831 | 19 |
| exprReg_7486 | gi|126640115|ref|NC_009085.1| | 1102184 | 1102275 | 92 |
| exprReg_7495 | gi|126640115|ref|NC_009085.1| | 1103807 | 1103835 | 29 |
| exprReg_7496 | gi|126640115|ref|NC_009085.1| | 1103867 | 1103883 | 17 |
| exprReg_7512 | gi|126640115|ref|NC_009085.1| | 1107131 | 1107192 | 62 |
| exprReg_7513 | gi|126640115|ref|NC_009085.1| | 1107210 | 1107250 | 41 |
| exprReg_7530 | gi|126640115|ref|NC_009085.1| | 1109557 | 1109668 | 112 |
| exprReg_7531 | gi|126640115|ref|NC_009085.1| | 1109733 | 1109886 | 154 |
| exprReg_7532 | gi|126640115|ref|NC_009085.1| | 1109893 | 1109916 | 24 |
| exprReg_7537 | gi|126640115|ref|NC_009085.1| | 1110430 | 1110501 | 72 |
| exprReg_7549 | gi|126640115|ref|NC_009085.1| | 1112743 | 1112763 | 21 |
| exprReg_7556 | gi|126640115|ref|NC_009085.1| | 1113954 | 1113972 | 19 |
| exprReg_7557 | gi|126640115|ref|NC_009085.1| | 1114064 | 1114081 | 18 |
| exprReg_7558 | gi|126640115|ref|NC_009085.1| | 1114090 | 1114172 | 83 |
| exprReg_7559 | gi|126640115|ref|NC_009085.1| | 1114201 | 1114228 | 28 |
| exprReg_7560 | gi|126640115|ref|NC_009085.1| | 1114266 | 1114359 | 94 |
| exprReg_7561 | gi|126640115|ref|NC_009085.1| | 1114373 | 1114391 | 19 |
| exprReg_7562 | gi|126640115|ref|NC_009085.1| | 1114427 | 1114450 | 24 |
| exprReg_7563 | gi|126640115|ref|NC_009085.1| | 1114455 | 1114492 | 38 |
| exprReg_7564 | gi|126640115|ref|NC_009085.1| | 1114558 | 1114576 | 19 |
| exprReg_7565 | gi|126640115|ref|NC_009085.1| | 1114591 | 1114615 | 25 |
| exprReg_7566 | gi|126640115|ref|NC_009085.1| | 1114627 | 1114674 | 48 |
| exprReg_7567 | gi|126640115|ref|NC_009085.1| | 1114809 | 1114895 | 87 |
| exprReg_7568 | gi|126640115|ref|NC_009085.1| | 1114990 | 1115027 | 38 |
| exprReg_7569 | gi|126640115|ref|NC_009085.1| | 1115051 | 1115067 | 17 |
| exprReg_7577 | gi|126640115|ref|NC_009085.1| | 1116645 | 1116703 | 59 |
| exprReg_7578 | gi|126640115|ref|NC_009085.1| | 1116836 | 1116880 | 45 |
| exprReg_7579 | gi|126640115|ref|NC_009085.1| | 1117156 | 1117248 | 93 |
| exprReg_7595 | gi|126640115|ref|NC_009085.1| | 1119255 | 1119284 | 30 |
| exprReg_7611 | gi|126640115|ref|NC_009085.1| | 1121507 | 1121570 | 64 |
| exprReg_7616 | gi|126640115|ref|NC_009085.1| | 1122489 | 1122508 | 20 |
| exprReg_7634 | gi|126640115|ref|NC_009085.1| | 1126235 | 1126253 | 19 |
| exprReg_7635 | gi|126640115|ref|NC_009085.1| | 1126495 | 1126562 | 68 |
| exprReg_7651 | gi|126640115|ref|NC_009085.1| | 1128357 | 1128442 | 86 |
| exprReg_7667 | gi|126640115|ref|NC_009085.1| | 1131636 | 1131939 | 304 |
| exprReg_7668 | gi|126640115|ref|NC_009085.1| | 1131953 | 1132075 | 123 |
| exprReg_7669 | gi|126640115|ref|NC_009085.1| | 1132104 | 1132137 | 34 |
| exprReg_7676 | gi|126640115|ref|NC_009085.1| | 1133051 | 1133068 | 18 |
| exprReg_7694 | gi|126640115|ref|NC_009085.1| | 1135686 | 1135724 | 39 |
| exprReg_7695 | gi|126640115|ref|NC_009085.1| | 1135817 | 1135894 | 78 |
| exprReg_7699 | gi|126640115|ref|NC_009085.1| | 1136607 | 1136646 | 40 |
| exprReg_7712 | gi|126640115|ref|NC_009085.1| | 1139643 | 1139721 | 79 |
| exprReg_7713 | gi|126640115|ref|NC_009085.1| | 1139809 | 1139855 | 47 |
| exprReg_7714 | gi|126640115|ref|NC_009085.1| | 1139882 | 1139922 | 41 |
| exprReg_7726 | gi|126640115|ref|NC_009085.1| | 1140850 | 1140868 | 19 |
| exprReg_7727 | gi|126640115|ref|NC_009085.1| | 1140872 | 1141029 | 158 |
| exprReg_7728 | gi|126640115|ref|NC_009085.1| | 1141051 | 1141090 | 40 |
| exprReg_7729 | gi|126640115|ref|NC_009085.1| | 1141275 | 1141357 | 83 |
| exprReg_7739 | gi|126640115|ref|NC_009085.1| | 1142623 | 1142639 | 17 |
| exprReg_7746 | gi|126640115|ref|NC_009085.1| | 1143295 | 1143364 | 70 |
| exprReg_7747 | gi|126640115|ref|NC_009085.1| | 1143377 | 1143396 | 20 |
| exprReg_7767 | gi|126640115|ref|NC_009085.1| | 1146357 | 1146376 | 20 |
| exprReg_7768 | gi|126640115|ref|NC_009085.1| | 1146426 | 1146570 | 145 |
| exprReg_7769 | gi|126640115|ref|NC_009085.1| | 1146588 | 1146641 | 54 |
| exprReg_7770 | gi|126640115|ref|NC_009085.1| | 1146645 | 1146673 | 29 |
| exprReg_7776 | gi|126640115|ref|NC_009085.1| | 1147416 | 1147460 | 45 |
| exprReg_7777 | gi|126640115|ref|NC_009085.1| | 1147475 | 1147522 | 48 |
| exprReg_7819 | gi|126640115|ref|NC_009085.1| | 1153414 | 1153431 | 18 |
| exprReg_7820 | gi|126640115|ref|NC_009085.1| | 1153453 | 1153470 | 18 |
| exprReg_7822 | gi|126640115|ref|NC_009085.1| | 1153789 | 1153807 | 19 |
| exprReg_7823 | gi|126640115|ref|NC_009085.1| | 1154001 | 1154075 | 75 |
| exprReg_7824 | gi|126640115|ref|NC_009085.1| | 1154128 | 1154222 | 95 |
| exprReg_7831 | gi|126640115|ref|NC_009085.1| | 1155106 | 1155145 | 40 |
| exprReg_7832 | gi|126640115|ref|NC_009085.1| | 1155197 | 1155226 | 30 |
| exprReg_7833 | gi|126640115|ref|NC_009085.1| | 1155245 | 1155303 | 59 |
| exprReg_7834 | gi|126640115|ref|NC_009085.1| | 1155397 | 1155491 | 95 |
| exprReg_7857 | gi|126640115|ref|NC_009085.1| | 1158653 | 1158672 | 20 |
| exprReg_7858 | gi|126640115|ref|NC_009085.1| | 1158699 | 1158729 | 31 |
| exprReg_7865 | gi|126640115|ref|NC_009085.1| | 1159901 | 1159930 | 30 |
| exprReg_7866 | gi|126640115|ref|NC_009085.1| | 1159934 | 1159990 | 57 |
| exprReg_7879 | gi|126640115|ref|NC_009085.1| | 1162171 | 1162203 | 33 |
| exprReg_7900 | gi|126640115|ref|NC_009085.1| | 1164074 | 1164091 | 18 |
| exprReg_7901 | gi|126640115|ref|NC_009085.1| | 1164102 | 1164118 | 17 |
| exprReg_7902 | gi|126640115|ref|NC_009085.1| | 1164159 | 1164285 | 127 |
| exprReg_7903 | gi|126640115|ref|NC_009085.1| | 1164376 | 1164394 | 19 |
| exprReg_7904 | gi|126640115|ref|NC_009085.1| | 1164448 | 1164541 | 94 |
| exprReg_7912 | gi|126640115|ref|NC_009085.1| | 1165972 | 1166168 | 197 |
| exprReg_7917 | gi|126640115|ref|NC_009085.1| | 1166682 | 1166699 | 18 |
| exprReg_7920 | gi|126640115|ref|NC_009085.1| | 1167200 | 1167218 | 19 |
| exprReg_7921 | gi|126640115|ref|NC_009085.1| | 1167280 | 1167298 | 19 |
| exprReg_7922 | gi|126640115|ref|NC_009085.1| | 1167308 | 1167424 | 117 |
| exprReg_7923 | gi|126640115|ref|NC_009085.1| | 1167542 | 1167561 | 20 |
| exprReg_7924 | gi|126640115|ref|NC_009085.1| | 1167701 | 1167828 | 128 |
| exprReg_7938 | gi|126640115|ref|NC_009085.1| | 1169503 | 1169535 | 33 |
| exprReg_7945 | gi|126640115|ref|NC_009085.1| | 1170504 | 1170615 | 112 |
| exprReg_7976 | gi|126640115|ref|NC_009085.1| | 1176298 | 1176314 | 17 |
| exprReg_7996 | gi|126640115|ref|NC_009085.1| | 1179467 | 1179535 | 69 |
| exprReg_7997 | gi|126640115|ref|NC_009085.1| | 1179632 | 1179702 | 71 |
| exprReg_8001 | gi|126640115|ref|NC_009085.1| | 1180262 | 1180333 | 72 |
| exprReg_8002 | gi|126640115|ref|NC_009085.1| | 1180335 | 1180386 | 52 |
| exprReg_8009 | gi|126640115|ref|NC_009085.1| | 1181729 | 1181769 | 41 |
| exprReg_8014 | gi|126640115|ref|NC_009085.1| | 1182460 | 1182529 | 70 |
| exprReg_8021 | gi|126640115|ref|NC_009085.1| | 1183180 | 1183231 | 52 |
| exprReg_8027 | gi|126640115|ref|NC_009085.1| | 1183983 | 1184110 | 128 |
| exprReg_8028 | gi|126640115|ref|NC_009085.1| | 1184369 | 1184412 | 44 |
| exprReg_8041 | gi|126640115|ref|NC_009085.1| | 1186713 | 1186765 | 53 |
| exprReg_8042 | gi|126640115|ref|NC_009085.1| | 1186800 | 1186871 | 72 |
| exprReg_8043 | gi|126640115|ref|NC_009085.1| | 1186877 | 1186943 | 67 |
| exprReg_8044 | gi|126640115|ref|NC_009085.1| | 1187051 | 1187067 | 17 |
| exprReg_8052 | gi|126640115|ref|NC_009085.1| | 1188512 | 1188604 | 93 |
| exprReg_8053 | gi|126640115|ref|NC_009085.1| | 1188611 | 1188627 | 17 |
| exprReg_8071 | gi|126640115|ref|NC_009085.1| | 1191258 | 1191277 | 20 |
| exprReg_8112 | gi|126640115|ref|NC_009085.1| | 1197798 | 1197814 | 17 |
| exprReg_8122 | gi|126640115|ref|NC_009085.1| | 1199132 | 1199153 | 22 |
| exprReg_8129 | gi|126640115|ref|NC_009085.1| | 1199807 | 1199895 | 89 |
| exprReg_8130 | gi|126640115|ref|NC_009085.1| | 1199971 | 1200002 | 32 |
| exprReg_8131 | gi|126640115|ref|NC_009085.1| | 1200046 | 1200070 | 25 |
| exprReg_8132 | gi|126640115|ref|NC_009085.1| | 1200124 | 1200142 | 19 |
| exprReg_8133 | gi|126640115|ref|NC_009085.1| | 1200150 | 1200166 | 17 |
| exprReg_8134 | gi|126640115|ref|NC_009085.1| | 1200264 | 1200367 | 104 |
| exprReg_8135 | gi|126640115|ref|NC_009085.1| | 1200377 | 1200400 | 24 |
| exprReg_8136 | gi|126640115|ref|NC_009085.1| | 1200447 | 1200679 | 233 |
| exprReg_8137 | gi|126640115|ref|NC_009085.1| | 1200698 | 1200715 | 18 |
| exprReg_8144 | gi|126640115|ref|NC_009085.1| | 1201649 | 1201668 | 20 |
| exprReg_8160 | gi|126640115|ref|NC_009085.1| | 1203674 | 1203692 | 19 |
| exprReg_8161 | gi|126640115|ref|NC_009085.1| | 1203701 | 1203755 | 55 |
| exprReg_8171 | gi|126640115|ref|NC_009085.1| | 1204909 | 1204936 | 28 |
| exprReg_8179 | gi|126640115|ref|NC_009085.1| | 1206131 | 1206151 | 21 |
| exprReg_8180 | gi|126640115|ref|NC_009085.1| | 1206199 | 1206224 | 26 |
| exprReg_8183 | gi|126640115|ref|NC_009085.1| | 1206609 | 1206627 | 19 |
| exprReg_8194 | gi|126640115|ref|NC_009085.1| | 1208271 | 1208289 | 19 |
| exprReg_8195 | gi|126640115|ref|NC_009085.1| | 1208312 | 1208378 | 67 |
| exprReg_8196 | gi|126640115|ref|NC_009085.1| | 1208396 | 1208440 | 45 |
| exprReg_8204 | gi|126640115|ref|NC_009085.1| | 1209841 | 1209885 | 45 |
| exprReg_8209 | gi|126640115|ref|NC_009085.1| | 1210500 | 1210763 | 264 |
| exprReg_8215 | gi|126640115|ref|NC_009085.1| | 1211337 | 1211353 | 17 |
| exprReg_8216 | gi|126640115|ref|NC_009085.1| | 1211355 | 1211436 | 82 |
| exprReg_8217 | gi|126640115|ref|NC_009085.1| | 1211469 | 1211496 | 28 |
| exprReg_8218 | gi|126640115|ref|NC_009085.1| | 1211500 | 1211521 | 22 |
| exprReg_8251 | gi|126640115|ref|NC_009085.1| | 1215159 | 1215176 | 18 |
| exprReg_8252 | gi|126640115|ref|NC_009085.1| | 1215181 | 1215355 | 175 |
| exprReg_8253 | gi|126640115|ref|NC_009085.1| | 1215425 | 1215442 | 18 |
| exprReg_8254 | gi|126640115|ref|NC_009085.1| | 1215456 | 1215497 | 42 |
| exprReg_8263 | gi|126640115|ref|NC_009085.1| | 1216967 | 1216983 | 17 |
| exprReg_8277 | gi|126640115|ref|NC_009085.1| | 1220415 | 1220431 | 17 |
| exprReg_8292 | gi|126640115|ref|NC_009085.1| | 1222212 | 1222229 | 18 |
| exprReg_8307 | gi|126640115|ref|NC_009085.1| | 1224772 | 1224844 | 73 |
| exprReg_8345 | gi|126640115|ref|NC_009085.1| | 1229436 | 1229462 | 27 |
| exprReg_8346 | gi|126640115|ref|NC_009085.1| | 1229518 | 1229537 | 20 |
| exprReg_8361 | gi|126640115|ref|NC_009085.1| | 1231394 | 1231428 | 35 |
| exprReg_8362 | gi|126640115|ref|NC_009085.1| | 1231511 | 1231586 | 76 |
| exprReg_8372 | gi|126640115|ref|NC_009085.1| | 1233399 | 1233459 | 61 |
| exprReg_8373 | gi|126640115|ref|NC_009085.1| | 1233505 | 1233538 | 34 |
| exprReg_8379 | gi|126640115|ref|NC_009085.1| | 1234219 | 1234235 | 17 |
| exprReg_8380 | gi|126640115|ref|NC_009085.1| | 1234256 | 1234289 | 34 |
| exprReg_8381 | gi|126640115|ref|NC_009085.1| | 1234350 | 1234374 | 25 |
| exprReg_8382 | gi|126640115|ref|NC_009085.1| | 1234413 | 1234481 | 69 |
| exprReg_8383 | gi|126640115|ref|NC_009085.1| | 1234551 | 1234789 | 239 |
| exprReg_8384 | gi|126640115|ref|NC_009085.1| | 1234801 | 1234899 | 99 |
| exprReg_8385 | gi|126640115|ref|NC_009085.1| | 1234927 | 1235023 | 97 |
| exprReg_8400 | gi|126640115|ref|NC_009085.1| | 1237227 | 1237317 | 91 |
| exprReg_8401 | gi|126640115|ref|NC_009085.1| | 1237353 | 1237413 | 61 |
| exprReg_8402 | gi|126640115|ref|NC_009085.1| | 1237578 | 1237666 | 89 |
| exprReg_8403 | gi|126640115|ref|NC_009085.1| | 1237698 | 1237724 | 27 |
| exprReg_8404 | gi|126640115|ref|NC_009085.1| | 1238057 | 1238115 | 59 |
| exprReg_8405 | gi|126640115|ref|NC_009085.1| | 1238158 | 1238179 | 22 |
| exprReg_8406 | gi|126640115|ref|NC_009085.1| | 1238196 | 1238217 | 22 |
| exprReg_8407 | gi|126640115|ref|NC_009085.1| | 1238328 | 1238374 | 47 |
| exprReg_8408 | gi|126640115|ref|NC_009085.1| | 1238495 | 1238515 | 21 |
| exprReg_8409 | gi|126640115|ref|NC_009085.1| | 1238578 | 1238600 | 23 |
| exprReg_8410 | gi|126640115|ref|NC_009085.1| | 1238619 | 1238641 | 23 |
| exprReg_8411 | gi|126640115|ref|NC_009085.1| | 1238660 | 1238746 | 87 |
| exprReg_8412 | gi|126640115|ref|NC_009085.1| | 1238796 | 1238891 | 96 |
| exprReg_8413 | gi|126640115|ref|NC_009085.1| | 1238893 | 1239025 | 133 |
| exprReg_8414 | gi|126640115|ref|NC_009085.1| | 1239094 | 1239212 | 119 |
| exprReg_8422 | gi|126640115|ref|NC_009085.1| | 1240355 | 1240574 | 220 |
| exprReg_8423 | gi|126640115|ref|NC_009085.1| | 1240649 | 1240668 | 20 |
| exprReg_8424 | gi|126640115|ref|NC_009085.1| | 1240693 | 1240712 | 20 |
| exprReg_8425 | gi|126640115|ref|NC_009085.1| | 1240807 | 1241120 | 314 |
| exprReg_8506 | gi|126640115|ref|NC_009085.1| | 1250838 | 1250854 | 17 |
| exprReg_8507 | gi|126640115|ref|NC_009085.1| | 1251009 | 1251147 | 139 |
| exprReg_8508 | gi|126640115|ref|NC_009085.1| | 1251216 | 1251285 | 70 |
| exprReg_8509 | gi|126640115|ref|NC_009085.1| | 1251292 | 1251310 | 19 |
| exprReg_8510 | gi|126640115|ref|NC_009085.1| | 1251313 | 1251373 | 61 |
| exprReg_8511 | gi|126640115|ref|NC_009085.1| | 1251395 | 1251422 | 28 |
| exprReg_8512 | gi|126640115|ref|NC_009085.1| | 1251438 | 1251456 | 19 |
| exprReg_8513 | gi|126640115|ref|NC_009085.1| | 1251713 | 1251730 | 18 |
| exprReg_8514 | gi|126640115|ref|NC_009085.1| | 1251925 | 1251945 | 21 |
| exprReg_8526 | gi|126640115|ref|NC_009085.1| | 1253588 | 1253607 | 20 |
| exprReg_8527 | gi|126640115|ref|NC_009085.1| | 1253611 | 1253635 | 25 |
| exprReg_8528 | gi|126640115|ref|NC_009085.1| | 1253639 | 1253655 | 17 |
| exprReg_8529 | gi|126640115|ref|NC_009085.1| | 1253662 | 1253678 | 17 |
| exprReg_8530 | gi|126640115|ref|NC_009085.1| | 1253690 | 1253718 | 29 |
| exprReg_8531 | gi|126640115|ref|NC_009085.1| | 1253725 | 1253792 | 68 |
| exprReg_8532 | gi|126640115|ref|NC_009085.1| | 1253799 | 1253817 | 19 |
| exprReg_8537 | gi|126640115|ref|NC_009085.1| | 1254719 | 1254741 | 23 |
| exprReg_8538 | gi|126640115|ref|NC_009085.1| | 1254743 | 1254786 | 44 |
| exprReg_8539 | gi|126640115|ref|NC_009085.1| | 1254881 | 1255043 | 163 |
| exprReg_8540 | gi|126640115|ref|NC_009085.1| | 1255070 | 1255113 | 44 |
| exprReg_8546 | gi|126640115|ref|NC_009085.1| | 1256410 | 1256433 | 24 |
| exprReg_8549 | gi|126640115|ref|NC_009085.1| | 1257092 | 1257110 | 19 |
| exprReg_8550 | gi|126640115|ref|NC_009085.1| | 1257371 | 1257387 | 17 |
| exprReg_8551 | gi|126640115|ref|NC_009085.1| | 1257447 | 1257472 | 26 |
| exprReg_8552 | gi|126640115|ref|NC_009085.1| | 1257493 | 1257521 | 29 |
| exprReg_8553 | gi|126640115|ref|NC_009085.1| | 1257544 | 1257648 | 105 |
| exprReg_8554 | gi|126640115|ref|NC_009085.1| | 1257652 | 1257743 | 92 |
| exprReg_8555 | gi|126640115|ref|NC_009085.1| | 1257772 | 1257858 | 87 |
| exprReg_8570 | gi|126640115|ref|NC_009085.1| | 1259788 | 1259832 | 45 |
| exprReg_8571 | gi|126640115|ref|NC_009085.1| | 1259845 | 1259861 | 17 |
| exprReg_8572 | gi|126640115|ref|NC_009085.1| | 1259905 | 1259979 | 75 |
| exprReg_8573 | gi|126640115|ref|NC_009085.1| | 1259990 | 1260045 | 56 |
| exprReg_8578 | gi|126640115|ref|NC_009085.1| | 1260882 | 1260932 | 51 |
| exprReg_8579 | gi|126640115|ref|NC_009085.1| | 1261024 | 1261071 | 48 |
| exprReg_8580 | gi|126640115|ref|NC_009085.1| | 1261205 | 1261225 | 21 |
| exprReg_8581 | gi|126640115|ref|NC_009085.1| | 1261387 | 1261414 | 28 |
| exprReg_8582 | gi|126640115|ref|NC_009085.1| | 1261576 | 1261621 | 46 |
| exprReg_8583 | gi|126640115|ref|NC_009085.1| | 1261680 | 1261901 | 222 |
| exprReg_8584 | gi|126640115|ref|NC_009085.1| | 1262025 | 1262055 | 31 |
| exprReg_8586 | gi|126640115|ref|NC_009085.1| | 1262750 | 1262777 | 28 |
| exprReg_8587 | gi|126640115|ref|NC_009085.1| | 1262816 | 1262840 | 25 |
| exprReg_8588 | gi|126640115|ref|NC_009085.1| | 1262872 | 1262910 | 39 |
| exprReg_8589 | gi|126640115|ref|NC_009085.1| | 1262954 | 1263165 | 212 |
| exprReg_8590 | gi|126640115|ref|NC_009085.1| | 1263280 | 1263310 | 31 |
| exprReg_8591 | gi|126640115|ref|NC_009085.1| | 1263487 | 1263556 | 70 |
| exprReg_8595 | gi|126640115|ref|NC_009085.1| | 1263965 | 1264062 | 98 |
| exprReg_8608 | gi|126640115|ref|NC_009085.1| | 1265704 | 1265727 | 24 |
| exprReg_8637 | gi|126640115|ref|NC_009085.1| | 1270246 | 1270266 | 21 |
| exprReg_8638 | gi|126640115|ref|NC_009085.1| | 1270330 | 1270346 | 17 |
| exprReg_8644 | gi|126640115|ref|NC_009085.1| | 1270988 | 1271004 | 17 |
| exprReg_8645 | gi|126640115|ref|NC_009085.1| | 1271007 | 1271024 | 18 |
| exprReg_8662 | gi|126640115|ref|NC_009085.1| | 1273425 | 1273462 | 38 |
| exprReg_8665 | gi|126640115|ref|NC_009085.1| | 1274059 | 1274077 | 19 |
| exprReg_8679 | gi|126640115|ref|NC_009085.1| | 1275882 | 1275899 | 18 |
| exprReg_8680 | gi|126640115|ref|NC_009085.1| | 1276013 | 1276067 | 55 |
| exprReg_8681 | gi|126640115|ref|NC_009085.1| | 1276217 | 1276267 | 51 |
| exprReg_8697 | gi|126640115|ref|NC_009085.1| | 1277663 | 1277706 | 44 |
| exprReg_8698 | gi|126640115|ref|NC_009085.1| | 1278045 | 1278067 | 23 |
| exprReg_8699 | gi|126640115|ref|NC_009085.1| | 1278164 | 1278186 | 23 |
| exprReg_8700 | gi|126640115|ref|NC_009085.1| | 1278274 | 1278340 | 67 |
| exprReg_8701 | gi|126640115|ref|NC_009085.1| | 1278957 | 1278976 | 20 |
| exprReg_8702 | gi|126640115|ref|NC_009085.1| | 1279146 | 1279280 | 135 |
| exprReg_8712 | gi|126640115|ref|NC_009085.1| | 1280957 | 1280996 | 40 |
| exprReg_8713 | gi|126640115|ref|NC_009085.1| | 1281033 | 1281232 | 200 |
| exprReg_8714 | gi|126640115|ref|NC_009085.1| | 1281242 | 1281270 | 29 |
| exprReg_8727 | gi|126640115|ref|NC_009085.1| | 1283046 | 1283121 | 76 |
| exprReg_8736 | gi|126640115|ref|NC_009085.1| | 1284620 | 1284646 | 27 |
| exprReg_8737 | gi|126640115|ref|NC_009085.1| | 1284667 | 1284688 | 22 |
| exprReg_8747 | gi|126640115|ref|NC_009085.1| | 1286206 | 1286239 | 34 |
| exprReg_8748 | gi|126640115|ref|NC_009085.1| | 1286284 | 1286330 | 47 |
| exprReg_8762 | gi|126640115|ref|NC_009085.1| | 1288964 | 1289000 | 37 |
| exprReg_8763 | gi|126640115|ref|NC_009085.1| | 1289042 | 1289074 | 33 |
| exprReg_8774 | gi|126640115|ref|NC_009085.1| | 1290677 | 1290693 | 17 |
| exprReg_8775 | gi|126640115|ref|NC_009085.1| | 1290789 | 1290838 | 50 |
| exprReg_8776 | gi|126640115|ref|NC_009085.1| | 1290847 | 1290897 | 51 |
| exprReg_8790 | gi|126640115|ref|NC_009085.1| | 1293135 | 1293207 | 73 |
| exprReg_8819 | gi|126640115|ref|NC_009085.1| | 1298590 | 1298633 | 44 |
| exprReg_8820 | gi|126640115|ref|NC_009085.1| | 1298660 | 1298730 | 71 |
| exprReg_8821 | gi|126640115|ref|NC_009085.1| | 1298733 | 1298819 | 87 |
| exprReg_8828 | gi|126640115|ref|NC_009085.1| | 1300113 | 1300162 | 50 |
| exprReg_8829 | gi|126640115|ref|NC_009085.1| | 1300202 | 1300232 | 31 |
| exprReg_8849 | gi|126640115|ref|NC_009085.1| | 1302725 | 1302746 | 22 |
| exprReg_8850 | gi|126640115|ref|NC_009085.1| | 1302748 | 1302774 | 27 |
| exprReg_8856 | gi|126640115|ref|NC_009085.1| | 1303192 | 1303239 | 48 |
| exprReg_8857 | gi|126640115|ref|NC_009085.1| | 1303280 | 1303312 | 33 |
| exprReg_8867 | gi|126640115|ref|NC_009085.1| | 1304895 | 1304950 | 56 |
| exprReg_8875 | gi|126640115|ref|NC_009085.1| | 1306505 | 1306550 | 46 |
| exprReg_8885 | gi|126640115|ref|NC_009085.1| | 1307941 | 1308036 | 96 |
| exprReg_8889 | gi|126640115|ref|NC_009085.1| | 1309256 | 1309272 | 17 |
| exprReg_8903 | gi|126640115|ref|NC_009085.1| | 1310937 | 1310960 | 24 |
| exprReg_8912 | gi|126640115|ref|NC_009085.1| | 1311832 | 1311848 | 17 |
| exprReg_8913 | gi|126640115|ref|NC_009085.1| | 1311892 | 1311937 | 46 |
| exprReg_8934 | gi|126640115|ref|NC_009085.1| | 1316343 | 1316445 | 103 |
| exprReg_8942 | gi|126640115|ref|NC_009085.1| | 1317889 | 1317928 | 40 |
| exprReg_8947 | gi|126640115|ref|NC_009085.1| | 1318878 | 1318913 | 36 |
| exprReg_8951 | gi|126640115|ref|NC_009085.1| | 1319782 | 1319822 | 41 |
| exprReg_8952 | gi|126640115|ref|NC_009085.1| | 1319868 | 1319886 | 19 |
| exprReg_8953 | gi|126640115|ref|NC_009085.1| | 1320002 | 1320055 | 54 |
| exprReg_8954 | gi|126640115|ref|NC_009085.1| | 1320360 | 1320398 | 39 |
| exprReg_8955 | gi|126640115|ref|NC_009085.1| | 1320483 | 1320560 | 78 |
| exprReg_8962 | gi|126640115|ref|NC_009085.1| | 1321718 | 1321749 | 32 |
| exprReg_8963 | gi|126640115|ref|NC_009085.1| | 1321762 | 1321784 | 23 |
| exprReg_8964 | gi|126640115|ref|NC_009085.1| | 1321787 | 1321853 | 67 |
| exprReg_8965 | gi|126640115|ref|NC_009085.1| | 1322125 | 1322264 | 140 |
| exprReg_8966 | gi|126640115|ref|NC_009085.1| | 1322269 | 1322286 | 18 |
| exprReg_8967 | gi|126640115|ref|NC_009085.1| | 1322315 | 1322336 | 22 |
| exprReg_8968 | gi|126640115|ref|NC_009085.1| | 1322348 | 1322417 | 70 |
| exprReg_8969 | gi|126640115|ref|NC_009085.1| | 1322477 | 1322494 | 18 |
| exprReg_8974 | gi|126640115|ref|NC_009085.1| | 1323387 | 1323479 | 93 |
| exprReg_8991 | gi|126640115|ref|NC_009085.1| | 1325759 | 1325788 | 30 |
| exprReg_8992 | gi|126640115|ref|NC_009085.1| | 1325826 | 1325905 | 80 |
| exprReg_8993 | gi|126640115|ref|NC_009085.1| | 1325985 | 1326057 | 73 |
| exprReg_8994 | gi|126640115|ref|NC_009085.1| | 1326114 | 1326197 | 84 |
| exprReg_8995 | gi|126640115|ref|NC_009085.1| | 1326209 | 1326427 | 219 |
| exprReg_8996 | gi|126640115|ref|NC_009085.1| | 1326502 | 1326562 | 61 |
| exprReg_9010 | gi|126640115|ref|NC_009085.1| | 1329844 | 1329889 | 46 |
| exprReg_9023 | gi|126640115|ref|NC_009085.1| | 1331257 | 1331424 | 168 |
| exprReg_9024 | gi|126640115|ref|NC_009085.1| | 1331476 | 1331497 | 22 |
| exprReg_9039 | gi|126640115|ref|NC_009085.1| | 1332861 | 1332937 | 77 |
| exprReg_9040 | gi|126640115|ref|NC_009085.1| | 1333006 | 1333071 | 66 |
| exprReg_9041 | gi|126640115|ref|NC_009085.1| | 1333082 | 1333112 | 31 |
| exprReg_9042 | gi|126640115|ref|NC_009085.1| | 1333133 | 1333307 | 175 |
| exprReg_9043 | gi|126640115|ref|NC_009085.1| | 1333338 | 1333354 | 17 |
| exprReg_9044 | gi|126640115|ref|NC_009085.1| | 1333402 | 1333422 | 21 |
| exprReg_9045 | gi|126640115|ref|NC_009085.1| | 1333458 | 1333516 | 59 |
| exprReg_9046 | gi|126640115|ref|NC_009085.1| | 1333560 | 1333742 | 183 |
| exprReg_9047 | gi|126640115|ref|NC_009085.1| | 1333751 | 1333868 | 118 |
| exprReg_9048 | gi|126640115|ref|NC_009085.1| | 1333879 | 1334178 | 300 |
| exprReg_9049 | gi|126640115|ref|NC_009085.1| | 1334249 | 1334395 | 147 |
| exprReg_9050 | gi|126640115|ref|NC_009085.1| | 1334492 | 1334680 | 189 |
| exprReg_9051 | gi|126640115|ref|NC_009085.1| | 1334683 | 1334774 | 92 |
| exprReg_9052 | gi|126640115|ref|NC_009085.1| | 1334780 | 1334816 | 37 |
| exprReg_9053 | gi|126640115|ref|NC_009085.1| | 1335294 | 1335312 | 19 |
| exprReg_9054 | gi|126640115|ref|NC_009085.1| | 1335464 | 1335544 | 81 |
| exprReg_9055 | gi|126640115|ref|NC_009085.1| | 1335636 | 1335704 | 69 |
| exprReg_9056 | gi|126640115|ref|NC_009085.1| | 1335764 | 1335824 | 61 |
| exprReg_9057 | gi|126640115|ref|NC_009085.1| | 1335844 | 1335890 | 47 |
| exprReg_9058 | gi|126640115|ref|NC_009085.1| | 1335933 | 1335961 | 29 |
| exprReg_9059 | gi|126640115|ref|NC_009085.1| | 1336043 | 1336072 | 30 |
| exprReg_9060 | gi|126640115|ref|NC_009085.1| | 1336411 | 1336446 | 36 |
| exprReg_9070 | gi|126640115|ref|NC_009085.1| | 1338293 | 1338311 | 19 |
| exprReg_9071 | gi|126640115|ref|NC_009085.1| | 1338442 | 1338572 | 131 |
| exprReg_9072 | gi|126640115|ref|NC_009085.1| | 1338630 | 1338735 | 106 |
| exprReg_9073 | gi|126640115|ref|NC_009085.1| | 1338747 | 1338772 | 26 |
| exprReg_9074 | gi|126640115|ref|NC_009085.1| | 1338790 | 1338947 | 158 |
| exprReg_9075 | gi|126640115|ref|NC_009085.1| | 1338974 | 1339054 | 81 |
| exprReg_9076 | gi|126640115|ref|NC_009085.1| | 1339062 | 1339078 | 17 |
| exprReg_9082 | gi|126640115|ref|NC_009085.1| | 1340143 | 1340160 | 18 |
| exprReg_9083 | gi|126640115|ref|NC_009085.1| | 1340328 | 1340475 | 148 |
| exprReg_9084 | gi|126640115|ref|NC_009085.1| | 1340729 | 1340759 | 31 |
| exprReg_9085 | gi|126640115|ref|NC_009085.1| | 1340806 | 1340872 | 67 |
| exprReg_9086 | gi|126640115|ref|NC_009085.1| | 1340932 | 1341027 | 96 |
| exprReg_9087 | gi|126640115|ref|NC_009085.1| | 1341075 | 1341212 | 138 |
| exprReg_9088 | gi|126640115|ref|NC_009085.1| | 1341216 | 1341277 | 62 |
| exprReg_9089 | gi|126640115|ref|NC_009085.1| | 1341340 | 1341392 | 53 |
| exprReg_9090 | gi|126640115|ref|NC_009085.1| | 1341451 | 1341512 | 62 |
| exprReg_9091 | gi|126640115|ref|NC_009085.1| | 1341611 | 1341666 | 56 |
| exprReg_9092 | gi|126640115|ref|NC_009085.1| | 1341721 | 1341749 | 29 |
| exprReg_9093 | gi|126640115|ref|NC_009085.1| | 1341889 | 1341971 | 83 |
| exprReg_9094 | gi|126640115|ref|NC_009085.1| | 1342076 | 1342101 | 26 |
| exprReg_9095 | gi|126640115|ref|NC_009085.1| | 1342129 | 1342185 | 57 |
| exprReg_9096 | gi|126640115|ref|NC_009085.1| | 1342260 | 1342301 | 42 |
| exprReg_9097 | gi|126640115|ref|NC_009085.1| | 1342304 | 1342481 | 178 |
| exprReg_9098 | gi|126640115|ref|NC_009085.1| | 1342669 | 1342691 | 23 |
| exprReg_9101 | gi|126640115|ref|NC_009085.1| | 1343355 | 1343464 | 110 |
| exprReg_9102 | gi|126640115|ref|NC_009085.1| | 1343593 | 1343630 | 38 |
| exprReg_9103 | gi|126640115|ref|NC_009085.1| | 1343662 | 1343717 | 56 |
| exprReg_9104 | gi|126640115|ref|NC_009085.1| | 1343824 | 1343879 | 56 |
| exprReg_9105 | gi|126640115|ref|NC_009085.1| | 1343964 | 1343985 | 22 |
| exprReg_9106 | gi|126640115|ref|NC_009085.1| | 1344108 | 1344149 | 42 |
| exprReg_9107 | gi|126640115|ref|NC_009085.1| | 1344176 | 1344193 | 18 |
| exprReg_9108 | gi|126640115|ref|NC_009085.1| | 1344262 | 1344334 | 73 |
| exprReg_9127 | gi|126640115|ref|NC_009085.1| | 1347637 | 1347714 | 78 |
| exprReg_9138 | gi|126640115|ref|NC_009085.1| | 1349773 | 1349918 | 146 |
| exprReg_9139 | gi|126640115|ref|NC_009085.1| | 1349954 | 1349975 | 22 |
| exprReg_9140 | gi|126640115|ref|NC_009085.1| | 1349977 | 1349994 | 18 |
| exprReg_9141 | gi|126640115|ref|NC_009085.1| | 1350342 | 1350468 | 127 |
| exprReg_9142 | gi|126640115|ref|NC_009085.1| | 1350487 | 1350590 | 104 |
| exprReg_9159 | gi|126640115|ref|NC_009085.1| | 1353951 | 1353988 | 38 |
| exprReg_9162 | gi|126640115|ref|NC_009085.1| | 1354495 | 1354513 | 19 |
| exprReg_9172 | gi|126640115|ref|NC_009085.1| | 1356429 | 1356495 | 67 |
| exprReg_9175 | gi|126640115|ref|NC_009085.1| | 1356973 | 1356989 | 17 |
| exprReg_9176 | gi|126640115|ref|NC_009085.1| | 1357006 | 1357056 | 51 |
| exprReg_9177 | gi|126640115|ref|NC_009085.1| | 1357095 | 1357162 | 68 |
| exprReg_9178 | gi|126640115|ref|NC_009085.1| | 1357189 | 1357213 | 25 |
| exprReg_9179 | gi|126640115|ref|NC_009085.1| | 1357272 | 1357290 | 19 |
| exprReg_9180 | gi|126640115|ref|NC_009085.1| | 1357311 | 1357331 | 21 |
| exprReg_9202 | gi|126640115|ref|NC_009085.1| | 1361307 | 1361355 | 49 |
| exprReg_9207 | gi|126640115|ref|NC_009085.1| | 1362131 | 1362172 | 42 |
| exprReg_9208 | gi|126640115|ref|NC_009085.1| | 1362188 | 1362215 | 28 |
| exprReg_9216 | gi|126640115|ref|NC_009085.1| | 1364012 | 1364028 | 17 |
| exprReg_9217 | gi|126640115|ref|NC_009085.1| | 1364137 | 1364202 | 66 |
| exprReg_9218 | gi|126640115|ref|NC_009085.1| | 1364259 | 1364281 | 23 |
| exprReg_9219 | gi|126640115|ref|NC_009085.1| | 1364362 | 1364669 | 308 |
| exprReg_9220 | gi|126640115|ref|NC_009085.1| | 1364712 | 1364769 | 58 |
| exprReg_9221 | gi|126640115|ref|NC_009085.1| | 1364798 | 1364901 | 104 |
| exprReg_9222 | gi|126640115|ref|NC_009085.1| | 1364981 | 1365031 | 51 |
| exprReg_9225 | gi|126640115|ref|NC_009085.1| | 1365553 | 1365640 | 88 |
| exprReg_9241 | gi|126640115|ref|NC_009085.1| | 1367688 | 1368004 | 317 |
| exprReg_9242 | gi|126640115|ref|NC_009085.1| | 1368046 | 1368095 | 50 |
| exprReg_9247 | gi|126640115|ref|NC_009085.1| | 1368706 | 1368936 | 231 |
| exprReg_9254 | gi|126640115|ref|NC_009085.1| | 1369647 | 1369684 | 38 |
| exprReg_9255 | gi|126640115|ref|NC_009085.1| | 1369692 | 1369766 | 75 |
| exprReg_9256 | gi|126640115|ref|NC_009085.1| | 1369770 | 1369868 | 99 |
| exprReg_9257 | gi|126640115|ref|NC_009085.1| | 1369951 | 1370043 | 93 |
| exprReg_9258 | gi|126640115|ref|NC_009085.1| | 1370051 | 1370068 | 18 |
| exprReg_9259 | gi|126640115|ref|NC_009085.1| | 1370098 | 1370340 | 243 |
| exprReg_9260 | gi|126640115|ref|NC_009085.1| | 1370345 | 1370377 | 33 |
| exprReg_9274 | gi|126640115|ref|NC_009085.1| | 1372227 | 1372244 | 18 |
| exprReg_9275 | gi|126640115|ref|NC_009085.1| | 1372338 | 1372628 | 291 |
| exprReg_9276 | gi|126640115|ref|NC_009085.1| | 1372640 | 1372840 | 201 |
| exprReg_9277 | gi|126640115|ref|NC_009085.1| | 1372870 | 1373040 | 171 |
| exprReg_9278 | gi|126640115|ref|NC_009085.1| | 1373042 | 1373232 | 191 |
| exprReg_9279 | gi|126640115|ref|NC_009085.1| | 1373237 | 1373266 | 30 |
| exprReg_9280 | gi|126640115|ref|NC_009085.1| | 1373467 | 1373485 | 19 |
| exprReg_9281 | gi|126640115|ref|NC_009085.1| | 1373489 | 1373506 | 18 |
| exprReg_9282 | gi|126640115|ref|NC_009085.1| | 1373539 | 1373573 | 35 |
| exprReg_9283 | gi|126640115|ref|NC_009085.1| | 1373675 | 1373740 | 66 |
| exprReg_9284 | gi|126640115|ref|NC_009085.1| | 1373766 | 1373849 | 84 |
| exprReg_9285 | gi|126640115|ref|NC_009085.1| | 1373918 | 1373935 | 18 |
| exprReg_9286 | gi|126640115|ref|NC_009085.1| | 1373950 | 1374026 | 77 |
| exprReg_9287 | gi|126640115|ref|NC_009085.1| | 1374035 | 1374063 | 29 |
| exprReg_9299 | gi|126640115|ref|NC_009085.1| | 1375442 | 1375485 | 44 |
| exprReg_9300 | gi|126640115|ref|NC_009085.1| | 1375529 | 1375550 | 22 |
| exprReg_9301 | gi|126640115|ref|NC_009085.1| | 1375566 | 1375607 | 42 |
| exprReg_9326 | gi|126640115|ref|NC_009085.1| | 1378244 | 1378262 | 19 |
| exprReg_9327 | gi|126640115|ref|NC_009085.1| | 1378264 | 1378284 | 21 |
| exprReg_9328 | gi|126640115|ref|NC_009085.1| | 1378338 | 1378491 | 154 |
| exprReg_9329 | gi|126640115|ref|NC_009085.1| | 1378554 | 1378646 | 93 |
| exprReg_9340 | gi|126640115|ref|NC_009085.1| | 1379805 | 1379830 | 26 |
| exprReg_9341 | gi|126640115|ref|NC_009085.1| | 1379875 | 1379892 | 18 |
| exprReg_9342 | gi|126640115|ref|NC_009085.1| | 1379929 | 1380018 | 90 |
| exprReg_9350 | gi|126640115|ref|NC_009085.1| | 1381157 | 1381184 | 28 |
| exprReg_9357 | gi|126640115|ref|NC_009085.1| | 1382564 | 1382604 | 41 |
| exprReg_9358 | gi|126640115|ref|NC_009085.1| | 1382730 | 1382755 | 26 |
| exprReg_9371 | gi|126640115|ref|NC_009085.1| | 1384894 | 1384911 | 18 |
| exprReg_9372 | gi|126640115|ref|NC_009085.1| | 1384939 | 1384958 | 20 |
| exprReg_9373 | gi|126640115|ref|NC_009085.1| | 1385025 | 1385131 | 107 |
| exprReg_9377 | gi|126640115|ref|NC_009085.1| | 1385641 | 1385799 | 159 |
| exprReg_9378 | gi|126640115|ref|NC_009085.1| | 1385884 | 1386195 | 312 |
| exprReg_9379 | gi|126640115|ref|NC_009085.1| | 1386240 | 1386259 | 20 |
| exprReg_9396 | gi|126640115|ref|NC_009085.1| | 1387919 | 1387939 | 21 |
| exprReg_9397 | gi|126640115|ref|NC_009085.1| | 1387958 | 1388047 | 90 |
| exprReg_9419 | gi|126640115|ref|NC_009085.1| | 1391677 | 1391800 | 124 |
| exprReg_9420 | gi|126640115|ref|NC_009085.1| | 1391824 | 1391915 | 92 |
| exprReg_9421 | gi|126640115|ref|NC_009085.1| | 1391934 | 1392092 | 159 |
| exprReg_9438 | gi|126640115|ref|NC_009085.1| | 1394059 | 1394196 | 138 |
| exprReg_9439 | gi|126640115|ref|NC_009085.1| | 1394198 | 1394214 | 17 |
| exprReg_9440 | gi|126640115|ref|NC_009085.1| | 1394233 | 1394253 | 21 |
| exprReg_9441 | gi|126640115|ref|NC_009085.1| | 1394262 | 1394290 | 29 |
| exprReg_9442 | gi|126640115|ref|NC_009085.1| | 1394576 | 1394750 | 175 |
| exprReg_9443 | gi|126640115|ref|NC_009085.1| | 1394800 | 1394817 | 18 |
| exprReg_9444 | gi|126640115|ref|NC_009085.1| | 1394892 | 1394930 | 39 |
| exprReg_9453 | gi|126640115|ref|NC_009085.1| | 1396063 | 1396183 | 121 |
| exprReg_9462 | gi|126640115|ref|NC_009085.1| | 1397388 | 1397566 | 179 |
| exprReg_9463 | gi|126640115|ref|NC_009085.1| | 1397644 | 1397661 | 18 |
| exprReg_9473 | gi|126640115|ref|NC_009085.1| | 1399008 | 1399027 | 20 |
| exprReg_9474 | gi|126640115|ref|NC_009085.1| | 1399051 | 1399070 | 20 |
| exprReg_9483 | gi|126640115|ref|NC_009085.1| | 1400367 | 1400441 | 75 |
| exprReg_9489 | gi|126640115|ref|NC_009085.1| | 1401248 | 1401273 | 26 |
| exprReg_9501 | gi|126640115|ref|NC_009085.1| | 1404015 | 1404035 | 21 |
| exprReg_9506 | gi|126640115|ref|NC_009085.1| | 1404724 | 1405041 | 318 |
| exprReg_9507 | gi|126640115|ref|NC_009085.1| | 1405051 | 1405103 | 53 |
| exprReg_9515 | gi|126640115|ref|NC_009085.1| | 1407065 | 1407083 | 19 |
| exprReg_9517 | gi|126640115|ref|NC_009085.1| | 1407346 | 1407366 | 21 |
| exprReg_9525 | gi|126640115|ref|NC_009085.1| | 1408234 | 1408282 | 49 |
| exprReg_9530 | gi|126640115|ref|NC_009085.1| | 1409078 | 1409112 | 35 |
| exprReg_9531 | gi|126640115|ref|NC_009085.1| | 1409121 | 1409198 | 78 |
| exprReg_9532 | gi|126640115|ref|NC_009085.1| | 1409307 | 1409325 | 19 |
| exprReg_9533 | gi|126640115|ref|NC_009085.1| | 1409366 | 1409529 | 164 |
| exprReg_9563 | gi|126640115|ref|NC_009085.1| | 1413200 | 1413270 | 71 |
| exprReg_9564 | gi|126640115|ref|NC_009085.1| | 1413305 | 1413333 | 29 |
| exprReg_9583 | gi|126640115|ref|NC_009085.1| | 1415621 | 1415652 | 32 |
| exprReg_9584 | gi|126640115|ref|NC_009085.1| | 1415660 | 1415677 | 18 |
| exprReg_9595 | gi|126640115|ref|NC_009085.1| | 1417543 | 1417596 | 54 |
| exprReg_9604 | gi|126640115|ref|NC_009085.1| | 1418768 | 1418796 | 29 |
| exprReg_9623 | gi|126640115|ref|NC_009085.1| | 1422738 | 1422819 | 82 |
| exprReg_9624 | gi|126640115|ref|NC_009085.1| | 1422899 | 1422926 | 28 |
| exprReg_9625 | gi|126640115|ref|NC_009085.1| | 1422980 | 1423024 | 45 |
| exprReg_9626 | gi|126640115|ref|NC_009085.1| | 1423089 | 1423148 | 60 |
| exprReg_9632 | gi|126640115|ref|NC_009085.1| | 1423949 | 1424057 | 109 |
| exprReg_9633 | gi|126640115|ref|NC_009085.1| | 1424135 | 1424177 | 43 |
| exprReg_9634 | gi|126640115|ref|NC_009085.1| | 1424277 | 1424310 | 34 |
| exprReg_9655 | gi|126640115|ref|NC_009085.1| | 1427737 | 1427779 | 43 |
| exprReg_9656 | gi|126640115|ref|NC_009085.1| | 1427782 | 1427945 | 164 |
| exprReg_9657 | gi|126640115|ref|NC_009085.1| | 1428086 | 1428282 | 197 |
| exprReg_9658 | gi|126640115|ref|NC_009085.1| | 1428294 | 1428356 | 63 |
| exprReg_9659 | gi|126640115|ref|NC_009085.1| | 1428438 | 1428517 | 80 |
| exprReg_9660 | gi|126640115|ref|NC_009085.1| | 1428931 | 1429082 | 152 |
| exprReg_9661 | gi|126640115|ref|NC_009085.1| | 1429085 | 1429121 | 37 |
| exprReg_9662 | gi|126640115|ref|NC_009085.1| | 1429150 | 1429175 | 26 |
| exprReg_9663 | gi|126640115|ref|NC_009085.1| | 1429186 | 1429271 | 86 |
| exprReg_9664 | gi|126640115|ref|NC_009085.1| | 1429291 | 1429309 | 19 |
| exprReg_9665 | gi|126640115|ref|NC_009085.1| | 1429327 | 1429391 | 65 |
| exprReg_9666 | gi|126640115|ref|NC_009085.1| | 1429424 | 1429509 | 86 |
| exprReg_9667 | gi|126640115|ref|NC_009085.1| | 1429520 | 1429544 | 25 |
| exprReg_9668 | gi|126640115|ref|NC_009085.1| | 1429861 | 1429905 | 45 |
| exprReg_9669 | gi|126640115|ref|NC_009085.1| | 1429917 | 1429986 | 70 |
| exprReg_9670 | gi|126640115|ref|NC_009085.1| | 1430029 | 1430061 | 33 |
| exprReg_9674 | gi|126640115|ref|NC_009085.1| | 1430557 | 1430575 | 19 |
| exprReg_9675 | gi|126640115|ref|NC_009085.1| | 1430609 | 1430634 | 26 |
| exprReg_9685 | gi|126640115|ref|NC_009085.1| | 1432789 | 1432863 | 75 |
| exprReg_9692 | gi|126640115|ref|NC_009085.1| | 1433974 | 1433991 | 18 |
| exprReg_9693 | gi|126640115|ref|NC_009085.1| | 1434224 | 1434250 | 27 |
| exprReg_9694 | gi|126640115|ref|NC_009085.1| | 1434280 | 1434339 | 60 |
| exprReg_9695 | gi|126640115|ref|NC_009085.1| | 1434688 | 1434849 | 162 |
| exprReg_9703 | gi|126640115|ref|NC_009085.1| | 1436227 | 1436247 | 21 |
| exprReg_9704 | gi|126640115|ref|NC_009085.1| | 1436566 | 1436614 | 49 |
| exprReg_9705 | gi|126640115|ref|NC_009085.1| | 1436619 | 1436639 | 21 |
| exprReg_9706 | gi|126640115|ref|NC_009085.1| | 1436708 | 1436727 | 20 |
| exprReg_9709 | gi|126640115|ref|NC_009085.1| | 1437125 | 1437153 | 29 |
| exprReg_9710 | gi|126640115|ref|NC_009085.1| | 1437237 | 1437259 | 23 |
| exprReg_9711 | gi|126640115|ref|NC_009085.1| | 1437461 | 1437526 | 66 |
| exprReg_9712 | gi|126640115|ref|NC_009085.1| | 1437548 | 1437609 | 62 |
| exprReg_9713 | gi|126640115|ref|NC_009085.1| | 1437779 | 1437795 | 17 |
| exprReg_9714 | gi|126640115|ref|NC_009085.1| | 1437812 | 1437834 | 23 |
| exprReg_9715 | gi|126640115|ref|NC_009085.1| | 1437873 | 1437889 | 17 |
| exprReg_9718 | gi|126640115|ref|NC_009085.1| | 1438479 | 1438546 | 68 |
| exprReg_9719 | gi|126640115|ref|NC_009085.1| | 1438653 | 1438796 | 144 |
| exprReg_9720 | gi|126640115|ref|NC_009085.1| | 1438838 | 1438864 | 27 |
| exprReg_9721 | gi|126640115|ref|NC_009085.1| | 1438932 | 1438965 | 34 |
| exprReg_9722 | gi|126640115|ref|NC_009085.1| | 1438982 | 1439000 | 19 |
| exprReg_9723 | gi|126640115|ref|NC_009085.1| | 1439079 | 1439102 | 24 |
| exprReg_9724 | gi|126640115|ref|NC_009085.1| | 1439244 | 1439263 | 20 |
| exprReg_9725 | gi|126640115|ref|NC_009085.1| | 1439350 | 1439419 | 70 |
| exprReg_9726 | gi|126640115|ref|NC_009085.1| | 1439518 | 1439559 | 42 |
| exprReg_9727 | gi|126640115|ref|NC_009085.1| | 1439594 | 1439615 | 22 |
| exprReg_9728 | gi|126640115|ref|NC_009085.1| | 1439693 | 1439821 | 129 |
| exprReg_9729 | gi|126640115|ref|NC_009085.1| | 1439946 | 1439965 | 20 |
| exprReg_9730 | gi|126640115|ref|NC_009085.1| | 1439990 | 1440034 | 45 |
| exprReg_9731 | gi|126640115|ref|NC_009085.1| | 1440115 | 1440132 | 18 |
| exprReg_9732 | gi|126640115|ref|NC_009085.1| | 1440384 | 1440410 | 27 |
| exprReg_9733 | gi|126640115|ref|NC_009085.1| | 1440476 | 1440513 | 38 |
| exprReg_9737 | gi|126640115|ref|NC_009085.1| | 1441469 | 1441486 | 18 |
| exprReg_9744 | gi|126640115|ref|NC_009085.1| | 1442635 | 1442700 | 66 |
| exprReg_9745 | gi|126640115|ref|NC_009085.1| | 1442718 | 1442734 | 17 |
| exprReg_9746 | gi|126640115|ref|NC_009085.1| | 1442862 | 1442903 | 42 |
| exprReg_9751 | gi|126640115|ref|NC_009085.1| | 1443494 | 1443579 | 86 |
| exprReg_9752 | gi|126640115|ref|NC_009085.1| | 1443714 | 1443732 | 19 |
| exprReg_9753 | gi|126640115|ref|NC_009085.1| | 1443735 | 1443754 | 20 |
| exprReg_9754 | gi|126640115|ref|NC_009085.1| | 1443874 | 1443987 | 114 |
| exprReg_9755 | gi|126640115|ref|NC_009085.1| | 1444236 | 1444261 | 26 |
| exprReg_9756 | gi|126640115|ref|NC_009085.1| | 1444311 | 1444344 | 34 |
| exprReg_9757 | gi|126640115|ref|NC_009085.1| | 1444352 | 1444372 | 21 |
| exprReg_9758 | gi|126640115|ref|NC_009085.1| | 1444381 | 1444436 | 56 |
| exprReg_9762 | gi|126640115|ref|NC_009085.1| | 1445359 | 1445412 | 54 |
| exprReg_9763 | gi|126640115|ref|NC_009085.1| | 1445474 | 1445588 | 115 |
| exprReg_9764 | gi|126640115|ref|NC_009085.1| | 1445636 | 1445664 | 29 |
| exprReg_9765 | gi|126640115|ref|NC_009085.1| | 1445698 | 1445771 | 74 |
| exprReg_9766 | gi|126640115|ref|NC_009085.1| | 1445793 | 1445814 | 22 |
| exprReg_9767 | gi|126640115|ref|NC_009085.1| | 1445819 | 1445908 | 90 |
| exprReg_9768 | gi|126640115|ref|NC_009085.1| | 1445911 | 1446117 | 207 |
| exprReg_9769 | gi|126640115|ref|NC_009085.1| | 1446267 | 1446316 | 50 |
| exprReg_9780 | gi|126640115|ref|NC_009085.1| | 1448157 | 1448183 | 27 |
| exprReg_9781 | gi|126640115|ref|NC_009085.1| | 1448192 | 1448251 | 60 |
| exprReg_9782 | gi|126640115|ref|NC_009085.1| | 1448257 | 1448312 | 56 |
| exprReg_9794 | gi|126640115|ref|NC_009085.1| | 1449232 | 1449290 | 59 |
| exprReg_9798 | gi|126640115|ref|NC_009085.1| | 1449732 | 1449774 | 43 |
| exprReg_9803 | gi|126640115|ref|NC_009085.1| | 1450363 | 1450379 | 17 |
| exprReg_9814 | gi|126640115|ref|NC_009085.1| | 1451601 | 1451639 | 39 |
| exprReg_9815 | gi|126640115|ref|NC_009085.1| | 1451793 | 1451900 | 108 |
| exprReg_9816 | gi|126640115|ref|NC_009085.1| | 1451914 | 1451950 | 37 |
| exprReg_9838 | gi|126640115|ref|NC_009085.1| | 1455504 | 1455533 | 30 |
| exprReg_9846 | gi|126640115|ref|NC_009085.1| | 1456732 | 1456814 | 83 |
| exprReg_9865 | gi|126640115|ref|NC_009085.1| | 1459614 | 1459634 | 21 |
| exprReg_9866 | gi|126640115|ref|NC_009085.1| | 1459669 | 1459760 | 92 |
| exprReg_9873 | gi|126640115|ref|NC_009085.1| | 1460655 | 1460687 | 33 |
| exprReg_9877 | gi|126640115|ref|NC_009085.1| | 1460964 | 1461018 | 55 |
| exprReg_9878 | gi|126640115|ref|NC_009085.1| | 1461038 | 1461089 | 52 |
| exprReg_9899 | gi|126640115|ref|NC_009085.1| | 1463784 | 1463801 | 18 |
| exprReg_9900 | gi|126640115|ref|NC_009085.1| | 1463883 | 1463900 | 18 |
| exprReg_9907 | gi|126640115|ref|NC_009085.1| | 1464876 | 1464944 | 69 |
| exprReg_9908 | gi|126640115|ref|NC_009085.1| | 1464948 | 1464964 | 17 |
| exprReg_9909 | gi|126640115|ref|NC_009085.1| | 1464999 | 1465044 | 46 |
| exprReg_9910 | gi|126640115|ref|NC_009085.1| | 1465068 | 1465112 | 45 |
| exprReg_9911 | gi|126640115|ref|NC_009085.1| | 1465159 | 1465216 | 58 |
| exprReg_9912 | gi|126640115|ref|NC_009085.1| | 1465498 | 1465521 | 24 |
| exprReg_9913 | gi|126640115|ref|NC_009085.1| | 1465523 | 1465653 | 131 |
| exprReg_9918 | gi|126640115|ref|NC_009085.1| | 1466362 | 1466464 | 103 |
| exprReg_9919 | gi|126640115|ref|NC_009085.1| | 1466491 | 1466509 | 19 |
| exprReg_9920 | gi|126640115|ref|NC_009085.1| | 1466523 | 1466543 | 21 |
| exprReg_9921 | gi|126640115|ref|NC_009085.1| | 1466651 | 1466720 | 70 |
| exprReg_9922 | gi|126640115|ref|NC_009085.1| | 1466802 | 1466941 | 140 |
| exprReg_9933 | gi|126640115|ref|NC_009085.1| | 1468271 | 1468439 | 169 |
| exprReg_9941 | gi|126640115|ref|NC_009085.1| | 1469423 | 1469790 | 368 |
| exprReg_9942 | gi|126640115|ref|NC_009085.1| | 1469813 | 1469831 | 19 |
| exprReg_9943 | gi|126640115|ref|NC_009085.1| | 1470083 | 1470100 | 18 |
| exprReg_9944 | gi|126640115|ref|NC_009085.1| | 1470133 | 1470157 | 25 |
| exprReg_9945 | gi|126640115|ref|NC_009085.1| | 1470169 | 1470194 | 26 |
| exprReg_9946 | gi|126640115|ref|NC_009085.1| | 1470287 | 1470303 | 17 |
| exprReg_9947 | gi|126640115|ref|NC_009085.1| | 1470399 | 1470435 | 37 |
| exprReg_9948 | gi|126640115|ref|NC_009085.1| | 1470479 | 1470595 | 117 |
| exprReg_9949 | gi|126640115|ref|NC_009085.1| | 1470614 | 1470640 | 27 |
| exprReg_9950 | gi|126640115|ref|NC_009085.1| | 1470884 | 1471200 | 317 |
| exprReg_9951 | gi|126640115|ref|NC_009085.1| | 1471244 | 1471261 | 18 |
| exprReg_9952 | gi|126640115|ref|NC_009085.1| | 1471318 | 1471359 | 42 |
| exprReg_9958 | gi|126640115|ref|NC_009085.1| | 1472256 | 1472317 | 62 |
| exprReg_9969 | gi|126640115|ref|NC_009085.1| | 1474120 | 1474159 | 40 |
| exprReg_9970 | gi|126640115|ref|NC_009085.1| | 1474267 | 1474358 | 92 |
| exprReg_9976 | gi|126640115|ref|NC_009085.1| | 1475396 | 1475420 | 25 |
| exprReg_9977 | gi|126640115|ref|NC_009085.1| | 1475428 | 1475532 | 105 |
| exprReg_9978 | gi|126640115|ref|NC_009085.1| | 1475625 | 1475653 | 29 |
| exprReg_9982 | gi|126640115|ref|NC_009085.1| | 1476396 | 1476462 | 67 |
| exprReg_9983 | gi|126640115|ref|NC_009085.1| | 1476526 | 1476574 | 49 |
| exprReg_9984 | gi|126640115|ref|NC_009085.1| | 1476662 | 1476681 | 20 |
| exprReg_9985 | gi|126640115|ref|NC_009085.1| | 1476689 | 1476827 | 139 |
| exprReg_9990 | gi|126640115|ref|NC_009085.1| | 1477748 | 1477781 | 34 |
| exprReg_9991 | gi|126640115|ref|NC_009085.1| | 1477932 | 1477948 | 17 |
| exprReg_9992 | gi|126640115|ref|NC_009085.1| | 1478059 | 1478079 | 21 |
| exprReg_9993 | gi|126640115|ref|NC_009085.1| | 1478160 | 1478189 | 30 |
| exprReg_9994 | gi|126640115|ref|NC_009085.1| | 1478328 | 1478363 | 36 |
| exprReg_9995 | gi|126640115|ref|NC_009085.1| | 1478427 | 1478444 | 18 |
| exprReg_9996 | gi|126640115|ref|NC_009085.1| | 1478448 | 1478465 | 18 |
| exprReg_9997 | gi|126640115|ref|NC_009085.1| | 1478468 | 1478487 | 20 |
| exprReg_9998 | gi|126640115|ref|NC_009085.1| | 1478550 | 1478603 | 54 |
| exprReg_10004 | gi|126640115|ref|NC_009085.1| | 1479196 | 1479249 | 54 |
| exprReg_10009 | gi|126640115|ref|NC_009085.1| | 1479781 | 1479819 | 39 |
| exprReg_10010 | gi|126640115|ref|NC_009085.1| | 1479822 | 1479840 | 19 |
| exprReg_10011 | gi|126640115|ref|NC_009085.1| | 1479950 | 1479987 | 38 |
| exprReg_10020 | gi|126640115|ref|NC_009085.1| | 1481394 | 1481446 | 53 |
| exprReg_10021 | gi|126640115|ref|NC_009085.1| | 1481683 | 1481716 | 34 |
| exprReg_10022 | gi|126640115|ref|NC_009085.1| | 1481787 | 1482138 | 352 |
| exprReg_10023 | gi|126640115|ref|NC_009085.1| | 1482344 | 1482361 | 18 |
| exprReg_10024 | gi|126640115|ref|NC_009085.1| | 1482399 | 1482424 | 26 |
| exprReg_10031 | gi|126640115|ref|NC_009085.1| | 1483468 | 1483509 | 42 |
| exprReg_10040 | gi|126640115|ref|NC_009085.1| | 1484864 | 1484899 | 36 |
| exprReg_10041 | gi|126640115|ref|NC_009085.1| | 1484960 | 1485034 | 75 |
| exprReg_10042 | gi|126640115|ref|NC_009085.1| | 1485057 | 1485098 | 42 |
| exprReg_10051 | gi|126640115|ref|NC_009085.1| | 1486514 | 1486622 | 109 |
| exprReg_10070 | gi|126640115|ref|NC_009085.1| | 1489908 | 1489927 | 20 |
| exprReg_10071 | gi|126640115|ref|NC_009085.1| | 1489951 | 1490010 | 60 |
| exprReg_10072 | gi|126640115|ref|NC_009085.1| | 1490092 | 1490206 | 115 |
| exprReg_10073 | gi|126640115|ref|NC_009085.1| | 1490332 | 1490348 | 17 |
| exprReg_10079 | gi|126640115|ref|NC_009085.1| | 1491435 | 1491464 | 30 |
| exprReg_10080 | gi|126640115|ref|NC_009085.1| | 1491613 | 1491631 | 19 |
| exprReg_10081 | gi|126640115|ref|NC_009085.1| | 1491683 | 1491733 | 51 |
| exprReg_10082 | gi|126640115|ref|NC_009085.1| | 1491847 | 1491872 | 26 |
| exprReg_10083 | gi|126640115|ref|NC_009085.1| | 1491876 | 1491899 | 24 |
| exprReg_10084 | gi|126640115|ref|NC_009085.1| | 1491912 | 1492095 | 184 |
| exprReg_10085 | gi|126640115|ref|NC_009085.1| | 1492105 | 1492135 | 31 |
| exprReg_10086 | gi|126640115|ref|NC_009085.1| | 1492340 | 1492366 | 27 |
| exprReg_10087 | gi|126640115|ref|NC_009085.1| | 1492387 | 1492426 | 40 |
| exprReg_10088 | gi|126640115|ref|NC_009085.1| | 1492459 | 1492476 | 18 |
| exprReg_10089 | gi|126640115|ref|NC_009085.1| | 1492824 | 1492979 | 156 |
| exprReg_10090 | gi|126640115|ref|NC_009085.1| | 1493140 | 1493445 | 306 |
| exprReg_10091 | gi|126640115|ref|NC_009085.1| | 1493819 | 1493836 | 18 |
| exprReg_10095 | gi|126640115|ref|NC_009085.1| | 1494280 | 1494299 | 20 |
| exprReg_10098 | gi|126640115|ref|NC_009085.1| | 1494777 | 1494815 | 39 |
| exprReg_10099 | gi|126640115|ref|NC_009085.1| | 1495048 | 1495095 | 48 |
| exprReg_10104 | gi|126640115|ref|NC_009085.1| | 1496348 | 1496446 | 99 |
| exprReg_10105 | gi|126640115|ref|NC_009085.1| | 1496456 | 1496600 | 145 |
| exprReg_10133 | gi|126640115|ref|NC_009085.1| | 1500715 | 1500793 | 79 |
| exprReg_10134 | gi|126640115|ref|NC_009085.1| | 1500805 | 1500827 | 23 |
| exprReg_10141 | gi|126640115|ref|NC_009085.1| | 1502048 | 1502114 | 67 |
| exprReg_10142 | gi|126640115|ref|NC_009085.1| | 1502129 | 1502176 | 48 |
| exprReg_10144 | gi|126640115|ref|NC_009085.1| | 1502740 | 1502764 | 25 |
| exprReg_10145 | gi|126640115|ref|NC_009085.1| | 1502783 | 1502800 | 18 |
| exprReg_10150 | gi|126640115|ref|NC_009085.1| | 1504378 | 1504400 | 23 |
| exprReg_10151 | gi|126640115|ref|NC_009085.1| | 1504749 | 1504765 | 17 |
| exprReg_10152 | gi|126640115|ref|NC_009085.1| | 1505323 | 1505366 | 44 |
| exprReg_10153 | gi|126640115|ref|NC_009085.1| | 1505379 | 1505400 | 22 |
| exprReg_10154 | gi|126640115|ref|NC_009085.1| | 1505433 | 1505453 | 21 |
| exprReg_10155 | gi|126640115|ref|NC_009085.1| | 1505514 | 1505642 | 129 |
| exprReg_10156 | gi|126640115|ref|NC_009085.1| | 1505674 | 1505717 | 44 |
| exprReg_10191 | gi|126640115|ref|NC_009085.1| | 1510801 | 1510826 | 26 |
| exprReg_10192 | gi|126640115|ref|NC_009085.1| | 1510921 | 1510957 | 37 |
| exprReg_10193 | gi|126640115|ref|NC_009085.1| | 1510986 | 1511023 | 38 |
| exprReg_10194 | gi|126640115|ref|NC_009085.1| | 1511148 | 1511292 | 145 |
| exprReg_10195 | gi|126640115|ref|NC_009085.1| | 1511299 | 1511321 | 23 |
| exprReg_10196 | gi|126640115|ref|NC_009085.1| | 1511430 | 1511506 | 77 |
| exprReg_10197 | gi|126640115|ref|NC_009085.1| | 1511512 | 1511541 | 30 |
| exprReg_10198 | gi|126640115|ref|NC_009085.1| | 1511569 | 1511600 | 32 |
| exprReg_10235 | gi|126640115|ref|NC_009085.1| | 1517272 | 1517304 | 33 |
| exprReg_10236 | gi|126640115|ref|NC_009085.1| | 1517317 | 1517358 | 42 |
| exprReg_10248 | gi|126640115|ref|NC_009085.1| | 1518402 | 1518460 | 59 |
| exprReg_10249 | gi|126640115|ref|NC_009085.1| | 1518842 | 1518859 | 18 |
| exprReg_10250 | gi|126640115|ref|NC_009085.1| | 1518976 | 1518992 | 17 |
| exprReg_10251 | gi|126640115|ref|NC_009085.1| | 1519004 | 1519120 | 117 |
| exprReg_10260 | gi|126640115|ref|NC_009085.1| | 1519941 | 1519959 | 19 |
| exprReg_10262 | gi|126640115|ref|NC_009085.1| | 1520236 | 1520252 | 17 |
| exprReg_10282 | gi|126640115|ref|NC_009085.1| | 1522871 | 1522888 | 18 |
| exprReg_10283 | gi|126640115|ref|NC_009085.1| | 1522984 | 1523004 | 21 |
| exprReg_10287 | gi|126640115|ref|NC_009085.1| | 1523639 | 1523711 | 73 |
| exprReg_10288 | gi|126640115|ref|NC_009085.1| | 1523722 | 1523739 | 18 |
| exprReg_10289 | gi|126640115|ref|NC_009085.1| | 1523758 | 1523811 | 54 |
| exprReg_10290 | gi|126640115|ref|NC_009085.1| | 1523830 | 1523917 | 88 |
| exprReg_10304 | gi|126640115|ref|NC_009085.1| | 1525627 | 1525675 | 49 |
| exprReg_10305 | gi|126640115|ref|NC_009085.1| | 1525677 | 1525706 | 30 |
| exprReg_10341 | gi|126640115|ref|NC_009085.1| | 1530954 | 1531028 | 75 |
| exprReg_10366 | gi|126640115|ref|NC_009085.1| | 1535861 | 1536045 | 185 |
| exprReg_10372 | gi|126640115|ref|NC_009085.1| | 1536970 | 1537056 | 87 |
| exprReg_10387 | gi|126640115|ref|NC_009085.1| | 1539211 | 1539267 | 57 |
| exprReg_10388 | gi|126640115|ref|NC_009085.1| | 1539319 | 1539363 | 45 |
| exprReg_10389 | gi|126640115|ref|NC_009085.1| | 1539389 | 1539483 | 95 |
| exprReg_10390 | gi|126640115|ref|NC_009085.1| | 1539490 | 1539577 | 88 |
| exprReg_10391 | gi|126640115|ref|NC_009085.1| | 1539601 | 1539633 | 33 |
| exprReg_10392 | gi|126640115|ref|NC_009085.1| | 1539655 | 1539730 | 76 |
| exprReg_10395 | gi|126640115|ref|NC_009085.1| | 1540134 | 1540151 | 18 |
| exprReg_10403 | gi|126640115|ref|NC_009085.1| | 1541510 | 1541633 | 124 |
| exprReg_10420 | gi|126640115|ref|NC_009085.1| | 1544258 | 1544313 | 56 |
| exprReg_10421 | gi|126640115|ref|NC_009085.1| | 1544393 | 1544450 | 58 |
| exprReg_10432 | gi|126640115|ref|NC_009085.1| | 1545777 | 1545809 | 33 |
| exprReg_10433 | gi|126640115|ref|NC_009085.1| | 1545876 | 1545938 | 63 |
| exprReg_10434 | gi|126640115|ref|NC_009085.1| | 1546041 | 1546152 | 112 |
| exprReg_10441 | gi|126640115|ref|NC_009085.1| | 1547249 | 1547383 | 135 |
| exprReg_10442 | gi|126640115|ref|NC_009085.1| | 1547481 | 1547525 | 45 |
| exprReg_10443 | gi|126640115|ref|NC_009085.1| | 1547769 | 1547788 | 20 |
| exprReg_10444 | gi|126640115|ref|NC_009085.1| | 1547816 | 1547835 | 20 |
| exprReg_10452 | gi|126640115|ref|NC_009085.1| | 1548896 | 1548988 | 93 |
| exprReg_10453 | gi|126640115|ref|NC_009085.1| | 1549197 | 1549402 | 206 |
| exprReg_10463 | gi|126640115|ref|NC_009085.1| | 1551503 | 1551520 | 18 |
| exprReg_10464 | gi|126640115|ref|NC_009085.1| | 1551543 | 1551561 | 19 |
| exprReg_10465 | gi|126640115|ref|NC_009085.1| | 1551571 | 1551652 | 82 |
| exprReg_10466 | gi|126640115|ref|NC_009085.1| | 1551880 | 1551997 | 118 |
| exprReg_10486 | gi|126640115|ref|NC_009085.1| | 1555178 | 1555223 | 46 |
| exprReg_10493 | gi|126640115|ref|NC_009085.1| | 1555745 | 1555844 | 100 |
| exprReg_10497 | gi|126640115|ref|NC_009085.1| | 1556435 | 1556451 | 17 |
| exprReg_10508 | gi|126640115|ref|NC_009085.1| | 1558321 | 1558371 | 51 |
| exprReg_10509 | gi|126640115|ref|NC_009085.1| | 1558429 | 1558486 | 58 |
| exprReg_10522 | gi|126640115|ref|NC_009085.1| | 1560141 | 1560210 | 70 |
| exprReg_10529 | gi|126640115|ref|NC_009085.1| | 1561926 | 1562009 | 84 |
| exprReg_10530 | gi|126640115|ref|NC_009085.1| | 1562339 | 1562384 | 46 |
| exprReg_10544 | gi|126640115|ref|NC_009085.1| | 1563820 | 1563848 | 29 |
| exprReg_10557 | gi|126640115|ref|NC_009085.1| | 1565829 | 1565849 | 21 |
| exprReg_10558 | gi|126640115|ref|NC_009085.1| | 1565852 | 1565868 | 17 |
| exprReg_10559 | gi|126640115|ref|NC_009085.1| | 1565898 | 1565964 | 67 |
| exprReg_10560 | gi|126640115|ref|NC_009085.1| | 1565975 | 1566010 | 36 |
| exprReg_10568 | gi|126640115|ref|NC_009085.1| | 1567527 | 1567545 | 19 |
| exprReg_10575 | gi|126640115|ref|NC_009085.1| | 1568819 | 1568940 | 122 |
| exprReg_10576 | gi|126640115|ref|NC_009085.1| | 1568982 | 1569074 | 93 |
| exprReg_10604 | gi|126640115|ref|NC_009085.1| | 1572979 | 1573086 | 108 |
| exprReg_10623 | gi|126640115|ref|NC_009085.1| | 1574960 | 1574982 | 23 |
| exprReg_10624 | gi|126640115|ref|NC_009085.1| | 1574987 | 1575061 | 75 |
| exprReg_10625 | gi|126640115|ref|NC_009085.1| | 1575072 | 1575140 | 69 |
| exprReg_10641 | gi|126640115|ref|NC_009085.1| | 1577175 | 1577228 | 54 |
| exprReg_10646 | gi|126640115|ref|NC_009085.1| | 1577859 | 1577877 | 19 |
| exprReg_10647 | gi|126640115|ref|NC_009085.1| | 1578024 | 1578065 | 42 |
| exprReg_10648 | gi|126640115|ref|NC_009085.1| | 1578107 | 1578148 | 42 |
| exprReg_10649 | gi|126640115|ref|NC_009085.1| | 1578169 | 1578187 | 19 |
| exprReg_10650 | gi|126640115|ref|NC_009085.1| | 1578190 | 1578207 | 18 |
| exprReg_10651 | gi|126640115|ref|NC_009085.1| | 1578220 | 1578256 | 37 |
| exprReg_10652 | gi|126640115|ref|NC_009085.1| | 1578329 | 1578464 | 136 |
| exprReg_10653 | gi|126640115|ref|NC_009085.1| | 1578474 | 1578673 | 200 |
| exprReg_10654 | gi|126640115|ref|NC_009085.1| | 1578683 | 1578756 | 74 |
| exprReg_10655 | gi|126640115|ref|NC_009085.1| | 1578765 | 1578831 | 67 |
| exprReg_10656 | gi|126640115|ref|NC_009085.1| | 1578925 | 1578946 | 22 |
| exprReg_10657 | gi|126640115|ref|NC_009085.1| | 1579020 | 1579063 | 44 |
| exprReg_10658 | gi|126640115|ref|NC_009085.1| | 1579453 | 1579500 | 48 |
| exprReg_10659 | gi|126640115|ref|NC_009085.1| | 1579701 | 1579755 | 55 |
| exprReg_10660 | gi|126640115|ref|NC_009085.1| | 1579992 | 1580013 | 22 |
| exprReg_10661 | gi|126640115|ref|NC_009085.1| | 1580048 | 1580106 | 59 |
| exprReg_10662 | gi|126640115|ref|NC_009085.1| | 1580294 | 1580314 | 21 |
| exprReg_10663 | gi|126640115|ref|NC_009085.1| | 1580354 | 1580390 | 37 |
| exprReg_10664 | gi|126640115|ref|NC_009085.1| | 1580400 | 1580556 | 157 |
| exprReg_10665 | gi|126640115|ref|NC_009085.1| | 1580921 | 1581212 | 292 |
| exprReg_10666 | gi|126640115|ref|NC_009085.1| | 1581232 | 1581276 | 45 |
| exprReg_10672 | gi|126640115|ref|NC_009085.1| | 1582050 | 1582067 | 18 |
| exprReg_10673 | gi|126640115|ref|NC_009085.1| | 1582200 | 1582268 | 69 |
| exprReg_10674 | gi|126640115|ref|NC_009085.1| | 1582292 | 1582323 | 32 |
| exprReg_10675 | gi|126640115|ref|NC_009085.1| | 1582649 | 1582669 | 21 |
| exprReg_10676 | gi|126640115|ref|NC_009085.1| | 1582741 | 1582765 | 25 |
| exprReg_10677 | gi|126640115|ref|NC_009085.1| | 1582872 | 1582889 | 18 |
| exprReg_10686 | gi|126640115|ref|NC_009085.1| | 1584651 | 1584685 | 35 |
| exprReg_10687 | gi|126640115|ref|NC_009085.1| | 1584781 | 1584799 | 19 |
| exprReg_10693 | gi|126640115|ref|NC_009085.1| | 1585610 | 1585630 | 21 |
| exprReg_10694 | gi|126640115|ref|NC_009085.1| | 1585725 | 1585752 | 28 |
| exprReg_10700 | gi|126640115|ref|NC_009085.1| | 1587255 | 1587488 | 234 |
| exprReg_10701 | gi|126640115|ref|NC_009085.1| | 1587515 | 1587534 | 20 |
| exprReg_10721 | gi|126640115|ref|NC_009085.1| | 1590152 | 1590250 | 99 |
| exprReg_10722 | gi|126640115|ref|NC_009085.1| | 1590430 | 1590446 | 17 |
| exprReg_10723 | gi|126640115|ref|NC_009085.1| | 1591048 | 1591065 | 18 |
| exprReg_10724 | gi|126640115|ref|NC_009085.1| | 1591173 | 1591191 | 19 |
| exprReg_10725 | gi|126640115|ref|NC_009085.1| | 1591340 | 1591361 | 22 |
| exprReg_10728 | gi|126640115|ref|NC_009085.1| | 1592156 | 1592213 | 58 |
| exprReg_10736 | gi|126640115|ref|NC_009085.1| | 1593641 | 1593698 | 58 |
| exprReg_10749 | gi|126640115|ref|NC_009085.1| | 1596160 | 1596229 | 70 |
| exprReg_10750 | gi|126640115|ref|NC_009085.1| | 1596310 | 1596346 | 37 |
| exprReg_10771 | gi|126640115|ref|NC_009085.1| | 1599591 | 1599612 | 22 |
| exprReg_10778 | gi|126640115|ref|NC_009085.1| | 1600319 | 1600338 | 20 |
| exprReg_10779 | gi|126640115|ref|NC_009085.1| | 1600820 | 1600849 | 30 |
| exprReg_10780 | gi|126640115|ref|NC_009085.1| | 1600881 | 1600900 | 20 |
| exprReg_10800 | gi|126640115|ref|NC_009085.1| | 1605174 | 1605243 | 70 |
| exprReg_10801 | gi|126640115|ref|NC_009085.1| | 1605255 | 1605286 | 32 |
| exprReg_10802 | gi|126640115|ref|NC_009085.1| | 1605296 | 1605333 | 38 |
| exprReg_10852 | gi|126640115|ref|NC_009085.1| | 1613095 | 1613163 | 69 |
| exprReg_10853 | gi|126640115|ref|NC_009085.1| | 1613254 | 1613423 | 170 |
| exprReg_10858 | gi|126640115|ref|NC_009085.1| | 1613954 | 1613986 | 33 |
| exprReg_10859 | gi|126640115|ref|NC_009085.1| | 1613999 | 1614097 | 99 |
| exprReg_10860 | gi|126640115|ref|NC_009085.1| | 1614197 | 1614213 | 17 |
| exprReg_10900 | gi|126640115|ref|NC_009085.1| | 1620259 | 1620277 | 19 |
| exprReg_10901 | gi|126640115|ref|NC_009085.1| | 1620364 | 1620438 | 75 |
| exprReg_10902 | gi|126640115|ref|NC_009085.1| | 1620536 | 1620557 | 22 |
| exprReg_10903 | gi|126640115|ref|NC_009085.1| | 1620614 | 1620634 | 21 |
| exprReg_10904 | gi|126640115|ref|NC_009085.1| | 1620636 | 1620667 | 32 |
| exprReg_10905 | gi|126640115|ref|NC_009085.1| | 1620682 | 1620700 | 19 |
| exprReg_10906 | gi|126640115|ref|NC_009085.1| | 1620716 | 1620736 | 21 |
| exprReg_10907 | gi|126640115|ref|NC_009085.1| | 1620810 | 1621033 | 224 |
| exprReg_10908 | gi|126640115|ref|NC_009085.1| | 1621288 | 1621313 | 26 |
| exprReg_10909 | gi|126640115|ref|NC_009085.1| | 1621433 | 1621468 | 36 |
| exprReg_10910 | gi|126640115|ref|NC_009085.1| | 1621507 | 1621530 | 24 |
| exprReg_10911 | gi|126640115|ref|NC_009085.1| | 1621536 | 1621553 | 18 |
| exprReg_10912 | gi|126640115|ref|NC_009085.1| | 1621604 | 1621652 | 49 |
| exprReg_10913 | gi|126640115|ref|NC_009085.1| | 1621912 | 1621929 | 18 |
| exprReg_10914 | gi|126640115|ref|NC_009085.1| | 1621984 | 1622003 | 20 |
| exprReg_10915 | gi|126640115|ref|NC_009085.1| | 1622138 | 1622173 | 36 |
| exprReg_10916 | gi|126640115|ref|NC_009085.1| | 1622225 | 1622253 | 29 |
| exprReg_10917 | gi|126640115|ref|NC_009085.1| | 1622404 | 1622471 | 68 |
| exprReg_10918 | gi|126640115|ref|NC_009085.1| | 1622501 | 1622568 | 68 |
| exprReg_10919 | gi|126640115|ref|NC_009085.1| | 1622596 | 1622616 | 21 |
| exprReg_10925 | gi|126640115|ref|NC_009085.1| | 1623031 | 1623096 | 66 |
| exprReg_10926 | gi|126640115|ref|NC_009085.1| | 1623098 | 1623339 | 242 |
| exprReg_10927 | gi|126640115|ref|NC_009085.1| | 1623355 | 1623413 | 59 |
| exprReg_10928 | gi|126640115|ref|NC_009085.1| | 1623427 | 1623451 | 25 |
| exprReg_10929 | gi|126640115|ref|NC_009085.1| | 1623680 | 1623713 | 34 |
| exprReg_10936 | gi|126640115|ref|NC_009085.1| | 1624234 | 1624320 | 87 |
| exprReg_10937 | gi|126640115|ref|NC_009085.1| | 1624373 | 1624556 | 184 |
| exprReg_10948 | gi|126640115|ref|NC_009085.1| | 1625882 | 1625899 | 18 |
| exprReg_10949 | gi|126640115|ref|NC_009085.1| | 1625920 | 1625946 | 27 |
| exprReg_10950 | gi|126640115|ref|NC_009085.1| | 1625967 | 1625990 | 24 |
| exprReg_10951 | gi|126640115|ref|NC_009085.1| | 1626054 | 1626128 | 75 |
| exprReg_10963 | gi|126640115|ref|NC_009085.1| | 1628089 | 1628107 | 19 |
| exprReg_10971 | gi|126640115|ref|NC_009085.1| | 1628996 | 1629018 | 23 |
| exprReg_10972 | gi|126640115|ref|NC_009085.1| | 1629073 | 1629147 | 75 |
| exprReg_10973 | gi|126640115|ref|NC_009085.1| | 1629193 | 1629229 | 37 |
| exprReg_10974 | gi|126640115|ref|NC_009085.1| | 1629395 | 1629469 | 75 |
| exprReg_10975 | gi|126640115|ref|NC_009085.1| | 1629493 | 1629510 | 18 |
| exprReg_10976 | gi|126640115|ref|NC_009085.1| | 1629527 | 1629570 | 44 |
| exprReg_10977 | gi|126640115|ref|NC_009085.1| | 1630004 | 1630049 | 46 |
| exprReg_10978 | gi|126640115|ref|NC_009085.1| | 1630135 | 1630156 | 22 |
| exprReg_10979 | gi|126640115|ref|NC_009085.1| | 1630180 | 1630234 | 55 |
| exprReg_10980 | gi|126640115|ref|NC_009085.1| | 1630261 | 1630358 | 98 |
| exprReg_10981 | gi|126640115|ref|NC_009085.1| | 1630374 | 1630647 | 274 |
| exprReg_10982 | gi|126640115|ref|NC_009085.1| | 1630664 | 1630686 | 23 |
| exprReg_10985 | gi|126640115|ref|NC_009085.1| | 1631120 | 1631150 | 31 |
| exprReg_10991 | gi|126640115|ref|NC_009085.1| | 1631899 | 1631949 | 51 |
| exprReg_10996 | gi|126640115|ref|NC_009085.1| | 1632789 | 1632805 | 17 |
| exprReg_11008 | gi|126640115|ref|NC_009085.1| | 1635045 | 1635161 | 117 |
| exprReg_11025 | gi|126640115|ref|NC_009085.1| | 1638554 | 1638607 | 54 |
| exprReg_11031 | gi|126640115|ref|NC_009085.1| | 1639498 | 1639538 | 41 |
| exprReg_11034 | gi|126640115|ref|NC_009085.1| | 1640183 | 1640204 | 22 |
| exprReg_11039 | gi|126640115|ref|NC_009085.1| | 1640918 | 1640941 | 24 |
| exprReg_11045 | gi|126640115|ref|NC_009085.1| | 1642295 | 1642358 | 64 |
| exprReg_11048 | gi|126640115|ref|NC_009085.1| | 1643158 | 1643199 | 42 |
| exprReg_11049 | gi|126640115|ref|NC_009085.1| | 1643264 | 1643507 | 244 |
| exprReg_11050 | gi|126640115|ref|NC_009085.1| | 1643546 | 1643563 | 18 |
| exprReg_11055 | gi|126640115|ref|NC_009085.1| | 1644116 | 1644216 | 101 |
| exprReg_11061 | gi|126640115|ref|NC_009085.1| | 1645026 | 1645053 | 28 |
| exprReg_11062 | gi|126640115|ref|NC_009085.1| | 1645178 | 1645236 | 59 |
| exprReg_11065 | gi|126640115|ref|NC_009085.1| | 1646089 | 1646202 | 114 |
| exprReg_11066 | gi|126640115|ref|NC_009085.1| | 1646293 | 1646318 | 26 |
| exprReg_11067 | gi|126640115|ref|NC_009085.1| | 1646440 | 1646516 | 77 |
| exprReg_11114 | gi|126640115|ref|NC_009085.1| | 1654634 | 1654720 | 87 |
| exprReg_11115 | gi|126640115|ref|NC_009085.1| | 1654842 | 1654862 | 21 |
| exprReg_11120 | gi|126640115|ref|NC_009085.1| | 1655691 | 1655708 | 18 |
| exprReg_11121 | gi|126640115|ref|NC_009085.1| | 1655855 | 1655897 | 43 |
| exprReg_11122 | gi|126640115|ref|NC_009085.1| | 1656005 | 1656028 | 24 |
| exprReg_11123 | gi|126640115|ref|NC_009085.1| | 1656084 | 1656148 | 65 |
| exprReg_11124 | gi|126640115|ref|NC_009085.1| | 1656199 | 1656232 | 34 |
| exprReg_11125 | gi|126640115|ref|NC_009085.1| | 1656237 | 1656356 | 120 |
| exprReg_11126 | gi|126640115|ref|NC_009085.1| | 1656367 | 1656423 | 57 |
| exprReg_11139 | gi|126640115|ref|NC_009085.1| | 1658514 | 1658546 | 33 |
| exprReg_11140 | gi|126640115|ref|NC_009085.1| | 1658607 | 1658630 | 24 |
| exprReg_11141 | gi|126640115|ref|NC_009085.1| | 1658667 | 1658762 | 96 |
| exprReg_11160 | gi|126640115|ref|NC_009085.1| | 1661766 | 1661809 | 44 |
| exprReg_11161 | gi|126640115|ref|NC_009085.1| | 1661981 | 1662041 | 61 |
| exprReg_11188 | gi|126640115|ref|NC_009085.1| | 1666727 | 1666761 | 35 |
| exprReg_11195 | gi|126640115|ref|NC_009085.1| | 1668168 | 1668189 | 22 |
| exprReg_11201 | gi|126640115|ref|NC_009085.1| | 1669114 | 1669155 | 42 |
| exprReg_11219 | gi|126640115|ref|NC_009085.1| | 1671236 | 1671253 | 18 |
| exprReg_11235 | gi|126640115|ref|NC_009085.1| | 1672747 | 1672765 | 19 |
| exprReg_11236 | gi|126640115|ref|NC_009085.1| | 1672820 | 1672846 | 27 |
| exprReg_11252 | gi|126640115|ref|NC_009085.1| | 1675475 | 1675491 | 17 |
| exprReg_11253 | gi|126640115|ref|NC_009085.1| | 1675517 | 1675619 | 103 |
| exprReg_11254 | gi|126640115|ref|NC_009085.1| | 1675654 | 1675740 | 87 |
| exprReg_11255 | gi|126640115|ref|NC_009085.1| | 1675758 | 1675775 | 18 |
| exprReg_11256 | gi|126640115|ref|NC_009085.1| | 1675782 | 1675851 | 70 |
| exprReg_11257 | gi|126640115|ref|NC_009085.1| | 1675858 | 1675905 | 48 |
| exprReg_11258 | gi|126640115|ref|NC_009085.1| | 1675926 | 1676015 | 90 |
| exprReg_11259 | gi|126640115|ref|NC_009085.1| | 1676036 | 1676075 | 40 |
| exprReg_11260 | gi|126640115|ref|NC_009085.1| | 1676103 | 1676177 | 75 |
| exprReg_11272 | gi|126640115|ref|NC_009085.1| | 1678483 | 1678538 | 56 |
| exprReg_11273 | gi|126640115|ref|NC_009085.1| | 1678579 | 1678622 | 44 |
| exprReg_11274 | gi|126640115|ref|NC_009085.1| | 1678732 | 1678760 | 29 |
| exprReg_11280 | gi|126640115|ref|NC_009085.1| | 1680278 | 1680294 | 17 |
| exprReg_11296 | gi|126640115|ref|NC_009085.1| | 1682945 | 1682963 | 19 |
| exprReg_11337 | gi|126640115|ref|NC_009085.1| | 1689557 | 1689574 | 18 |
| exprReg_11338 | gi|126640115|ref|NC_009085.1| | 1689616 | 1689632 | 17 |
| exprReg_11347 | gi|126640115|ref|NC_009085.1| | 1691138 | 1691154 | 17 |
| exprReg_11348 | gi|126640115|ref|NC_009085.1| | 1691277 | 1691312 | 36 |
| exprReg_11349 | gi|126640115|ref|NC_009085.1| | 1691396 | 1691435 | 40 |
| exprReg_11354 | gi|126640115|ref|NC_009085.1| | 1692417 | 1692499 | 83 |
| exprReg_11355 | gi|126640115|ref|NC_009085.1| | 1692502 | 1692545 | 44 |
| exprReg_11368 | gi|126640115|ref|NC_009085.1| | 1694306 | 1694354 | 49 |
| exprReg_11369 | gi|126640115|ref|NC_009085.1| | 1694391 | 1694444 | 54 |
| exprReg_11375 | gi|126640115|ref|NC_009085.1| | 1695522 | 1695546 | 25 |
| exprReg_11376 | gi|126640115|ref|NC_009085.1| | 1695561 | 1695589 | 29 |
| exprReg_11386 | gi|126640115|ref|NC_009085.1| | 1697559 | 1697714 | 156 |
| exprReg_11394 | gi|126640115|ref|NC_009085.1| | 1698751 | 1698875 | 125 |
| exprReg_11395 | gi|126640115|ref|NC_009085.1| | 1698907 | 1698947 | 41 |
| exprReg_11420 | gi|126640115|ref|NC_009085.1| | 1703416 | 1703534 | 119 |
| exprReg_11428 | gi|126640115|ref|NC_009085.1| | 1704347 | 1704372 | 26 |
| exprReg_11435 | gi|126640115|ref|NC_009085.1| | 1705253 | 1705330 | 78 |
| exprReg_11467 | gi|126640115|ref|NC_009085.1| | 1708846 | 1708927 | 82 |
| exprReg_11468 | gi|126640115|ref|NC_009085.1| | 1708929 | 1708996 | 68 |
| exprReg_11473 | gi|126640115|ref|NC_009085.1| | 1710119 | 1710168 | 50 |
| exprReg_11474 | gi|126640115|ref|NC_009085.1| | 1710214 | 1710242 | 29 |
| exprReg_11475 | gi|126640115|ref|NC_009085.1| | 1710266 | 1710368 | 103 |
| exprReg_11476 | gi|126640115|ref|NC_009085.1| | 1710828 | 1710846 | 19 |
| exprReg_11494 | gi|126640115|ref|NC_009085.1| | 1713942 | 1714026 | 85 |
| exprReg_11495 | gi|126640115|ref|NC_009085.1| | 1714039 | 1714074 | 36 |
| exprReg_11496 | gi|126640115|ref|NC_009085.1| | 1714076 | 1714108 | 33 |
| exprReg_11506 | gi|126640115|ref|NC_009085.1| | 1715294 | 1715340 | 47 |
| exprReg_11507 | gi|126640115|ref|NC_009085.1| | 1715353 | 1715374 | 22 |
| exprReg_11508 | gi|126640115|ref|NC_009085.1| | 1715378 | 1715466 | 89 |
| exprReg_11514 | gi|126640115|ref|NC_009085.1| | 1716771 | 1716824 | 54 |
| exprReg_11547 | gi|126640115|ref|NC_009085.1| | 1720444 | 1720465 | 22 |
| exprReg_11556 | gi|126640115|ref|NC_009085.1| | 1721793 | 1721815 | 23 |
| exprReg_11557 | gi|126640115|ref|NC_009085.1| | 1721821 | 1721916 | 96 |
| exprReg_11564 | gi|126640115|ref|NC_009085.1| | 1722338 | 1722427 | 90 |
| exprReg_11565 | gi|126640115|ref|NC_009085.1| | 1722481 | 1722513 | 33 |
| exprReg_11566 | gi|126640115|ref|NC_009085.1| | 1722530 | 1722591 | 62 |
| exprReg_11567 | gi|126640115|ref|NC_009085.1| | 1722640 | 1722665 | 26 |
| exprReg_11568 | gi|126640115|ref|NC_009085.1| | 1722728 | 1722785 | 58 |
| exprReg_11569 | gi|126640115|ref|NC_009085.1| | 1722851 | 1722867 | 17 |
| exprReg_11573 | gi|126640115|ref|NC_009085.1| | 1723540 | 1723661 | 122 |
| exprReg_11577 | gi|126640115|ref|NC_009085.1| | 1724246 | 1724309 | 64 |
| exprReg_11578 | gi|126640115|ref|NC_009085.1| | 1724486 | 1724659 | 174 |
| exprReg_11582 | gi|126640115|ref|NC_009085.1| | 1725471 | 1725491 | 21 |
| exprReg_11583 | gi|126640115|ref|NC_009085.1| | 1725525 | 1725544 | 20 |
| exprReg_11584 | gi|126640115|ref|NC_009085.1| | 1725554 | 1725576 | 23 |
| exprReg_11585 | gi|126640115|ref|NC_009085.1| | 1725622 | 1725674 | 53 |
| exprReg_11595 | gi|126640115|ref|NC_009085.1| | 1727062 | 1727093 | 32 |
| exprReg_11601 | gi|126640115|ref|NC_009085.1| | 1727853 | 1727880 | 28 |
| exprReg_11602 | gi|126640115|ref|NC_009085.1| | 1727944 | 1727965 | 22 |
| exprReg_11605 | gi|126640115|ref|NC_009085.1| | 1728904 | 1728945 | 42 |
| exprReg_11620 | gi|126640115|ref|NC_009085.1| | 1731885 | 1731951 | 67 |
| exprReg_11621 | gi|126640115|ref|NC_009085.1| | 1732017 | 1732037 | 21 |
| exprReg_11633 | gi|126640115|ref|NC_009085.1| | 1734927 | 1734977 | 51 |
| exprReg_11640 | gi|126640115|ref|NC_009085.1| | 1735778 | 1735842 | 65 |
| exprReg_11641 | gi|126640115|ref|NC_009085.1| | 1736067 | 1736292 | 226 |
| exprReg_11642 | gi|126640115|ref|NC_009085.1| | 1736319 | 1736348 | 30 |
| exprReg_11643 | gi|126640115|ref|NC_009085.1| | 1736351 | 1736369 | 19 |
| exprReg_11644 | gi|126640115|ref|NC_009085.1| | 1736384 | 1736421 | 38 |
| exprReg_11645 | gi|126640115|ref|NC_009085.1| | 1736456 | 1736479 | 24 |
| exprReg_11655 | gi|126640115|ref|NC_009085.1| | 1737261 | 1737284 | 24 |
| exprReg_11668 | gi|126640115|ref|NC_009085.1| | 1738962 | 1739000 | 39 |
| exprReg_11674 | gi|126640115|ref|NC_009085.1| | 1741196 | 1741213 | 18 |
| exprReg_11675 | gi|126640115|ref|NC_009085.1| | 1741314 | 1741335 | 22 |
| exprReg_11676 | gi|126640115|ref|NC_009085.1| | 1741356 | 1741373 | 18 |
| exprReg_11685 | gi|126640115|ref|NC_009085.1| | 1742356 | 1742611 | 256 |
| exprReg_11686 | gi|126640115|ref|NC_009085.1| | 1742620 | 1742675 | 56 |
| exprReg_11696 | gi|126640115|ref|NC_009085.1| | 1743965 | 1744023 | 59 |
| exprReg_11697 | gi|126640115|ref|NC_009085.1| | 1744233 | 1744260 | 28 |
| exprReg_11698 | gi|126640115|ref|NC_009085.1| | 1744284 | 1744384 | 101 |
| exprReg_11699 | gi|126640115|ref|NC_009085.1| | 1744431 | 1744467 | 37 |
| exprReg_11710 | gi|126640115|ref|NC_009085.1| | 1745461 | 1745483 | 23 |
| exprReg_11711 | gi|126640115|ref|NC_009085.1| | 1745492 | 1745749 | 258 |
| exprReg_11712 | gi|126640115|ref|NC_009085.1| | 1745803 | 1745885 | 83 |
| exprReg_11713 | gi|126640115|ref|NC_009085.1| | 1745948 | 1745964 | 17 |
| exprReg_11714 | gi|126640115|ref|NC_009085.1| | 1746022 | 1746058 | 37 |
| exprReg_11718 | gi|126640115|ref|NC_009085.1| | 1746961 | 1747019 | 59 |
| exprReg_11719 | gi|126640115|ref|NC_009085.1| | 1747196 | 1747258 | 63 |
| exprReg_11742 | gi|126640115|ref|NC_009085.1| | 1750485 | 1750520 | 36 |
| exprReg_11743 | gi|126640115|ref|NC_009085.1| | 1750585 | 1750659 | 75 |
| exprReg_11744 | gi|126640115|ref|NC_009085.1| | 1750693 | 1750725 | 33 |
| exprReg_11751 | gi|126640115|ref|NC_009085.1| | 1752252 | 1752296 | 45 |
| exprReg_11755 | gi|126640115|ref|NC_009085.1| | 1753003 | 1753022 | 20 |
| exprReg_11756 | gi|126640115|ref|NC_009085.1| | 1753100 | 1753132 | 33 |
| exprReg_11757 | gi|126640115|ref|NC_009085.1| | 1753347 | 1753395 | 49 |
| exprReg_11758 | gi|126640115|ref|NC_009085.1| | 1753517 | 1753619 | 103 |
| exprReg_11792 | gi|126640115|ref|NC_009085.1| | 1758064 | 1758098 | 35 |
| exprReg_11796 | gi|126640115|ref|NC_009085.1| | 1758666 | 1758683 | 18 |
| exprReg_11797 | gi|126640115|ref|NC_009085.1| | 1758725 | 1758772 | 48 |
| exprReg_11798 | gi|126640115|ref|NC_009085.1| | 1759130 | 1759182 | 53 |
| exprReg_11799 | gi|126640115|ref|NC_009085.1| | 1759339 | 1759367 | 29 |
| exprReg_11800 | gi|126640115|ref|NC_009085.1| | 1759537 | 1759599 | 63 |
| exprReg_11801 | gi|126640115|ref|NC_009085.1| | 1759603 | 1759694 | 92 |
| exprReg_11802 | gi|126640115|ref|NC_009085.1| | 1759702 | 1759765 | 64 |
| exprReg_11803 | gi|126640115|ref|NC_009085.1| | 1759783 | 1759804 | 22 |
| exprReg_11821 | gi|126640115|ref|NC_009085.1| | 1761897 | 1761994 | 98 |
| exprReg_11839 | gi|126640115|ref|NC_009085.1| | 1763965 | 1764045 | 81 |
| exprReg_11840 | gi|126640115|ref|NC_009085.1| | 1764083 | 1764192 | 110 |
| exprReg_11841 | gi|126640115|ref|NC_009085.1| | 1764241 | 1764257 | 17 |
| exprReg_11848 | gi|126640115|ref|NC_009085.1| | 1764902 | 1764955 | 54 |
| exprReg_11853 | gi|126640115|ref|NC_009085.1| | 1765328 | 1765347 | 20 |
| exprReg_11854 | gi|126640115|ref|NC_009085.1| | 1765388 | 1765466 | 79 |
| exprReg_11859 | gi|126640115|ref|NC_009085.1| | 1766436 | 1766554 | 119 |
| exprReg_11860 | gi|126640115|ref|NC_009085.1| | 1766618 | 1766649 | 32 |
| exprReg_11861 | gi|126640115|ref|NC_009085.1| | 1766659 | 1766839 | 181 |
| exprReg_11866 | gi|126640115|ref|NC_009085.1| | 1767255 | 1767276 | 22 |
| exprReg_11867 | gi|126640115|ref|NC_009085.1| | 1767280 | 1767300 | 21 |
| exprReg_11868 | gi|126640115|ref|NC_009085.1| | 1767316 | 1767372 | 57 |
| exprReg_11869 | gi|126640115|ref|NC_009085.1| | 1767375 | 1767398 | 24 |
| exprReg_11870 | gi|126640115|ref|NC_009085.1| | 1767494 | 1767510 | 17 |
| exprReg_11871 | gi|126640115|ref|NC_009085.1| | 1767682 | 1768047 | 366 |
| exprReg_11876 | gi|126640115|ref|NC_009085.1| | 1769027 | 1769352 | 326 |
| exprReg_11877 | gi|126640115|ref|NC_009085.1| | 1769750 | 1769843 | 94 |
| exprReg_11889 | gi|126640115|ref|NC_009085.1| | 1770704 | 1770849 | 146 |
| exprReg_11890 | gi|126640115|ref|NC_009085.1| | 1770942 | 1770962 | 21 |
| exprReg_11891 | gi|126640115|ref|NC_009085.1| | 1770976 | 1770992 | 17 |
| exprReg_11903 | gi|126640115|ref|NC_009085.1| | 1772386 | 1772402 | 17 |
| exprReg_11904 | gi|126640115|ref|NC_009085.1| | 1772444 | 1772460 | 17 |
| exprReg_11913 | gi|126640115|ref|NC_009085.1| | 1773758 | 1773825 | 68 |
| exprReg_11914 | gi|126640115|ref|NC_009085.1| | 1773905 | 1773973 | 69 |
| exprReg_11919 | gi|126640115|ref|NC_009085.1| | 1774949 | 1775027 | 79 |
| exprReg_11929 | gi|126640115|ref|NC_009085.1| | 1776173 | 1776243 | 71 |
| exprReg_11930 | gi|126640115|ref|NC_009085.1| | 1776403 | 1776557 | 155 |
| exprReg_11931 | gi|126640115|ref|NC_009085.1| | 1776650 | 1776717 | 68 |
| exprReg_11976 | gi|126640115|ref|NC_009085.1| | 1781327 | 1781360 | 34 |
| exprReg_11985 | gi|126640115|ref|NC_009085.1| | 1782678 | 1782713 | 36 |
| exprReg_11986 | gi|126640115|ref|NC_009085.1| | 1782720 | 1782781 | 62 |
| exprReg_11987 | gi|126640115|ref|NC_009085.1| | 1782796 | 1782881 | 86 |
| exprReg_11988 | gi|126640115|ref|NC_009085.1| | 1782884 | 1782901 | 18 |
| exprReg_12007 | gi|126640115|ref|NC_009085.1| | 1785315 | 1785370 | 56 |
| exprReg_12008 | gi|126640115|ref|NC_009085.1| | 1785372 | 1785475 | 104 |
| exprReg_12031 | gi|126640115|ref|NC_009085.1| | 1788602 | 1788707 | 106 |
| exprReg_12046 | gi|126640115|ref|NC_009085.1| | 1790539 | 1790609 | 71 |
| exprReg_12047 | gi|126640115|ref|NC_009085.1| | 1790622 | 1790661 | 40 |
| exprReg_12048 | gi|126640115|ref|NC_009085.1| | 1790680 | 1790702 | 23 |
| exprReg_12049 | gi|126640115|ref|NC_009085.1| | 1790845 | 1790863 | 19 |
| exprReg_12050 | gi|126640115|ref|NC_009085.1| | 1791028 | 1791044 | 17 |
| exprReg_12055 | gi|126640115|ref|NC_009085.1| | 1791562 | 1791584 | 23 |
| exprReg_12062 | gi|126640115|ref|NC_009085.1| | 1792401 | 1792419 | 19 |
| exprReg_12063 | gi|126640115|ref|NC_009085.1| | 1792433 | 1792526 | 94 |
| exprReg_12064 | gi|126640115|ref|NC_009085.1| | 1792620 | 1792688 | 69 |
| exprReg_12070 | gi|126640115|ref|NC_009085.1| | 1793843 | 1793884 | 42 |
| exprReg_12071 | gi|126640115|ref|NC_009085.1| | 1793886 | 1793902 | 17 |
| exprReg_12080 | gi|126640115|ref|NC_009085.1| | 1794778 | 1794863 | 86 |
| exprReg_12081 | gi|126640115|ref|NC_009085.1| | 1794892 | 1794963 | 72 |
| exprReg_12082 | gi|126640115|ref|NC_009085.1| | 1795079 | 1795096 | 18 |
| exprReg_12083 | gi|126640115|ref|NC_009085.1| | 1795111 | 1795148 | 38 |
| exprReg_12093 | gi|126640115|ref|NC_009085.1| | 1796355 | 1796412 | 58 |
| exprReg_12094 | gi|126640115|ref|NC_009085.1| | 1796475 | 1796542 | 68 |
| exprReg_12095 | gi|126640115|ref|NC_009085.1| | 1796606 | 1796628 | 23 |
| exprReg_12096 | gi|126640115|ref|NC_009085.1| | 1796633 | 1796675 | 43 |
| exprReg_12132 | gi|126640115|ref|NC_009085.1| | 1801050 | 1801091 | 42 |
| exprReg_12136 | gi|126640115|ref|NC_009085.1| | 1801804 | 1801870 | 67 |
| exprReg_12150 | gi|126640115|ref|NC_009085.1| | 1803495 | 1803578 | 84 |
| exprReg_12158 | gi|126640115|ref|NC_009085.1| | 1804229 | 1804257 | 29 |
| exprReg_12159 | gi|126640115|ref|NC_009085.1| | 1804277 | 1804309 | 33 |
| exprReg_12160 | gi|126640115|ref|NC_009085.1| | 1804312 | 1804329 | 18 |
| exprReg_12169 | gi|126640115|ref|NC_009085.1| | 1805961 | 1806019 | 59 |
| exprReg_12194 | gi|126640115|ref|NC_009085.1| | 1808762 | 1808809 | 48 |
| exprReg_12195 | gi|126640115|ref|NC_009085.1| | 1808812 | 1808830 | 19 |
| exprReg_12196 | gi|126640115|ref|NC_009085.1| | 1808886 | 1808917 | 32 |
| exprReg_12197 | gi|126640115|ref|NC_009085.1| | 1808922 | 1808961 | 40 |
| exprReg_12228 | gi|126640115|ref|NC_009085.1| | 1812695 | 1812716 | 22 |
| exprReg_12229 | gi|126640115|ref|NC_009085.1| | 1812745 | 1812766 | 22 |
| exprReg_12241 | gi|126640115|ref|NC_009085.1| | 1814422 | 1814439 | 18 |
| exprReg_12282 | gi|126640115|ref|NC_009085.1| | 1820573 | 1820656 | 84 |
| exprReg_12290 | gi|126640115|ref|NC_009085.1| | 1822175 | 1822204 | 30 |
| exprReg_12296 | gi|126640115|ref|NC_009085.1| | 1822649 | 1822672 | 24 |
| exprReg_12298 | gi|126640115|ref|NC_009085.1| | 1823001 | 1823019 | 19 |
| exprReg_12299 | gi|126640115|ref|NC_009085.1| | 1823065 | 1823090 | 26 |
| exprReg_12322 | gi|126640115|ref|NC_009085.1| | 1826210 | 1826306 | 97 |
| exprReg_12323 | gi|126640115|ref|NC_009085.1| | 1826363 | 1826385 | 23 |
| exprReg_12324 | gi|126640115|ref|NC_009085.1| | 1826425 | 1826441 | 17 |
| exprReg_12325 | gi|126640115|ref|NC_009085.1| | 1826444 | 1826462 | 19 |
| exprReg_12326 | gi|126640115|ref|NC_009085.1| | 1826497 | 1826590 | 94 |
| exprReg_12335 | gi|126640115|ref|NC_009085.1| | 1827484 | 1827502 | 19 |
| exprReg_12336 | gi|126640115|ref|NC_009085.1| | 1827590 | 1827621 | 32 |
| exprReg_12344 | gi|126640115|ref|NC_009085.1| | 1828823 | 1828840 | 18 |
| exprReg_12345 | gi|126640115|ref|NC_009085.1| | 1828844 | 1828896 | 53 |
| exprReg_12346 | gi|126640115|ref|NC_009085.1| | 1829023 | 1829083 | 61 |
| exprReg_12347 | gi|126640115|ref|NC_009085.1| | 1829205 | 1829221 | 17 |
| exprReg_12348 | gi|126640115|ref|NC_009085.1| | 1829279 | 1829296 | 18 |
| exprReg_12351 | gi|126640115|ref|NC_009085.1| | 1829905 | 1830055 | 151 |
| exprReg_12352 | gi|126640115|ref|NC_009085.1| | 1830101 | 1830136 | 36 |
| exprReg_12353 | gi|126640115|ref|NC_009085.1| | 1830226 | 1830248 | 23 |
| exprReg_12355 | gi|126640115|ref|NC_009085.1| | 1830781 | 1830918 | 138 |
| exprReg_12356 | gi|126640115|ref|NC_009085.1| | 1831014 | 1831091 | 78 |
| exprReg_12357 | gi|126640115|ref|NC_009085.1| | 1831120 | 1831339 | 220 |
| exprReg_12358 | gi|126640115|ref|NC_009085.1| | 1831432 | 1831539 | 108 |
| exprReg_12359 | gi|126640115|ref|NC_009085.1| | 1831637 | 1831656 | 20 |
| exprReg_12360 | gi|126640115|ref|NC_009085.1| | 1831747 | 1831782 | 36 |
| exprReg_12361 | gi|126640115|ref|NC_009085.1| | 1831821 | 1831950 | 130 |
| exprReg_12362 | gi|126640115|ref|NC_009085.1| | 1831965 | 1832069 | 105 |
| exprReg_12363 | gi|126640115|ref|NC_009085.1| | 1832158 | 1832181 | 24 |
| exprReg_12364 | gi|126640115|ref|NC_009085.1| | 1832315 | 1832368 | 54 |
| exprReg_12365 | gi|126640115|ref|NC_009085.1| | 1832667 | 1832706 | 40 |
| exprReg_12366 | gi|126640115|ref|NC_009085.1| | 1832765 | 1832781 | 17 |
| exprReg_12367 | gi|126640115|ref|NC_009085.1| | 1832808 | 1832931 | 124 |
| exprReg_12368 | gi|126640115|ref|NC_009085.1| | 1832947 | 1833031 | 85 |
| exprReg_12369 | gi|126640115|ref|NC_009085.1| | 1833130 | 1833159 | 30 |
| exprReg_12370 | gi|126640115|ref|NC_009085.1| | 1833173 | 1833196 | 24 |
| exprReg_12371 | gi|126640115|ref|NC_009085.1| | 1833214 | 1833261 | 48 |
| exprReg_12372 | gi|126640115|ref|NC_009085.1| | 1833378 | 1833397 | 20 |
| exprReg_12376 | gi|126640115|ref|NC_009085.1| | 1834035 | 1834097 | 63 |
| exprReg_12377 | gi|126640115|ref|NC_009085.1| | 1834255 | 1834351 | 97 |
| exprReg_12386 | gi|126640115|ref|NC_009085.1| | 1837132 | 1837323 | 192 |
| exprReg_12387 | gi|126640115|ref|NC_009085.1| | 1837431 | 1837524 | 94 |
| exprReg_12388 | gi|126640115|ref|NC_009085.1| | 1837571 | 1837587 | 17 |
| exprReg_12389 | gi|126640115|ref|NC_009085.1| | 1837663 | 1837695 | 33 |
| exprReg_12390 | gi|126640115|ref|NC_009085.1| | 1837741 | 1838257 | 517 |
| exprReg_12391 | gi|126640115|ref|NC_009085.1| | 1838502 | 1838726 | 225 |
| exprReg_12392 | gi|126640115|ref|NC_009085.1| | 1838799 | 1838826 | 28 |
| exprReg_12393 | gi|126640115|ref|NC_009085.1| | 1838947 | 1838964 | 18 |
| exprReg_12394 | gi|126640115|ref|NC_009085.1| | 1839012 | 1839185 | 174 |
| exprReg_12395 | gi|126640115|ref|NC_009085.1| | 1839287 | 1839311 | 25 |
| exprReg_12396 | gi|126640115|ref|NC_009085.1| | 1839362 | 1839420 | 59 |
| exprReg_12397 | gi|126640115|ref|NC_009085.1| | 1839589 | 1839613 | 25 |
| exprReg_12398 | gi|126640115|ref|NC_009085.1| | 1839640 | 1839704 | 65 |
| exprReg_12399 | gi|126640115|ref|NC_009085.1| | 1839873 | 1839936 | 64 |
| exprReg_12400 | gi|126640115|ref|NC_009085.1| | 1839980 | 1840054 | 75 |
| exprReg_12401 | gi|126640115|ref|NC_009085.1| | 1840182 | 1840198 | 17 |
| exprReg_12402 | gi|126640115|ref|NC_009085.1| | 1840268 | 1840314 | 47 |
| exprReg_12403 | gi|126640115|ref|NC_009085.1| | 1840318 | 1840391 | 74 |
| exprReg_12404 | gi|126640115|ref|NC_009085.1| | 1840435 | 1840451 | 17 |
| exprReg_12405 | gi|126640115|ref|NC_009085.1| | 1840722 | 1840866 | 145 |
| exprReg_12406 | gi|126640115|ref|NC_009085.1| | 1840898 | 1841013 | 116 |
| exprReg_12407 | gi|126640115|ref|NC_009085.1| | 1841099 | 1841136 | 38 |
| exprReg_12408 | gi|126640115|ref|NC_009085.1| | 1841259 | 1841332 | 74 |
| exprReg_12409 | gi|126640115|ref|NC_009085.1| | 1841532 | 1841587 | 56 |
| exprReg_12410 | gi|126640115|ref|NC_009085.1| | 1841600 | 1841679 | 80 |
| exprReg_12411 | gi|126640115|ref|NC_009085.1| | 1841794 | 1841829 | 36 |
| exprReg_12412 | gi|126640115|ref|NC_009085.1| | 1841858 | 1841910 | 53 |
| exprReg_12413 | gi|126640115|ref|NC_009085.1| | 1842016 | 1842110 | 95 |
| exprReg_12414 | gi|126640115|ref|NC_009085.1| | 1842128 | 1842198 | 71 |
| exprReg_12415 | gi|126640115|ref|NC_009085.1| | 1842231 | 1842266 | 36 |
| exprReg_12420 | gi|126640115|ref|NC_009085.1| | 1842740 | 1842757 | 18 |
| exprReg_12421 | gi|126640115|ref|NC_009085.1| | 1842818 | 1842870 | 53 |
| exprReg_12422 | gi|126640115|ref|NC_009085.1| | 1843089 | 1843118 | 30 |
| exprReg_12423 | gi|126640115|ref|NC_009085.1| | 1843247 | 1843277 | 31 |
| exprReg_12425 | gi|126640115|ref|NC_009085.1| | 1844117 | 1844240 | 124 |
| exprReg_12432 | gi|126640115|ref|NC_009085.1| | 1845390 | 1845490 | 101 |
| exprReg_12448 | gi|126640115|ref|NC_009085.1| | 1848565 | 1848637 | 73 |
| exprReg_12449 | gi|126640115|ref|NC_009085.1| | 1848666 | 1848730 | 65 |
| exprReg_12459 | gi|126640115|ref|NC_009085.1| | 1850562 | 1850578 | 17 |
| exprReg_12460 | gi|126640115|ref|NC_009085.1| | 1850622 | 1850875 | 254 |
| exprReg_12461 | gi|126640115|ref|NC_009085.1| | 1850939 | 1851036 | 98 |
| exprReg_12462 | gi|126640115|ref|NC_009085.1| | 1851194 | 1851320 | 127 |
| exprReg_12463 | gi|126640115|ref|NC_009085.1| | 1851341 | 1851514 | 174 |
| exprReg_12464 | gi|126640115|ref|NC_009085.1| | 1851676 | 1851694 | 19 |
| exprReg_12465 | gi|126640115|ref|NC_009085.1| | 1851764 | 1851851 | 88 |
| exprReg_12490 | gi|126640115|ref|NC_009085.1| | 1855552 | 1855693 | 142 |
| exprReg_12491 | gi|126640115|ref|NC_009085.1| | 1855735 | 1855855 | 121 |
| exprReg_12492 | gi|126640115|ref|NC_009085.1| | 1855927 | 1855943 | 17 |
| exprReg_12493 | gi|126640115|ref|NC_009085.1| | 1856067 | 1856109 | 43 |
| exprReg_12494 | gi|126640115|ref|NC_009085.1| | 1856148 | 1856166 | 19 |
| exprReg_12495 | gi|126640115|ref|NC_009085.1| | 1856243 | 1856321 | 79 |
| exprReg_12496 | gi|126640115|ref|NC_009085.1| | 1856441 | 1856475 | 35 |
| exprReg_12497 | gi|126640115|ref|NC_009085.1| | 1856488 | 1856524 | 37 |
| exprReg_12498 | gi|126640115|ref|NC_009085.1| | 1856585 | 1856616 | 32 |
| exprReg_12499 | gi|126640115|ref|NC_009085.1| | 1856674 | 1856699 | 26 |
| exprReg_12522 | gi|126640115|ref|NC_009085.1| | 1859487 | 1859505 | 19 |
| exprReg_12531 | gi|126640115|ref|NC_009085.1| | 1861628 | 1861645 | 18 |
| exprReg_12532 | gi|126640115|ref|NC_009085.1| | 1861715 | 1861788 | 74 |
| exprReg_12533 | gi|126640115|ref|NC_009085.1| | 1861835 | 1861884 | 50 |
| exprReg_12534 | gi|126640115|ref|NC_009085.1| | 1862286 | 1862448 | 163 |
| exprReg_12539 | gi|126640115|ref|NC_009085.1| | 1863193 | 1863326 | 134 |
| exprReg_12540 | gi|126640115|ref|NC_009085.1| | 1863411 | 1863510 | 100 |
| exprReg_12541 | gi|126640115|ref|NC_009085.1| | 1863748 | 1863781 | 34 |
| exprReg_12542 | gi|126640115|ref|NC_009085.1| | 1863945 | 1863975 | 31 |
| exprReg_12543 | gi|126640115|ref|NC_009085.1| | 1864078 | 1864140 | 63 |
| exprReg_12544 | gi|126640115|ref|NC_009085.1| | 1864152 | 1864175 | 24 |
| exprReg_12545 | gi|126640115|ref|NC_009085.1| | 1864198 | 1864224 | 27 |
| exprReg_12546 | gi|126640115|ref|NC_009085.1| | 1864230 | 1864250 | 21 |
| exprReg_12547 | gi|126640115|ref|NC_009085.1| | 1864270 | 1864294 | 25 |
| exprReg_12548 | gi|126640115|ref|NC_009085.1| | 1864326 | 1864342 | 17 |
| exprReg_12564 | gi|126640115|ref|NC_009085.1| | 1866444 | 1866484 | 41 |
| exprReg_12565 | gi|126640115|ref|NC_009085.1| | 1866543 | 1866608 | 66 |
| exprReg_12579 | gi|126640115|ref|NC_009085.1| | 1868552 | 1868665 | 114 |
| exprReg_12580 | gi|126640115|ref|NC_009085.1| | 1868921 | 1869065 | 145 |
| exprReg_12581 | gi|126640115|ref|NC_009085.1| | 1869070 | 1869117 | 48 |
| exprReg_12582 | gi|126640115|ref|NC_009085.1| | 1869136 | 1869171 | 36 |
| exprReg_12583 | gi|126640115|ref|NC_009085.1| | 1869308 | 1869455 | 148 |
| exprReg_12584 | gi|126640115|ref|NC_009085.1| | 1869457 | 1869482 | 26 |
| exprReg_12590 | gi|126640115|ref|NC_009085.1| | 1870142 | 1870176 | 35 |
| exprReg_12604 | gi|126640115|ref|NC_009085.1| | 1872979 | 1873001 | 23 |
| exprReg_12605 | gi|126640115|ref|NC_009085.1| | 1873080 | 1873171 | 92 |
| exprReg_12619 | gi|126640115|ref|NC_009085.1| | 1874934 | 1875076 | 143 |
| exprReg_12620 | gi|126640115|ref|NC_009085.1| | 1875079 | 1875097 | 19 |
| exprReg_12648 | gi|126640115|ref|NC_009085.1| | 1878745 | 1878774 | 30 |
| exprReg_12649 | gi|126640115|ref|NC_009085.1| | 1878790 | 1878814 | 25 |
| exprReg_12664 | gi|126640115|ref|NC_009085.1| | 1880908 | 1880929 | 22 |
| exprReg_12665 | gi|126640115|ref|NC_009085.1| | 1880982 | 1881002 | 21 |
| exprReg_12684 | gi|126640115|ref|NC_009085.1| | 1883356 | 1883408 | 53 |
| exprReg_12685 | gi|126640115|ref|NC_009085.1| | 1883423 | 1883453 | 31 |
| exprReg_12686 | gi|126640115|ref|NC_009085.1| | 1883575 | 1883804 | 230 |
| exprReg_12687 | gi|126640115|ref|NC_009085.1| | 1883954 | 1884110 | 157 |
| exprReg_12688 | gi|126640115|ref|NC_009085.1| | 1884116 | 1884151 | 36 |
| exprReg_12689 | gi|126640115|ref|NC_009085.1| | 1884299 | 1884343 | 45 |
| exprReg_12703 | gi|126640115|ref|NC_009085.1| | 1885756 | 1885788 | 33 |
| exprReg_12706 | gi|126640115|ref|NC_009085.1| | 1886082 | 1886176 | 95 |
| exprReg_12707 | gi|126640115|ref|NC_009085.1| | 1886270 | 1886326 | 57 |
| exprReg_12708 | gi|126640115|ref|NC_009085.1| | 1886377 | 1886423 | 47 |
| exprReg_12715 | gi|126640115|ref|NC_009085.1| | 1887535 | 1887624 | 90 |
| exprReg_12723 | gi|126640115|ref|NC_009085.1| | 1888421 | 1888483 | 63 |
| exprReg_12732 | gi|126640115|ref|NC_009085.1| | 1889811 | 1889881 | 71 |
| exprReg_12733 | gi|126640115|ref|NC_009085.1| | 1889960 | 1890058 | 99 |
| exprReg_12756 | gi|126640115|ref|NC_009085.1| | 1893485 | 1893502 | 18 |
| exprReg_12757 | gi|126640115|ref|NC_009085.1| | 1893571 | 1893605 | 35 |
| exprReg_12787 | gi|126640115|ref|NC_009085.1| | 1897607 | 1897634 | 28 |
| exprReg_12788 | gi|126640115|ref|NC_009085.1| | 1897655 | 1897671 | 17 |
| exprReg_12789 | gi|126640115|ref|NC_009085.1| | 1897676 | 1897696 | 21 |
| exprReg_12790 | gi|126640115|ref|NC_009085.1| | 1897706 | 1897724 | 19 |
| exprReg_12819 | gi|126640115|ref|NC_009085.1| | 1901620 | 1901842 | 223 |
| exprReg_12827 | gi|126640115|ref|NC_009085.1| | 1902947 | 1902979 | 33 |
| exprReg_12828 | gi|126640115|ref|NC_009085.1| | 1903009 | 1903056 | 48 |
| exprReg_12829 | gi|126640115|ref|NC_009085.1| | 1903069 | 1903085 | 17 |
| exprReg_12830 | gi|126640115|ref|NC_009085.1| | 1903124 | 1903163 | 40 |
| exprReg_12841 | gi|126640115|ref|NC_009085.1| | 1904177 | 1904193 | 17 |
| exprReg_12851 | gi|126640115|ref|NC_009085.1| | 1905679 | 1905697 | 19 |
| exprReg_12852 | gi|126640115|ref|NC_009085.1| | 1905774 | 1905793 | 20 |
| exprReg_12853 | gi|126640115|ref|NC_009085.1| | 1905795 | 1905835 | 41 |
| exprReg_12854 | gi|126640115|ref|NC_009085.1| | 1905848 | 1905877 | 30 |
| exprReg_12855 | gi|126640115|ref|NC_009085.1| | 1905929 | 1905952 | 24 |
| exprReg_12866 | gi|126640115|ref|NC_009085.1| | 1907219 | 1907265 | 47 |
| exprReg_12867 | gi|126640115|ref|NC_009085.1| | 1907284 | 1907307 | 24 |
| exprReg_12878 | gi|126640115|ref|NC_009085.1| | 1908448 | 1908520 | 73 |
| exprReg_12885 | gi|126640115|ref|NC_009085.1| | 1909642 | 1909784 | 143 |
| exprReg_12886 | gi|126640115|ref|NC_009085.1| | 1909799 | 1909940 | 142 |
| exprReg_12898 | gi|126640115|ref|NC_009085.1| | 1911336 | 1911354 | 19 |
| exprReg_12899 | gi|126640115|ref|NC_009085.1| | 1911374 | 1911509 | 136 |
| exprReg_12900 | gi|126640115|ref|NC_009085.1| | 1911610 | 1911645 | 36 |
| exprReg_12905 | gi|126640115|ref|NC_009085.1| | 1912008 | 1912049 | 42 |
| exprReg_12906 | gi|126640115|ref|NC_009085.1| | 1912089 | 1912106 | 18 |
| exprReg_12907 | gi|126640115|ref|NC_009085.1| | 1912212 | 1912228 | 17 |
| exprReg_12910 | gi|126640115|ref|NC_009085.1| | 1912651 | 1912690 | 40 |
| exprReg_12911 | gi|126640115|ref|NC_009085.1| | 1912903 | 1913013 | 111 |
| exprReg_12930 | gi|126640115|ref|NC_009085.1| | 1914817 | 1914857 | 41 |
| exprReg_12988 | gi|126640115|ref|NC_009085.1| | 1921468 | 1921514 | 47 |
| exprReg_12992 | gi|126640115|ref|NC_009085.1| | 1922274 | 1922361 | 88 |
| exprReg_13015 | gi|126640115|ref|NC_009085.1| | 1925395 | 1925412 | 18 |
| exprReg_13016 | gi|126640115|ref|NC_009085.1| | 1925452 | 1925509 | 58 |
| exprReg_13017 | gi|126640115|ref|NC_009085.1| | 1925633 | 1925649 | 17 |
| exprReg_13018 | gi|126640115|ref|NC_009085.1| | 1925739 | 1925786 | 48 |
| exprReg_13031 | gi|126640115|ref|NC_009085.1| | 1927341 | 1927358 | 18 |
| exprReg_13032 | gi|126640115|ref|NC_009085.1| | 1927615 | 1927634 | 20 |
| exprReg_13033 | gi|126640115|ref|NC_009085.1| | 1927668 | 1927689 | 22 |
| exprReg_13053 | gi|126640115|ref|NC_009085.1| | 1930787 | 1930856 | 70 |
| exprReg_13054 | gi|126640115|ref|NC_009085.1| | 1930863 | 1930879 | 17 |
| exprReg_13055 | gi|126640115|ref|NC_009085.1| | 1931104 | 1931120 | 17 |
| exprReg_13056 | gi|126640115|ref|NC_009085.1| | 1931208 | 1931226 | 19 |
| exprReg_13057 | gi|126640115|ref|NC_009085.1| | 1931314 | 1931397 | 84 |
| exprReg_13058 | gi|126640115|ref|NC_009085.1| | 1931412 | 1931430 | 19 |
| exprReg_13063 | gi|126640115|ref|NC_009085.1| | 1931990 | 1932011 | 22 |
| exprReg_13064 | gi|126640115|ref|NC_009085.1| | 1932027 | 1932112 | 86 |
| exprReg_13065 | gi|126640115|ref|NC_009085.1| | 1932162 | 1932182 | 21 |
| exprReg_13073 | gi|126640115|ref|NC_009085.1| | 1933022 | 1933043 | 22 |
| exprReg_13077 | gi|126640115|ref|NC_009085.1| | 1933701 | 1933767 | 67 |
| exprReg_13078 | gi|126640115|ref|NC_009085.1| | 1933799 | 1933855 | 57 |
| exprReg_13087 | gi|126640115|ref|NC_009085.1| | 1935459 | 1935512 | 54 |
| exprReg_13093 | gi|126640115|ref|NC_009085.1| | 1936174 | 1936192 | 19 |
| exprReg_13107 | gi|126640115|ref|NC_009085.1| | 1938252 | 1938275 | 24 |
| exprReg_13108 | gi|126640115|ref|NC_009085.1| | 1938306 | 1938343 | 38 |
| exprReg_13138 | gi|126640115|ref|NC_009085.1| | 1942702 | 1942724 | 23 |
| exprReg_13139 | gi|126640115|ref|NC_009085.1| | 1942907 | 1942957 | 51 |
| exprReg_13140 | gi|126640115|ref|NC_009085.1| | 1942959 | 1942996 | 38 |
| exprReg_13151 | gi|126640115|ref|NC_009085.1| | 1944651 | 1944672 | 22 |
| exprReg_13152 | gi|126640115|ref|NC_009085.1| | 1944680 | 1944702 | 23 |
| exprReg_13153 | gi|126640115|ref|NC_009085.1| | 1944728 | 1944802 | 75 |
| exprReg_13188 | gi|126640115|ref|NC_009085.1| | 1950633 | 1950764 | 132 |
| exprReg_13189 | gi|126640115|ref|NC_009085.1| | 1950813 | 1950832 | 20 |
| exprReg_13190 | gi|126640115|ref|NC_009085.1| | 1950838 | 1950866 | 29 |
| exprReg_13191 | gi|126640115|ref|NC_009085.1| | 1950869 | 1950906 | 38 |
| exprReg_13194 | gi|126640115|ref|NC_009085.1| | 1951466 | 1951493 | 28 |
| exprReg_13195 | gi|126640115|ref|NC_009085.1| | 1951528 | 1951551 | 24 |
| exprReg_13198 | gi|126640115|ref|NC_009085.1| | 1951938 | 1951955 | 18 |
| exprReg_13199 | gi|126640115|ref|NC_009085.1| | 1951979 | 1951997 | 19 |
| exprReg_13200 | gi|126640115|ref|NC_009085.1| | 1952134 | 1952173 | 40 |
| exprReg_13208 | gi|126640115|ref|NC_009085.1| | 1953264 | 1953329 | 66 |
| exprReg_13209 | gi|126640115|ref|NC_009085.1| | 1953423 | 1953474 | 52 |
| exprReg_13212 | gi|126640115|ref|NC_009085.1| | 1954515 | 1954534 | 20 |
| exprReg_13213 | gi|126640115|ref|NC_009085.1| | 1954620 | 1954641 | 22 |
| exprReg_13214 | gi|126640115|ref|NC_009085.1| | 1954685 | 1954758 | 74 |
| exprReg_13215 | gi|126640115|ref|NC_009085.1| | 1954764 | 1954806 | 43 |
| exprReg_13216 | gi|126640115|ref|NC_009085.1| | 1954866 | 1954907 | 42 |
| exprReg_13217 | gi|126640115|ref|NC_009085.1| | 1954990 | 1955006 | 17 |
| exprReg_13220 | gi|126640115|ref|NC_009085.1| | 1955323 | 1955415 | 93 |
| exprReg_13221 | gi|126640115|ref|NC_009085.1| | 1955881 | 1955898 | 18 |
| exprReg_13222 | gi|126640115|ref|NC_009085.1| | 1956095 | 1956131 | 37 |
| exprReg_13223 | gi|126640115|ref|NC_009085.1| | 1956277 | 1956301 | 25 |
| exprReg_13224 | gi|126640115|ref|NC_009085.1| | 1956323 | 1956395 | 73 |
| exprReg_13225 | gi|126640115|ref|NC_009085.1| | 1956442 | 1956473 | 32 |
| exprReg_13226 | gi|126640115|ref|NC_009085.1| | 1956488 | 1956507 | 20 |
| exprReg_13227 | gi|126640115|ref|NC_009085.1| | 1956874 | 1956899 | 26 |
| exprReg_13236 | gi|126640115|ref|NC_009085.1| | 1958477 | 1958496 | 20 |
| exprReg_13237 | gi|126640115|ref|NC_009085.1| | 1958605 | 1958622 | 18 |
| exprReg_13238 | gi|126640115|ref|NC_009085.1| | 1958766 | 1958817 | 52 |
| exprReg_13239 | gi|126640115|ref|NC_009085.1| | 1958827 | 1958863 | 37 |
| exprReg_13248 | gi|126640115|ref|NC_009085.1| | 1960080 | 1960163 | 84 |
| exprReg_13249 | gi|126640115|ref|NC_009085.1| | 1960184 | 1960219 | 36 |
| exprReg_13250 | gi|126640115|ref|NC_009085.1| | 1960223 | 1960274 | 52 |
| exprReg_13251 | gi|126640115|ref|NC_009085.1| | 1960289 | 1960372 | 84 |
| exprReg_13252 | gi|126640115|ref|NC_009085.1| | 1960380 | 1960396 | 17 |
| exprReg_13253 | gi|126640115|ref|NC_009085.1| | 1960428 | 1960459 | 32 |
| exprReg_13254 | gi|126640115|ref|NC_009085.1| | 1960491 | 1960563 | 73 |
| exprReg_13255 | gi|126640115|ref|NC_009085.1| | 1960570 | 1960590 | 21 |
| exprReg_13260 | gi|126640115|ref|NC_009085.1| | 1960994 | 1961029 | 36 |
| exprReg_13266 | gi|126640115|ref|NC_009085.1| | 1961732 | 1961843 | 112 |
| exprReg_13267 | gi|126640115|ref|NC_009085.1| | 1961901 | 1961917 | 17 |
| exprReg_13274 | gi|126640115|ref|NC_009085.1| | 1963102 | 1963147 | 46 |
| exprReg_13275 | gi|126640115|ref|NC_009085.1| | 1963239 | 1963263 | 25 |
| exprReg_13276 | gi|126640115|ref|NC_009085.1| | 1963320 | 1963418 | 99 |
| exprReg_13277 | gi|126640115|ref|NC_009085.1| | 1963470 | 1963722 | 253 |
| exprReg_13278 | gi|126640115|ref|NC_009085.1| | 1963734 | 1963781 | 48 |
| exprReg_13279 | gi|126640115|ref|NC_009085.1| | 1963910 | 1963943 | 34 |
| exprReg_13280 | gi|126640115|ref|NC_009085.1| | 1963957 | 1963992 | 36 |
| exprReg_13281 | gi|126640115|ref|NC_009085.1| | 1963997 | 1964135 | 139 |
| exprReg_13282 | gi|126640115|ref|NC_009085.1| | 1964166 | 1964197 | 32 |
| exprReg_13285 | gi|126640115|ref|NC_009085.1| | 1964569 | 1964588 | 20 |
| exprReg_13286 | gi|126640115|ref|NC_009085.1| | 1964594 | 1964612 | 19 |
| exprReg_13294 | gi|126640115|ref|NC_009085.1| | 1965650 | 1965734 | 85 |
| exprReg_13305 | gi|126640115|ref|NC_009085.1| | 1966647 | 1966673 | 27 |
| exprReg_13322 | gi|126640115|ref|NC_009085.1| | 1969066 | 1969103 | 38 |
| exprReg_13323 | gi|126640115|ref|NC_009085.1| | 1969141 | 1969224 | 84 |
| exprReg_13324 | gi|126640115|ref|NC_009085.1| | 1969268 | 1969319 | 52 |
| exprReg_13325 | gi|126640115|ref|NC_009085.1| | 1969373 | 1969389 | 17 |
| exprReg_13347 | gi|126640115|ref|NC_009085.1| | 1972796 | 1972944 | 149 |
| exprReg_13361 | gi|126640115|ref|NC_009085.1| | 1974907 | 1975025 | 119 |
| exprReg_13371 | gi|126640115|ref|NC_009085.1| | 1975812 | 1975832 | 21 |
| exprReg_13372 | gi|126640115|ref|NC_009085.1| | 1976376 | 1976443 | 68 |
| exprReg_13373 | gi|126640115|ref|NC_009085.1| | 1976472 | 1976621 | 150 |
| exprReg_13390 | gi|126640115|ref|NC_009085.1| | 1979964 | 1980056 | 93 |
| exprReg_13401 | gi|126640115|ref|NC_009085.1| | 1981051 | 1981120 | 70 |
| exprReg_13410 | gi|126640115|ref|NC_009085.1| | 1982243 | 1982291 | 49 |
| exprReg_13418 | gi|126640115|ref|NC_009085.1| | 1983264 | 1983281 | 18 |
| exprReg_13422 | gi|126640115|ref|NC_009085.1| | 1984222 | 1984238 | 17 |
| exprReg_13431 | gi|126640115|ref|NC_009085.1| | 1985426 | 1985460 | 35 |
| exprReg_13436 | gi|126640115|ref|NC_009085.1| | 1986696 | 1986718 | 23 |
| exprReg_13460 | gi|126640115|ref|NC_009085.1| | 1990999 | 1991037 | 39 |
| exprReg_13461 | gi|126640115|ref|NC_009085.1| | 1991270 | 1991286 | 17 |
| exprReg_13462 | gi|126640115|ref|NC_009085.1| | 1991301 | 1991328 | 28 |
| exprReg_13475 | gi|126640115|ref|NC_009085.1| | 1992558 | 1992676 | 119 |
| exprReg_13476 | gi|126640115|ref|NC_009085.1| | 1992678 | 1992700 | 23 |
| exprReg_13477 | gi|126640115|ref|NC_009085.1| | 1992713 | 1992730 | 18 |
| exprReg_13491 | gi|126640115|ref|NC_009085.1| | 1994863 | 1994922 | 60 |
| exprReg_13492 | gi|126640115|ref|NC_009085.1| | 1995027 | 1995044 | 18 |
| exprReg_13506 | gi|126640115|ref|NC_009085.1| | 1997697 | 1997746 | 50 |
| exprReg_13517 | gi|126640115|ref|NC_009085.1| | 2000237 | 2000258 | 22 |
| exprReg_13526 | gi|126640115|ref|NC_009085.1| | 2001892 | 2001972 | 81 |
| exprReg_13536 | gi|126640115|ref|NC_009085.1| | 2003916 | 2003938 | 23 |
| exprReg_13537 | gi|126640115|ref|NC_009085.1| | 2003979 | 2004020 | 42 |
| exprReg_13568 | gi|126640115|ref|NC_009085.1| | 2008072 | 2008148 | 77 |
| exprReg_13569 | gi|126640115|ref|NC_009085.1| | 2008199 | 2008233 | 35 |
| exprReg_13570 | gi|126640115|ref|NC_009085.1| | 2008238 | 2008268 | 31 |
| exprReg_13571 | gi|126640115|ref|NC_009085.1| | 2008352 | 2008531 | 180 |
| exprReg_13572 | gi|126640115|ref|NC_009085.1| | 2008580 | 2008617 | 38 |
| exprReg_13573 | gi|126640115|ref|NC_009085.1| | 2008712 | 2008787 | 76 |
| exprReg_13574 | gi|126640115|ref|NC_009085.1| | 2009218 | 2009240 | 23 |
| exprReg_13582 | gi|126640115|ref|NC_009085.1| | 2010282 | 2010350 | 69 |
| exprReg_13583 | gi|126640115|ref|NC_009085.1| | 2010370 | 2010512 | 143 |
| exprReg_13598 | gi|126640115|ref|NC_009085.1| | 2012622 | 2012662 | 41 |
| exprReg_13621 | gi|126640115|ref|NC_009085.1| | 2015329 | 2015364 | 36 |
| exprReg_13622 | gi|126640115|ref|NC_009085.1| | 2015372 | 2015388 | 17 |
| exprReg_13623 | gi|126640115|ref|NC_009085.1| | 2015414 | 2015436 | 23 |
| exprReg_13624 | gi|126640115|ref|NC_009085.1| | 2015438 | 2015476 | 39 |
| exprReg_13625 | gi|126640115|ref|NC_009085.1| | 2015479 | 2015561 | 83 |
| exprReg_13631 | gi|126640115|ref|NC_009085.1| | 2016553 | 2016569 | 17 |
| exprReg_13632 | gi|126640115|ref|NC_009085.1| | 2016818 | 2016834 | 17 |
| exprReg_13633 | gi|126640115|ref|NC_009085.1| | 2016901 | 2017033 | 133 |
| exprReg_13634 | gi|126640115|ref|NC_009085.1| | 2017056 | 2017107 | 52 |
| exprReg_13635 | gi|126640115|ref|NC_009085.1| | 2017112 | 2017134 | 23 |
| exprReg_13636 | gi|126640115|ref|NC_009085.1| | 2017172 | 2017229 | 58 |
| exprReg_13637 | gi|126640115|ref|NC_009085.1| | 2017423 | 2017456 | 34 |
| exprReg_13638 | gi|126640115|ref|NC_009085.1| | 2017488 | 2017504 | 17 |
| exprReg_13642 | gi|126640115|ref|NC_009085.1| | 2018446 | 2018468 | 23 |
| exprReg_13645 | gi|126640115|ref|NC_009085.1| | 2019069 | 2019287 | 219 |
| exprReg_13646 | gi|126640115|ref|NC_009085.1| | 2019409 | 2019426 | 18 |
| exprReg_13647 | gi|126640115|ref|NC_009085.1| | 2019468 | 2019486 | 19 |
| exprReg_13648 | gi|126640115|ref|NC_009085.1| | 2019520 | 2019566 | 47 |
| exprReg_13649 | gi|126640115|ref|NC_009085.1| | 2019682 | 2019707 | 26 |
| exprReg_13650 | gi|126640115|ref|NC_009085.1| | 2019739 | 2019820 | 82 |
| exprReg_13651 | gi|126640115|ref|NC_009085.1| | 2019829 | 2019854 | 26 |
| exprReg_13652 | gi|126640115|ref|NC_009085.1| | 2019917 | 2019978 | 62 |
| exprReg_13653 | gi|126640115|ref|NC_009085.1| | 2020010 | 2020158 | 149 |
| exprReg_13654 | gi|126640115|ref|NC_009085.1| | 2020414 | 2020490 | 77 |
| exprReg_13655 | gi|126640115|ref|NC_009085.1| | 2020571 | 2020587 | 17 |
| exprReg_13666 | gi|126640115|ref|NC_009085.1| | 2021993 | 2022023 | 31 |
| exprReg_13667 | gi|126640115|ref|NC_009085.1| | 2022034 | 2022093 | 60 |
| exprReg_13672 | gi|126640115|ref|NC_009085.1| | 2022987 | 2023066 | 80 |
| exprReg_13678 | gi|126640115|ref|NC_009085.1| | 2024827 | 2024847 | 21 |
| exprReg_13679 | gi|126640115|ref|NC_009085.1| | 2024884 | 2024900 | 17 |
| exprReg_13683 | gi|126640115|ref|NC_009085.1| | 2025559 | 2025577 | 19 |
| exprReg_13684 | gi|126640115|ref|NC_009085.1| | 2025869 | 2025897 | 29 |
| exprReg_13713 | gi|126640115|ref|NC_009085.1| | 2031723 | 2031742 | 20 |
| exprReg_13714 | gi|126640115|ref|NC_009085.1| | 2031807 | 2032003 | 197 |
| exprReg_13715 | gi|126640115|ref|NC_009085.1| | 2032034 | 2032050 | 17 |
| exprReg_13716 | gi|126640115|ref|NC_009085.1| | 2032063 | 2032122 | 60 |
| exprReg_13717 | gi|126640115|ref|NC_009085.1| | 2032126 | 2032148 | 23 |
| exprReg_13718 | gi|126640115|ref|NC_009085.1| | 2032249 | 2032280 | 32 |
| exprReg_13722 | gi|126640115|ref|NC_009085.1| | 2032571 | 2032587 | 17 |
| exprReg_13723 | gi|126640115|ref|NC_009085.1| | 2032602 | 2032680 | 79 |
| exprReg_13727 | gi|126640115|ref|NC_009085.1| | 2033450 | 2033466 | 17 |
| exprReg_13728 | gi|126640115|ref|NC_009085.1| | 2033511 | 2033598 | 88 |
| exprReg_13729 | gi|126640115|ref|NC_009085.1| | 2033600 | 2033737 | 138 |
| exprReg_13730 | gi|126640115|ref|NC_009085.1| | 2033773 | 2033830 | 58 |
| exprReg_13731 | gi|126640115|ref|NC_009085.1| | 2033841 | 2033858 | 18 |
| exprReg_13732 | gi|126640115|ref|NC_009085.1| | 2033893 | 2033911 | 19 |
| exprReg_13733 | gi|126640115|ref|NC_009085.1| | 2034009 | 2034026 | 18 |
| exprReg_13734 | gi|126640115|ref|NC_009085.1| | 2034038 | 2034085 | 48 |
| exprReg_13735 | gi|126640115|ref|NC_009085.1| | 2034093 | 2034111 | 19 |
| exprReg_13736 | gi|126640115|ref|NC_009085.1| | 2034127 | 2034145 | 19 |
| exprReg_13737 | gi|126640115|ref|NC_009085.1| | 2034214 | 2034285 | 72 |
| exprReg_13750 | gi|126640115|ref|NC_009085.1| | 2036394 | 2036446 | 53 |
| exprReg_13773 | gi|126640115|ref|NC_009085.1| | 2040089 | 2040164 | 76 |
| exprReg_13774 | gi|126640115|ref|NC_009085.1| | 2040198 | 2040242 | 45 |
| exprReg_13778 | gi|126640115|ref|NC_009085.1| | 2040816 | 2040864 | 49 |
| exprReg_13782 | gi|126640115|ref|NC_009085.1| | 2041865 | 2041884 | 20 |
| exprReg_13792 | gi|126640115|ref|NC_009085.1| | 2042755 | 2042822 | 68 |
| exprReg_13793 | gi|126640115|ref|NC_009085.1| | 2042904 | 2043072 | 169 |
| exprReg_13819 | gi|126640115|ref|NC_009085.1| | 2048489 | 2048650 | 162 |
| exprReg_13820 | gi|126640115|ref|NC_009085.1| | 2048686 | 2048755 | 70 |
| exprReg_13821 | gi|126640115|ref|NC_009085.1| | 2048762 | 2048825 | 64 |
| exprReg_13822 | gi|126640115|ref|NC_009085.1| | 2048858 | 2048874 | 17 |
| exprReg_13823 | gi|126640115|ref|NC_009085.1| | 2049134 | 2049311 | 178 |
| exprReg_13824 | gi|126640115|ref|NC_009085.1| | 2049561 | 2049603 | 43 |
| exprReg_13832 | gi|126640115|ref|NC_009085.1| | 2050499 | 2050517 | 19 |
| exprReg_13833 | gi|126640115|ref|NC_009085.1| | 2050562 | 2050592 | 31 |
| exprReg_13834 | gi|126640115|ref|NC_009085.1| | 2050813 | 2050831 | 19 |
| exprReg_13835 | gi|126640115|ref|NC_009085.1| | 2051048 | 2051098 | 51 |
| exprReg_13841 | gi|126640115|ref|NC_009085.1| | 2052131 | 2052190 | 60 |
| exprReg_13849 | gi|126640115|ref|NC_009085.1| | 2053605 | 2053804 | 200 |
| exprReg_13850 | gi|126640115|ref|NC_009085.1| | 2053861 | 2053880 | 20 |
| exprReg_13851 | gi|126640115|ref|NC_009085.1| | 2053893 | 2054012 | 120 |
| exprReg_13852 | gi|126640115|ref|NC_009085.1| | 2054017 | 2054107 | 91 |
| exprReg_13853 | gi|126640115|ref|NC_009085.1| | 2054118 | 2054194 | 77 |
| exprReg_13854 | gi|126640115|ref|NC_009085.1| | 2054197 | 2054285 | 89 |
| exprReg_13857 | gi|126640115|ref|NC_009085.1| | 2054870 | 2054893 | 24 |
| exprReg_13858 | gi|126640115|ref|NC_009085.1| | 2054939 | 2055066 | 128 |
| exprReg_13859 | gi|126640115|ref|NC_009085.1| | 2055146 | 2055452 | 307 |
| exprReg_13867 | gi|126640115|ref|NC_009085.1| | 2057082 | 2057123 | 42 |
| exprReg_13868 | gi|126640115|ref|NC_009085.1| | 2057171 | 2057233 | 63 |
| exprReg_13869 | gi|126640115|ref|NC_009085.1| | 2057489 | 2057576 | 88 |
| exprReg_13870 | gi|126640115|ref|NC_009085.1| | 2057590 | 2057639 | 50 |
| exprReg_13871 | gi|126640115|ref|NC_009085.1| | 2057696 | 2057815 | 120 |
| exprReg_13872 | gi|126640115|ref|NC_009085.1| | 2057846 | 2057867 | 22 |
| exprReg_13873 | gi|126640115|ref|NC_009085.1| | 2058023 | 2058048 | 26 |
| exprReg_13883 | gi|126640115|ref|NC_009085.1| | 2059608 | 2059625 | 18 |
| exprReg_13884 | gi|126640115|ref|NC_009085.1| | 2059656 | 2059700 | 45 |
| exprReg_13916 | gi|126640115|ref|NC_009085.1| | 2064639 | 2064660 | 22 |
| exprReg_13917 | gi|126640115|ref|NC_009085.1| | 2064735 | 2064885 | 151 |
| exprReg_13940 | gi|126640115|ref|NC_009085.1| | 2067454 | 2067528 | 75 |
| exprReg_13941 | gi|126640115|ref|NC_009085.1| | 2068051 | 2068103 | 53 |
| exprReg_13950 | gi|126640115|ref|NC_009085.1| | 2070538 | 2070565 | 28 |
| exprReg_13951 | gi|126640115|ref|NC_009085.1| | 2070697 | 2070714 | 18 |
| exprReg_13952 | gi|126640115|ref|NC_009085.1| | 2070949 | 2070965 | 17 |
| exprReg_13953 | gi|126640115|ref|NC_009085.1| | 2071244 | 2071287 | 44 |
| exprReg_13954 | gi|126640115|ref|NC_009085.1| | 2071515 | 2071531 | 17 |
| exprReg_13955 | gi|126640115|ref|NC_009085.1| | 2071937 | 2071985 | 49 |
| exprReg_13956 | gi|126640115|ref|NC_009085.1| | 2072008 | 2072229 | 222 |
| exprReg_13957 | gi|126640115|ref|NC_009085.1| | 2072241 | 2072257 | 17 |
| exprReg_13958 | gi|126640115|ref|NC_009085.1| | 2072399 | 2072441 | 43 |
| exprReg_13959 | gi|126640115|ref|NC_009085.1| | 2072500 | 2072554 | 55 |
| exprReg_13975 | gi|126640115|ref|NC_009085.1| | 2075105 | 2075122 | 18 |
| exprReg_13976 | gi|126640115|ref|NC_009085.1| | 2075138 | 2075154 | 17 |
| exprReg_13990 | gi|126640115|ref|NC_009085.1| | 2077439 | 2077589 | 151 |
| exprReg_13991 | gi|126640115|ref|NC_009085.1| | 2077663 | 2077690 | 28 |
| exprReg_14008 | gi|126640115|ref|NC_009085.1| | 2080608 | 2080626 | 19 |
| exprReg_14019 | gi|126640115|ref|NC_009085.1| | 2082684 | 2082832 | 149 |
| exprReg_14020 | gi|126640115|ref|NC_009085.1| | 2082927 | 2083072 | 146 |
| exprReg_14026 | gi|126640115|ref|NC_009085.1| | 2084112 | 2084147 | 36 |
| exprReg_14027 | gi|126640115|ref|NC_009085.1| | 2084250 | 2084271 | 22 |
| exprReg_14034 | gi|126640115|ref|NC_009085.1| | 2085427 | 2085445 | 19 |
| exprReg_14049 | gi|126640115|ref|NC_009085.1| | 2087633 | 2087655 | 23 |
| exprReg_14054 | gi|126640115|ref|NC_009085.1| | 2088732 | 2088783 | 52 |
| exprReg_14077 | gi|126640115|ref|NC_009085.1| | 2093707 | 2093743 | 37 |
| exprReg_14083 | gi|126640115|ref|NC_009085.1| | 2094643 | 2094669 | 27 |
| exprReg_14086 | gi|126640115|ref|NC_009085.1| | 2095294 | 2095312 | 19 |
| exprReg_14087 | gi|126640115|ref|NC_009085.1| | 2095329 | 2095345 | 17 |
| exprReg_14088 | gi|126640115|ref|NC_009085.1| | 2095390 | 2095505 | 116 |
| exprReg_14099 | gi|126640115|ref|NC_009085.1| | 2097070 | 2097096 | 27 |
| exprReg_14107 | gi|126640115|ref|NC_009085.1| | 2098150 | 2098239 | 90 |
| exprReg_14113 | gi|126640115|ref|NC_009085.1| | 2099266 | 2099284 | 19 |
| exprReg_14118 | gi|126640115|ref|NC_009085.1| | 2100182 | 2100236 | 55 |
| exprReg_14165 | gi|126640115|ref|NC_009085.1| | 2108024 | 2108064 | 41 |
| exprReg_14166 | gi|126640115|ref|NC_009085.1| | 2108236 | 2108253 | 18 |
| exprReg_14167 | gi|126640115|ref|NC_009085.1| | 2108320 | 2108339 | 20 |
| exprReg_14168 | gi|126640115|ref|NC_009085.1| | 2108386 | 2108402 | 17 |
| exprReg_14169 | gi|126640115|ref|NC_009085.1| | 2108532 | 2108561 | 30 |
| exprReg_14170 | gi|126640115|ref|NC_009085.1| | 2108839 | 2108874 | 36 |
| exprReg_14171 | gi|126640115|ref|NC_009085.1| | 2108963 | 2109006 | 44 |
| exprReg_14178 | gi|126640115|ref|NC_009085.1| | 2109709 | 2109738 | 30 |
| exprReg_14179 | gi|126640115|ref|NC_009085.1| | 2109762 | 2109783 | 22 |
| exprReg_14180 | gi|126640115|ref|NC_009085.1| | 2109804 | 2109919 | 116 |
| exprReg_14181 | gi|126640115|ref|NC_009085.1| | 2109943 | 2109962 | 20 |
| exprReg_14182 | gi|126640115|ref|NC_009085.1| | 2109982 | 2109999 | 18 |
| exprReg_14183 | gi|126640115|ref|NC_009085.1| | 2110091 | 2110111 | 21 |
| exprReg_14184 | gi|126640115|ref|NC_009085.1| | 2110158 | 2110189 | 32 |
| exprReg_14185 | gi|126640115|ref|NC_009085.1| | 2110195 | 2110211 | 17 |
| exprReg_14186 | gi|126640115|ref|NC_009085.1| | 2110282 | 2110332 | 51 |
| exprReg_14187 | gi|126640115|ref|NC_009085.1| | 2110363 | 2110379 | 17 |
| exprReg_14188 | gi|126640115|ref|NC_009085.1| | 2110517 | 2110613 | 97 |
| exprReg_14189 | gi|126640115|ref|NC_009085.1| | 2110616 | 2110638 | 23 |
| exprReg_14190 | gi|126640115|ref|NC_009085.1| | 2110738 | 2110754 | 17 |
| exprReg_14191 | gi|126640115|ref|NC_009085.1| | 2110765 | 2110786 | 22 |
| exprReg_14192 | gi|126640115|ref|NC_009085.1| | 2110900 | 2110916 | 17 |
| exprReg_14193 | gi|126640115|ref|NC_009085.1| | 2110959 | 2110985 | 27 |
| exprReg_14194 | gi|126640115|ref|NC_009085.1| | 2111105 | 2111324 | 220 |
| exprReg_14195 | gi|126640115|ref|NC_009085.1| | 2111448 | 2111485 | 38 |
| exprReg_14196 | gi|126640115|ref|NC_009085.1| | 2111524 | 2111542 | 19 |
| exprReg_14197 | gi|126640115|ref|NC_009085.1| | 2111550 | 2111658 | 109 |
| exprReg_14198 | gi|126640115|ref|NC_009085.1| | 2111733 | 2111777 | 45 |
| exprReg_14199 | gi|126640115|ref|NC_009085.1| | 2111890 | 2111952 | 63 |
| exprReg_14200 | gi|126640115|ref|NC_009085.1| | 2111996 | 2112143 | 148 |
| exprReg_14201 | gi|126640115|ref|NC_009085.1| | 2112147 | 2112202 | 56 |
| exprReg_14202 | gi|126640115|ref|NC_009085.1| | 2112239 | 2112284 | 46 |
| exprReg_14203 | gi|126640115|ref|NC_009085.1| | 2112547 | 2112599 | 53 |
| exprReg_14204 | gi|126640115|ref|NC_009085.1| | 2112702 | 2112733 | 32 |
| exprReg_14205 | gi|126640115|ref|NC_009085.1| | 2112806 | 2112823 | 18 |
| exprReg_14206 | gi|126640115|ref|NC_009085.1| | 2112838 | 2112876 | 39 |
| exprReg_14207 | gi|126640115|ref|NC_009085.1| | 2112991 | 2113022 | 32 |
| exprReg_14208 | gi|126640115|ref|NC_009085.1| | 2113331 | 2113348 | 18 |
| exprReg_14209 | gi|126640115|ref|NC_009085.1| | 2113451 | 2113485 | 35 |
| exprReg_14210 | gi|126640115|ref|NC_009085.1| | 2113507 | 2113525 | 19 |
| exprReg_14214 | gi|126640115|ref|NC_009085.1| | 2114111 | 2114127 | 17 |
| exprReg_14215 | gi|126640115|ref|NC_009085.1| | 2114206 | 2114254 | 49 |
| exprReg_14216 | gi|126640115|ref|NC_009085.1| | 2114454 | 2114647 | 194 |
| exprReg_14230 | gi|126640115|ref|NC_009085.1| | 2117481 | 2117508 | 28 |
| exprReg_14231 | gi|126640115|ref|NC_009085.1| | 2117512 | 2117633 | 122 |
| exprReg_14240 | gi|126640115|ref|NC_009085.1| | 2118822 | 2118869 | 48 |
| exprReg_14241 | gi|126640115|ref|NC_009085.1| | 2118875 | 2118953 | 79 |
| exprReg_14242 | gi|126640115|ref|NC_009085.1| | 2119088 | 2119122 | 35 |
| exprReg_14243 | gi|126640115|ref|NC_009085.1| | 2119164 | 2119186 | 23 |
| exprReg_14254 | gi|126640115|ref|NC_009085.1| | 2121012 | 2121038 | 27 |
| exprReg_14258 | gi|126640115|ref|NC_009085.1| | 2121459 | 2121496 | 38 |
| exprReg_14263 | gi|126640115|ref|NC_009085.1| | 2122339 | 2122370 | 32 |
| exprReg_14264 | gi|126640115|ref|NC_009085.1| | 2122429 | 2122448 | 20 |
| exprReg_14276 | gi|126640115|ref|NC_009085.1| | 2124034 | 2124050 | 17 |
| exprReg_14277 | gi|126640115|ref|NC_009085.1| | 2124260 | 2124282 | 23 |
| exprReg_14278 | gi|126640115|ref|NC_009085.1| | 2124364 | 2124401 | 38 |
| exprReg_14286 | gi|126640115|ref|NC_009085.1| | 2125128 | 2125153 | 26 |
| exprReg_14291 | gi|126640115|ref|NC_009085.1| | 2125783 | 2125808 | 26 |
| exprReg_14297 | gi|126640115|ref|NC_009085.1| | 2127030 | 2127055 | 26 |
| exprReg_14302 | gi|126640115|ref|NC_009085.1| | 2127721 | 2127774 | 54 |
| exprReg_14303 | gi|126640115|ref|NC_009085.1| | 2127880 | 2127915 | 36 |
| exprReg_14314 | gi|126640115|ref|NC_009085.1| | 2129809 | 2129867 | 59 |
| exprReg_14315 | gi|126640115|ref|NC_009085.1| | 2129898 | 2130074 | 177 |
| exprReg_14322 | gi|126640115|ref|NC_009085.1| | 2131291 | 2131332 | 42 |
| exprReg_14326 | gi|126640115|ref|NC_009085.1| | 2131870 | 2131888 | 19 |
| exprReg_14327 | gi|126640115|ref|NC_009085.1| | 2131895 | 2131916 | 22 |
| exprReg_14332 | gi|126640115|ref|NC_009085.1| | 2133030 | 2133115 | 86 |
| exprReg_14333 | gi|126640115|ref|NC_009085.1| | 2133118 | 2133175 | 58 |
| exprReg_14344 | gi|126640115|ref|NC_009085.1| | 2134435 | 2134477 | 43 |
| exprReg_14349 | gi|126640115|ref|NC_009085.1| | 2135387 | 2135482 | 96 |
| exprReg_14350 | gi|126640115|ref|NC_009085.1| | 2135503 | 2135561 | 59 |
| exprReg_14351 | gi|126640115|ref|NC_009085.1| | 2135670 | 2135699 | 30 |
| exprReg_14373 | gi|126640115|ref|NC_009085.1| | 2140151 | 2140240 | 90 |
| exprReg_14376 | gi|126640115|ref|NC_009085.1| | 2140529 | 2140627 | 99 |
| exprReg_14386 | gi|126640115|ref|NC_009085.1| | 2142638 | 2142676 | 39 |
| exprReg_14396 | gi|126640115|ref|NC_009085.1| | 2144136 | 2144154 | 19 |
| exprReg_14397 | gi|126640115|ref|NC_009085.1| | 2144168 | 2144200 | 33 |
| exprReg_14402 | gi|126640115|ref|NC_009085.1| | 2145005 | 2145036 | 32 |
| exprReg_14403 | gi|126640115|ref|NC_009085.1| | 2145052 | 2145227 | 176 |
| exprReg_14414 | gi|126640115|ref|NC_009085.1| | 2146771 | 2146841 | 71 |
| exprReg_14415 | gi|126640115|ref|NC_009085.1| | 2146845 | 2146875 | 31 |
| exprReg_14421 | gi|126640115|ref|NC_009085.1| | 2147882 | 2147949 | 68 |
| exprReg_14422 | gi|126640115|ref|NC_009085.1| | 2147952 | 2147985 | 34 |
| exprReg_14438 | gi|126640115|ref|NC_009085.1| | 2151498 | 2151600 | 103 |
| exprReg_14439 | gi|126640115|ref|NC_009085.1| | 2151636 | 2151761 | 126 |
| exprReg_14448 | gi|126640115|ref|NC_009085.1| | 2154046 | 2154065 | 20 |
| exprReg_14460 | gi|126640115|ref|NC_009085.1| | 2155484 | 2155548 | 65 |
| exprReg_14466 | gi|126640115|ref|NC_009085.1| | 2156891 | 2156963 | 73 |
| exprReg_14477 | gi|126640115|ref|NC_009085.1| | 2159184 | 2159200 | 17 |
| exprReg_14478 | gi|126640115|ref|NC_009085.1| | 2160317 | 2160336 | 20 |
| exprReg_14486 | gi|126640115|ref|NC_009085.1| | 2161508 | 2161562 | 55 |
| exprReg_14495 | gi|126640115|ref|NC_009085.1| | 2163279 | 2163296 | 18 |
| exprReg_14496 | gi|126640115|ref|NC_009085.1| | 2163373 | 2163458 | 86 |
| exprReg_14497 | gi|126640115|ref|NC_009085.1| | 2163515 | 2163531 | 17 |
| exprReg_14517 | gi|126640115|ref|NC_009085.1| | 2167059 | 2167121 | 63 |
| exprReg_14518 | gi|126640115|ref|NC_009085.1| | 2167139 | 2167164 | 26 |
| exprReg_14519 | gi|126640115|ref|NC_009085.1| | 2167181 | 2167247 | 67 |
| exprReg_14528 | gi|126640115|ref|NC_009085.1| | 2168258 | 2168312 | 55 |
| exprReg_14529 | gi|126640115|ref|NC_009085.1| | 2168319 | 2168354 | 36 |
| exprReg_14530 | gi|126640115|ref|NC_009085.1| | 2168359 | 2168378 | 20 |
| exprReg_14531 | gi|126640115|ref|NC_009085.1| | 2168407 | 2168447 | 41 |
| exprReg_14537 | gi|126640115|ref|NC_009085.1| | 2168988 | 2169007 | 20 |
| exprReg_14538 | gi|126640115|ref|NC_009085.1| | 2169012 | 2169046 | 35 |
| exprReg_14539 | gi|126640115|ref|NC_009085.1| | 2169192 | 2169257 | 66 |
| exprReg_14549 | gi|126640115|ref|NC_009085.1| | 2170744 | 2170790 | 47 |
| exprReg_14581 | gi|126640115|ref|NC_009085.1| | 2176174 | 2176212 | 39 |
| exprReg_14586 | gi|126640115|ref|NC_009085.1| | 2177025 | 2177090 | 66 |
| exprReg_14599 | gi|126640115|ref|NC_009085.1| | 2178658 | 2178691 | 34 |
| exprReg_14600 | gi|126640115|ref|NC_009085.1| | 2178730 | 2178800 | 71 |
| exprReg_14604 | gi|126640115|ref|NC_009085.1| | 2179543 | 2179572 | 30 |
| exprReg_14605 | gi|126640115|ref|NC_009085.1| | 2179588 | 2179672 | 85 |
| exprReg_14612 | gi|126640115|ref|NC_009085.1| | 2180375 | 2180471 | 97 |
| exprReg_14626 | gi|126640115|ref|NC_009085.1| | 2183217 | 2183322 | 106 |
| exprReg_14635 | gi|126640115|ref|NC_009085.1| | 2184587 | 2184676 | 90 |
| exprReg_14646 | gi|126640115|ref|NC_009085.1| | 2186098 | 2186168 | 71 |
| exprReg_14652 | gi|126640115|ref|NC_009085.1| | 2186963 | 2187028 | 66 |
| exprReg_14653 | gi|126640115|ref|NC_009085.1| | 2187055 | 2187105 | 51 |
| exprReg_14658 | gi|126640115|ref|NC_009085.1| | 2187686 | 2187702 | 17 |
| exprReg_14664 | gi|126640115|ref|NC_009085.1| | 2188401 | 2188417 | 17 |
| exprReg_14673 | gi|126640115|ref|NC_009085.1| | 2190220 | 2190295 | 76 |
| exprReg_14696 | gi|126640115|ref|NC_009085.1| | 2194940 | 2194975 | 36 |
| exprReg_14697 | gi|126640115|ref|NC_009085.1| | 2195100 | 2195129 | 30 |
| exprReg_14703 | gi|126640115|ref|NC_009085.1| | 2195931 | 2196043 | 113 |
| exprReg_14706 | gi|126640115|ref|NC_009085.1| | 2196472 | 2196597 | 126 |
| exprReg_14707 | gi|126640115|ref|NC_009085.1| | 2196874 | 2196932 | 59 |
| exprReg_14708 | gi|126640115|ref|NC_009085.1| | 2197110 | 2197131 | 22 |
| exprReg_14742 | gi|126640115|ref|NC_009085.1| | 2201919 | 2202138 | 220 |
| exprReg_14743 | gi|126640115|ref|NC_009085.1| | 2202163 | 2202181 | 19 |
| exprReg_14744 | gi|126640115|ref|NC_009085.1| | 2202266 | 2202288 | 23 |
| exprReg_14745 | gi|126640115|ref|NC_009085.1| | 2202296 | 2202319 | 24 |
| exprReg_14746 | gi|126640115|ref|NC_009085.1| | 2202354 | 2202418 | 65 |
| exprReg_14747 | gi|126640115|ref|NC_009085.1| | 2202434 | 2202451 | 18 |
| exprReg_14748 | gi|126640115|ref|NC_009085.1| | 2202613 | 2202630 | 18 |
| exprReg_14757 | gi|126640115|ref|NC_009085.1| | 2204411 | 2204427 | 17 |
| exprReg_14776 | gi|126640115|ref|NC_009085.1| | 2208038 | 2208085 | 48 |
| exprReg_14777 | gi|126640115|ref|NC_009085.1| | 2208106 | 2208140 | 35 |
| exprReg_14800 | gi|126640115|ref|NC_009085.1| | 2210555 | 2210641 | 87 |
| exprReg_14801 | gi|126640115|ref|NC_009085.1| | 2210661 | 2210789 | 129 |
| exprReg_14802 | gi|126640115|ref|NC_009085.1| | 2210834 | 2210986 | 153 |
| exprReg_14845 | gi|126640115|ref|NC_009085.1| | 2216074 | 2216095 | 22 |
| exprReg_14846 | gi|126640115|ref|NC_009085.1| | 2216097 | 2216116 | 20 |
| exprReg_14847 | gi|126640115|ref|NC_009085.1| | 2216142 | 2216173 | 32 |
| exprReg_14848 | gi|126640115|ref|NC_009085.1| | 2216223 | 2216269 | 47 |
| exprReg_14849 | gi|126640115|ref|NC_009085.1| | 2216290 | 2216366 | 77 |
| exprReg_14850 | gi|126640115|ref|NC_009085.1| | 2216371 | 2216397 | 27 |
| exprReg_14851 | gi|126640115|ref|NC_009085.1| | 2216409 | 2216427 | 19 |
| exprReg_14852 | gi|126640115|ref|NC_009085.1| | 2216443 | 2216497 | 55 |
| exprReg_14853 | gi|126640115|ref|NC_009085.1| | 2216510 | 2216530 | 21 |
| exprReg_14854 | gi|126640115|ref|NC_009085.1| | 2216535 | 2216551 | 17 |
| exprReg_14855 | gi|126640115|ref|NC_009085.1| | 2216634 | 2216707 | 74 |
| exprReg_14856 | gi|126640115|ref|NC_009085.1| | 2216724 | 2216741 | 18 |
| exprReg_14857 | gi|126640115|ref|NC_009085.1| | 2216766 | 2216876 | 111 |
| exprReg_14858 | gi|126640115|ref|NC_009085.1| | 2216896 | 2216923 | 28 |
| exprReg_14859 | gi|126640115|ref|NC_009085.1| | 2216942 | 2217071 | 130 |
| exprReg_14860 | gi|126640115|ref|NC_009085.1| | 2217088 | 2217130 | 43 |
| exprReg_14861 | gi|126640115|ref|NC_009085.1| | 2217202 | 2217223 | 22 |
| exprReg_14862 | gi|126640115|ref|NC_009085.1| | 2217307 | 2217338 | 32 |
| exprReg_14863 | gi|126640115|ref|NC_009085.1| | 2217341 | 2217363 | 23 |
| exprReg_14864 | gi|126640115|ref|NC_009085.1| | 2217385 | 2217401 | 17 |
| exprReg_14865 | gi|126640115|ref|NC_009085.1| | 2217456 | 2217473 | 18 |
| exprReg_14866 | gi|126640115|ref|NC_009085.1| | 2217490 | 2217506 | 17 |
| exprReg_14867 | gi|126640115|ref|NC_009085.1| | 2217514 | 2217544 | 31 |
| exprReg_14868 | gi|126640115|ref|NC_009085.1| | 2217560 | 2217713 | 154 |
| exprReg_14883 | gi|126640115|ref|NC_009085.1| | 2218985 | 2219049 | 65 |
| exprReg_14884 | gi|126640115|ref|NC_009085.1| | 2219122 | 2219159 | 38 |
| exprReg_14885 | gi|126640115|ref|NC_009085.1| | 2219188 | 2219227 | 40 |
| exprReg_14886 | gi|126640115|ref|NC_009085.1| | 2219233 | 2219269 | 37 |
| exprReg_14887 | gi|126640115|ref|NC_009085.1| | 2219309 | 2219333 | 25 |
| exprReg_14888 | gi|126640115|ref|NC_009085.1| | 2219336 | 2219467 | 132 |
| exprReg_14912 | gi|126640115|ref|NC_009085.1| | 2221586 | 2221602 | 17 |
| exprReg_14913 | gi|126640115|ref|NC_009085.1| | 2221615 | 2221631 | 17 |
| exprReg_14949 | gi|126640115|ref|NC_009085.1| | 2226908 | 2227099 | 192 |
| exprReg_14950 | gi|126640115|ref|NC_009085.1| | 2227185 | 2227235 | 51 |
| exprReg_14967 | gi|126640115|ref|NC_009085.1| | 2229308 | 2229326 | 19 |
| exprReg_14968 | gi|126640115|ref|NC_009085.1| | 2229386 | 2229406 | 21 |
| exprReg_14969 | gi|126640115|ref|NC_009085.1| | 2229535 | 2229556 | 22 |
| exprReg_14970 | gi|126640115|ref|NC_009085.1| | 2229578 | 2229598 | 21 |
| exprReg_14980 | gi|126640115|ref|NC_009085.1| | 2230644 | 2230774 | 131 |
| exprReg_14981 | gi|126640115|ref|NC_009085.1| | 2230802 | 2230836 | 35 |
| exprReg_14984 | gi|126640115|ref|NC_009085.1| | 2231962 | 2232044 | 83 |
| exprReg_15004 | gi|126640115|ref|NC_009085.1| | 2234966 | 2235016 | 51 |
| exprReg_15011 | gi|126640115|ref|NC_009085.1| | 2236555 | 2236616 | 62 |
| exprReg_15012 | gi|126640115|ref|NC_009085.1| | 2236666 | 2236698 | 33 |
| exprReg_15013 | gi|126640115|ref|NC_009085.1| | 2236786 | 2236824 | 39 |
| exprReg_15016 | gi|126640115|ref|NC_009085.1| | 2237446 | 2237472 | 27 |
| exprReg_15028 | gi|126640115|ref|NC_009085.1| | 2239141 | 2239165 | 25 |
| exprReg_15031 | gi|126640115|ref|NC_009085.1| | 2240108 | 2240147 | 40 |
| exprReg_15032 | gi|126640115|ref|NC_009085.1| | 2240177 | 2240233 | 57 |
| exprReg_15033 | gi|126640115|ref|NC_009085.1| | 2240267 | 2240290 | 24 |
| exprReg_15047 | gi|126640115|ref|NC_009085.1| | 2242450 | 2242506 | 57 |
| exprReg_15048 | gi|126640115|ref|NC_009085.1| | 2242511 | 2242590 | 80 |
| exprReg_15054 | gi|126640115|ref|NC_009085.1| | 2243829 | 2243848 | 20 |
| exprReg_15068 | gi|126640115|ref|NC_009085.1| | 2245612 | 2245666 | 55 |
| exprReg_15069 | gi|126640115|ref|NC_009085.1| | 2245883 | 2246008 | 126 |
| exprReg_15083 | gi|126640115|ref|NC_009085.1| | 2247210 | 2247231 | 22 |
| exprReg_15084 | gi|126640115|ref|NC_009085.1| | 2247248 | 2247276 | 29 |
| exprReg_15085 | gi|126640115|ref|NC_009085.1| | 2247281 | 2247301 | 21 |
| exprReg_15086 | gi|126640115|ref|NC_009085.1| | 2247357 | 2247394 | 38 |
| exprReg_15089 | gi|126640115|ref|NC_009085.1| | 2247993 | 2248051 | 59 |
| exprReg_15090 | gi|126640115|ref|NC_009085.1| | 2248073 | 2248205 | 133 |
| exprReg_15091 | gi|126640115|ref|NC_009085.1| | 2248219 | 2248295 | 77 |
| exprReg_15098 | gi|126640115|ref|NC_009085.1| | 2249877 | 2249897 | 21 |
| exprReg_15099 | gi|126640115|ref|NC_009085.1| | 2249911 | 2249968 | 58 |
| exprReg_15100 | gi|126640115|ref|NC_009085.1| | 2249989 | 2250006 | 18 |
| exprReg_15101 | gi|126640115|ref|NC_009085.1| | 2250070 | 2250113 | 44 |
| exprReg_15102 | gi|126640115|ref|NC_009085.1| | 2250168 | 2250209 | 42 |
| exprReg_15123 | gi|126640115|ref|NC_009085.1| | 2252874 | 2252918 | 45 |
| exprReg_15140 | gi|126640115|ref|NC_009085.1| | 2256010 | 2256156 | 147 |
| exprReg_15143 | gi|126640115|ref|NC_009085.1| | 2256882 | 2256900 | 19 |
| exprReg_15144 | gi|126640115|ref|NC_009085.1| | 2256922 | 2257005 | 84 |
| exprReg_15145 | gi|126640115|ref|NC_009085.1| | 2257020 | 2257037 | 18 |
| exprReg_15146 | gi|126640115|ref|NC_009085.1| | 2257149 | 2257219 | 71 |
| exprReg_15147 | gi|126640115|ref|NC_009085.1| | 2257604 | 2257740 | 137 |
| exprReg_15148 | gi|126640115|ref|NC_009085.1| | 2257776 | 2257805 | 30 |
| exprReg_15162 | gi|126640115|ref|NC_009085.1| | 2259788 | 2259965 | 178 |
| exprReg_15182 | gi|126640115|ref|NC_009085.1| | 2261751 | 2261783 | 33 |
| exprReg_15183 | gi|126640115|ref|NC_009085.1| | 2261792 | 2261845 | 54 |
| exprReg_15184 | gi|126640115|ref|NC_009085.1| | 2261863 | 2261938 | 76 |
| exprReg_15185 | gi|126640115|ref|NC_009085.1| | 2262017 | 2262115 | 99 |
| exprReg_15186 | gi|126640115|ref|NC_009085.1| | 2262200 | 2262219 | 20 |
| exprReg_15187 | gi|126640115|ref|NC_009085.1| | 2262240 | 2262267 | 28 |
| exprReg_15190 | gi|126640115|ref|NC_009085.1| | 2262858 | 2262877 | 20 |
| exprReg_15191 | gi|126640115|ref|NC_009085.1| | 2262895 | 2262948 | 54 |
| exprReg_15192 | gi|126640115|ref|NC_009085.1| | 2262967 | 2263066 | 100 |
| exprReg_15209 | gi|126640115|ref|NC_009085.1| | 2265553 | 2265572 | 20 |
| exprReg_15210 | gi|126640115|ref|NC_009085.1| | 2265649 | 2265669 | 21 |
| exprReg_15211 | gi|126640115|ref|NC_009085.1| | 2265835 | 2265871 | 37 |
| exprReg_15212 | gi|126640115|ref|NC_009085.1| | 2266149 | 2266174 | 26 |
| exprReg_15213 | gi|126640115|ref|NC_009085.1| | 2266219 | 2266266 | 48 |
| exprReg_15214 | gi|126640115|ref|NC_009085.1| | 2266406 | 2266433 | 28 |
| exprReg_15215 | gi|126640115|ref|NC_009085.1| | 2266529 | 2266651 | 123 |
| exprReg_15216 | gi|126640115|ref|NC_009085.1| | 2266683 | 2266701 | 19 |
| exprReg_15217 | gi|126640115|ref|NC_009085.1| | 2266828 | 2266847 | 20 |
| exprReg_15218 | gi|126640115|ref|NC_009085.1| | 2266891 | 2266918 | 28 |
| exprReg_15219 | gi|126640115|ref|NC_009085.1| | 2266951 | 2266968 | 18 |
| exprReg_15238 | gi|126640115|ref|NC_009085.1| | 2270441 | 2270467 | 27 |
| exprReg_15239 | gi|126640115|ref|NC_009085.1| | 2270560 | 2270576 | 17 |
| exprReg_15245 | gi|126640115|ref|NC_009085.1| | 2271660 | 2271678 | 19 |
| exprReg_15246 | gi|126640115|ref|NC_009085.1| | 2271691 | 2271724 | 34 |
| exprReg_15247 | gi|126640115|ref|NC_009085.1| | 2271766 | 2271813 | 48 |
| exprReg_15248 | gi|126640115|ref|NC_009085.1| | 2271833 | 2271938 | 106 |
| exprReg_15249 | gi|126640115|ref|NC_009085.1| | 2272206 | 2272222 | 17 |
| exprReg_15250 | gi|126640115|ref|NC_009085.1| | 2272242 | 2272259 | 18 |
| exprReg_15251 | gi|126640115|ref|NC_009085.1| | 2272484 | 2272667 | 184 |
| exprReg_15252 | gi|126640115|ref|NC_009085.1| | 2272725 | 2272747 | 23 |
| exprReg_15253 | gi|126640115|ref|NC_009085.1| | 2272932 | 2273141 | 210 |
| exprReg_15263 | gi|126640115|ref|NC_009085.1| | 2274060 | 2274076 | 17 |
| exprReg_15282 | gi|126640115|ref|NC_009085.1| | 2276025 | 2276042 | 18 |
| exprReg_15283 | gi|126640115|ref|NC_009085.1| | 2276069 | 2276089 | 21 |
| exprReg_15284 | gi|126640115|ref|NC_009085.1| | 2276165 | 2276183 | 19 |
| exprReg_15285 | gi|126640115|ref|NC_009085.1| | 2276239 | 2276270 | 32 |
| exprReg_15286 | gi|126640115|ref|NC_009085.1| | 2276285 | 2276355 | 71 |
| exprReg_15297 | gi|126640115|ref|NC_009085.1| | 2277864 | 2277903 | 40 |
| exprReg_15369 | gi|126640115|ref|NC_009085.1| | 2287255 | 2287301 | 47 |
| exprReg_15386 | gi|126640115|ref|NC_009085.1| | 2289642 | 2289679 | 38 |
| exprReg_15387 | gi|126640115|ref|NC_009085.1| | 2289708 | 2289729 | 22 |
| exprReg_15388 | gi|126640115|ref|NC_009085.1| | 2289737 | 2289852 | 116 |
| exprReg_15389 | gi|126640115|ref|NC_009085.1| | 2289933 | 2289972 | 40 |
| exprReg_15413 | gi|126640115|ref|NC_009085.1| | 2292513 | 2292554 | 42 |
| exprReg_15419 | gi|126640115|ref|NC_009085.1| | 2293412 | 2293459 | 48 |
| exprReg_15450 | gi|126640115|ref|NC_009085.1| | 2297598 | 2297765 | 168 |
| exprReg_15451 | gi|126640115|ref|NC_009085.1| | 2297778 | 2297873 | 96 |
| exprReg_15456 | gi|126640115|ref|NC_009085.1| | 2298538 | 2298554 | 17 |
| exprReg_15457 | gi|126640115|ref|NC_009085.1| | 2298608 | 2298627 | 20 |
| exprReg_15458 | gi|126640115|ref|NC_009085.1| | 2298639 | 2298655 | 17 |
| exprReg_15459 | gi|126640115|ref|NC_009085.1| | 2298697 | 2298714 | 18 |
| exprReg_15460 | gi|126640115|ref|NC_009085.1| | 2298872 | 2299043 | 172 |
| exprReg_15461 | gi|126640115|ref|NC_009085.1| | 2299081 | 2299114 | 34 |
| exprReg_15462 | gi|126640115|ref|NC_009085.1| | 2299167 | 2299187 | 21 |
| exprReg_15463 | gi|126640115|ref|NC_009085.1| | 2299300 | 2299341 | 42 |
| exprReg_15464 | gi|126640115|ref|NC_009085.1| | 2299451 | 2299468 | 18 |
| exprReg_15465 | gi|126640115|ref|NC_009085.1| | 2299470 | 2299500 | 31 |
| exprReg_15466 | gi|126640115|ref|NC_009085.1| | 2299809 | 2299826 | 18 |
| exprReg_15479 | gi|126640115|ref|NC_009085.1| | 2301298 | 2301318 | 21 |
| exprReg_15499 | gi|126640115|ref|NC_009085.1| | 2304674 | 2304711 | 38 |
| exprReg_15500 | gi|126640115|ref|NC_009085.1| | 2304721 | 2304762 | 42 |
| exprReg_15501 | gi|126640115|ref|NC_009085.1| | 2304850 | 2304866 | 17 |
| exprReg_15502 | gi|126640115|ref|NC_009085.1| | 2304950 | 2304972 | 23 |
| exprReg_15503 | gi|126640115|ref|NC_009085.1| | 2305021 | 2305044 | 24 |
| exprReg_15530 | gi|126640115|ref|NC_009085.1| | 2308009 | 2308028 | 20 |
| exprReg_15531 | gi|126640115|ref|NC_009085.1| | 2308078 | 2308096 | 19 |
| exprReg_15532 | gi|126640115|ref|NC_009085.1| | 2308162 | 2308191 | 30 |
| exprReg_15542 | gi|126640115|ref|NC_009085.1| | 2308936 | 2308967 | 32 |
| exprReg_15543 | gi|126640115|ref|NC_009085.1| | 2309056 | 2309076 | 21 |
| exprReg_15544 | gi|126640115|ref|NC_009085.1| | 2309096 | 2309130 | 35 |
| exprReg_15556 | gi|126640115|ref|NC_009085.1| | 2310772 | 2310789 | 18 |
| exprReg_15557 | gi|126640115|ref|NC_009085.1| | 2310806 | 2310886 | 81 |
| exprReg_15558 | gi|126640115|ref|NC_009085.1| | 2310919 | 2310936 | 18 |
| exprReg_15559 | gi|126640115|ref|NC_009085.1| | 2310957 | 2310993 | 37 |
| exprReg_15600 | gi|126640115|ref|NC_009085.1| | 2316797 | 2316910 | 114 |
| exprReg_15620 | gi|126640115|ref|NC_009085.1| | 2321070 | 2321124 | 55 |
| exprReg_15621 | gi|126640115|ref|NC_009085.1| | 2321242 | 2321304 | 63 |
| exprReg_15622 | gi|126640115|ref|NC_009085.1| | 2321316 | 2321336 | 21 |
| exprReg_15623 | gi|126640115|ref|NC_009085.1| | 2321370 | 2321494 | 125 |
| exprReg_15624 | gi|126640115|ref|NC_009085.1| | 2321862 | 2321980 | 119 |
| exprReg_15629 | gi|126640115|ref|NC_009085.1| | 2322604 | 2322626 | 23 |
| exprReg_15630 | gi|126640115|ref|NC_009085.1| | 2322650 | 2322678 | 29 |
| exprReg_15634 | gi|126640115|ref|NC_009085.1| | 2323766 | 2323787 | 22 |
| exprReg_15635 | gi|126640115|ref|NC_009085.1| | 2323934 | 2323951 | 18 |
| exprReg_15636 | gi|126640115|ref|NC_009085.1| | 2323991 | 2324011 | 21 |
| exprReg_15645 | gi|126640115|ref|NC_009085.1| | 2325262 | 2325278 | 17 |
| exprReg_15660 | gi|126640115|ref|NC_009085.1| | 2327726 | 2327742 | 17 |
| exprReg_15673 | gi|126640115|ref|NC_009085.1| | 2329538 | 2329579 | 42 |
| exprReg_15674 | gi|126640115|ref|NC_009085.1| | 2329671 | 2329796 | 126 |
| exprReg_15675 | gi|126640115|ref|NC_009085.1| | 2329854 | 2329877 | 24 |
| exprReg_15676 | gi|126640115|ref|NC_009085.1| | 2329883 | 2329905 | 23 |
| exprReg_15677 | gi|126640115|ref|NC_009085.1| | 2330052 | 2330070 | 19 |
| exprReg_15678 | gi|126640115|ref|NC_009085.1| | 2330193 | 2330234 | 42 |
| exprReg_15679 | gi|126640115|ref|NC_009085.1| | 2330251 | 2330300 | 50 |
| exprReg_15680 | gi|126640115|ref|NC_009085.1| | 2330303 | 2330397 | 95 |
| exprReg_15681 | gi|126640115|ref|NC_009085.1| | 2330423 | 2330447 | 25 |
| exprReg_15682 | gi|126640115|ref|NC_009085.1| | 2330494 | 2330510 | 17 |
| exprReg_15683 | gi|126640115|ref|NC_009085.1| | 2330513 | 2330534 | 22 |
| exprReg_15684 | gi|126640115|ref|NC_009085.1| | 2330578 | 2330594 | 17 |
| exprReg_15685 | gi|126640115|ref|NC_009085.1| | 2330696 | 2330746 | 51 |
| exprReg_15686 | gi|126640115|ref|NC_009085.1| | 2330854 | 2330885 | 32 |
| exprReg_15687 | gi|126640115|ref|NC_009085.1| | 2330895 | 2330966 | 72 |
| exprReg_15692 | gi|126640115|ref|NC_009085.1| | 2331725 | 2331783 | 59 |
| exprReg_15699 | gi|126640115|ref|NC_009085.1| | 2333009 | 2333025 | 17 |
| exprReg_15700 | gi|126640115|ref|NC_009085.1| | 2333053 | 2333070 | 18 |
| exprReg_15701 | gi|126640115|ref|NC_009085.1| | 2333488 | 2333648 | 161 |
| exprReg_15705 | gi|126640115|ref|NC_009085.1| | 2334524 | 2334624 | 101 |
| exprReg_15725 | gi|126640115|ref|NC_009085.1| | 2338000 | 2338030 | 31 |
| exprReg_15726 | gi|126640115|ref|NC_009085.1| | 2338036 | 2338220 | 185 |
| exprReg_15727 | gi|126640115|ref|NC_009085.1| | 2338342 | 2338524 | 183 |
| exprReg_15728 | gi|126640115|ref|NC_009085.1| | 2338603 | 2338640 | 38 |
| exprReg_15729 | gi|126640115|ref|NC_009085.1| | 2338848 | 2338949 | 102 |
| exprReg_15730 | gi|126640115|ref|NC_009085.1| | 2339137 | 2339243 | 107 |
| exprReg_15731 | gi|126640115|ref|NC_009085.1| | 2339269 | 2339285 | 17 |
| exprReg_15732 | gi|126640115|ref|NC_009085.1| | 2339359 | 2339431 | 73 |
| exprReg_15733 | gi|126640115|ref|NC_009085.1| | 2339434 | 2339453 | 20 |
| exprReg_15763 | gi|126640115|ref|NC_009085.1| | 2344557 | 2344600 | 44 |
| exprReg_15764 | gi|126640115|ref|NC_009085.1| | 2344730 | 2344798 | 69 |
| exprReg_15765 | gi|126640115|ref|NC_009085.1| | 2345084 | 2345100 | 17 |
| exprReg_15766 | gi|126640115|ref|NC_009085.1| | 2345109 | 2345164 | 56 |
| exprReg_15767 | gi|126640115|ref|NC_009085.1| | 2345167 | 2345206 | 40 |
| exprReg_15768 | gi|126640115|ref|NC_009085.1| | 2345246 | 2345264 | 19 |
| exprReg_15769 | gi|126640115|ref|NC_009085.1| | 2345427 | 2345448 | 22 |
| exprReg_15774 | gi|126640115|ref|NC_009085.1| | 2346203 | 2346221 | 19 |
| exprReg_15775 | gi|126640115|ref|NC_009085.1| | 2346294 | 2346332 | 39 |
| exprReg_15776 | gi|126640115|ref|NC_009085.1| | 2346490 | 2346513 | 24 |
| exprReg_15777 | gi|126640115|ref|NC_009085.1| | 2346524 | 2346705 | 182 |
| exprReg_15778 | gi|126640115|ref|NC_009085.1| | 2346750 | 2346797 | 48 |
| exprReg_15779 | gi|126640115|ref|NC_009085.1| | 2346901 | 2346934 | 34 |
| exprReg_15791 | gi|126640115|ref|NC_009085.1| | 2349312 | 2349365 | 54 |
| exprReg_15792 | gi|126640115|ref|NC_009085.1| | 2349569 | 2349789 | 221 |
| exprReg_15793 | gi|126640115|ref|NC_009085.1| | 2349892 | 2350101 | 210 |
| exprReg_15794 | gi|126640115|ref|NC_009085.1| | 2350151 | 2350222 | 72 |
| exprReg_15795 | gi|126640115|ref|NC_009085.1| | 2350385 | 2350410 | 26 |
| exprReg_15796 | gi|126640115|ref|NC_009085.1| | 2350436 | 2350480 | 45 |
| exprReg_15797 | gi|126640115|ref|NC_009085.1| | 2350526 | 2350550 | 25 |
| exprReg_15801 | gi|126640115|ref|NC_009085.1| | 2351018 | 2351056 | 39 |
| exprReg_15802 | gi|126640115|ref|NC_009085.1| | 2351222 | 2351255 | 34 |
| exprReg_15803 | gi|126640115|ref|NC_009085.1| | 2351298 | 2351352 | 55 |
| exprReg_15804 | gi|126640115|ref|NC_009085.1| | 2351607 | 2351648 | 42 |
| exprReg_15805 | gi|126640115|ref|NC_009085.1| | 2351660 | 2351679 | 20 |
| exprReg_15806 | gi|126640115|ref|NC_009085.1| | 2351683 | 2351711 | 29 |
| exprReg_15808 | gi|126640115|ref|NC_009085.1| | 2352824 | 2353014 | 191 |
| exprReg_15812 | gi|126640115|ref|NC_009085.1| | 2353799 | 2353905 | 107 |
| exprReg_15817 | gi|126640115|ref|NC_009085.1| | 2354916 | 2354948 | 33 |
| exprReg_15818 | gi|126640115|ref|NC_009085.1| | 2354954 | 2355032 | 79 |
| exprReg_15819 | gi|126640115|ref|NC_009085.1| | 2355092 | 2355163 | 72 |
| exprReg_15820 | gi|126640115|ref|NC_009085.1| | 2355230 | 2355267 | 38 |
| exprReg_15821 | gi|126640115|ref|NC_009085.1| | 2355509 | 2355662 | 154 |
| exprReg_15822 | gi|126640115|ref|NC_009085.1| | 2355837 | 2355938 | 102 |
| exprReg_15823 | gi|126640115|ref|NC_009085.1| | 2356122 | 2356164 | 43 |
| exprReg_15847 | gi|126640115|ref|NC_009085.1| | 2361328 | 2361346 | 19 |
| exprReg_15849 | gi|126640115|ref|NC_009085.1| | 2361902 | 2362047 | 146 |
| exprReg_15850 | gi|126640115|ref|NC_009085.1| | 2362069 | 2362168 | 100 |
| exprReg_15851 | gi|126640115|ref|NC_009085.1| | 2362210 | 2362227 | 18 |
| exprReg_15852 | gi|126640115|ref|NC_009085.1| | 2362378 | 2362437 | 60 |
| exprReg_15853 | gi|126640115|ref|NC_009085.1| | 2362471 | 2362487 | 17 |
| exprReg_15854 | gi|126640115|ref|NC_009085.1| | 2362510 | 2362569 | 60 |
| exprReg_15855 | gi|126640115|ref|NC_009085.1| | 2362793 | 2362853 | 61 |
| exprReg_15856 | gi|126640115|ref|NC_009085.1| | 2362910 | 2362971 | 62 |
| exprReg_15857 | gi|126640115|ref|NC_009085.1| | 2362986 | 2363069 | 84 |
| exprReg_15858 | gi|126640115|ref|NC_009085.1| | 2363164 | 2363207 | 44 |
| exprReg_15859 | gi|126640115|ref|NC_009085.1| | 2363219 | 2363291 | 73 |
| exprReg_15860 | gi|126640115|ref|NC_009085.1| | 2363305 | 2363427 | 123 |
| exprReg_15861 | gi|126640115|ref|NC_009085.1| | 2363453 | 2363475 | 23 |
| exprReg_15862 | gi|126640115|ref|NC_009085.1| | 2363586 | 2363675 | 90 |
| exprReg_15863 | gi|126640115|ref|NC_009085.1| | 2363731 | 2363844 | 114 |
| exprReg_15864 | gi|126640115|ref|NC_009085.1| | 2363890 | 2363949 | 60 |
| exprReg_15865 | gi|126640115|ref|NC_009085.1| | 2364040 | 2364115 | 76 |
| exprReg_15869 | gi|126640115|ref|NC_009085.1| | 2364970 | 2364989 | 20 |
| exprReg_15870 | gi|126640115|ref|NC_009085.1| | 2365007 | 2365127 | 121 |
| exprReg_15871 | gi|126640115|ref|NC_009085.1| | 2365269 | 2365318 | 50 |
| exprReg_15872 | gi|126640115|ref|NC_009085.1| | 2365364 | 2365415 | 52 |
| exprReg_15873 | gi|126640115|ref|NC_009085.1| | 2365421 | 2365467 | 47 |
| exprReg_15874 | gi|126640115|ref|NC_009085.1| | 2365470 | 2365610 | 141 |
| exprReg_15875 | gi|126640115|ref|NC_009085.1| | 2365767 | 2365831 | 65 |
| exprReg_15876 | gi|126640115|ref|NC_009085.1| | 2365918 | 2365995 | 78 |
| exprReg_15877 | gi|126640115|ref|NC_009085.1| | 2366019 | 2366066 | 48 |
| exprReg_15880 | gi|126640115|ref|NC_009085.1| | 2367590 | 2367736 | 147 |
| exprReg_15881 | gi|126640115|ref|NC_009085.1| | 2367744 | 2367834 | 91 |
| exprReg_15882 | gi|126640115|ref|NC_009085.1| | 2367891 | 2367919 | 29 |
| exprReg_15883 | gi|126640115|ref|NC_009085.1| | 2368018 | 2368064 | 47 |
| exprReg_15884 | gi|126640115|ref|NC_009085.1| | 2368521 | 2368668 | 148 |
| exprReg_15891 | gi|126640115|ref|NC_009085.1| | 2370485 | 2370515 | 31 |
| exprReg_15892 | gi|126640115|ref|NC_009085.1| | 2370827 | 2370880 | 54 |
| exprReg_15893 | gi|126640115|ref|NC_009085.1| | 2371018 | 2371038 | 21 |
| exprReg_15894 | gi|126640115|ref|NC_009085.1| | 2371051 | 2371114 | 64 |
| exprReg_15895 | gi|126640115|ref|NC_009085.1| | 2371206 | 2371223 | 18 |
| exprReg_15896 | gi|126640115|ref|NC_009085.1| | 2371596 | 2371612 | 17 |
| exprReg_15897 | gi|126640115|ref|NC_009085.1| | 2371632 | 2371710 | 79 |
| exprReg_15898 | gi|126640115|ref|NC_009085.1| | 2371768 | 2371880 | 113 |
| exprReg_15906 | gi|126640115|ref|NC_009085.1| | 2372406 | 2372472 | 67 |
| exprReg_15907 | gi|126640115|ref|NC_009085.1| | 2372490 | 2372533 | 44 |
| exprReg_15908 | gi|126640115|ref|NC_009085.1| | 2372547 | 2372590 | 44 |
| exprReg_15909 | gi|126640115|ref|NC_009085.1| | 2372630 | 2372661 | 32 |
| exprReg_15910 | gi|126640115|ref|NC_009085.1| | 2372690 | 2372708 | 19 |
| exprReg_15911 | gi|126640115|ref|NC_009085.1| | 2372710 | 2372929 | 220 |
| exprReg_15912 | gi|126640115|ref|NC_009085.1| | 2372951 | 2373023 | 73 |
| exprReg_15913 | gi|126640115|ref|NC_009085.1| | 2373053 | 2373097 | 45 |
| exprReg_15914 | gi|126640115|ref|NC_009085.1| | 2373250 | 2373353 | 104 |
| exprReg_15915 | gi|126640115|ref|NC_009085.1| | 2373512 | 2373541 | 30 |
| exprReg_15916 | gi|126640115|ref|NC_009085.1| | 2373640 | 2373664 | 25 |
| exprReg_15917 | gi|126640115|ref|NC_009085.1| | 2373723 | 2373812 | 90 |
| exprReg_15918 | gi|126640115|ref|NC_009085.1| | 2373969 | 2374000 | 32 |
| exprReg_15919 | gi|126640115|ref|NC_009085.1| | 2374022 | 2374049 | 28 |
| exprReg_15920 | gi|126640115|ref|NC_009085.1| | 2374095 | 2374154 | 60 |
| exprReg_15921 | gi|126640115|ref|NC_009085.1| | 2374156 | 2374201 | 46 |
| exprReg_15925 | gi|126640115|ref|NC_009085.1| | 2374878 | 2374926 | 49 |
| exprReg_15934 | gi|126640115|ref|NC_009085.1| | 2376648 | 2376705 | 58 |
| exprReg_15935 | gi|126640115|ref|NC_009085.1| | 2376780 | 2376796 | 17 |
| exprReg_15936 | gi|126640115|ref|NC_009085.1| | 2376836 | 2376873 | 38 |
| exprReg_15937 | gi|126640115|ref|NC_009085.1| | 2376922 | 2376942 | 21 |
| exprReg_15938 | gi|126640115|ref|NC_009085.1| | 2377241 | 2377267 | 27 |
| exprReg_15939 | gi|126640115|ref|NC_009085.1| | 2377325 | 2377371 | 47 |
| exprReg_15940 | gi|126640115|ref|NC_009085.1| | 2377457 | 2377526 | 70 |
| exprReg_15941 | gi|126640115|ref|NC_009085.1| | 2377546 | 2377692 | 147 |
| exprReg_15942 | gi|126640115|ref|NC_009085.1| | 2377763 | 2378062 | 300 |
| exprReg_15943 | gi|126640115|ref|NC_009085.1| | 2378254 | 2378305 | 52 |
| exprReg_15944 | gi|126640115|ref|NC_009085.1| | 2378477 | 2378516 | 40 |
| exprReg_15945 | gi|126640115|ref|NC_009085.1| | 2378531 | 2378558 | 28 |
| exprReg_15946 | gi|126640115|ref|NC_009085.1| | 2378583 | 2378818 | 236 |
| exprReg_15947 | gi|126640115|ref|NC_009085.1| | 2378825 | 2378865 | 41 |
| exprReg_15948 | gi|126640115|ref|NC_009085.1| | 2378917 | 2378963 | 47 |
| exprReg_15954 | gi|126640115|ref|NC_009085.1| | 2380050 | 2380081 | 32 |
| exprReg_15955 | gi|126640115|ref|NC_009085.1| | 2380211 | 2380304 | 94 |
| exprReg_15956 | gi|126640115|ref|NC_009085.1| | 2380520 | 2380570 | 51 |
| exprReg_15963 | gi|126640115|ref|NC_009085.1| | 2381600 | 2381680 | 81 |
| exprReg_15964 | gi|126640115|ref|NC_009085.1| | 2381693 | 2381736 | 44 |
| exprReg_15965 | gi|126640115|ref|NC_009085.1| | 2381854 | 2381914 | 61 |
| exprReg_15966 | gi|126640115|ref|NC_009085.1| | 2381934 | 2381978 | 45 |
| exprReg_15974 | gi|126640115|ref|NC_009085.1| | 2382709 | 2382736 | 28 |
| exprReg_15975 | gi|126640115|ref|NC_009085.1| | 2383113 | 2383193 | 81 |
| exprReg_15976 | gi|126640115|ref|NC_009085.1| | 2383286 | 2383303 | 18 |
| exprReg_16004 | gi|126640115|ref|NC_009085.1| | 2387187 | 2387235 | 49 |
| exprReg_16005 | gi|126640115|ref|NC_009085.1| | 2387238 | 2387326 | 89 |
| exprReg_16012 | gi|126640115|ref|NC_009085.1| | 2387716 | 2387738 | 23 |
| exprReg_16021 | gi|126640115|ref|NC_009085.1| | 2388433 | 2388461 | 29 |
| exprReg_16031 | gi|126640115|ref|NC_009085.1| | 2389366 | 2389382 | 17 |
| exprReg_16037 | gi|126640115|ref|NC_009085.1| | 2389940 | 2389961 | 22 |
| exprReg_16038 | gi|126640115|ref|NC_009085.1| | 2390099 | 2390297 | 199 |
| exprReg_16050 | gi|126640115|ref|NC_009085.1| | 2391401 | 2391421 | 21 |
| exprReg_16051 | gi|126640115|ref|NC_009085.1| | 2391430 | 2391590 | 161 |
| exprReg_16052 | gi|126640115|ref|NC_009085.1| | 2391813 | 2391835 | 23 |
| exprReg_16053 | gi|126640115|ref|NC_009085.1| | 2391851 | 2391867 | 17 |
| exprReg_16066 | gi|126640115|ref|NC_009085.1| | 2393430 | 2393450 | 21 |
| exprReg_16082 | gi|126640115|ref|NC_009085.1| | 2395711 | 2395843 | 133 |
| exprReg_16083 | gi|126640115|ref|NC_009085.1| | 2395923 | 2395946 | 24 |
| exprReg_16106 | gi|126640115|ref|NC_009085.1| | 2397995 | 2398090 | 96 |
| exprReg_16107 | gi|126640115|ref|NC_009085.1| | 2398110 | 2398131 | 22 |
| exprReg_16117 | gi|126640115|ref|NC_009085.1| | 2399766 | 2399806 | 41 |
| exprReg_16118 | gi|126640115|ref|NC_009085.1| | 2399823 | 2399895 | 73 |
| exprReg_16130 | gi|126640115|ref|NC_009085.1| | 2401622 | 2401654 | 33 |
| exprReg_16131 | gi|126640115|ref|NC_009085.1| | 2401727 | 2401743 | 17 |
| exprReg_16132 | gi|126640115|ref|NC_009085.1| | 2401794 | 2401843 | 50 |
| exprReg_16137 | gi|126640115|ref|NC_009085.1| | 2402506 | 2402522 | 17 |
| exprReg_16138 | gi|126640115|ref|NC_009085.1| | 2402688 | 2402706 | 19 |
| exprReg_16150 | gi|126640115|ref|NC_009085.1| | 2404138 | 2404167 | 30 |
| exprReg_16151 | gi|126640115|ref|NC_009085.1| | 2404200 | 2404217 | 18 |
| exprReg_16152 | gi|126640115|ref|NC_009085.1| | 2404292 | 2404326 | 35 |
| exprReg_16153 | gi|126640115|ref|NC_009085.1| | 2404359 | 2404391 | 33 |
| exprReg_16154 | gi|126640115|ref|NC_009085.1| | 2404422 | 2404488 | 67 |
| exprReg_16155 | gi|126640115|ref|NC_009085.1| | 2404502 | 2404575 | 74 |
| exprReg_16175 | gi|126640115|ref|NC_009085.1| | 2406530 | 2406553 | 24 |
| exprReg_16176 | gi|126640115|ref|NC_009085.1| | 2406588 | 2406647 | 60 |
| exprReg_16199 | gi|126640115|ref|NC_009085.1| | 2410085 | 2410101 | 17 |
| exprReg_16205 | gi|126640115|ref|NC_009085.1| | 2411174 | 2411236 | 63 |
| exprReg_16224 | gi|126640115|ref|NC_009085.1| | 2414815 | 2414835 | 21 |
| exprReg_16225 | gi|126640115|ref|NC_009085.1| | 2414867 | 2414900 | 34 |
| exprReg_16226 | gi|126640115|ref|NC_009085.1| | 2414973 | 2415252 | 280 |
| exprReg_16229 | gi|126640115|ref|NC_009085.1| | 2415735 | 2415752 | 18 |
| exprReg_16230 | gi|126640115|ref|NC_009085.1| | 2416070 | 2416086 | 17 |
| exprReg_16231 | gi|126640115|ref|NC_009085.1| | 2416237 | 2416265 | 29 |
| exprReg_16232 | gi|126640115|ref|NC_009085.1| | 2416349 | 2416393 | 45 |
| exprReg_16233 | gi|126640115|ref|NC_009085.1| | 2416827 | 2416969 | 143 |
| exprReg_16236 | gi|126640115|ref|NC_009085.1| | 2417428 | 2417529 | 102 |
| exprReg_16237 | gi|126640115|ref|NC_009085.1| | 2417636 | 2417683 | 48 |
| exprReg_16248 | gi|126640115|ref|NC_009085.1| | 2419802 | 2419888 | 87 |
| exprReg_16249 | gi|126640115|ref|NC_009085.1| | 2419913 | 2419977 | 65 |
| exprReg_16250 | gi|126640115|ref|NC_009085.1| | 2420057 | 2420148 | 92 |
| exprReg_16256 | gi|126640115|ref|NC_009085.1| | 2421418 | 2421437 | 20 |
| exprReg_16257 | gi|126640115|ref|NC_009085.1| | 2421738 | 2421754 | 17 |
| exprReg_16267 | gi|126640115|ref|NC_009085.1| | 2422928 | 2423097 | 170 |
| exprReg_16279 | gi|126640115|ref|NC_009085.1| | 2425109 | 2425125 | 17 |
| exprReg_16280 | gi|126640115|ref|NC_009085.1| | 2425140 | 2425158 | 19 |
| exprReg_16281 | gi|126640115|ref|NC_009085.1| | 2425240 | 2425259 | 20 |
| exprReg_16282 | gi|126640115|ref|NC_009085.1| | 2425302 | 2425344 | 43 |
| exprReg_16283 | gi|126640115|ref|NC_009085.1| | 2425392 | 2425409 | 18 |
| exprReg_16284 | gi|126640115|ref|NC_009085.1| | 2425666 | 2425704 | 39 |
| exprReg_16285 | gi|126640115|ref|NC_009085.1| | 2426004 | 2426032 | 29 |
| exprReg_16286 | gi|126640115|ref|NC_009085.1| | 2426103 | 2426227 | 125 |
| exprReg_16287 | gi|126640115|ref|NC_009085.1| | 2426317 | 2426334 | 18 |
| exprReg_16288 | gi|126640115|ref|NC_009085.1| | 2426511 | 2426748 | 238 |
| exprReg_16289 | gi|126640115|ref|NC_009085.1| | 2426775 | 2426837 | 63 |
| exprReg_16290 | gi|126640115|ref|NC_009085.1| | 2426959 | 2427052 | 94 |
| exprReg_16302 | gi|126640115|ref|NC_009085.1| | 2428529 | 2428585 | 57 |
| exprReg_16303 | gi|126640115|ref|NC_009085.1| | 2428589 | 2428641 | 53 |
| exprReg_16304 | gi|126640115|ref|NC_009085.1| | 2428903 | 2428938 | 36 |
| exprReg_16323 | gi|126640115|ref|NC_009085.1| | 2431608 | 2431660 | 53 |
| exprReg_16324 | gi|126640115|ref|NC_009085.1| | 2431689 | 2431744 | 56 |
| exprReg_16325 | gi|126640115|ref|NC_009085.1| | 2432018 | 2432233 | 216 |
| exprReg_16326 | gi|126640115|ref|NC_009085.1| | 2432454 | 2432480 | 27 |
| exprReg_16327 | gi|126640115|ref|NC_009085.1| | 2432713 | 2432747 | 35 |
| exprReg_16328 | gi|126640115|ref|NC_009085.1| | 2432907 | 2432955 | 49 |
| exprReg_16329 | gi|126640115|ref|NC_009085.1| | 2433424 | 2433441 | 18 |
| exprReg_16330 | gi|126640115|ref|NC_009085.1| | 2433508 | 2433524 | 17 |
| exprReg_16338 | gi|126640115|ref|NC_009085.1| | 2434635 | 2434658 | 24 |
| exprReg_16341 | gi|126640115|ref|NC_009085.1| | 2435317 | 2435410 | 94 |
| exprReg_16349 | gi|126640115|ref|NC_009085.1| | 2436834 | 2436865 | 32 |
| exprReg_16350 | gi|126640115|ref|NC_009085.1| | 2436986 | 2437010 | 25 |
| exprReg_16351 | gi|126640115|ref|NC_009085.1| | 2437099 | 2437115 | 17 |
| exprReg_16360 | gi|126640115|ref|NC_009085.1| | 2437960 | 2437976 | 17 |
| exprReg_16379 | gi|126640115|ref|NC_009085.1| | 2440510 | 2440562 | 53 |
| exprReg_16380 | gi|126640115|ref|NC_009085.1| | 2440574 | 2440597 | 24 |
| exprReg_16391 | gi|126640115|ref|NC_009085.1| | 2441850 | 2441899 | 50 |
| exprReg_16411 | gi|126640115|ref|NC_009085.1| | 2445109 | 2445127 | 19 |
| exprReg_16412 | gi|126640115|ref|NC_009085.1| | 2445139 | 2445179 | 41 |
| exprReg_16413 | gi|126640115|ref|NC_009085.1| | 2445192 | 2445209 | 18 |
| exprReg_16414 | gi|126640115|ref|NC_009085.1| | 2445233 | 2445306 | 74 |
| exprReg_16416 | gi|126640115|ref|NC_009085.1| | 2445770 | 2445818 | 49 |
| exprReg_16453 | gi|126640115|ref|NC_009085.1| | 2450024 | 2450041 | 18 |
| exprReg_16454 | gi|126640115|ref|NC_009085.1| | 2450079 | 2450106 | 28 |
| exprReg_16455 | gi|126640115|ref|NC_009085.1| | 2450230 | 2450397 | 168 |
| exprReg_16462 | gi|126640115|ref|NC_009085.1| | 2451583 | 2451602 | 20 |
| exprReg_16463 | gi|126640115|ref|NC_009085.1| | 2451644 | 2451666 | 23 |
| exprReg_16469 | gi|126640115|ref|NC_009085.1| | 2452286 | 2452305 | 20 |
| exprReg_16470 | gi|126640115|ref|NC_009085.1| | 2452310 | 2452343 | 34 |
| exprReg_16471 | gi|126640115|ref|NC_009085.1| | 2452345 | 2452368 | 24 |
| exprReg_16486 | gi|126640115|ref|NC_009085.1| | 2453577 | 2453635 | 59 |
| exprReg_16487 | gi|126640115|ref|NC_009085.1| | 2453662 | 2454033 | 372 |
| exprReg_16488 | gi|126640115|ref|NC_009085.1| | 2454043 | 2454061 | 19 |
| exprReg_16489 | gi|126640115|ref|NC_009085.1| | 2454081 | 2454101 | 21 |
| exprReg_16490 | gi|126640115|ref|NC_009085.1| | 2454118 | 2454209 | 92 |
| exprReg_16493 | gi|126640115|ref|NC_009085.1| | 2455697 | 2455783 | 87 |
| exprReg_16500 | gi|126640115|ref|NC_009085.1| | 2457241 | 2457288 | 48 |
| exprReg_16509 | gi|126640115|ref|NC_009085.1| | 2458879 | 2458914 | 36 |
| exprReg_16514 | gi|126640115|ref|NC_009085.1| | 2459482 | 2459636 | 155 |
| exprReg_16521 | gi|126640115|ref|NC_009085.1| | 2460383 | 2460407 | 25 |
| exprReg_16522 | gi|126640115|ref|NC_009085.1| | 2460461 | 2460519 | 59 |
| exprReg_16531 | gi|126640115|ref|NC_009085.1| | 2462500 | 2462543 | 44 |
| exprReg_16532 | gi|126640115|ref|NC_009085.1| | 2462581 | 2462597 | 17 |
| exprReg_16533 | gi|126640115|ref|NC_009085.1| | 2462619 | 2462701 | 83 |
| exprReg_16538 | gi|126640115|ref|NC_009085.1| | 2463207 | 2463234 | 28 |
| exprReg_16546 | gi|126640115|ref|NC_009085.1| | 2463932 | 2463991 | 60 |
| exprReg_16547 | gi|126640115|ref|NC_009085.1| | 2464178 | 2464200 | 23 |
| exprReg_16550 | gi|126640115|ref|NC_009085.1| | 2464821 | 2464863 | 43 |
| exprReg_16557 | gi|126640115|ref|NC_009085.1| | 2465826 | 2466001 | 176 |
| exprReg_16558 | gi|126640115|ref|NC_009085.1| | 2466008 | 2466238 | 231 |
| exprReg_16559 | gi|126640115|ref|NC_009085.1| | 2466256 | 2466360 | 105 |
| exprReg_16560 | gi|126640115|ref|NC_009085.1| | 2466371 | 2466433 | 63 |
| exprReg_16574 | gi|126640115|ref|NC_009085.1| | 2468535 | 2468556 | 22 |
| exprReg_16575 | gi|126640115|ref|NC_009085.1| | 2468565 | 2468621 | 57 |
| exprReg_16576 | gi|126640115|ref|NC_009085.1| | 2468633 | 2468652 | 20 |
| exprReg_16577 | gi|126640115|ref|NC_009085.1| | 2468656 | 2468686 | 31 |
| exprReg_16588 | gi|126640115|ref|NC_009085.1| | 2469986 | 2470021 | 36 |
| exprReg_16592 | gi|126640115|ref|NC_009085.1| | 2470970 | 2471000 | 31 |
| exprReg_16605 | gi|126640115|ref|NC_009085.1| | 2472482 | 2472585 | 104 |
| exprReg_16606 | gi|126640115|ref|NC_009085.1| | 2472659 | 2472778 | 120 |
| exprReg_16607 | gi|126640115|ref|NC_009085.1| | 2472815 | 2472835 | 21 |
| exprReg_16608 | gi|126640115|ref|NC_009085.1| | 2472873 | 2472890 | 18 |
| exprReg_16609 | gi|126640115|ref|NC_009085.1| | 2472899 | 2472916 | 18 |
| exprReg_16610 | gi|126640115|ref|NC_009085.1| | 2472918 | 2472937 | 20 |
| exprReg_16622 | gi|126640115|ref|NC_009085.1| | 2474472 | 2474501 | 30 |
| exprReg_16624 | gi|126640115|ref|NC_009085.1| | 2475758 | 2475850 | 93 |
| exprReg_16625 | gi|126640115|ref|NC_009085.1| | 2475930 | 2475954 | 25 |
| exprReg_16626 | gi|126640115|ref|NC_009085.1| | 2476163 | 2476227 | 65 |
| exprReg_16644 | gi|126640115|ref|NC_009085.1| | 2478681 | 2479169 | 489 |
| exprReg_16645 | gi|126640115|ref|NC_009085.1| | 2479188 | 2479205 | 18 |
| exprReg_16661 | gi|126640115|ref|NC_009085.1| | 2481864 | 2482048 | 185 |
| exprReg_16662 | gi|126640115|ref|NC_009085.1| | 2482092 | 2482164 | 73 |
| exprReg_16663 | gi|126640115|ref|NC_009085.1| | 2482186 | 2482228 | 43 |
| exprReg_16705 | gi|126640115|ref|NC_009085.1| | 2487149 | 2487251 | 103 |
| exprReg_16723 | gi|126640115|ref|NC_009085.1| | 2490084 | 2490217 | 134 |
| exprReg_16724 | gi|126640115|ref|NC_009085.1| | 2490221 | 2490336 | 116 |
| exprReg_16733 | gi|126640115|ref|NC_009085.1| | 2491386 | 2491411 | 26 |
| exprReg_16734 | gi|126640115|ref|NC_009085.1| | 2491414 | 2491447 | 34 |
| exprReg_16735 | gi|126640115|ref|NC_009085.1| | 2491450 | 2491579 | 130 |
| exprReg_16736 | gi|126640115|ref|NC_009085.1| | 2491586 | 2491635 | 50 |
| exprReg_16747 | gi|126640115|ref|NC_009085.1| | 2492908 | 2492941 | 34 |
| exprReg_16748 | gi|126640115|ref|NC_009085.1| | 2493125 | 2493141 | 17 |
| exprReg_16760 | gi|126640115|ref|NC_009085.1| | 2495209 | 2495239 | 31 |
| exprReg_16761 | gi|126640115|ref|NC_009085.1| | 2495313 | 2495344 | 32 |
| exprReg_16780 | gi|126640115|ref|NC_009085.1| | 2497929 | 2498035 | 107 |
| exprReg_16810 | gi|126640115|ref|NC_009085.1| | 2502367 | 2502524 | 158 |
| exprReg_16811 | gi|126640115|ref|NC_009085.1| | 2502526 | 2502632 | 107 |
| exprReg_16831 | gi|126640115|ref|NC_009085.1| | 2504666 | 2504744 | 79 |
| exprReg_16855 | gi|126640115|ref|NC_009085.1| | 2508074 | 2508121 | 48 |
| exprReg_16856 | gi|126640115|ref|NC_009085.1| | 2508221 | 2508247 | 27 |
| exprReg_16873 | gi|126640115|ref|NC_009085.1| | 2510119 | 2510151 | 33 |
| exprReg_16874 | gi|126640115|ref|NC_009085.1| | 2510194 | 2510218 | 25 |
| exprReg_16876 | gi|126640115|ref|NC_009085.1| | 2510596 | 2510722 | 127 |
| exprReg_16898 | gi|126640115|ref|NC_009085.1| | 2514504 | 2514539 | 36 |
| exprReg_16899 | gi|126640115|ref|NC_009085.1| | 2514635 | 2514656 | 22 |
| exprReg_16903 | gi|126640115|ref|NC_009085.1| | 2515445 | 2515479 | 35 |
| exprReg_16908 | gi|126640115|ref|NC_009085.1| | 2516157 | 2516263 | 107 |
| exprReg_16909 | gi|126640115|ref|NC_009085.1| | 2516280 | 2516331 | 52 |
| exprReg_16910 | gi|126640115|ref|NC_009085.1| | 2516333 | 2516638 | 306 |
| exprReg_16911 | gi|126640115|ref|NC_009085.1| | 2516642 | 2516676 | 35 |
| exprReg_16912 | gi|126640115|ref|NC_009085.1| | 2516685 | 2516740 | 56 |
| exprReg_16922 | gi|126640115|ref|NC_009085.1| | 2518582 | 2518626 | 45 |
| exprReg_16923 | gi|126640115|ref|NC_009085.1| | 2518712 | 2518734 | 23 |
| exprReg_16924 | gi|126640115|ref|NC_009085.1| | 2518746 | 2518763 | 18 |
| exprReg_16925 | gi|126640115|ref|NC_009085.1| | 2518843 | 2518864 | 22 |
| exprReg_16934 | gi|126640115|ref|NC_009085.1| | 2520257 | 2520274 | 18 |
| exprReg_16949 | gi|126640115|ref|NC_009085.1| | 2521874 | 2521899 | 26 |
| exprReg_16968 | gi|126640115|ref|NC_009085.1| | 2524383 | 2524414 | 32 |
| exprReg_16969 | gi|126640115|ref|NC_009085.1| | 2524442 | 2524469 | 28 |
| exprReg_16970 | gi|126640115|ref|NC_009085.1| | 2524493 | 2524531 | 39 |
| exprReg_16971 | gi|126640115|ref|NC_009085.1| | 2524577 | 2524601 | 25 |
| exprReg_16972 | gi|126640115|ref|NC_009085.1| | 2524807 | 2524907 | 101 |
| exprReg_16981 | gi|126640115|ref|NC_009085.1| | 2525800 | 2525816 | 17 |
| exprReg_16982 | gi|126640115|ref|NC_009085.1| | 2525838 | 2525897 | 60 |
| exprReg_17002 | gi|126640115|ref|NC_009085.1| | 2528348 | 2528365 | 18 |
| exprReg_17003 | gi|126640115|ref|NC_009085.1| | 2528423 | 2528442 | 20 |
| exprReg_17040 | gi|126640115|ref|NC_009085.1| | 2533516 | 2533572 | 57 |
| exprReg_17053 | gi|126640115|ref|NC_009085.1| | 2535695 | 2535713 | 19 |
| exprReg_17054 | gi|126640115|ref|NC_009085.1| | 2535763 | 2535787 | 25 |
| exprReg_17055 | gi|126640115|ref|NC_009085.1| | 2535805 | 2535825 | 21 |
| exprReg_17056 | gi|126640115|ref|NC_009085.1| | 2535857 | 2535914 | 58 |
| exprReg_17062 | gi|126640115|ref|NC_009085.1| | 2536456 | 2536472 | 17 |
| exprReg_17079 | gi|126640115|ref|NC_009085.1| | 2538388 | 2538408 | 21 |
| exprReg_17084 | gi|126640115|ref|NC_009085.1| | 2538980 | 2539089 | 110 |
| exprReg_17085 | gi|126640115|ref|NC_009085.1| | 2539104 | 2539148 | 45 |
| exprReg_17108 | gi|126640115|ref|NC_009085.1| | 2541998 | 2542080 | 83 |
| exprReg_17109 | gi|126640115|ref|NC_009085.1| | 2542122 | 2542177 | 56 |
| exprReg_17110 | gi|126640115|ref|NC_009085.1| | 2542195 | 2542222 | 28 |
| exprReg_17111 | gi|126640115|ref|NC_009085.1| | 2542308 | 2542441 | 134 |
| exprReg_17118 | gi|126640115|ref|NC_009085.1| | 2543450 | 2543467 | 18 |
| exprReg_17119 | gi|126640115|ref|NC_009085.1| | 2543555 | 2543573 | 19 |
| exprReg_17130 | gi|126640115|ref|NC_009085.1| | 2545571 | 2545623 | 53 |
| exprReg_17147 | gi|126640115|ref|NC_009085.1| | 2548075 | 2548168 | 94 |
| exprReg_17148 | gi|126640115|ref|NC_009085.1| | 2548210 | 2548254 | 45 |
| exprReg_17149 | gi|126640115|ref|NC_009085.1| | 2548259 | 2548374 | 116 |
| exprReg_17152 | gi|126640115|ref|NC_009085.1| | 2548668 | 2548687 | 20 |
| exprReg_17153 | gi|126640115|ref|NC_009085.1| | 2548707 | 2548727 | 21 |
| exprReg_17179 | gi|126640115|ref|NC_009085.1| | 2552956 | 2552972 | 17 |
| exprReg_17180 | gi|126640115|ref|NC_009085.1| | 2553028 | 2553067 | 40 |
| exprReg_17181 | gi|126640115|ref|NC_009085.1| | 2553092 | 2553120 | 29 |
| exprReg_17192 | gi|126640115|ref|NC_009085.1| | 2554738 | 2554829 | 92 |
| exprReg_17200 | gi|126640115|ref|NC_009085.1| | 2556187 | 2556204 | 18 |
| exprReg_17210 | gi|126640115|ref|NC_009085.1| | 2557475 | 2557494 | 20 |
| exprReg_17211 | gi|126640115|ref|NC_009085.1| | 2557531 | 2557548 | 18 |
| exprReg_17212 | gi|126640115|ref|NC_009085.1| | 2557575 | 2557591 | 17 |
| exprReg_17213 | gi|126640115|ref|NC_009085.1| | 2557655 | 2557684 | 30 |
| exprReg_17222 | gi|126640115|ref|NC_009085.1| | 2559828 | 2559909 | 82 |
| exprReg_17233 | gi|126640115|ref|NC_009085.1| | 2561754 | 2561779 | 26 |
| exprReg_17234 | gi|126640115|ref|NC_009085.1| | 2561827 | 2561849 | 23 |
| exprReg_17235 | gi|126640115|ref|NC_009085.1| | 2561897 | 2561920 | 24 |
| exprReg_17236 | gi|126640115|ref|NC_009085.1| | 2562283 | 2562303 | 21 |
| exprReg_17248 | gi|126640115|ref|NC_009085.1| | 2563993 | 2564012 | 20 |
| exprReg_17249 | gi|126640115|ref|NC_009085.1| | 2564074 | 2564126 | 53 |
| exprReg_17250 | gi|126640115|ref|NC_009085.1| | 2564389 | 2564501 | 113 |
| exprReg_17278 | gi|126640115|ref|NC_009085.1| | 2568537 | 2568555 | 19 |
| exprReg_17287 | gi|126640115|ref|NC_009085.1| | 2570311 | 2570371 | 61 |
| exprReg_17288 | gi|126640115|ref|NC_009085.1| | 2570483 | 2570804 | 322 |
| exprReg_17289 | gi|126640115|ref|NC_009085.1| | 2571076 | 2571096 | 21 |
| exprReg_17295 | gi|126640115|ref|NC_009085.1| | 2572497 | 2572520 | 24 |
| exprReg_17297 | gi|126640115|ref|NC_009085.1| | 2573203 | 2573251 | 49 |
| exprReg_17298 | gi|126640115|ref|NC_009085.1| | 2573396 | 2573517 | 122 |
| exprReg_17299 | gi|126640115|ref|NC_009085.1| | 2573519 | 2573540 | 22 |
| exprReg_17310 | gi|126640115|ref|NC_009085.1| | 2575136 | 2575181 | 46 |
| exprReg_17311 | gi|126640115|ref|NC_009085.1| | 2575295 | 2575368 | 74 |
| exprReg_17312 | gi|126640115|ref|NC_009085.1| | 2575428 | 2575463 | 36 |
| exprReg_17313 | gi|126640115|ref|NC_009085.1| | 2575499 | 2575601 | 103 |
| exprReg_17364 | gi|126640115|ref|NC_009085.1| | 2581135 | 2581155 | 21 |
| exprReg_17366 | gi|126640115|ref|NC_009085.1| | 2581804 | 2581928 | 125 |
| exprReg_17367 | gi|126640115|ref|NC_009085.1| | 2582452 | 2582473 | 22 |
| exprReg_17368 | gi|126640115|ref|NC_009085.1| | 2582478 | 2582494 | 17 |
| exprReg_17369 | gi|126640115|ref|NC_009085.1| | 2582571 | 2582594 | 24 |
| exprReg_17370 | gi|126640115|ref|NC_009085.1| | 2582613 | 2582630 | 18 |
| exprReg_17371 | gi|126640115|ref|NC_009085.1| | 2582675 | 2582695 | 21 |
| exprReg_17372 | gi|126640115|ref|NC_009085.1| | 2582697 | 2582716 | 20 |
| exprReg_17373 | gi|126640115|ref|NC_009085.1| | 2582730 | 2582803 | 74 |
| exprReg_17374 | gi|126640115|ref|NC_009085.1| | 2582990 | 2583033 | 44 |
| exprReg_17375 | gi|126640115|ref|NC_009085.1| | 2583089 | 2583106 | 18 |
| exprReg_17376 | gi|126640115|ref|NC_009085.1| | 2583108 | 2583179 | 72 |
| exprReg_17383 | gi|126640115|ref|NC_009085.1| | 2583909 | 2583930 | 22 |
| exprReg_17384 | gi|126640115|ref|NC_009085.1| | 2584112 | 2584133 | 22 |
| exprReg_17395 | gi|126640115|ref|NC_009085.1| | 2586173 | 2586221 | 49 |
| exprReg_17396 | gi|126640115|ref|NC_009085.1| | 2586224 | 2586273 | 50 |
| exprReg_17397 | gi|126640115|ref|NC_009085.1| | 2586414 | 2586435 | 22 |
| exprReg_17398 | gi|126640115|ref|NC_009085.1| | 2586570 | 2586589 | 20 |
| exprReg_17404 | gi|126640115|ref|NC_009085.1| | 2587772 | 2587813 | 42 |
| exprReg_17412 | gi|126640115|ref|NC_009085.1| | 2589604 | 2589623 | 20 |
| exprReg_17413 | gi|126640115|ref|NC_009085.1| | 2589776 | 2589889 | 114 |
| exprReg_17414 | gi|126640115|ref|NC_009085.1| | 2590131 | 2590288 | 158 |
| exprReg_17417 | gi|126640115|ref|NC_009085.1| | 2590657 | 2590734 | 78 |
| exprReg_17418 | gi|126640115|ref|NC_009085.1| | 2590969 | 2591065 | 97 |
| exprReg_17419 | gi|126640115|ref|NC_009085.1| | 2591100 | 2591130 | 31 |
| exprReg_17420 | gi|126640115|ref|NC_009085.1| | 2591139 | 2591189 | 51 |
| exprReg_17421 | gi|126640115|ref|NC_009085.1| | 2591260 | 2591303 | 44 |
| exprReg_17422 | gi|126640115|ref|NC_009085.1| | 2591380 | 2591441 | 62 |
| exprReg_17423 | gi|126640115|ref|NC_009085.1| | 2591464 | 2591484 | 21 |
| exprReg_17427 | gi|126640115|ref|NC_009085.1| | 2592175 | 2592195 | 21 |
| exprReg_17446 | gi|126640115|ref|NC_009085.1| | 2594516 | 2594564 | 49 |
| exprReg_17450 | gi|126640115|ref|NC_009085.1| | 2595086 | 2595114 | 29 |
| exprReg_17457 | gi|126640115|ref|NC_009085.1| | 2596512 | 2596551 | 40 |
| exprReg_17467 | gi|126640115|ref|NC_009085.1| | 2598193 | 2598225 | 33 |
| exprReg_17476 | gi|126640115|ref|NC_009085.1| | 2599852 | 2599870 | 19 |
| exprReg_17477 | gi|126640115|ref|NC_009085.1| | 2599901 | 2599955 | 55 |
| exprReg_17506 | gi|126640115|ref|NC_009085.1| | 2603735 | 2603834 | 100 |
| exprReg_17515 | gi|126640115|ref|NC_009085.1| | 2604833 | 2604947 | 115 |
| exprReg_17516 | gi|126640115|ref|NC_009085.1| | 2605060 | 2605082 | 23 |
| exprReg_17517 | gi|126640115|ref|NC_009085.1| | 2605097 | 2605127 | 31 |
| exprReg_17518 | gi|126640115|ref|NC_009085.1| | 2605151 | 2605176 | 26 |
| exprReg_17519 | gi|126640115|ref|NC_009085.1| | 2605190 | 2605206 | 17 |
| exprReg_17531 | gi|126640115|ref|NC_009085.1| | 2606865 | 2607055 | 191 |
| exprReg_17545 | gi|126640115|ref|NC_009085.1| | 2609198 | 2609227 | 30 |
| exprReg_17546 | gi|126640115|ref|NC_009085.1| | 2609244 | 2609265 | 22 |
| exprReg_17547 | gi|126640115|ref|NC_009085.1| | 2609303 | 2609357 | 55 |
| exprReg_17548 | gi|126640115|ref|NC_009085.1| | 2609422 | 2609458 | 37 |
| exprReg_17549 | gi|126640115|ref|NC_009085.1| | 2609481 | 2609500 | 20 |
| exprReg_17557 | gi|126640115|ref|NC_009085.1| | 2610400 | 2610431 | 32 |
| exprReg_17558 | gi|126640115|ref|NC_009085.1| | 2610438 | 2610467 | 30 |
| exprReg_17562 | gi|126640115|ref|NC_009085.1| | 2611077 | 2611118 | 42 |
| exprReg_17563 | gi|126640115|ref|NC_009085.1| | 2611129 | 2611161 | 33 |
| exprReg_17564 | gi|126640115|ref|NC_009085.1| | 2611181 | 2611217 | 37 |
| exprReg_17582 | gi|126640115|ref|NC_009085.1| | 2613119 | 2613142 | 24 |
| exprReg_17583 | gi|126640115|ref|NC_009085.1| | 2613186 | 2613206 | 21 |
| exprReg_17628 | gi|126640115|ref|NC_009085.1| | 2618491 | 2618528 | 38 |
| exprReg_17636 | gi|126640115|ref|NC_009085.1| | 2619591 | 2619623 | 33 |
| exprReg_17642 | gi|126640115|ref|NC_009085.1| | 2620144 | 2620160 | 17 |
| exprReg_17643 | gi|126640115|ref|NC_009085.1| | 2620194 | 2620379 | 186 |
| exprReg_17644 | gi|126640115|ref|NC_009085.1| | 2620476 | 2620509 | 34 |
| exprReg_17661 | gi|126640115|ref|NC_009085.1| | 2622500 | 2622576 | 77 |
| exprReg_17662 | gi|126640115|ref|NC_009085.1| | 2622739 | 2622791 | 53 |
| exprReg_17663 | gi|126640115|ref|NC_009085.1| | 2622884 | 2622923 | 40 |
| exprReg_17664 | gi|126640115|ref|NC_009085.1| | 2622947 | 2622971 | 25 |
| exprReg_17667 | gi|126640115|ref|NC_009085.1| | 2624017 | 2624037 | 21 |
| exprReg_17693 | gi|126640115|ref|NC_009085.1| | 2626949 | 2627009 | 61 |
| exprReg_17707 | gi|126640115|ref|NC_009085.1| | 2629444 | 2629492 | 49 |
| exprReg_17708 | gi|126640115|ref|NC_009085.1| | 2629505 | 2629557 | 53 |
| exprReg_17709 | gi|126640115|ref|NC_009085.1| | 2629565 | 2629616 | 52 |
| exprReg_17710 | gi|126640115|ref|NC_009085.1| | 2629625 | 2629649 | 25 |
| exprReg_17711 | gi|126640115|ref|NC_009085.1| | 2629651 | 2629678 | 28 |
| exprReg_17712 | gi|126640115|ref|NC_009085.1| | 2629742 | 2629761 | 20 |
| exprReg_17719 | gi|126640115|ref|NC_009085.1| | 2630761 | 2630778 | 18 |
| exprReg_17720 | gi|126640115|ref|NC_009085.1| | 2630781 | 2630797 | 17 |
| exprReg_17730 | gi|126640115|ref|NC_009085.1| | 2632120 | 2632138 | 19 |
| exprReg_17731 | gi|126640115|ref|NC_009085.1| | 2632161 | 2632253 | 93 |
| exprReg_17732 | gi|126640115|ref|NC_009085.1| | 2632321 | 2632455 | 135 |
| exprReg_17737 | gi|126640115|ref|NC_009085.1| | 2632925 | 2632948 | 24 |
| exprReg_17738 | gi|126640115|ref|NC_009085.1| | 2632983 | 2633120 | 138 |
| exprReg_17754 | gi|126640115|ref|NC_009085.1| | 2635574 | 2635591 | 18 |
| exprReg_17755 | gi|126640115|ref|NC_009085.1| | 2635611 | 2635701 | 91 |
| exprReg_17777 | gi|126640115|ref|NC_009085.1| | 2638672 | 2638719 | 48 |
| exprReg_17790 | gi|126640115|ref|NC_009085.1| | 2639640 | 2639662 | 23 |
| exprReg_17791 | gi|126640115|ref|NC_009085.1| | 2639683 | 2639729 | 47 |
| exprReg_17792 | gi|126640115|ref|NC_009085.1| | 2639753 | 2639951 | 199 |
| exprReg_17793 | gi|126640115|ref|NC_009085.1| | 2639999 | 2640028 | 30 |
| exprReg_17794 | gi|126640115|ref|NC_009085.1| | 2640046 | 2640068 | 23 |
| exprReg_17795 | gi|126640115|ref|NC_009085.1| | 2640078 | 2640097 | 20 |
| exprReg_17796 | gi|126640115|ref|NC_009085.1| | 2640130 | 2640188 | 59 |
| exprReg_17797 | gi|126640115|ref|NC_009085.1| | 2640197 | 2640239 | 43 |
| exprReg_17810 | gi|126640115|ref|NC_009085.1| | 2642122 | 2642142 | 21 |
| exprReg_17811 | gi|126640115|ref|NC_009085.1| | 2642196 | 2642224 | 29 |
| exprReg_17812 | gi|126640115|ref|NC_009085.1| | 2642292 | 2642312 | 21 |
| exprReg_17823 | gi|126640115|ref|NC_009085.1| | 2643532 | 2643553 | 22 |
| exprReg_17824 | gi|126640115|ref|NC_009085.1| | 2643626 | 2643676 | 51 |
| exprReg_17825 | gi|126640115|ref|NC_009085.1| | 2643741 | 2643762 | 22 |
| exprReg_17826 | gi|126640115|ref|NC_009085.1| | 2643795 | 2643822 | 28 |
| exprReg_17827 | gi|126640115|ref|NC_009085.1| | 2643929 | 2643982 | 54 |
| exprReg_17828 | gi|126640115|ref|NC_009085.1| | 2644114 | 2644151 | 38 |
| exprReg_17829 | gi|126640115|ref|NC_009085.1| | 2644161 | 2644188 | 28 |
| exprReg_17830 | gi|126640115|ref|NC_009085.1| | 2644203 | 2644221 | 19 |
| exprReg_17831 | gi|126640115|ref|NC_009085.1| | 2644241 | 2644264 | 24 |
| exprReg_17850 | gi|126640115|ref|NC_009085.1| | 2646448 | 2646597 | 150 |
| exprReg_17851 | gi|126640115|ref|NC_009085.1| | 2646666 | 2646846 | 181 |
| exprReg_17879 | gi|126640115|ref|NC_009085.1| | 2650744 | 2650987 | 244 |
| exprReg_17897 | gi|126640115|ref|NC_009085.1| | 2652834 | 2652919 | 86 |
| exprReg_17898 | gi|126640115|ref|NC_009085.1| | 2652939 | 2652964 | 26 |
| exprReg_17899 | gi|126640115|ref|NC_009085.1| | 2652986 | 2653052 | 67 |
| exprReg_17900 | gi|126640115|ref|NC_009085.1| | 2653080 | 2653119 | 40 |
| exprReg_17912 | gi|126640115|ref|NC_009085.1| | 2655496 | 2655516 | 21 |
| exprReg_17936 | gi|126640115|ref|NC_009085.1| | 2659644 | 2659661 | 18 |
| exprReg_17960 | gi|126640115|ref|NC_009085.1| | 2662951 | 2663065 | 115 |
| exprReg_17974 | gi|126640115|ref|NC_009085.1| | 2665485 | 2665547 | 63 |
| exprReg_17975 | gi|126640115|ref|NC_009085.1| | 2665592 | 2665656 | 65 |
| exprReg_17976 | gi|126640115|ref|NC_009085.1| | 2665658 | 2665676 | 19 |
| exprReg_17984 | gi|126640115|ref|NC_009085.1| | 2666411 | 2666486 | 76 |
| exprReg_17991 | gi|126640115|ref|NC_009085.1| | 2667727 | 2667743 | 17 |
| exprReg_18001 | gi|126640115|ref|NC_009085.1| | 2669265 | 2669305 | 41 |
| exprReg_18023 | gi|126640115|ref|NC_009085.1| | 2672389 | 2672417 | 29 |
| exprReg_18051 | gi|126640115|ref|NC_009085.1| | 2676453 | 2676742 | 290 |
| exprReg_18058 | gi|126640115|ref|NC_009085.1| | 2677884 | 2677903 | 20 |
| exprReg_18060 | gi|126640115|ref|NC_009085.1| | 2678518 | 2678538 | 21 |
| exprReg_18098 | gi|126640115|ref|NC_009085.1| | 2683074 | 2683104 | 31 |
| exprReg_18108 | gi|126640115|ref|NC_009085.1| | 2684203 | 2684230 | 28 |
| exprReg_18109 | gi|126640115|ref|NC_009085.1| | 2684247 | 2684337 | 91 |
| exprReg_18115 | gi|126640115|ref|NC_009085.1| | 2685208 | 2685228 | 21 |
| exprReg_18116 | gi|126640115|ref|NC_009085.1| | 2685441 | 2685459 | 19 |
| exprReg_18117 | gi|126640115|ref|NC_009085.1| | 2685485 | 2685537 | 53 |
| exprReg_18118 | gi|126640115|ref|NC_009085.1| | 2685621 | 2685657 | 37 |
| exprReg_18124 | gi|126640115|ref|NC_009085.1| | 2686060 | 2686108 | 49 |
| exprReg_18131 | gi|126640115|ref|NC_009085.1| | 2687694 | 2687834 | 141 |
| exprReg_18132 | gi|126640115|ref|NC_009085.1| | 2687839 | 2687901 | 63 |
| exprReg_18133 | gi|126640115|ref|NC_009085.1| | 2687939 | 2687989 | 51 |
| exprReg_18138 | gi|126640115|ref|NC_009085.1| | 2688576 | 2688614 | 39 |
| exprReg_18149 | gi|126640115|ref|NC_009085.1| | 2689987 | 2690007 | 21 |
| exprReg_18150 | gi|126640115|ref|NC_009085.1| | 2690051 | 2690131 | 81 |
| exprReg_18151 | gi|126640115|ref|NC_009085.1| | 2690136 | 2690152 | 17 |
| exprReg_18152 | gi|126640115|ref|NC_009085.1| | 2690203 | 2690537 | 335 |
| exprReg_18153 | gi|126640115|ref|NC_009085.1| | 2690595 | 2690611 | 17 |
| exprReg_18161 | gi|126640115|ref|NC_009085.1| | 2691593 | 2691753 | 161 |
| exprReg_18170 | gi|126640115|ref|NC_009085.1| | 2692995 | 2693032 | 38 |
| exprReg_18182 | gi|126640115|ref|NC_009085.1| | 2694742 | 2694771 | 30 |
| exprReg_18202 | gi|126640115|ref|NC_009085.1| | 2697485 | 2697559 | 75 |
| exprReg_18203 | gi|126640115|ref|NC_009085.1| | 2697585 | 2697614 | 30 |
| exprReg_18204 | gi|126640115|ref|NC_009085.1| | 2697620 | 2697645 | 26 |
| exprReg_18205 | gi|126640115|ref|NC_009085.1| | 2697668 | 2697690 | 23 |
| exprReg_18211 | gi|126640115|ref|NC_009085.1| | 2698886 | 2698902 | 17 |
| exprReg_18212 | gi|126640115|ref|NC_009085.1| | 2698958 | 2699055 | 98 |
| exprReg_18213 | gi|126640115|ref|NC_009085.1| | 2699121 | 2699145 | 25 |
| exprReg_18219 | gi|126640115|ref|NC_009085.1| | 2699773 | 2699937 | 165 |
| exprReg_18220 | gi|126640115|ref|NC_009085.1| | 2699965 | 2700008 | 44 |
| exprReg_18232 | gi|126640115|ref|NC_009085.1| | 2701560 | 2701596 | 37 |
| exprReg_18240 | gi|126640115|ref|NC_009085.1| | 2702523 | 2702593 | 71 |
| exprReg_18241 | gi|126640115|ref|NC_009085.1| | 2702617 | 2702633 | 17 |
| exprReg_18242 | gi|126640115|ref|NC_009085.1| | 2702640 | 2702740 | 101 |
| exprReg_18257 | gi|126640115|ref|NC_009085.1| | 2705229 | 2705312 | 84 |
| exprReg_18260 | gi|126640115|ref|NC_009085.1| | 2705935 | 2706076 | 142 |
| exprReg_18261 | gi|126640115|ref|NC_009085.1| | 2706100 | 2706121 | 22 |
| exprReg_18279 | gi|126640115|ref|NC_009085.1| | 2708961 | 2709014 | 54 |
| exprReg_18311 | gi|126640115|ref|NC_009085.1| | 2713507 | 2713523 | 17 |
| exprReg_18312 | gi|126640115|ref|NC_009085.1| | 2713531 | 2713567 | 37 |
| exprReg_18318 | gi|126640115|ref|NC_009085.1| | 2714021 | 2714061 | 41 |
| exprReg_18319 | gi|126640115|ref|NC_009085.1| | 2714133 | 2714250 | 118 |
| exprReg_18322 | gi|126640115|ref|NC_009085.1| | 2714860 | 2714891 | 32 |
| exprReg_18329 | gi|126640115|ref|NC_009085.1| | 2716287 | 2716310 | 24 |
| exprReg_18342 | gi|126640115|ref|NC_009085.1| | 2718331 | 2718348 | 18 |
| exprReg_18353 | gi|126640115|ref|NC_009085.1| | 2719869 | 2719937 | 69 |
| exprReg_18365 | gi|126640115|ref|NC_009085.1| | 2721473 | 2721598 | 126 |
| exprReg_18366 | gi|126640115|ref|NC_009085.1| | 2721668 | 2721782 | 115 |
| exprReg_18397 | gi|126640115|ref|NC_009085.1| | 2725543 | 2725641 | 99 |
| exprReg_18398 | gi|126640115|ref|NC_009085.1| | 2725658 | 2725677 | 20 |
| exprReg_18399 | gi|126640115|ref|NC_009085.1| | 2725816 | 2725837 | 22 |
| exprReg_18434 | gi|126640115|ref|NC_009085.1| | 2732281 | 2732427 | 147 |
| exprReg_18442 | gi|126640115|ref|NC_009085.1| | 2733430 | 2733480 | 51 |
| exprReg_18451 | gi|126640115|ref|NC_009085.1| | 2734326 | 2734390 | 65 |
| exprReg_18463 | gi|126640115|ref|NC_009085.1| | 2735887 | 2735924 | 38 |
| exprReg_18478 | gi|126640115|ref|NC_009085.1| | 2738248 | 2738271 | 24 |
| exprReg_18489 | gi|126640115|ref|NC_009085.1| | 2739802 | 2739925 | 124 |
| exprReg_18490 | gi|126640115|ref|NC_009085.1| | 2740008 | 2740063 | 56 |
| exprReg_18491 | gi|126640115|ref|NC_009085.1| | 2740098 | 2740182 | 85 |
| exprReg_18497 | gi|126640115|ref|NC_009085.1| | 2740872 | 2740909 | 38 |
| exprReg_18511 | gi|126640115|ref|NC_009085.1| | 2743815 | 2743957 | 143 |
| exprReg_18512 | gi|126640115|ref|NC_009085.1| | 2743976 | 2744050 | 75 |
| exprReg_18513 | gi|126640115|ref|NC_009085.1| | 2744066 | 2744131 | 66 |
| exprReg_18514 | gi|126640115|ref|NC_009085.1| | 2744146 | 2744176 | 31 |
| exprReg_18515 | gi|126640115|ref|NC_009085.1| | 2744185 | 2744208 | 24 |
| exprReg_18516 | gi|126640115|ref|NC_009085.1| | 2744216 | 2744237 | 22 |
| exprReg_18517 | gi|126640115|ref|NC_009085.1| | 2744291 | 2744311 | 21 |
| exprReg_18518 | gi|126640115|ref|NC_009085.1| | 2744339 | 2744367 | 29 |
| exprReg_18519 | gi|126640115|ref|NC_009085.1| | 2744376 | 2744428 | 53 |
| exprReg_18520 | gi|126640115|ref|NC_009085.1| | 2744488 | 2744515 | 28 |
| exprReg_18524 | gi|126640115|ref|NC_009085.1| | 2745308 | 2745342 | 35 |
| exprReg_18532 | gi|126640115|ref|NC_009085.1| | 2746566 | 2746651 | 86 |
| exprReg_18533 | gi|126640115|ref|NC_009085.1| | 2746705 | 2746818 | 114 |
| exprReg_18559 | gi|126640115|ref|NC_009085.1| | 2750067 | 2750117 | 51 |
| exprReg_18564 | gi|126640115|ref|NC_009085.1| | 2750893 | 2750920 | 28 |
| exprReg_18574 | gi|126640115|ref|NC_009085.1| | 2752461 | 2752511 | 51 |
| exprReg_18575 | gi|126640115|ref|NC_009085.1| | 2752545 | 2752585 | 41 |
| exprReg_18587 | gi|126640115|ref|NC_009085.1| | 2754109 | 2754188 | 80 |
| exprReg_18596 | gi|126640115|ref|NC_009085.1| | 2755425 | 2755518 | 94 |
| exprReg_18604 | gi|126640115|ref|NC_009085.1| | 2756545 | 2756562 | 18 |
| exprReg_18637 | gi|126640115|ref|NC_009085.1| | 2761406 | 2761556 | 151 |
| exprReg_18672 | gi|126640115|ref|NC_009085.1| | 2765975 | 2766082 | 108 |
| exprReg_18678 | gi|126640115|ref|NC_009085.1| | 2766838 | 2766886 | 49 |
| exprReg_18688 | gi|126640115|ref|NC_009085.1| | 2768787 | 2768810 | 24 |
| exprReg_18689 | gi|126640115|ref|NC_009085.1| | 2768841 | 2768857 | 17 |
| exprReg_18690 | gi|126640115|ref|NC_009085.1| | 2768986 | 2769016 | 31 |
| exprReg_18691 | gi|126640115|ref|NC_009085.1| | 2769187 | 2769242 | 56 |
| exprReg_18692 | gi|126640115|ref|NC_009085.1| | 2769273 | 2769366 | 94 |
| exprReg_18708 | gi|126640115|ref|NC_009085.1| | 2771480 | 2771532 | 53 |
| exprReg_18718 | gi|126640115|ref|NC_009085.1| | 2773218 | 2773234 | 17 |
| exprReg_18719 | gi|126640115|ref|NC_009085.1| | 2773796 | 2773815 | 20 |
| exprReg_18735 | gi|126640115|ref|NC_009085.1| | 2775148 | 2775165 | 18 |
| exprReg_18736 | gi|126640115|ref|NC_009085.1| | 2775201 | 2775250 | 50 |
| exprReg_18737 | gi|126640115|ref|NC_009085.1| | 2775261 | 2775282 | 22 |
| exprReg_18738 | gi|126640115|ref|NC_009085.1| | 2775380 | 2775405 | 26 |
| exprReg_18739 | gi|126640115|ref|NC_009085.1| | 2775462 | 2775478 | 17 |
| exprReg_18749 | gi|126640115|ref|NC_009085.1| | 2777412 | 2777547 | 136 |
| exprReg_18750 | gi|126640115|ref|NC_009085.1| | 2777677 | 2777730 | 54 |
| exprReg_18754 | gi|126640115|ref|NC_009085.1| | 2778621 | 2778650 | 30 |
| exprReg_18755 | gi|126640115|ref|NC_009085.1| | 2778687 | 2778891 | 205 |
| exprReg_18756 | gi|126640115|ref|NC_009085.1| | 2779010 | 2779137 | 128 |
| exprReg_18757 | gi|126640115|ref|NC_009085.1| | 2779139 | 2779358 | 220 |
| exprReg_18758 | gi|126640115|ref|NC_009085.1| | 2779436 | 2779459 | 24 |
| exprReg_18759 | gi|126640115|ref|NC_009085.1| | 2779492 | 2779556 | 65 |
| exprReg_18760 | gi|126640115|ref|NC_009085.1| | 2779703 | 2779725 | 23 |
| exprReg_18761 | gi|126640115|ref|NC_009085.1| | 2779940 | 2779976 | 37 |
| exprReg_18762 | gi|126640115|ref|NC_009085.1| | 2779996 | 2780023 | 28 |
| exprReg_18763 | gi|126640115|ref|NC_009085.1| | 2780112 | 2780244 | 133 |
| exprReg_18764 | gi|126640115|ref|NC_009085.1| | 2780275 | 2780369 | 95 |
| exprReg_18765 | gi|126640115|ref|NC_009085.1| | 2780486 | 2780505 | 20 |
| exprReg_18766 | gi|126640115|ref|NC_009085.1| | 2780641 | 2780722 | 82 |
| exprReg_18767 | gi|126640115|ref|NC_009085.1| | 2780757 | 2780882 | 126 |
| exprReg_18768 | gi|126640115|ref|NC_009085.1| | 2781009 | 2781084 | 76 |
| exprReg_18773 | gi|126640115|ref|NC_009085.1| | 2782507 | 2782549 | 43 |
| exprReg_18774 | gi|126640115|ref|NC_009085.1| | 2782670 | 2782732 | 63 |
| exprReg_18775 | gi|126640115|ref|NC_009085.1| | 2782884 | 2782901 | 18 |
| exprReg_18780 | gi|126640115|ref|NC_009085.1| | 2783758 | 2783796 | 39 |
| exprReg_18781 | gi|126640115|ref|NC_009085.1| | 2783839 | 2783929 | 91 |
| exprReg_18782 | gi|126640115|ref|NC_009085.1| | 2783988 | 2784059 | 72 |
| exprReg_18786 | gi|126640115|ref|NC_009085.1| | 2785085 | 2785104 | 20 |
| exprReg_18787 | gi|126640115|ref|NC_009085.1| | 2785116 | 2785137 | 22 |
| exprReg_18799 | gi|126640115|ref|NC_009085.1| | 2787091 | 2787154 | 64 |
| exprReg_18814 | gi|126640115|ref|NC_009085.1| | 2789582 | 2789599 | 18 |
| exprReg_18819 | gi|126640115|ref|NC_009085.1| | 2790673 | 2790713 | 41 |
| exprReg_18820 | gi|126640115|ref|NC_009085.1| | 2790908 | 2790929 | 22 |
| exprReg_18836 | gi|126640115|ref|NC_009085.1| | 2793488 | 2793532 | 45 |
| exprReg_18841 | gi|126640115|ref|NC_009085.1| | 2794370 | 2794529 | 160 |
| exprReg_18854 | gi|126640115|ref|NC_009085.1| | 2796607 | 2796661 | 55 |
| exprReg_18876 | gi|126640115|ref|NC_009085.1| | 2800326 | 2800342 | 17 |
| exprReg_18877 | gi|126640115|ref|NC_009085.1| | 2800346 | 2800386 | 41 |
| exprReg_18886 | gi|126640115|ref|NC_009085.1| | 2801519 | 2801539 | 21 |
| exprReg_18894 | gi|126640115|ref|NC_009085.1| | 2803071 | 2803140 | 70 |
| exprReg_18900 | gi|126640115|ref|NC_009085.1| | 2803769 | 2803984 | 216 |
| exprReg_18925 | gi|126640115|ref|NC_009085.1| | 2806628 | 2806647 | 20 |
| exprReg_18945 | gi|126640115|ref|NC_009085.1| | 2810210 | 2810271 | 62 |
| exprReg_18972 | gi|126640115|ref|NC_009085.1| | 2812854 | 2812874 | 21 |
| exprReg_18977 | gi|126640115|ref|NC_009085.1| | 2813713 | 2813729 | 17 |
| exprReg_18992 | gi|126640115|ref|NC_009085.1| | 2815719 | 2815738 | 20 |
| exprReg_19002 | gi|126640115|ref|NC_009085.1| | 2817586 | 2817628 | 43 |
| exprReg_19003 | gi|126640115|ref|NC_009085.1| | 2817660 | 2817680 | 21 |
| exprReg_19008 | gi|126640115|ref|NC_009085.1| | 2818286 | 2818392 | 107 |
| exprReg_19009 | gi|126640115|ref|NC_009085.1| | 2818434 | 2818487 | 54 |
| exprReg_19010 | gi|126640115|ref|NC_009085.1| | 2818672 | 2818694 | 23 |
| exprReg_19011 | gi|126640115|ref|NC_009085.1| | 2818839 | 2818859 | 21 |
| exprReg_19017 | gi|126640115|ref|NC_009085.1| | 2819679 | 2819749 | 71 |
| exprReg_19028 | gi|126640115|ref|NC_009085.1| | 2820837 | 2820901 | 65 |
| exprReg_19043 | gi|126640115|ref|NC_009085.1| | 2824006 | 2824030 | 25 |
| exprReg_19057 | gi|126640115|ref|NC_009085.1| | 2825449 | 2825520 | 72 |
| exprReg_19082 | gi|126640115|ref|NC_009085.1| | 2828264 | 2828306 | 43 |
| exprReg_19083 | gi|126640115|ref|NC_009085.1| | 2828339 | 2828372 | 34 |
| exprReg_19084 | gi|126640115|ref|NC_009085.1| | 2828410 | 2828429 | 20 |
| exprReg_19085 | gi|126640115|ref|NC_009085.1| | 2828592 | 2828611 | 20 |
| exprReg_19091 | gi|126640115|ref|NC_009085.1| | 2829357 | 2829405 | 49 |
| exprReg_19092 | gi|126640115|ref|NC_009085.1| | 2829423 | 2829630 | 208 |
| exprReg_19109 | gi|126640115|ref|NC_009085.1| | 2831880 | 2831919 | 40 |
| exprReg_19110 | gi|126640115|ref|NC_009085.1| | 2831945 | 2831987 | 43 |
| exprReg_19119 | gi|126640115|ref|NC_009085.1| | 2833315 | 2833345 | 31 |
| exprReg_19120 | gi|126640115|ref|NC_009085.1| | 2833360 | 2833387 | 28 |
| exprReg_19121 | gi|126640115|ref|NC_009085.1| | 2833393 | 2833418 | 26 |
| exprReg_19122 | gi|126640115|ref|NC_009085.1| | 2833435 | 2833483 | 49 |
| exprReg_19123 | gi|126640115|ref|NC_009085.1| | 2833485 | 2833533 | 49 |
| exprReg_19132 | gi|126640115|ref|NC_009085.1| | 2834612 | 2834639 | 28 |
| exprReg_19133 | gi|126640115|ref|NC_009085.1| | 2834654 | 2834684 | 31 |
| exprReg_19134 | gi|126640115|ref|NC_009085.1| | 2834954 | 2835010 | 57 |
| exprReg_19142 | gi|126640115|ref|NC_009085.1| | 2836170 | 2836201 | 32 |
| exprReg_19143 | gi|126640115|ref|NC_009085.1| | 2836207 | 2836324 | 118 |
| exprReg_19144 | gi|126640115|ref|NC_009085.1| | 2836360 | 2836501 | 142 |
| exprReg_19155 | gi|126640115|ref|NC_009085.1| | 2838080 | 2838122 | 43 |
| exprReg_19156 | gi|126640115|ref|NC_009085.1| | 2838140 | 2838167 | 28 |
| exprReg_19157 | gi|126640115|ref|NC_009085.1| | 2838185 | 2838226 | 42 |
| exprReg_19158 | gi|126640115|ref|NC_009085.1| | 2838229 | 2838252 | 24 |
| exprReg_19159 | gi|126640115|ref|NC_009085.1| | 2838277 | 2838299 | 23 |
| exprReg_19160 | gi|126640115|ref|NC_009085.1| | 2838349 | 2838408 | 60 |
| exprReg_19172 | gi|126640115|ref|NC_009085.1| | 2840136 | 2840162 | 27 |
| exprReg_19173 | gi|126640115|ref|NC_009085.1| | 2840190 | 2840206 | 17 |
| exprReg_19174 | gi|126640115|ref|NC_009085.1| | 2840237 | 2840298 | 62 |
| exprReg_19175 | gi|126640115|ref|NC_009085.1| | 2840377 | 2840527 | 151 |
| exprReg_19176 | gi|126640115|ref|NC_009085.1| | 2840606 | 2840651 | 46 |
| exprReg_19201 | gi|126640115|ref|NC_009085.1| | 2844022 | 2844088 | 67 |
| exprReg_19213 | gi|126640115|ref|NC_009085.1| | 2845539 | 2845675 | 137 |
| exprReg_19214 | gi|126640115|ref|NC_009085.1| | 2845746 | 2845788 | 43 |
| exprReg_19215 | gi|126640115|ref|NC_009085.1| | 2845819 | 2845933 | 115 |
| exprReg_19220 | gi|126640115|ref|NC_009085.1| | 2846454 | 2846504 | 51 |
| exprReg_19228 | gi|126640115|ref|NC_009085.1| | 2847534 | 2847552 | 19 |
| exprReg_19274 | gi|126640115|ref|NC_009085.1| | 2853352 | 2853400 | 49 |
| exprReg_19275 | gi|126640115|ref|NC_009085.1| | 2853432 | 2853482 | 51 |
| exprReg_19276 | gi|126640115|ref|NC_009085.1| | 2853518 | 2853581 | 64 |
| exprReg_19277 | gi|126640115|ref|NC_009085.1| | 2853586 | 2853623 | 38 |
| exprReg_19282 | gi|126640115|ref|NC_009085.1| | 2854554 | 2854792 | 239 |
| exprReg_19283 | gi|126640115|ref|NC_009085.1| | 2854866 | 2854897 | 32 |
| exprReg_19284 | gi|126640115|ref|NC_009085.1| | 2854918 | 2854970 | 53 |
| exprReg_19285 | gi|126640115|ref|NC_009085.1| | 2854981 | 2855049 | 69 |
| exprReg_19286 | gi|126640115|ref|NC_009085.1| | 2855090 | 2855109 | 20 |
| exprReg_19287 | gi|126640115|ref|NC_009085.1| | 2855138 | 2855220 | 83 |
| exprReg_19297 | gi|126640115|ref|NC_009085.1| | 2856473 | 2856566 | 94 |
| exprReg_19307 | gi|126640115|ref|NC_009085.1| | 2857698 | 2857804 | 107 |
| exprReg_19323 | gi|126640115|ref|NC_009085.1| | 2859905 | 2859922 | 18 |
| exprReg_19324 | gi|126640115|ref|NC_009085.1| | 2859939 | 2860059 | 121 |
| exprReg_19328 | gi|126640115|ref|NC_009085.1| | 2860559 | 2860576 | 18 |
| exprReg_19346 | gi|126640115|ref|NC_009085.1| | 2863248 | 2863265 | 18 |
| exprReg_19347 | gi|126640115|ref|NC_009085.1| | 2863332 | 2863384 | 53 |
| exprReg_19353 | gi|126640115|ref|NC_009085.1| | 2864186 | 2864236 | 51 |
| exprReg_19363 | gi|126640115|ref|NC_009085.1| | 2865658 | 2865690 | 33 |
| exprReg_19364 | gi|126640115|ref|NC_009085.1| | 2865817 | 2865880 | 64 |
| exprReg_19381 | gi|126640115|ref|NC_009085.1| | 2868511 | 2868667 | 157 |
| exprReg_19382 | gi|126640115|ref|NC_009085.1| | 2868743 | 2868762 | 20 |
| exprReg_19383 | gi|126640115|ref|NC_009085.1| | 2868770 | 2868829 | 60 |
| exprReg_19384 | gi|126640115|ref|NC_009085.1| | 2868832 | 2868886 | 55 |
| exprReg_19385 | gi|126640115|ref|NC_009085.1| | 2868915 | 2868935 | 21 |
| exprReg_19386 | gi|126640115|ref|NC_009085.1| | 2869045 | 2869196 | 152 |
| exprReg_19387 | gi|126640115|ref|NC_009085.1| | 2869304 | 2869320 | 17 |
| exprReg_19388 | gi|126640115|ref|NC_009085.1| | 2869340 | 2869398 | 59 |
| exprReg_19395 | gi|126640115|ref|NC_009085.1| | 2870697 | 2870854 | 158 |
| exprReg_19401 | gi|126640115|ref|NC_009085.1| | 2871797 | 2871814 | 18 |
| exprReg_19420 | gi|126640115|ref|NC_009085.1| | 2874171 | 2874408 | 238 |
| exprReg_19433 | gi|126640115|ref|NC_009085.1| | 2876322 | 2876427 | 106 |
| exprReg_19434 | gi|126640115|ref|NC_009085.1| | 2876443 | 2876468 | 26 |
| exprReg_19435 | gi|126640115|ref|NC_009085.1| | 2876471 | 2876537 | 67 |
| exprReg_19446 | gi|126640115|ref|NC_009085.1| | 2877729 | 2877782 | 54 |
| exprReg_19447 | gi|126640115|ref|NC_009085.1| | 2877818 | 2877834 | 17 |
| exprReg_19448 | gi|126640115|ref|NC_009085.1| | 2877860 | 2877939 | 80 |
| exprReg_19449 | gi|126640115|ref|NC_009085.1| | 2877941 | 2877959 | 19 |
| exprReg_19459 | gi|126640115|ref|NC_009085.1| | 2879807 | 2879835 | 29 |
| exprReg_19460 | gi|126640115|ref|NC_009085.1| | 2879854 | 2879910 | 57 |
| exprReg_19461 | gi|126640115|ref|NC_009085.1| | 2879944 | 2879961 | 18 |
| exprReg_19462 | gi|126640115|ref|NC_009085.1| | 2880007 | 2880079 | 73 |
| exprReg_19477 | gi|126640115|ref|NC_009085.1| | 2881326 | 2881352 | 27 |
| exprReg_19478 | gi|126640115|ref|NC_009085.1| | 2881363 | 2881414 | 52 |
| exprReg_19479 | gi|126640115|ref|NC_009085.1| | 2881438 | 2881464 | 27 |
| exprReg_19486 | gi|126640115|ref|NC_009085.1| | 2882231 | 2882275 | 45 |
| exprReg_19487 | gi|126640115|ref|NC_009085.1| | 2882295 | 2882357 | 63 |
| exprReg_19495 | gi|126640115|ref|NC_009085.1| | 2883180 | 2883202 | 23 |
| exprReg_19507 | gi|126640115|ref|NC_009085.1| | 2885254 | 2885279 | 26 |
| exprReg_19508 | gi|126640115|ref|NC_009085.1| | 2885296 | 2885375 | 80 |
| exprReg_19509 | gi|126640115|ref|NC_009085.1| | 2885390 | 2885500 | 111 |
| exprReg_19510 | gi|126640115|ref|NC_009085.1| | 2885513 | 2885536 | 24 |
| exprReg_19511 | gi|126640115|ref|NC_009085.1| | 2885554 | 2885606 | 53 |
| exprReg_19533 | gi|126640115|ref|NC_009085.1| | 2887887 | 2887907 | 21 |
| exprReg_19548 | gi|126640115|ref|NC_009085.1| | 2889484 | 2889526 | 43 |
| exprReg_19549 | gi|126640115|ref|NC_009085.1| | 2889536 | 2889613 | 78 |
| exprReg_19550 | gi|126640115|ref|NC_009085.1| | 2889657 | 2889730 | 74 |
| exprReg_19565 | gi|126640115|ref|NC_009085.1| | 2891562 | 2891582 | 21 |
| exprReg_19588 | gi|126640115|ref|NC_009085.1| | 2894537 | 2894558 | 22 |
| exprReg_19604 | gi|126640115|ref|NC_009085.1| | 2896923 | 2896942 | 20 |
| exprReg_19617 | gi|126640115|ref|NC_009085.1| | 2898548 | 2898601 | 54 |
| exprReg_19633 | gi|126640115|ref|NC_009085.1| | 2901217 | 2901308 | 92 |
| exprReg_19634 | gi|126640115|ref|NC_009085.1| | 2901421 | 2901443 | 23 |
| exprReg_19637 | gi|126640115|ref|NC_009085.1| | 2901894 | 2902069 | 176 |
| exprReg_19645 | gi|126640115|ref|NC_009085.1| | 2904063 | 2904089 | 27 |
| exprReg_19646 | gi|126640115|ref|NC_009085.1| | 2904092 | 2904124 | 33 |
| exprReg_19647 | gi|126640115|ref|NC_009085.1| | 2904150 | 2904188 | 39 |
| exprReg_19648 | gi|126640115|ref|NC_009085.1| | 2904277 | 2904294 | 18 |
| exprReg_19656 | gi|126640115|ref|NC_009085.1| | 2905221 | 2905238 | 18 |
| exprReg_19657 | gi|126640115|ref|NC_009085.1| | 2905288 | 2905304 | 17 |
| exprReg_19668 | gi|126640115|ref|NC_009085.1| | 2907057 | 2907106 | 50 |
| exprReg_19669 | gi|126640115|ref|NC_009085.1| | 2907153 | 2907246 | 94 |
| exprReg_19680 | gi|126640115|ref|NC_009085.1| | 2908432 | 2908471 | 40 |
| exprReg_19681 | gi|126640115|ref|NC_009085.1| | 2908569 | 2908640 | 72 |
| exprReg_19682 | gi|126640115|ref|NC_009085.1| | 2908646 | 2908669 | 24 |
| exprReg_19683 | gi|126640115|ref|NC_009085.1| | 2908684 | 2908729 | 46 |
| exprReg_19684 | gi|126640115|ref|NC_009085.1| | 2908752 | 2908772 | 21 |
| exprReg_19717 | gi|126640115|ref|NC_009085.1| | 2912460 | 2912481 | 22 |
| exprReg_19718 | gi|126640115|ref|NC_009085.1| | 2912953 | 2913011 | 59 |
| exprReg_19719 | gi|126640115|ref|NC_009085.1| | 2913021 | 2913200 | 180 |
| exprReg_19727 | gi|126640115|ref|NC_009085.1| | 2914544 | 2914564 | 21 |
| exprReg_19735 | gi|126640115|ref|NC_009085.1| | 2915372 | 2915396 | 25 |
| exprReg_19736 | gi|126640115|ref|NC_009085.1| | 2915414 | 2915467 | 54 |
| exprReg_19744 | gi|126640115|ref|NC_009085.1| | 2917003 | 2917050 | 48 |
| exprReg_19751 | gi|126640115|ref|NC_009085.1| | 2917857 | 2917895 | 39 |
| exprReg_19775 | gi|126640115|ref|NC_009085.1| | 2920772 | 2920796 | 25 |
| exprReg_19776 | gi|126640115|ref|NC_009085.1| | 2920798 | 2920832 | 35 |
| exprReg_19791 | gi|126640115|ref|NC_009085.1| | 2922740 | 2922764 | 25 |
| exprReg_19792 | gi|126640115|ref|NC_009085.1| | 2922766 | 2922807 | 42 |
| exprReg_19793 | gi|126640115|ref|NC_009085.1| | 2922832 | 2922914 | 83 |
| exprReg_19794 | gi|126640115|ref|NC_009085.1| | 2923056 | 2923101 | 46 |
| exprReg_19795 | gi|126640115|ref|NC_009085.1| | 2923130 | 2923148 | 19 |
| exprReg_19802 | gi|126640115|ref|NC_009085.1| | 2924088 | 2924112 | 25 |
| exprReg_19812 | gi|126640115|ref|NC_009085.1| | 2925599 | 2925644 | 46 |
| exprReg_19813 | gi|126640115|ref|NC_009085.1| | 2925749 | 2925817 | 69 |
| exprReg_19814 | gi|126640115|ref|NC_009085.1| | 2925821 | 2925841 | 21 |
| exprReg_19815 | gi|126640115|ref|NC_009085.1| | 2925843 | 2925881 | 39 |
| exprReg_19816 | gi|126640115|ref|NC_009085.1| | 2925905 | 2925965 | 61 |
| exprReg_19832 | gi|126640115|ref|NC_009085.1| | 2928489 | 2928532 | 44 |
| exprReg_19844 | gi|126640115|ref|NC_009085.1| | 2930306 | 2930322 | 17 |
| exprReg_19845 | gi|126640115|ref|NC_009085.1| | 2930382 | 2930466 | 85 |
| exprReg_19846 | gi|126640115|ref|NC_009085.1| | 2930477 | 2930519 | 43 |
| exprReg_19847 | gi|126640115|ref|NC_009085.1| | 2930558 | 2930588 | 31 |
| exprReg_19854 | gi|126640115|ref|NC_009085.1| | 2931536 | 2931640 | 105 |
| exprReg_19877 | gi|126640115|ref|NC_009085.1| | 2935355 | 2935400 | 46 |
| exprReg_19890 | gi|126640115|ref|NC_009085.1| | 2937272 | 2937320 | 49 |
| exprReg_19898 | gi|126640115|ref|NC_009085.1| | 2938250 | 2938272 | 23 |
| exprReg_19914 | gi|126640115|ref|NC_009085.1| | 2940286 | 2940317 | 32 |
| exprReg_19921 | gi|126640115|ref|NC_009085.1| | 2941618 | 2941637 | 20 |
| exprReg_19924 | gi|126640115|ref|NC_009085.1| | 2942120 | 2942137 | 18 |
| exprReg_19931 | gi|126640115|ref|NC_009085.1| | 2942858 | 2942916 | 59 |
| exprReg_19932 | gi|126640115|ref|NC_009085.1| | 2942944 | 2942962 | 19 |
| exprReg_19942 | gi|126640115|ref|NC_009085.1| | 2944100 | 2944255 | 156 |
| exprReg_19943 | gi|126640115|ref|NC_009085.1| | 2944479 | 2944497 | 19 |
| exprReg_19944 | gi|126640115|ref|NC_009085.1| | 2944502 | 2944567 | 66 |
| exprReg_19954 | gi|126640115|ref|NC_009085.1| | 2945490 | 2945544 | 55 |
| exprReg_19974 | gi|126640115|ref|NC_009085.1| | 2947613 | 2947660 | 48 |
| exprReg_19990 | gi|126640115|ref|NC_009085.1| | 2949262 | 2949283 | 22 |
| exprReg_19995 | gi|126640115|ref|NC_009085.1| | 2950119 | 2950381 | 263 |
| exprReg_19999 | gi|126640115|ref|NC_009085.1| | 2951094 | 2951111 | 18 |
| exprReg_20000 | gi|126640115|ref|NC_009085.1| | 2951123 | 2951139 | 17 |
| exprReg_20005 | gi|126640115|ref|NC_009085.1| | 2951869 | 2951888 | 20 |
| exprReg_20015 | gi|126640115|ref|NC_009085.1| | 2953424 | 2953467 | 44 |
| exprReg_20016 | gi|126640115|ref|NC_009085.1| | 2953472 | 2953501 | 30 |
| exprReg_20017 | gi|126640115|ref|NC_009085.1| | 2953547 | 2953567 | 21 |
| exprReg_20020 | gi|126640115|ref|NC_009085.1| | 2954404 | 2954459 | 56 |
| exprReg_20021 | gi|126640115|ref|NC_009085.1| | 2954492 | 2954597 | 106 |
| exprReg_20022 | gi|126640115|ref|NC_009085.1| | 2954663 | 2954680 | 18 |
| exprReg_20023 | gi|126640115|ref|NC_009085.1| | 2954824 | 2954888 | 65 |
| exprReg_20024 | gi|126640115|ref|NC_009085.1| | 2954933 | 2954950 | 18 |
| exprReg_20025 | gi|126640115|ref|NC_009085.1| | 2954974 | 2954995 | 22 |
| exprReg_20026 | gi|126640115|ref|NC_009085.1| | 2955110 | 2955294 | 185 |
| exprReg_20027 | gi|126640115|ref|NC_009085.1| | 2955312 | 2955380 | 69 |
| exprReg_20028 | gi|126640115|ref|NC_009085.1| | 2955387 | 2955672 | 286 |
| exprReg_20047 | gi|126640115|ref|NC_009085.1| | 2958114 | 2958136 | 23 |
| exprReg_20048 | gi|126640115|ref|NC_009085.1| | 2958145 | 2958171 | 27 |
| exprReg_20068 | gi|126640115|ref|NC_009085.1| | 2960387 | 2960405 | 19 |
| exprReg_20105 | gi|126640115|ref|NC_009085.1| | 2966031 | 2966139 | 109 |
| exprReg_20106 | gi|126640115|ref|NC_009085.1| | 2966158 | 2966251 | 94 |
| exprReg_20107 | gi|126640115|ref|NC_009085.1| | 2966393 | 2966463 | 71 |
| exprReg_20123 | gi|126640115|ref|NC_009085.1| | 2968414 | 2968448 | 35 |
| exprReg_20124 | gi|126640115|ref|NC_009085.1| | 2968487 | 2968519 | 33 |
| exprReg_20125 | gi|126640115|ref|NC_009085.1| | 2968556 | 2968575 | 20 |
| exprReg_20127 | gi|126640115|ref|NC_009085.1| | 2969024 | 2969043 | 20 |
| exprReg_20128 | gi|126640115|ref|NC_009085.1| | 2969106 | 2969208 | 103 |
| exprReg_20133 | gi|126640115|ref|NC_009085.1| | 2969966 | 2969988 | 23 |
| exprReg_20154 | gi|126640115|ref|NC_009085.1| | 2972444 | 2972463 | 20 |
| exprReg_20155 | gi|126640115|ref|NC_009085.1| | 2972500 | 2972523 | 24 |
| exprReg_20156 | gi|126640115|ref|NC_009085.1| | 2972555 | 2972578 | 24 |
| exprReg_20169 | gi|126640115|ref|NC_009085.1| | 2974047 | 2974068 | 22 |
| exprReg_20243 | gi|126640115|ref|NC_009085.1| | 2985691 | 2985807 | 117 |
| exprReg_20244 | gi|126640115|ref|NC_009085.1| | 2985855 | 2985871 | 17 |
| exprReg_20245 | gi|126640115|ref|NC_009085.1| | 2985898 | 2985914 | 17 |
| exprReg_20246 | gi|126640115|ref|NC_009085.1| | 2986005 | 2986071 | 67 |
| exprReg_20247 | gi|126640115|ref|NC_009085.1| | 2986208 | 2986265 | 58 |
| exprReg_20248 | gi|126640115|ref|NC_009085.1| | 2986273 | 2986294 | 22 |
| exprReg_20257 | gi|126640115|ref|NC_009085.1| | 2987267 | 2987283 | 17 |
| exprReg_20258 | gi|126640115|ref|NC_009085.1| | 2987347 | 2987364 | 18 |
| exprReg_20259 | gi|126640115|ref|NC_009085.1| | 2987366 | 2987389 | 24 |
| exprReg_20260 | gi|126640115|ref|NC_009085.1| | 2987473 | 2987489 | 17 |
| exprReg_20261 | gi|126640115|ref|NC_009085.1| | 2987494 | 2987516 | 23 |
| exprReg_20262 | gi|126640115|ref|NC_009085.1| | 2987531 | 2987547 | 17 |
| exprReg_20263 | gi|126640115|ref|NC_009085.1| | 2987553 | 2987570 | 18 |
| exprReg_20264 | gi|126640115|ref|NC_009085.1| | 2987685 | 2987701 | 17 |
| exprReg_20270 | gi|126640115|ref|NC_009085.1| | 2988435 | 2988479 | 45 |
| exprReg_20271 | gi|126640115|ref|NC_009085.1| | 2988570 | 2988822 | 253 |
| exprReg_20272 | gi|126640115|ref|NC_009085.1| | 2988891 | 2988907 | 17 |
| exprReg_20273 | gi|126640115|ref|NC_009085.1| | 2988972 | 2989312 | 341 |
| exprReg_20274 | gi|126640115|ref|NC_009085.1| | 2989416 | 2989565 | 150 |
| exprReg_20283 | gi|126640115|ref|NC_009085.1| | 2990628 | 2990705 | 78 |
| exprReg_20284 | gi|126640115|ref|NC_009085.1| | 2990718 | 2990744 | 27 |
| exprReg_20285 | gi|126640115|ref|NC_009085.1| | 2990767 | 2990783 | 17 |
| exprReg_20308 | gi|126640115|ref|NC_009085.1| | 2994946 | 2995002 | 57 |
| exprReg_20309 | gi|126640115|ref|NC_009085.1| | 2995028 | 2995048 | 21 |
| exprReg_20310 | gi|126640115|ref|NC_009085.1| | 2995068 | 2995166 | 99 |
| exprReg_20323 | gi|126640115|ref|NC_009085.1| | 2996309 | 2996352 | 44 |
| exprReg_20361 | gi|126640115|ref|NC_009085.1| | 3001458 | 3001496 | 39 |
| exprReg_20383 | gi|126640115|ref|NC_009085.1| | 3004639 | 3004823 | 185 |
| exprReg_20384 | gi|126640115|ref|NC_009085.1| | 3004845 | 3004913 | 69 |
| exprReg_20385 | gi|126640115|ref|NC_009085.1| | 3005013 | 3005055 | 43 |
| exprReg_20386 | gi|126640115|ref|NC_009085.1| | 3005101 | 3005118 | 18 |
| exprReg_20387 | gi|126640115|ref|NC_009085.1| | 3005130 | 3005155 | 26 |
| exprReg_20397 | gi|126640115|ref|NC_009085.1| | 3006043 | 3006091 | 49 |
| exprReg_20398 | gi|126640115|ref|NC_009085.1| | 3006187 | 3006232 | 46 |
| exprReg_20403 | gi|126640115|ref|NC_009085.1| | 3006912 | 3007013 | 102 |
| exprReg_20438 | gi|126640115|ref|NC_009085.1| | 3010823 | 3010843 | 21 |
| exprReg_20460 | gi|126640115|ref|NC_009085.1| | 3012626 | 3012644 | 19 |
| exprReg_20461 | gi|126640115|ref|NC_009085.1| | 3012659 | 3012751 | 93 |
| exprReg_20462 | gi|126640115|ref|NC_009085.1| | 3012766 | 3012811 | 46 |
| exprReg_20534 | gi|126640115|ref|NC_009085.1| | 3024160 | 3024416 | 257 |
| exprReg_20542 | gi|126640115|ref|NC_009085.1| | 3025275 | 3025291 | 17 |
| exprReg_20546 | gi|126640115|ref|NC_009085.1| | 3026017 | 3026070 | 54 |
| exprReg_20547 | gi|126640115|ref|NC_009085.1| | 3026085 | 3026103 | 19 |
| exprReg_20567 | gi|126640115|ref|NC_009085.1| | 3028609 | 3028670 | 62 |
| exprReg_20574 | gi|126640115|ref|NC_009085.1| | 3029427 | 3029450 | 24 |
| exprReg_20602 | gi|126640115|ref|NC_009085.1| | 3032631 | 3032720 | 90 |
| exprReg_20611 | gi|126640115|ref|NC_009085.1| | 3033606 | 3033669 | 64 |
| exprReg_20618 | gi|126640115|ref|NC_009085.1| | 3034874 | 3034941 | 68 |
| exprReg_20620 | gi|126640115|ref|NC_009085.1| | 3035701 | 3035797 | 97 |
| exprReg_20621 | gi|126640115|ref|NC_009085.1| | 3036107 | 3036126 | 20 |
| exprReg_20644 | gi|126640115|ref|NC_009085.1| | 3040557 | 3040587 | 31 |
| exprReg_20645 | gi|126640115|ref|NC_009085.1| | 3040623 | 3040655 | 33 |
| exprReg_20654 | gi|126640115|ref|NC_009085.1| | 3042884 | 3042905 | 22 |
| exprReg_20664 | gi|126640115|ref|NC_009085.1| | 3043691 | 3043710 | 20 |
| exprReg_20665 | gi|126640115|ref|NC_009085.1| | 3043738 | 3043759 | 22 |
| exprReg_20666 | gi|126640115|ref|NC_009085.1| | 3043766 | 3043803 | 38 |
| exprReg_20667 | gi|126640115|ref|NC_009085.1| | 3043813 | 3043925 | 113 |
| exprReg_20668 | gi|126640115|ref|NC_009085.1| | 3043967 | 3043994 | 28 |
| exprReg_20669 | gi|126640115|ref|NC_009085.1| | 3044035 | 3044116 | 82 |
| exprReg_20685 | gi|126640115|ref|NC_009085.1| | 3046681 | 3046755 | 75 |
| exprReg_20686 | gi|126640115|ref|NC_009085.1| | 3046757 | 3046783 | 27 |
| exprReg_20687 | gi|126640115|ref|NC_009085.1| | 3046795 | 3046816 | 22 |
| exprReg_20688 | gi|126640115|ref|NC_009085.1| | 3046835 | 3046872 | 38 |
| exprReg_20689 | gi|126640115|ref|NC_009085.1| | 3046881 | 3046900 | 20 |
| exprReg_20690 | gi|126640115|ref|NC_009085.1| | 3046908 | 3047103 | 196 |
| exprReg_20700 | gi|126640115|ref|NC_009085.1| | 3048643 | 3048708 | 66 |
| exprReg_20701 | gi|126640115|ref|NC_009085.1| | 3048843 | 3049001 | 159 |
| exprReg_20736 | gi|126640115|ref|NC_009085.1| | 3053534 | 3053577 | 44 |
| exprReg_20741 | gi|126640115|ref|NC_009085.1| | 3054194 | 3054221 | 28 |
| exprReg_20744 | gi|126640115|ref|NC_009085.1| | 3054933 | 3055072 | 140 |
| exprReg_20762 | gi|126640115|ref|NC_009085.1| | 3057544 | 3057570 | 27 |
| exprReg_20763 | gi|126640115|ref|NC_009085.1| | 3057592 | 3057611 | 20 |
| exprReg_20764 | gi|126640115|ref|NC_009085.1| | 3057657 | 3057713 | 57 |
| exprReg_20774 | gi|126640115|ref|NC_009085.1| | 3059611 | 3059655 | 45 |
| exprReg_20798 | gi|126640115|ref|NC_009085.1| | 3063823 | 3064045 | 223 |
| exprReg_20799 | gi|126640115|ref|NC_009085.1| | 3064492 | 3064658 | 167 |
| exprReg_20800 | gi|126640115|ref|NC_009085.1| | 3064741 | 3064759 | 19 |
| exprReg_20801 | gi|126640115|ref|NC_009085.1| | 3064903 | 3064949 | 47 |
| exprReg_20802 | gi|126640115|ref|NC_009085.1| | 3064956 | 3064974 | 19 |
| exprReg_20803 | gi|126640115|ref|NC_009085.1| | 3065158 | 3065176 | 19 |
| exprReg_20804 | gi|126640115|ref|NC_009085.1| | 3065215 | 3065240 | 26 |
| exprReg_20805 | gi|126640115|ref|NC_009085.1| | 3065326 | 3065353 | 28 |
| exprReg_20806 | gi|126640115|ref|NC_009085.1| | 3065359 | 3065404 | 46 |
| exprReg_20807 | gi|126640115|ref|NC_009085.1| | 3065450 | 3065534 | 85 |
| exprReg_20815 | gi|126640115|ref|NC_009085.1| | 3066091 | 3066230 | 140 |
| exprReg_20816 | gi|126640115|ref|NC_009085.1| | 3066260 | 3066288 | 29 |
| exprReg_20821 | gi|126640115|ref|NC_009085.1| | 3066914 | 3066933 | 20 |
| exprReg_20822 | gi|126640115|ref|NC_009085.1| | 3066962 | 3066988 | 27 |
| exprReg_20823 | gi|126640115|ref|NC_009085.1| | 3066994 | 3067014 | 21 |
| exprReg_20824 | gi|126640115|ref|NC_009085.1| | 3067029 | 3067120 | 92 |
| exprReg_20825 | gi|126640115|ref|NC_009085.1| | 3067175 | 3067241 | 67 |
| exprReg_20826 | gi|126640115|ref|NC_009085.1| | 3067243 | 3067333 | 91 |
| exprReg_20827 | gi|126640115|ref|NC_009085.1| | 3067498 | 3067515 | 18 |
| exprReg_20828 | gi|126640115|ref|NC_009085.1| | 3067544 | 3067650 | 107 |
| exprReg_20829 | gi|126640115|ref|NC_009085.1| | 3067724 | 3067754 | 31 |
| exprReg_20830 | gi|126640115|ref|NC_009085.1| | 3067769 | 3067920 | 152 |
| exprReg_20831 | gi|126640115|ref|NC_009085.1| | 3067925 | 3067942 | 18 |
| exprReg_20832 | gi|126640115|ref|NC_009085.1| | 3068015 | 3068033 | 19 |
| exprReg_20833 | gi|126640115|ref|NC_009085.1| | 3068137 | 3068222 | 86 |
| exprReg_20834 | gi|126640115|ref|NC_009085.1| | 3068632 | 3068765 | 134 |
| exprReg_20835 | gi|126640115|ref|NC_009085.1| | 3068960 | 3069064 | 105 |
| exprReg_20836 | gi|126640115|ref|NC_009085.1| | 3069085 | 3069168 | 84 |
| exprReg_20837 | gi|126640115|ref|NC_009085.1| | 3069184 | 3069389 | 206 |
| exprReg_20866 | gi|126640115|ref|NC_009085.1| | 3074263 | 3074395 | 133 |
| exprReg_20867 | gi|126640115|ref|NC_009085.1| | 3074508 | 3074524 | 17 |
| exprReg_20868 | gi|126640115|ref|NC_009085.1| | 3074529 | 3074545 | 17 |
| exprReg_20869 | gi|126640115|ref|NC_009085.1| | 3074605 | 3074647 | 43 |
| exprReg_20870 | gi|126640115|ref|NC_009085.1| | 3074691 | 3074712 | 22 |
| exprReg_20871 | gi|126640115|ref|NC_009085.1| | 3074880 | 3074941 | 62 |
| exprReg_20872 | gi|126640115|ref|NC_009085.1| | 3075004 | 3075023 | 20 |
| exprReg_20873 | gi|126640115|ref|NC_009085.1| | 3075067 | 3075142 | 76 |
| exprReg_20878 | gi|126640115|ref|NC_009085.1| | 3076054 | 3076073 | 20 |
| exprReg_20879 | gi|126640115|ref|NC_009085.1| | 3076077 | 3076126 | 50 |
| exprReg_20880 | gi|126640115|ref|NC_009085.1| | 3076137 | 3076177 | 41 |
| exprReg_20881 | gi|126640115|ref|NC_009085.1| | 3076230 | 3076259 | 30 |
| exprReg_20888 | gi|126640115|ref|NC_009085.1| | 3077405 | 3077424 | 20 |
| exprReg_20892 | gi|126640115|ref|NC_009085.1| | 3078022 | 3078040 | 19 |
| exprReg_20902 | gi|126640115|ref|NC_009085.1| | 3079016 | 3079074 | 59 |
| exprReg_20908 | gi|126640115|ref|NC_009085.1| | 3080364 | 3080402 | 39 |
| exprReg_20909 | gi|126640115|ref|NC_009085.1| | 3080428 | 3080480 | 53 |
| exprReg_20910 | gi|126640115|ref|NC_009085.1| | 3080527 | 3080615 | 89 |
| exprReg_20928 | gi|126640115|ref|NC_009085.1| | 3082069 | 3082085 | 17 |
| exprReg_20929 | gi|126640115|ref|NC_009085.1| | 3082101 | 3082181 | 81 |
| exprReg_20954 | gi|126640115|ref|NC_009085.1| | 3085282 | 3085371 | 90 |
| exprReg_20955 | gi|126640115|ref|NC_009085.1| | 3085467 | 3085490 | 24 |
| exprReg_20974 | gi|126640115|ref|NC_009085.1| | 3087618 | 3087659 | 42 |
| exprReg_20975 | gi|126640115|ref|NC_009085.1| | 3087669 | 3087714 | 46 |
| exprReg_20978 | gi|126640115|ref|NC_009085.1| | 3088217 | 3088259 | 43 |
| exprReg_20993 | gi|126640115|ref|NC_009085.1| | 3090428 | 3090444 | 17 |
| exprReg_21009 | gi|126640115|ref|NC_009085.1| | 3092187 | 3092205 | 19 |
| exprReg_21010 | gi|126640115|ref|NC_009085.1| | 3092458 | 3092492 | 35 |
| exprReg_21011 | gi|126640115|ref|NC_009085.1| | 3092508 | 3092624 | 117 |
| exprReg_21059 | gi|126640115|ref|NC_009085.1| | 3097663 | 3097682 | 20 |
| exprReg_21060 | gi|126640115|ref|NC_009085.1| | 3098006 | 3098067 | 62 |
| exprReg_21061 | gi|126640115|ref|NC_009085.1| | 3098071 | 3098132 | 62 |
| exprReg_21067 | gi|126640115|ref|NC_009085.1| | 3099348 | 3099402 | 55 |
| exprReg_21076 | gi|126640115|ref|NC_009085.1| | 3100509 | 3100531 | 23 |
| exprReg_21077 | gi|126640115|ref|NC_009085.1| | 3100538 | 3100558 | 21 |
| exprReg_21078 | gi|126640115|ref|NC_009085.1| | 3100603 | 3100797 | 195 |
| exprReg_21083 | gi|126640115|ref|NC_009085.1| | 3101551 | 3101795 | 245 |
| exprReg_21093 | gi|126640115|ref|NC_009085.1| | 3103617 | 3103698 | 82 |
| exprReg_21094 | gi|126640115|ref|NC_009085.1| | 3103794 | 3103810 | 17 |
| exprReg_21095 | gi|126640115|ref|NC_009085.1| | 3103911 | 3104020 | 110 |
| exprReg_21097 | gi|126640115|ref|NC_009085.1| | 3104505 | 3104530 | 26 |
| exprReg_21099 | gi|126640115|ref|NC_009085.1| | 3104895 | 3105000 | 106 |
| exprReg_21105 | gi|126640115|ref|NC_009085.1| | 3105807 | 3105823 | 17 |
| exprReg_21118 | gi|126640115|ref|NC_009085.1| | 3108795 | 3108811 | 17 |
| exprReg_21119 | gi|126640115|ref|NC_009085.1| | 3108841 | 3108889 | 49 |
| exprReg_21122 | gi|126640115|ref|NC_009085.1| | 3109229 | 3109288 | 60 |
| exprReg_21132 | gi|126640115|ref|NC_009085.1| | 3110240 | 3110296 | 57 |
| exprReg_21133 | gi|126640115|ref|NC_009085.1| | 3110310 | 3110333 | 24 |
| exprReg_21134 | gi|126640115|ref|NC_009085.1| | 3110361 | 3110377 | 17 |
| exprReg_21135 | gi|126640115|ref|NC_009085.1| | 3110437 | 3110538 | 102 |
| exprReg_21174 | gi|126640115|ref|NC_009085.1| | 3115673 | 3115689 | 17 |
| exprReg_21183 | gi|126640115|ref|NC_009085.1| | 3116908 | 3117006 | 99 |
| exprReg_21189 | gi|126640115|ref|NC_009085.1| | 3117523 | 3117539 | 17 |
| exprReg_21190 | gi|126640115|ref|NC_009085.1| | 3117679 | 3117703 | 25 |
| exprReg_21195 | gi|126640115|ref|NC_009085.1| | 3118171 | 3118194 | 24 |
| exprReg_21197 | gi|126640115|ref|NC_009085.1| | 3118623 | 3118722 | 100 |
| exprReg_21198 | gi|126640115|ref|NC_009085.1| | 3118803 | 3119017 | 215 |
| exprReg_21199 | gi|126640115|ref|NC_009085.1| | 3119073 | 3119096 | 24 |
| exprReg_21200 | gi|126640115|ref|NC_009085.1| | 3119137 | 3119242 | 106 |
| exprReg_21212 | gi|126640115|ref|NC_009085.1| | 3120955 | 3120997 | 43 |
| exprReg_21213 | gi|126640115|ref|NC_009085.1| | 3121014 | 3121030 | 17 |
| exprReg_21223 | gi|126640115|ref|NC_009085.1| | 3122701 | 3122752 | 52 |
| exprReg_21268 | gi|126640115|ref|NC_009085.1| | 3130265 | 3130282 | 18 |
| exprReg_21275 | gi|126640115|ref|NC_009085.1| | 3131213 | 3131229 | 17 |
| exprReg_21276 | gi|126640115|ref|NC_009085.1| | 3131300 | 3131456 | 157 |
| exprReg_21291 | gi|126640115|ref|NC_009085.1| | 3133092 | 3133147 | 56 |
| exprReg_21292 | gi|126640115|ref|NC_009085.1| | 3133234 | 3133253 | 20 |
| exprReg_21293 | gi|126640115|ref|NC_009085.1| | 3133257 | 3133354 | 98 |
| exprReg_21294 | gi|126640115|ref|NC_009085.1| | 3133363 | 3133396 | 34 |
| exprReg_21295 | gi|126640115|ref|NC_009085.1| | 3133414 | 3133431 | 18 |
| exprReg_21306 | gi|126640115|ref|NC_009085.1| | 3134492 | 3134595 | 104 |
| exprReg_21307 | gi|126640115|ref|NC_009085.1| | 3134623 | 3134640 | 18 |
| exprReg_21320 | gi|126640115|ref|NC_009085.1| | 3135909 | 3135925 | 17 |
| exprReg_21321 | gi|126640115|ref|NC_009085.1| | 3135933 | 3136009 | 77 |
| exprReg_21322 | gi|126640115|ref|NC_009085.1| | 3136014 | 3136058 | 45 |
| exprReg_21323 | gi|126640115|ref|NC_009085.1| | 3136150 | 3136166 | 17 |
| exprReg_21337 | gi|126640115|ref|NC_009085.1| | 3138760 | 3139043 | 284 |
| exprReg_21343 | gi|126640115|ref|NC_009085.1| | 3139766 | 3139791 | 26 |
| exprReg_21344 | gi|126640115|ref|NC_009085.1| | 3139814 | 3139922 | 109 |
| exprReg_21345 | gi|126640115|ref|NC_009085.1| | 3139964 | 3139989 | 26 |
| exprReg_21346 | gi|126640115|ref|NC_009085.1| | 3140058 | 3140233 | 176 |
| exprReg_21347 | gi|126640115|ref|NC_009085.1| | 3140249 | 3140288 | 40 |
| exprReg_21348 | gi|126640115|ref|NC_009085.1| | 3140326 | 3140369 | 44 |
| exprReg_21349 | gi|126640115|ref|NC_009085.1| | 3140386 | 3140404 | 19 |
| exprReg_21350 | gi|126640115|ref|NC_009085.1| | 3140544 | 3140634 | 91 |
| exprReg_21351 | gi|126640115|ref|NC_009085.1| | 3140695 | 3140745 | 51 |
| exprReg_21352 | gi|126640115|ref|NC_009085.1| | 3140783 | 3140942 | 160 |
| exprReg_21357 | gi|126640115|ref|NC_009085.1| | 3141733 | 3141839 | 107 |
| exprReg_21358 | gi|126640115|ref|NC_009085.1| | 3141846 | 3142045 | 200 |
| exprReg_21360 | gi|126640115|ref|NC_009085.1| | 3142547 | 3142563 | 17 |
| exprReg_21373 | gi|126640115|ref|NC_009085.1| | 3143735 | 3143765 | 31 |
| exprReg_21374 | gi|126640115|ref|NC_009085.1| | 3143833 | 3143949 | 117 |
| exprReg_21375 | gi|126640115|ref|NC_009085.1| | 3143994 | 3144105 | 112 |
| exprReg_21376 | gi|126640115|ref|NC_009085.1| | 3144115 | 3144131 | 17 |
| exprReg_21377 | gi|126640115|ref|NC_009085.1| | 3144200 | 3144252 | 53 |
| exprReg_21391 | gi|126640115|ref|NC_009085.1| | 3148235 | 3148252 | 18 |
| exprReg_21392 | gi|126640115|ref|NC_009085.1| | 3148401 | 3148666 | 266 |
| exprReg_21408 | gi|126640115|ref|NC_009085.1| | 3151597 | 3151626 | 30 |
| exprReg_21417 | gi|126640115|ref|NC_009085.1| | 3152777 | 3152828 | 52 |
| exprReg_21431 | gi|126640115|ref|NC_009085.1| | 3154451 | 3154507 | 57 |
| exprReg_21442 | gi|126640115|ref|NC_009085.1| | 3156591 | 3156701 | 111 |
| exprReg_21448 | gi|126640115|ref|NC_009085.1| | 3157720 | 3157866 | 147 |
| exprReg_21449 | gi|126640115|ref|NC_009085.1| | 3157884 | 3157905 | 22 |
| exprReg_21450 | gi|126640115|ref|NC_009085.1| | 3157930 | 3157971 | 42 |
| exprReg_21451 | gi|126640115|ref|NC_009085.1| | 3158117 | 3158171 | 55 |
| exprReg_21472 | gi|126640115|ref|NC_009085.1| | 3159994 | 3160012 | 19 |
| exprReg_21473 | gi|126640115|ref|NC_009085.1| | 3160029 | 3160164 | 136 |
| exprReg_21474 | gi|126640115|ref|NC_009085.1| | 3160192 | 3160210 | 19 |
| exprReg_21487 | gi|126640115|ref|NC_009085.1| | 3162050 | 3162123 | 74 |
| exprReg_21493 | gi|126640115|ref|NC_009085.1| | 3163593 | 3163632 | 40 |
| exprReg_21503 | gi|126640115|ref|NC_009085.1| | 3164639 | 3164657 | 19 |
| exprReg_21505 | gi|126640115|ref|NC_009085.1| | 3165022 | 3165054 | 33 |
| exprReg_21506 | gi|126640115|ref|NC_009085.1| | 3165059 | 3165079 | 21 |
| exprReg_21518 | gi|126640115|ref|NC_009085.1| | 3165882 | 3165902 | 21 |
| exprReg_21525 | gi|126640115|ref|NC_009085.1| | 3166665 | 3166784 | 120 |
| exprReg_21530 | gi|126640115|ref|NC_009085.1| | 3167768 | 3167816 | 49 |
| exprReg_21531 | gi|126640115|ref|NC_009085.1| | 3167835 | 3167851 | 17 |
| exprReg_21532 | gi|126640115|ref|NC_009085.1| | 3167867 | 3167895 | 29 |
| exprReg_21533 | gi|126640115|ref|NC_009085.1| | 3167946 | 3167964 | 19 |
| exprReg_21542 | gi|126640115|ref|NC_009085.1| | 3168987 | 3169049 | 63 |
| exprReg_21543 | gi|126640115|ref|NC_009085.1| | 3169069 | 3169196 | 128 |
| exprReg_21563 | gi|126640115|ref|NC_009085.1| | 3171131 | 3171341 | 211 |
| exprReg_21611 | gi|126640115|ref|NC_009085.1| | 3177888 | 3177922 | 35 |
| exprReg_21614 | gi|126640115|ref|NC_009085.1| | 3178264 | 3178307 | 44 |
| exprReg_21619 | gi|126640115|ref|NC_009085.1| | 3178744 | 3178823 | 80 |
| exprReg_21620 | gi|126640115|ref|NC_009085.1| | 3178830 | 3179121 | 292 |
| exprReg_21628 | gi|126640115|ref|NC_009085.1| | 3179863 | 3179880 | 18 |
| exprReg_21629 | gi|126640115|ref|NC_009085.1| | 3179894 | 3179910 | 17 |
| exprReg_21632 | gi|126640115|ref|NC_009085.1| | 3180418 | 3180472 | 55 |
| exprReg_21633 | gi|126640115|ref|NC_009085.1| | 3180478 | 3180546 | 69 |
| exprReg_21634 | gi|126640115|ref|NC_009085.1| | 3180666 | 3180700 | 35 |
| exprReg_21635 | gi|126640115|ref|NC_009085.1| | 3180703 | 3180732 | 30 |
| exprReg_21636 | gi|126640115|ref|NC_009085.1| | 3180816 | 3180833 | 18 |
| exprReg_21637 | gi|126640115|ref|NC_009085.1| | 3180836 | 3180903 | 68 |
| exprReg_21668 | gi|126640115|ref|NC_009085.1| | 3185485 | 3185642 | 158 |
| exprReg_21676 | gi|126640115|ref|NC_009085.1| | 3186405 | 3186596 | 192 |
| exprReg_21677 | gi|126640115|ref|NC_009085.1| | 3186676 | 3186699 | 24 |
| exprReg_21678 | gi|126640115|ref|NC_009085.1| | 3186733 | 3186780 | 48 |
| exprReg_21679 | gi|126640115|ref|NC_009085.1| | 3186788 | 3186852 | 65 |
| exprReg_21680 | gi|126640115|ref|NC_009085.1| | 3186856 | 3186874 | 19 |
| exprReg_21688 | gi|126640115|ref|NC_009085.1| | 3187699 | 3187725 | 27 |
| exprReg_21696 | gi|126640115|ref|NC_009085.1| | 3189062 | 3189126 | 65 |
| exprReg_21697 | gi|126640115|ref|NC_009085.1| | 3189181 | 3189205 | 25 |
| exprReg_21718 | gi|126640115|ref|NC_009085.1| | 3191920 | 3191948 | 29 |
| exprReg_21728 | gi|126640115|ref|NC_009085.1| | 3193572 | 3193618 | 47 |
| exprReg_21729 | gi|126640115|ref|NC_009085.1| | 3193647 | 3193737 | 91 |
| exprReg_21730 | gi|126640115|ref|NC_009085.1| | 3193740 | 3193783 | 44 |
| exprReg_21737 | gi|126640115|ref|NC_009085.1| | 3195063 | 3195228 | 166 |
| exprReg_21738 | gi|126640115|ref|NC_009085.1| | 3195231 | 3195252 | 22 |
| exprReg_21739 | gi|126640115|ref|NC_009085.1| | 3195264 | 3195289 | 26 |
| exprReg_21740 | gi|126640115|ref|NC_009085.1| | 3195295 | 3195311 | 17 |
| exprReg_21741 | gi|126640115|ref|NC_009085.1| | 3195418 | 3195435 | 18 |
| exprReg_21742 | gi|126640115|ref|NC_009085.1| | 3195447 | 3195463 | 17 |
| exprReg_21743 | gi|126640115|ref|NC_009085.1| | 3195470 | 3195493 | 24 |
| exprReg_21744 | gi|126640115|ref|NC_009085.1| | 3195603 | 3195624 | 22 |
| exprReg_21755 | gi|126640115|ref|NC_009085.1| | 3196902 | 3197052 | 151 |
| exprReg_21762 | gi|126640115|ref|NC_009085.1| | 3198650 | 3198744 | 95 |
| exprReg_21763 | gi|126640115|ref|NC_009085.1| | 3198749 | 3198813 | 65 |
| exprReg_21768 | gi|126640115|ref|NC_009085.1| | 3199336 | 3199395 | 60 |
| exprReg_21769 | gi|126640115|ref|NC_009085.1| | 3199428 | 3199462 | 35 |
| exprReg_21775 | gi|126640115|ref|NC_009085.1| | 3200099 | 3200118 | 20 |
| exprReg_21778 | gi|126640115|ref|NC_009085.1| | 3200473 | 3200522 | 50 |
| exprReg_21788 | gi|126640115|ref|NC_009085.1| | 3203073 | 3203131 | 59 |
| exprReg_21796 | gi|126640115|ref|NC_009085.1| | 3203910 | 3203959 | 50 |
| exprReg_21797 | gi|126640115|ref|NC_009085.1| | 3203997 | 3204026 | 30 |
| exprReg_21828 | gi|126640115|ref|NC_009085.1| | 3208775 | 3208821 | 47 |
| exprReg_21829 | gi|126640115|ref|NC_009085.1| | 3208881 | 3208941 | 61 |
| exprReg_21830 | gi|126640115|ref|NC_009085.1| | 3209078 | 3209133 | 56 |
| exprReg_21843 | gi|126640115|ref|NC_009085.1| | 3211872 | 3211889 | 18 |
| exprReg_21844 | gi|126640115|ref|NC_009085.1| | 3211942 | 3211969 | 28 |
| exprReg_21845 | gi|126640115|ref|NC_009085.1| | 3212005 | 3212171 | 167 |
| exprReg_21848 | gi|126640115|ref|NC_009085.1| | 3212937 | 3212979 | 43 |
| exprReg_21849 | gi|126640115|ref|NC_009085.1| | 3213095 | 3213117 | 23 |
| exprReg_21850 | gi|126640115|ref|NC_009085.1| | 3213397 | 3213507 | 111 |
| exprReg_21851 | gi|126640115|ref|NC_009085.1| | 3213519 | 3213574 | 56 |
| exprReg_21868 | gi|126640115|ref|NC_009085.1| | 3215819 | 3215931 | 113 |
| exprReg_21874 | gi|126640115|ref|NC_009085.1| | 3216732 | 3216821 | 90 |
| exprReg_21887 | gi|126640115|ref|NC_009085.1| | 3218193 | 3218209 | 17 |
| exprReg_21888 | gi|126640115|ref|NC_009085.1| | 3218213 | 3218300 | 88 |
| exprReg_21889 | gi|126640115|ref|NC_009085.1| | 3218302 | 3218451 | 150 |
| exprReg_21915 | gi|126640115|ref|NC_009085.1| | 3221887 | 3221974 | 88 |
| exprReg_21925 | gi|126640115|ref|NC_009085.1| | 3223506 | 3223622 | 117 |
| exprReg_21946 | gi|126640115|ref|NC_009085.1| | 3226597 | 3226629 | 33 |
| exprReg_21957 | gi|126640115|ref|NC_009085.1| | 3228806 | 3228903 | 98 |
| exprReg_21958 | gi|126640115|ref|NC_009085.1| | 3228922 | 3229051 | 130 |
| exprReg_21979 | gi|126640115|ref|NC_009085.1| | 3232123 | 3232139 | 17 |
| exprReg_21980 | gi|126640115|ref|NC_009085.1| | 3232156 | 3232195 | 40 |
| exprReg_21995 | gi|126640115|ref|NC_009085.1| | 3233493 | 3233509 | 17 |
| exprReg_21996 | gi|126640115|ref|NC_009085.1| | 3233513 | 3233531 | 19 |
| exprReg_21997 | gi|126640115|ref|NC_009085.1| | 3233564 | 3233580 | 17 |
| exprReg_22007 | gi|126640115|ref|NC_009085.1| | 3234805 | 3234825 | 21 |
| exprReg_22008 | gi|126640115|ref|NC_009085.1| | 3234830 | 3234868 | 39 |
| exprReg_22009 | gi|126640115|ref|NC_009085.1| | 3234940 | 3234973 | 34 |
| exprReg_22017 | gi|126640115|ref|NC_009085.1| | 3235888 | 3235919 | 32 |
| exprReg_22018 | gi|126640115|ref|NC_009085.1| | 3235933 | 3236101 | 169 |
| exprReg_22019 | gi|126640115|ref|NC_009085.1| | 3236157 | 3236215 | 59 |
| exprReg_22029 | gi|126640115|ref|NC_009085.1| | 3237708 | 3237794 | 87 |
| exprReg_22030 | gi|126640115|ref|NC_009085.1| | 3237803 | 3237819 | 17 |
| exprReg_22031 | gi|126640115|ref|NC_009085.1| | 3237822 | 3237839 | 18 |
| exprReg_22032 | gi|126640115|ref|NC_009085.1| | 3237917 | 3238148 | 232 |
| exprReg_22033 | gi|126640115|ref|NC_009085.1| | 3238185 | 3238286 | 102 |
| exprReg_22034 | gi|126640115|ref|NC_009085.1| | 3238319 | 3238361 | 43 |
| exprReg_22035 | gi|126640115|ref|NC_009085.1| | 3238433 | 3238489 | 57 |
| exprReg_22036 | gi|126640115|ref|NC_009085.1| | 3238676 | 3238721 | 46 |
| exprReg_22037 | gi|126640115|ref|NC_009085.1| | 3238764 | 3238780 | 17 |
| exprReg_22038 | gi|126640115|ref|NC_009085.1| | 3238840 | 3238856 | 17 |
| exprReg_22039 | gi|126640115|ref|NC_009085.1| | 3238864 | 3238945 | 82 |
| exprReg_22040 | gi|126640115|ref|NC_009085.1| | 3238954 | 3238988 | 35 |
| exprReg_22058 | gi|126640115|ref|NC_009085.1| | 3240761 | 3240808 | 48 |
| exprReg_22059 | gi|126640115|ref|NC_009085.1| | 3240884 | 3240923 | 40 |
| exprReg_22079 | gi|126640115|ref|NC_009085.1| | 3243204 | 3243282 | 79 |
| exprReg_22082 | gi|126640115|ref|NC_009085.1| | 3243779 | 3243796 | 18 |
| exprReg_22083 | gi|126640115|ref|NC_009085.1| | 3243834 | 3243896 | 63 |
| exprReg_22101 | gi|126640115|ref|NC_009085.1| | 3246598 | 3246631 | 34 |
| exprReg_22108 | gi|126640115|ref|NC_009085.1| | 3247458 | 3247479 | 22 |
| exprReg_22114 | gi|126640115|ref|NC_009085.1| | 3249002 | 3249019 | 18 |
| exprReg_22117 | gi|126640115|ref|NC_009085.1| | 3249724 | 3249763 | 40 |
| exprReg_22118 | gi|126640115|ref|NC_009085.1| | 3249768 | 3249799 | 32 |
| exprReg_22126 | gi|126640115|ref|NC_009085.1| | 3250808 | 3250826 | 19 |
| exprReg_22134 | gi|126640115|ref|NC_009085.1| | 3252159 | 3252249 | 91 |
| exprReg_22192 | gi|126640115|ref|NC_009085.1| | 3260671 | 3260709 | 39 |
| exprReg_22205 | gi|126640115|ref|NC_009085.1| | 3262763 | 3262812 | 50 |
| exprReg_22258 | gi|126640115|ref|NC_009085.1| | 3267643 | 3267659 | 17 |
| exprReg_22268 | gi|126640115|ref|NC_009085.1| | 3269529 | 3269618 | 90 |
| exprReg_22269 | gi|126640115|ref|NC_009085.1| | 3269863 | 3269879 | 17 |
| exprReg_22275 | gi|126640115|ref|NC_009085.1| | 3270621 | 3270642 | 22 |
| exprReg_22276 | gi|126640115|ref|NC_009085.1| | 3270669 | 3270717 | 49 |
| exprReg_22277 | gi|126640115|ref|NC_009085.1| | 3270723 | 3270751 | 29 |
| exprReg_22278 | gi|126640115|ref|NC_009085.1| | 3270815 | 3270858 | 44 |
| exprReg_22284 | gi|126640115|ref|NC_009085.1| | 3271444 | 3271491 | 48 |
| exprReg_22285 | gi|126640115|ref|NC_009085.1| | 3271531 | 3271547 | 17 |
| exprReg_22286 | gi|126640115|ref|NC_009085.1| | 3271550 | 3271571 | 22 |
| exprReg_22287 | gi|126640115|ref|NC_009085.1| | 3271644 | 3271660 | 17 |
| exprReg_22288 | gi|126640115|ref|NC_009085.1| | 3271692 | 3271708 | 17 |
| exprReg_22289 | gi|126640115|ref|NC_009085.1| | 3271725 | 3271776 | 52 |
| exprReg_22304 | gi|126640115|ref|NC_009085.1| | 3273126 | 3273256 | 131 |
| exprReg_22305 | gi|126640115|ref|NC_009085.1| | 3273271 | 3273334 | 64 |
| exprReg_22306 | gi|126640115|ref|NC_009085.1| | 3273364 | 3273432 | 69 |
| exprReg_22312 | gi|126640115|ref|NC_009085.1| | 3274478 | 3274498 | 21 |
| exprReg_22326 | gi|126640115|ref|NC_009085.1| | 3276614 | 3276669 | 56 |
| exprReg_22328 | gi|126640115|ref|NC_009085.1| | 3277468 | 3277523 | 56 |
| exprReg_22329 | gi|126640115|ref|NC_009085.1| | 3277529 | 3277647 | 119 |
| exprReg_22342 | gi|126640115|ref|NC_009085.1| | 3278953 | 3278971 | 19 |
| exprReg_22343 | gi|126640115|ref|NC_009085.1| | 3279024 | 3279103 | 80 |
| exprReg_22346 | gi|126640115|ref|NC_009085.1| | 3279599 | 3279648 | 50 |
| exprReg_22347 | gi|126640115|ref|NC_009085.1| | 3279657 | 3279697 | 41 |
| exprReg_22362 | gi|126640115|ref|NC_009085.1| | 3281651 | 3281676 | 26 |
| exprReg_22363 | gi|126640115|ref|NC_009085.1| | 3281686 | 3281711 | 26 |
| exprReg_22364 | gi|126640115|ref|NC_009085.1| | 3281802 | 3281920 | 119 |
| exprReg_22365 | gi|126640115|ref|NC_009085.1| | 3281944 | 3281961 | 18 |
| exprReg_22366 | gi|126640115|ref|NC_009085.1| | 3281971 | 3281992 | 22 |
| exprReg_22367 | gi|126640115|ref|NC_009085.1| | 3282005 | 3282037 | 33 |
| exprReg_22379 | gi|126640115|ref|NC_009085.1| | 3283362 | 3283379 | 18 |
| exprReg_22380 | gi|126640115|ref|NC_009085.1| | 3283401 | 3283421 | 21 |
| exprReg_22386 | gi|126640115|ref|NC_009085.1| | 3284366 | 3284386 | 21 |
| exprReg_22387 | gi|126640115|ref|NC_009085.1| | 3284390 | 3284493 | 104 |
| exprReg_22396 | gi|126640115|ref|NC_009085.1| | 3285819 | 3285842 | 24 |
| exprReg_22397 | gi|126640115|ref|NC_009085.1| | 3285855 | 3285999 | 145 |
| exprReg_22398 | gi|126640115|ref|NC_009085.1| | 3286093 | 3286115 | 23 |
| exprReg_22416 | gi|126640115|ref|NC_009085.1| | 3289692 | 3289736 | 45 |
| exprReg_22431 | gi|126640115|ref|NC_009085.1| | 3291838 | 3291857 | 20 |
| exprReg_22449 | gi|126640115|ref|NC_009085.1| | 3293876 | 3294035 | 160 |
| exprReg_22465 | gi|126640115|ref|NC_009085.1| | 3296369 | 3296427 | 59 |
| exprReg_22466 | gi|126640115|ref|NC_009085.1| | 3296451 | 3296619 | 169 |
| exprReg_22467 | gi|126640115|ref|NC_009085.1| | 3296623 | 3296721 | 99 |
| exprReg_22477 | gi|126640115|ref|NC_009085.1| | 3297943 | 3298004 | 62 |
| exprReg_22490 | gi|126640115|ref|NC_009085.1| | 3299307 | 3299331 | 25 |
| exprReg_22491 | gi|126640115|ref|NC_009085.1| | 3299388 | 3299410 | 23 |
| exprReg_22499 | gi|126640115|ref|NC_009085.1| | 3300670 | 3300752 | 83 |
| exprReg_22500 | gi|126640115|ref|NC_009085.1| | 3300825 | 3300892 | 68 |
| exprReg_22505 | gi|126640115|ref|NC_009085.1| | 3301839 | 3301910 | 72 |
| exprReg_22508 | gi|126640115|ref|NC_009085.1| | 3302447 | 3302466 | 20 |
| exprReg_22519 | gi|126640115|ref|NC_009085.1| | 3304113 | 3304147 | 35 |
| exprReg_22544 | gi|126640115|ref|NC_009085.1| | 3306949 | 3306983 | 35 |
| exprReg_22545 | gi|126640115|ref|NC_009085.1| | 3307088 | 3307104 | 17 |
| exprReg_22551 | gi|126640115|ref|NC_009085.1| | 3307879 | 3307981 | 103 |
| exprReg_22565 | gi|126640115|ref|NC_009085.1| | 3309674 | 3309721 | 48 |
| exprReg_22588 | gi|126640115|ref|NC_009085.1| | 3312731 | 3312749 | 19 |
| exprReg_22589 | gi|126640115|ref|NC_009085.1| | 3312752 | 3312773 | 22 |
| exprReg_22590 | gi|126640115|ref|NC_009085.1| | 3312951 | 3312983 | 33 |
| exprReg_22591 | gi|126640115|ref|NC_009085.1| | 3313078 | 3313097 | 20 |
| exprReg_22594 | gi|126640115|ref|NC_009085.1| | 3313655 | 3313676 | 22 |
| exprReg_22595 | gi|126640115|ref|NC_009085.1| | 3313688 | 3313808 | 121 |
| exprReg_22596 | gi|126640115|ref|NC_009085.1| | 3313879 | 3313951 | 73 |
| exprReg_22597 | gi|126640115|ref|NC_009085.1| | 3313955 | 3314102 | 148 |
| exprReg_22614 | gi|126640115|ref|NC_009085.1| | 3316213 | 3316232 | 20 |
| exprReg_22615 | gi|126640115|ref|NC_009085.1| | 3316243 | 3316259 | 17 |
| exprReg_22625 | gi|126640115|ref|NC_009085.1| | 3318340 | 3318461 | 122 |
| exprReg_22630 | gi|126640115|ref|NC_009085.1| | 3319387 | 3319491 | 105 |
| exprReg_22635 | gi|126640115|ref|NC_009085.1| | 3320541 | 3320584 | 44 |
| exprReg_22636 | gi|126640115|ref|NC_009085.1| | 3320709 | 3320727 | 19 |
| exprReg_22664 | gi|126640115|ref|NC_009085.1| | 3324891 | 3324907 | 17 |
| exprReg_22665 | gi|126640115|ref|NC_009085.1| | 3324929 | 3324961 | 33 |
| exprReg_22666 | gi|126640115|ref|NC_009085.1| | 3324972 | 3325027 | 56 |
| exprReg_22667 | gi|126640115|ref|NC_009085.1| | 3325094 | 3325110 | 17 |
| exprReg_22668 | gi|126640115|ref|NC_009085.1| | 3325113 | 3325160 | 48 |
| exprReg_22669 | gi|126640115|ref|NC_009085.1| | 3325204 | 3325392 | 189 |
| exprReg_22686 | gi|126640115|ref|NC_009085.1| | 3327351 | 3327395 | 45 |
| exprReg_22687 | gi|126640115|ref|NC_009085.1| | 3327400 | 3327447 | 48 |
| exprReg_22692 | gi|126640115|ref|NC_009085.1| | 3328068 | 3328160 | 93 |
| exprReg_22702 | gi|126640115|ref|NC_009085.1| | 3329341 | 3329415 | 75 |
| exprReg_22713 | gi|126640115|ref|NC_009085.1| | 3330816 | 3330923 | 108 |
| exprReg_22726 | gi|126640115|ref|NC_009085.1| | 3332958 | 3332975 | 18 |
| exprReg_22738 | gi|126640115|ref|NC_009085.1| | 3334602 | 3334625 | 24 |
| exprReg_22739 | gi|126640115|ref|NC_009085.1| | 3334658 | 3334677 | 20 |
| exprReg_22742 | gi|126640115|ref|NC_009085.1| | 3335367 | 3335557 | 191 |
| exprReg_22756 | gi|126640115|ref|NC_009085.1| | 3337426 | 3337495 | 70 |
| exprReg_22763 | gi|126640115|ref|NC_009085.1| | 3339367 | 3339386 | 20 |
| exprReg_22771 | gi|126640115|ref|NC_009085.1| | 3340262 | 3340286 | 25 |
| exprReg_22772 | gi|126640115|ref|NC_009085.1| | 3340319 | 3340342 | 24 |
| exprReg_22773 | gi|126640115|ref|NC_009085.1| | 3340458 | 3340474 | 17 |
| exprReg_22778 | gi|126640115|ref|NC_009085.1| | 3340896 | 3341060 | 165 |
| exprReg_22810 | gi|126640115|ref|NC_009085.1| | 3345681 | 3345864 | 184 |
| exprReg_22822 | gi|126640115|ref|NC_009085.1| | 3347610 | 3347711 | 102 |
| exprReg_22826 | gi|126640115|ref|NC_009085.1| | 3347912 | 3347931 | 20 |
| exprReg_22827 | gi|126640115|ref|NC_009085.1| | 3347951 | 3347972 | 22 |
| exprReg_22828 | gi|126640115|ref|NC_009085.1| | 3347978 | 3348018 | 41 |
| exprReg_22846 | gi|126640115|ref|NC_009085.1| | 3349825 | 3349846 | 22 |
| exprReg_22847 | gi|126640115|ref|NC_009085.1| | 3349952 | 3349970 | 19 |
| exprReg_22848 | gi|126640115|ref|NC_009085.1| | 3349973 | 3350083 | 111 |
| exprReg_22849 | gi|126640115|ref|NC_009085.1| | 3350124 | 3350143 | 20 |
| exprReg_22850 | gi|126640115|ref|NC_009085.1| | 3350291 | 3350310 | 20 |
| exprReg_22851 | gi|126640115|ref|NC_009085.1| | 3350358 | 3350431 | 74 |
| exprReg_22852 | gi|126640115|ref|NC_009085.1| | 3350505 | 3350531 | 27 |
| exprReg_22853 | gi|126640115|ref|NC_009085.1| | 3350545 | 3350577 | 33 |
| exprReg_22854 | gi|126640115|ref|NC_009085.1| | 3350617 | 3350657 | 41 |
| exprReg_22855 | gi|126640115|ref|NC_009085.1| | 3350692 | 3350708 | 17 |
| exprReg_22856 | gi|126640115|ref|NC_009085.1| | 3350770 | 3350842 | 73 |
| exprReg_22857 | gi|126640115|ref|NC_009085.1| | 3350880 | 3350899 | 20 |
| exprReg_22880 | gi|126640115|ref|NC_009085.1| | 3353483 | 3353523 | 41 |
| exprReg_22889 | gi|126640115|ref|NC_009085.1| | 3355278 | 3355295 | 18 |
| exprReg_22890 | gi|126640115|ref|NC_009085.1| | 3355307 | 3355369 | 63 |
| exprReg_22899 | gi|126640115|ref|NC_009085.1| | 3356249 | 3356265 | 17 |
| exprReg_22931 | gi|126640115|ref|NC_009085.1| | 3361831 | 3361912 | 82 |
| exprReg_22932 | gi|126640115|ref|NC_009085.1| | 3362184 | 3362240 | 57 |
| exprReg_22933 | gi|126640115|ref|NC_009085.1| | 3362246 | 3362264 | 19 |
| exprReg_22934 | gi|126640115|ref|NC_009085.1| | 3362458 | 3362481 | 24 |
| exprReg_22935 | gi|126640115|ref|NC_009085.1| | 3362499 | 3362524 | 26 |
| exprReg_22936 | gi|126640115|ref|NC_009085.1| | 3362552 | 3362568 | 17 |
| exprReg_22937 | gi|126640115|ref|NC_009085.1| | 3362606 | 3362626 | 21 |
| exprReg_22938 | gi|126640115|ref|NC_009085.1| | 3362662 | 3362683 | 22 |
| exprReg_22939 | gi|126640115|ref|NC_009085.1| | 3362702 | 3362724 | 23 |
| exprReg_22940 | gi|126640115|ref|NC_009085.1| | 3362741 | 3362783 | 43 |
| exprReg_22941 | gi|126640115|ref|NC_009085.1| | 3362809 | 3362856 | 48 |
| exprReg_22942 | gi|126640115|ref|NC_009085.1| | 3362859 | 3362890 | 32 |
| exprReg_22943 | gi|126640115|ref|NC_009085.1| | 3362921 | 3363018 | 98 |
| exprReg_22944 | gi|126640115|ref|NC_009085.1| | 3363056 | 3363078 | 23 |
| exprReg_22945 | gi|126640115|ref|NC_009085.1| | 3363211 | 3363230 | 20 |
| exprReg_22946 | gi|126640115|ref|NC_009085.1| | 3363248 | 3363266 | 19 |
| exprReg_22967 | gi|126640115|ref|NC_009085.1| | 3367021 | 3367054 | 34 |
| exprReg_22968 | gi|126640115|ref|NC_009085.1| | 3367056 | 3367167 | 112 |
| exprReg_22969 | gi|126640115|ref|NC_009085.1| | 3367340 | 3367434 | 95 |
| exprReg_22970 | gi|126640115|ref|NC_009085.1| | 3367625 | 3367656 | 32 |
| exprReg_22971 | gi|126640115|ref|NC_009085.1| | 3367690 | 3367716 | 27 |
| exprReg_22972 | gi|126640115|ref|NC_009085.1| | 3367866 | 3367889 | 24 |
| exprReg_22973 | gi|126640115|ref|NC_009085.1| | 3367897 | 3367921 | 25 |
| exprReg_22974 | gi|126640115|ref|NC_009085.1| | 3367956 | 3367972 | 17 |
| exprReg_22975 | gi|126640115|ref|NC_009085.1| | 3367985 | 3368005 | 21 |
| exprReg_22976 | gi|126640115|ref|NC_009085.1| | 3368017 | 3368075 | 59 |
| exprReg_22977 | gi|126640115|ref|NC_009085.1| | 3368087 | 3368106 | 20 |
| exprReg_22978 | gi|126640115|ref|NC_009085.1| | 3368228 | 3368278 | 51 |
| exprReg_22979 | gi|126640115|ref|NC_009085.1| | 3368291 | 3368402 | 112 |
| exprReg_22980 | gi|126640115|ref|NC_009085.1| | 3368438 | 3368534 | 97 |
| exprReg_22981 | gi|126640115|ref|NC_009085.1| | 3368615 | 3368662 | 48 |
| exprReg_22982 | gi|126640115|ref|NC_009085.1| | 3368818 | 3368837 | 20 |
| exprReg_22983 | gi|126640115|ref|NC_009085.1| | 3368890 | 3368987 | 98 |
| exprReg_22984 | gi|126640115|ref|NC_009085.1| | 3369003 | 3369059 | 57 |
| exprReg_22985 | gi|126640115|ref|NC_009085.1| | 3369262 | 3369308 | 47 |
| exprReg_22986 | gi|126640115|ref|NC_009085.1| | 3369319 | 3369339 | 21 |
| exprReg_22987 | gi|126640115|ref|NC_009085.1| | 3369390 | 3369410 | 21 |
| exprReg_22988 | gi|126640115|ref|NC_009085.1| | 3369568 | 3369590 | 23 |
| exprReg_22989 | gi|126640115|ref|NC_009085.1| | 3369619 | 3369641 | 23 |
| exprReg_22990 | gi|126640115|ref|NC_009085.1| | 3369679 | 3369715 | 37 |
| exprReg_22991 | gi|126640115|ref|NC_009085.1| | 3369826 | 3369842 | 17 |
| exprReg_23000 | gi|126640115|ref|NC_009085.1| | 3371073 | 3371241 | 169 |
| exprReg_23001 | gi|126640115|ref|NC_009085.1| | 3371248 | 3371268 | 21 |
| exprReg_23002 | gi|126640115|ref|NC_009085.1| | 3371281 | 3371331 | 51 |
| exprReg_23003 | gi|126640115|ref|NC_009085.1| | 3371350 | 3371369 | 20 |
| exprReg_23005 | gi|126640115|ref|NC_009085.1| | 3371656 | 3371682 | 27 |
| exprReg_23006 | gi|126640115|ref|NC_009085.1| | 3371733 | 3371761 | 29 |
| exprReg_23013 | gi|126640115|ref|NC_009085.1| | 3372851 | 3372911 | 61 |
| exprReg_23014 | gi|126640115|ref|NC_009085.1| | 3372943 | 3372976 | 34 |
| exprReg_23015 | gi|126640115|ref|NC_009085.1| | 3372984 | 3373040 | 57 |
| exprReg_23016 | gi|126640115|ref|NC_009085.1| | 3373051 | 3373168 | 118 |
| exprReg_23017 | gi|126640115|ref|NC_009085.1| | 3373245 | 3373285 | 41 |
| exprReg_23018 | gi|126640115|ref|NC_009085.1| | 3373318 | 3373340 | 23 |
| exprReg_23019 | gi|126640115|ref|NC_009085.1| | 3373375 | 3373395 | 21 |
| exprReg_23020 | gi|126640115|ref|NC_009085.1| | 3373406 | 3373423 | 18 |
| exprReg_23021 | gi|126640115|ref|NC_009085.1| | 3373510 | 3373566 | 57 |
| exprReg_23022 | gi|126640115|ref|NC_009085.1| | 3373600 | 3373681 | 82 |
| exprReg_23023 | gi|126640115|ref|NC_009085.1| | 3373751 | 3373854 | 104 |
| exprReg_23039 | gi|126640115|ref|NC_009085.1| | 3376303 | 3376320 | 18 |
| exprReg_23063 | gi|126640115|ref|NC_009085.1| | 3380472 | 3380551 | 80 |
| exprReg_23067 | gi|126640115|ref|NC_009085.1| | 3381431 | 3381452 | 22 |
| exprReg_23068 | gi|126640115|ref|NC_009085.1| | 3381466 | 3381496 | 31 |
| exprReg_23087 | gi|126640115|ref|NC_009085.1| | 3384414 | 3384433 | 20 |
| exprReg_23088 | gi|126640115|ref|NC_009085.1| | 3384458 | 3384526 | 69 |
| exprReg_23089 | gi|126640115|ref|NC_009085.1| | 3384534 | 3384568 | 35 |
| exprReg_23090 | gi|126640115|ref|NC_009085.1| | 3384577 | 3384614 | 38 |
| exprReg_23115 | gi|126640115|ref|NC_009085.1| | 3387605 | 3387631 | 27 |
| exprReg_23116 | gi|126640115|ref|NC_009085.1| | 3387650 | 3387667 | 18 |
| exprReg_23128 | gi|126640115|ref|NC_009085.1| | 3389035 | 3389054 | 20 |
| exprReg_23129 | gi|126640115|ref|NC_009085.1| | 3389105 | 3389128 | 24 |
| exprReg_23130 | gi|126640115|ref|NC_009085.1| | 3389205 | 3389229 | 25 |
| exprReg_23131 | gi|126640115|ref|NC_009085.1| | 3389262 | 3389424 | 163 |
| exprReg_23132 | gi|126640115|ref|NC_009085.1| | 3389510 | 3389626 | 117 |
| exprReg_23133 | gi|126640115|ref|NC_009085.1| | 3389668 | 3389691 | 24 |
| exprReg_23134 | gi|126640115|ref|NC_009085.1| | 3389712 | 3389760 | 49 |
| exprReg_23135 | gi|126640115|ref|NC_009085.1| | 3389826 | 3389906 | 81 |
| exprReg_23136 | gi|126640115|ref|NC_009085.1| | 3390014 | 3390118 | 105 |
| exprReg_23137 | gi|126640115|ref|NC_009085.1| | 3390181 | 3390197 | 17 |
| exprReg_23138 | gi|126640115|ref|NC_009085.1| | 3390332 | 3390353 | 22 |
| exprReg_23139 | gi|126640115|ref|NC_009085.1| | 3390402 | 3390451 | 50 |
| exprReg_23140 | gi|126640115|ref|NC_009085.1| | 3390607 | 3390628 | 22 |
| exprReg_23141 | gi|126640115|ref|NC_009085.1| | 3390634 | 3390692 | 59 |
| exprReg_23142 | gi|126640115|ref|NC_009085.1| | 3390696 | 3390713 | 18 |
| exprReg_23167 | gi|126640115|ref|NC_009085.1| | 3393555 | 3393599 | 45 |
| exprReg_23168 | gi|126640115|ref|NC_009085.1| | 3393633 | 3393679 | 47 |
| exprReg_23169 | gi|126640115|ref|NC_009085.1| | 3393711 | 3393887 | 177 |
| exprReg_23170 | gi|126640115|ref|NC_009085.1| | 3393990 | 3394006 | 17 |
| exprReg_23171 | gi|126640115|ref|NC_009085.1| | 3394009 | 3394028 | 20 |
| exprReg_23172 | gi|126640115|ref|NC_009085.1| | 3394048 | 3394091 | 44 |
| exprReg_23173 | gi|126640115|ref|NC_009085.1| | 3394106 | 3394184 | 79 |
| exprReg_23185 | gi|126640115|ref|NC_009085.1| | 3395768 | 3395786 | 19 |
| exprReg_23186 | gi|126640115|ref|NC_009085.1| | 3395810 | 3395830 | 21 |
| exprReg_23187 | gi|126640115|ref|NC_009085.1| | 3395906 | 3395991 | 86 |
| exprReg_23196 | gi|126640115|ref|NC_009085.1| | 3397666 | 3397684 | 19 |
| exprReg_23199 | gi|126640115|ref|NC_009085.1| | 3398006 | 3398022 | 17 |
| exprReg_23219 | gi|126640115|ref|NC_009085.1| | 3401041 | 3401176 | 136 |
| exprReg_23235 | gi|126640115|ref|NC_009085.1| | 3403969 | 3404010 | 42 |
| exprReg_23236 | gi|126640115|ref|NC_009085.1| | 3404154 | 3404172 | 19 |
| exprReg_23237 | gi|126640115|ref|NC_009085.1| | 3404221 | 3404242 | 22 |
| exprReg_23238 | gi|126640115|ref|NC_009085.1| | 3404267 | 3404341 | 75 |
| exprReg_23239 | gi|126640115|ref|NC_009085.1| | 3404381 | 3404399 | 19 |
| exprReg_23240 | gi|126640115|ref|NC_009085.1| | 3404418 | 3404646 | 229 |
| exprReg_23266 | gi|126640115|ref|NC_009085.1| | 3407689 | 3407760 | 72 |
| exprReg_23267 | gi|126640115|ref|NC_009085.1| | 3407762 | 3408023 | 262 |
| exprReg_23283 | gi|126640115|ref|NC_009085.1| | 3410172 | 3410233 | 62 |
| exprReg_23299 | gi|126640115|ref|NC_009085.1| | 3412510 | 3412559 | 50 |
| exprReg_23300 | gi|126640115|ref|NC_009085.1| | 3412745 | 3412821 | 77 |
| exprReg_23304 | gi|126640115|ref|NC_009085.1| | 3413265 | 3413300 | 36 |
| exprReg_23314 | gi|126640115|ref|NC_009085.1| | 3414320 | 3414397 | 78 |
| exprReg_23322 | gi|126640115|ref|NC_009085.1| | 3415479 | 3415510 | 32 |
| exprReg_23323 | gi|126640115|ref|NC_009085.1| | 3415607 | 3415657 | 51 |
| exprReg_23324 | gi|126640115|ref|NC_009085.1| | 3415661 | 3415751 | 91 |
| exprReg_23325 | gi|126640115|ref|NC_009085.1| | 3415765 | 3415981 | 217 |
| exprReg_23326 | gi|126640115|ref|NC_009085.1| | 3416115 | 3416179 | 65 |
| exprReg_23327 | gi|126640115|ref|NC_009085.1| | 3416335 | 3416515 | 181 |
| exprReg_23328 | gi|126640115|ref|NC_009085.1| | 3416547 | 3416844 | 298 |
| exprReg_23329 | gi|126640115|ref|NC_009085.1| | 3416894 | 3416952 | 59 |
| exprReg_23330 | gi|126640115|ref|NC_009085.1| | 3416994 | 3417245 | 252 |
| exprReg_23331 | gi|126640115|ref|NC_009085.1| | 3417340 | 3417464 | 125 |
| exprReg_23332 | gi|126640115|ref|NC_009085.1| | 3417468 | 3417508 | 41 |
| exprReg_23333 | gi|126640115|ref|NC_009085.1| | 3417522 | 3417550 | 29 |
| exprReg_23334 | gi|126640115|ref|NC_009085.1| | 3417570 | 3417598 | 29 |
| exprReg_23335 | gi|126640115|ref|NC_009085.1| | 3417748 | 3417777 | 30 |
| exprReg_23336 | gi|126640115|ref|NC_009085.1| | 3417838 | 3417916 | 79 |
| exprReg_23337 | gi|126640115|ref|NC_009085.1| | 3418023 | 3418233 | 211 |
| exprReg_23338 | gi|126640115|ref|NC_009085.1| | 3418429 | 3418563 | 135 |
| exprReg_23339 | gi|126640115|ref|NC_009085.1| | 3418665 | 3418712 | 48 |
| exprReg_23340 | gi|126640115|ref|NC_009085.1| | 3418790 | 3418815 | 26 |
| exprReg_23341 | gi|126640115|ref|NC_009085.1| | 3418937 | 3419153 | 217 |
| exprReg_23342 | gi|126640115|ref|NC_009085.1| | 3419248 | 3419277 | 30 |
| exprReg_23343 | gi|126640115|ref|NC_009085.1| | 3419296 | 3419402 | 107 |
| exprReg_23344 | gi|126640115|ref|NC_009085.1| | 3419425 | 3419460 | 36 |
| exprReg_23345 | gi|126640115|ref|NC_009085.1| | 3419512 | 3419866 | 355 |
| exprReg_23346 | gi|126640115|ref|NC_009085.1| | 3419899 | 3419916 | 18 |
| exprReg_23347 | gi|126640115|ref|NC_009085.1| | 3419920 | 3419969 | 50 |
| exprReg_23348 | gi|126640115|ref|NC_009085.1| | 3419971 | 3419989 | 19 |
| exprReg_23349 | gi|126640115|ref|NC_009085.1| | 3419992 | 3420057 | 66 |
| exprReg_23350 | gi|126640115|ref|NC_009085.1| | 3420062 | 3420197 | 136 |
| exprReg_23351 | gi|126640115|ref|NC_009085.1| | 3420298 | 3420336 | 39 |
| exprReg_23352 | gi|126640115|ref|NC_009085.1| | 3420388 | 3420409 | 22 |
| exprReg_23359 | gi|126640115|ref|NC_009085.1| | 3421807 | 3421872 | 66 |
| exprReg_23360 | gi|126640115|ref|NC_009085.1| | 3421882 | 3422008 | 127 |
| exprReg_23361 | gi|126640115|ref|NC_009085.1| | 3422081 | 3422099 | 19 |
| exprReg_23362 | gi|126640115|ref|NC_009085.1| | 3422128 | 3422227 | 100 |
| exprReg_23363 | gi|126640115|ref|NC_009085.1| | 3422293 | 3422334 | 42 |
| exprReg_23364 | gi|126640115|ref|NC_009085.1| | 3422340 | 3422360 | 21 |
| exprReg_23372 | gi|126640115|ref|NC_009085.1| | 3423300 | 3423317 | 18 |
| exprReg_23373 | gi|126640115|ref|NC_009085.1| | 3423368 | 3423384 | 17 |
| exprReg_23374 | gi|126640115|ref|NC_009085.1| | 3423398 | 3423468 | 71 |
| exprReg_23375 | gi|126640115|ref|NC_009085.1| | 3423491 | 3423514 | 24 |
| exprReg_23376 | gi|126640115|ref|NC_009085.1| | 3423623 | 3423645 | 23 |
| exprReg_23377 | gi|126640115|ref|NC_009085.1| | 3423722 | 3423749 | 28 |
| exprReg_23378 | gi|126640115|ref|NC_009085.1| | 3423817 | 3423910 | 94 |
| exprReg_23379 | gi|126640115|ref|NC_009085.1| | 3423965 | 3423982 | 18 |
| exprReg_23380 | gi|126640115|ref|NC_009085.1| | 3424056 | 3424080 | 25 |
| exprReg_23381 | gi|126640115|ref|NC_009085.1| | 3424110 | 3424129 | 20 |
| exprReg_23382 | gi|126640115|ref|NC_009085.1| | 3424134 | 3424156 | 23 |
| exprReg_23383 | gi|126640115|ref|NC_009085.1| | 3424239 | 3424293 | 55 |
| exprReg_23384 | gi|126640115|ref|NC_009085.1| | 3424404 | 3424466 | 63 |
| exprReg_23385 | gi|126640115|ref|NC_009085.1| | 3424507 | 3424569 | 63 |
| exprReg_23386 | gi|126640115|ref|NC_009085.1| | 3424736 | 3424793 | 58 |
| exprReg_23387 | gi|126640115|ref|NC_009085.1| | 3424802 | 3424900 | 99 |
| exprReg_23388 | gi|126640115|ref|NC_009085.1| | 3424953 | 3424969 | 17 |
| exprReg_23389 | gi|126640115|ref|NC_009085.1| | 3424987 | 3425030 | 44 |
| exprReg_23390 | gi|126640115|ref|NC_009085.1| | 3425059 | 3425086 | 28 |
| exprReg_23391 | gi|126640115|ref|NC_009085.1| | 3425091 | 3425132 | 42 |
| exprReg_23392 | gi|126640115|ref|NC_009085.1| | 3425209 | 3425239 | 31 |
| exprReg_23393 | gi|126640115|ref|NC_009085.1| | 3425370 | 3425536 | 167 |
| exprReg_23394 | gi|126640115|ref|NC_009085.1| | 3425553 | 3425573 | 21 |
| exprReg_23395 | gi|126640115|ref|NC_009085.1| | 3425692 | 3425708 | 17 |
| exprReg_23396 | gi|126640115|ref|NC_009085.1| | 3425842 | 3425860 | 19 |
| exprReg_23397 | gi|126640115|ref|NC_009085.1| | 3425913 | 3425937 | 25 |
| exprReg_23398 | gi|126640115|ref|NC_009085.1| | 3425948 | 3426033 | 86 |
| exprReg_23399 | gi|126640115|ref|NC_009085.1| | 3426218 | 3426283 | 66 |
| exprReg_23400 | gi|126640115|ref|NC_009085.1| | 3426304 | 3426325 | 22 |
| exprReg_23401 | gi|126640115|ref|NC_009085.1| | 3426402 | 3426421 | 20 |
| exprReg_23402 | gi|126640115|ref|NC_009085.1| | 3426456 | 3426565 | 110 |
| exprReg_23403 | gi|126640115|ref|NC_009085.1| | 3426584 | 3426614 | 31 |
| exprReg_23404 | gi|126640115|ref|NC_009085.1| | 3426717 | 3426762 | 46 |
| exprReg_23405 | gi|126640115|ref|NC_009085.1| | 3426792 | 3426870 | 79 |
| exprReg_23406 | gi|126640115|ref|NC_009085.1| | 3427101 | 3427122 | 22 |
| exprReg_23407 | gi|126640115|ref|NC_009085.1| | 3427255 | 3427290 | 36 |
| exprReg_23408 | gi|126640115|ref|NC_009085.1| | 3427381 | 3427399 | 19 |
| exprReg_23415 | gi|126640115|ref|NC_009085.1| | 3428708 | 3428803 | 96 |
| exprReg_23416 | gi|126640115|ref|NC_009085.1| | 3428982 | 3429026 | 45 |
| exprReg_23417 | gi|126640115|ref|NC_009085.1| | 3429086 | 3429168 | 83 |
| exprReg_23418 | gi|126640115|ref|NC_009085.1| | 3429405 | 3429451 | 47 |
| exprReg_23419 | gi|126640115|ref|NC_009085.1| | 3429642 | 3429714 | 73 |
| exprReg_23420 | gi|126640115|ref|NC_009085.1| | 3429776 | 3429800 | 25 |
| exprReg_23421 | gi|126640115|ref|NC_009085.1| | 3429888 | 3429921 | 34 |
| exprReg_23435 | gi|126640115|ref|NC_009085.1| | 3432143 | 3432161 | 19 |
| exprReg_23436 | gi|126640115|ref|NC_009085.1| | 3432175 | 3432215 | 41 |
| exprReg_23437 | gi|126640115|ref|NC_009085.1| | 3432243 | 3432351 | 109 |
| exprReg_23438 | gi|126640115|ref|NC_009085.1| | 3432395 | 3432459 | 65 |
| exprReg_23442 | gi|126640115|ref|NC_009085.1| | 3432799 | 3432815 | 17 |
| exprReg_23443 | gi|126640115|ref|NC_009085.1| | 3432924 | 3433166 | 243 |
| exprReg_23444 | gi|126640115|ref|NC_009085.1| | 3433168 | 3433241 | 74 |
| exprReg_23445 | gi|126640115|ref|NC_009085.1| | 3433283 | 3433299 | 17 |
| exprReg_23446 | gi|126640115|ref|NC_009085.1| | 3433328 | 3433442 | 115 |
| exprReg_23447 | gi|126640115|ref|NC_009085.1| | 3433510 | 3433560 | 51 |
| exprReg_23448 | gi|126640115|ref|NC_009085.1| | 3433576 | 3433670 | 95 |
| exprReg_23449 | gi|126640115|ref|NC_009085.1| | 3433680 | 3433699 | 20 |
| exprReg_23450 | gi|126640115|ref|NC_009085.1| | 3433729 | 3433826 | 98 |
| exprReg_23451 | gi|126640115|ref|NC_009085.1| | 3433830 | 3433943 | 114 |
| exprReg_23452 | gi|126640115|ref|NC_009085.1| | 3433966 | 3434085 | 120 |
| exprReg_23453 | gi|126640115|ref|NC_009085.1| | 3434119 | 3434160 | 42 |
| exprReg_23454 | gi|126640115|ref|NC_009085.1| | 3434207 | 3434259 | 53 |
| exprReg_23455 | gi|126640115|ref|NC_009085.1| | 3434357 | 3434377 | 21 |
| exprReg_23464 | gi|126640115|ref|NC_009085.1| | 3435799 | 3435831 | 33 |
| exprReg_23466 | gi|126640115|ref|NC_009085.1| | 3441603 | 3441628 | 26 |
| exprReg_23471 | gi|126640115|ref|NC_009085.1| | 3442166 | 3442214 | 49 |
| exprReg_23472 | gi|126640115|ref|NC_009085.1| | 3442273 | 3442290 | 18 |
| exprReg_23482 | gi|126640115|ref|NC_009085.1| | 3443362 | 3443407 | 46 |
| exprReg_23483 | gi|126640115|ref|NC_009085.1| | 3443551 | 3443591 | 41 |
| exprReg_23484 | gi|126640115|ref|NC_009085.1| | 3443669 | 3443719 | 51 |
| exprReg_23485 | gi|126640115|ref|NC_009085.1| | 3443739 | 3444010 | 272 |
| exprReg_23486 | gi|126640115|ref|NC_009085.1| | 3444077 | 3444241 | 165 |
| exprReg_23487 | gi|126640115|ref|NC_009085.1| | 3444268 | 3444371 | 104 |
| exprReg_23488 | gi|126640115|ref|NC_009085.1| | 3444382 | 3444430 | 49 |
| exprReg_23489 | gi|126640115|ref|NC_009085.1| | 3444571 | 3444694 | 124 |
| exprReg_23490 | gi|126640115|ref|NC_009085.1| | 3444704 | 3444750 | 47 |
| exprReg_23507 | gi|126640115|ref|NC_009085.1| | 3447373 | 3447399 | 27 |
| exprReg_23508 | gi|126640115|ref|NC_009085.1| | 3447401 | 3447465 | 65 |
| exprReg_23515 | gi|126640115|ref|NC_009085.1| | 3449583 | 3449645 | 63 |
| exprReg_23516 | gi|126640115|ref|NC_009085.1| | 3449828 | 3449919 | 92 |
| exprReg_23517 | gi|126640115|ref|NC_009085.1| | 3450010 | 3450026 | 17 |
| exprReg_23518 | gi|126640115|ref|NC_009085.1| | 3450098 | 3450116 | 19 |
| exprReg_23519 | gi|126640115|ref|NC_009085.1| | 3450261 | 3450463 | 203 |
| exprReg_23520 | gi|126640115|ref|NC_009085.1| | 3450470 | 3450556 | 87 |
| exprReg_23521 | gi|126640115|ref|NC_009085.1| | 3450915 | 3450967 | 53 |
| exprReg_23522 | gi|126640115|ref|NC_009085.1| | 3451048 | 3451069 | 22 |
| exprReg_23523 | gi|126640115|ref|NC_009085.1| | 3451151 | 3451168 | 18 |
| exprReg_23524 | gi|126640115|ref|NC_009085.1| | 3451222 | 3451243 | 22 |
| exprReg_23525 | gi|126640115|ref|NC_009085.1| | 3451262 | 3451278 | 17 |
| exprReg_23526 | gi|126640115|ref|NC_009085.1| | 3451764 | 3451784 | 21 |
| exprReg_23530 | gi|126640115|ref|NC_009085.1| | 3452305 | 3452325 | 21 |
| exprReg_23537 | gi|126640115|ref|NC_009085.1| | 3453400 | 3453455 | 56 |
| exprReg_23538 | gi|126640115|ref|NC_009085.1| | 3453470 | 3453655 | 186 |
| exprReg_23539 | gi|126640115|ref|NC_009085.1| | 3453734 | 3453790 | 57 |
| exprReg_23540 | gi|126640115|ref|NC_009085.1| | 3453999 | 3454022 | 24 |
| exprReg_23541 | gi|126640115|ref|NC_009085.1| | 3454099 | 3454120 | 22 |
| exprReg_23542 | gi|126640115|ref|NC_009085.1| | 3454154 | 3454205 | 52 |
| exprReg_23557 | gi|126640115|ref|NC_009085.1| | 3456466 | 3456576 | 111 |
| exprReg_23558 | gi|126640115|ref|NC_009085.1| | 3456617 | 3456653 | 37 |
| exprReg_23559 | gi|126640115|ref|NC_009085.1| | 3456705 | 3456723 | 19 |
| exprReg_23560 | gi|126640115|ref|NC_009085.1| | 3456725 | 3456780 | 56 |
| exprReg_23571 | gi|126640115|ref|NC_009085.1| | 3458158 | 3458345 | 188 |
| exprReg_23572 | gi|126640115|ref|NC_009085.1| | 3458356 | 3458632 | 277 |
| exprReg_23573 | gi|126640115|ref|NC_009085.1| | 3458672 | 3458702 | 31 |
| exprReg_23584 | gi|126640115|ref|NC_009085.1| | 3460214 | 3460462 | 249 |
| exprReg_23592 | gi|126640115|ref|NC_009085.1| | 3461446 | 3461468 | 23 |
| exprReg_23593 | gi|126640115|ref|NC_009085.1| | 3461530 | 3461634 | 105 |
| exprReg_23594 | gi|126640115|ref|NC_009085.1| | 3461692 | 3461712 | 21 |
| exprReg_23595 | gi|126640115|ref|NC_009085.1| | 3461720 | 3461821 | 102 |
| exprReg_23596 | gi|126640115|ref|NC_009085.1| | 3461862 | 3461901 | 40 |
| exprReg_23597 | gi|126640115|ref|NC_009085.1| | 3461904 | 3462069 | 166 |
| exprReg_23603 | gi|126640115|ref|NC_009085.1| | 3463203 | 3463249 | 47 |
| exprReg_23604 | gi|126640115|ref|NC_009085.1| | 3463411 | 3463589 | 179 |
| exprReg_23611 | gi|126640115|ref|NC_009085.1| | 3464652 | 3464716 | 65 |
| exprReg_23612 | gi|126640115|ref|NC_009085.1| | 3464725 | 3464748 | 24 |
| exprReg_23619 | gi|126640115|ref|NC_009085.1| | 3465943 | 3465963 | 21 |
| exprReg_23620 | gi|126640115|ref|NC_009085.1| | 3465987 | 3466003 | 17 |
| exprReg_23624 | gi|126640115|ref|NC_009085.1| | 3466745 | 3466762 | 18 |
| exprReg_23625 | gi|126640115|ref|NC_009085.1| | 3466768 | 3466824 | 57 |
| exprReg_23635 | gi|126640115|ref|NC_009085.1| | 3467830 | 3467862 | 33 |
| exprReg_23636 | gi|126640115|ref|NC_009085.1| | 3467865 | 3467881 | 17 |
| exprReg_23667 | gi|126640115|ref|NC_009085.1| | 3473792 | 3473942 | 151 |
| exprReg_23672 | gi|126640115|ref|NC_009085.1| | 3474609 | 3474636 | 28 |
| exprReg_23679 | gi|126640115|ref|NC_009085.1| | 3475793 | 3475817 | 25 |
| exprReg_23680 | gi|126640115|ref|NC_009085.1| | 3475840 | 3475885 | 46 |
| exprReg_23689 | gi|126640115|ref|NC_009085.1| | 3477249 | 3477348 | 100 |
| exprReg_23690 | gi|126640115|ref|NC_009085.1| | 3477352 | 3477370 | 19 |
| exprReg_23691 | gi|126640115|ref|NC_009085.1| | 3477373 | 3477427 | 55 |
| exprReg_23692 | gi|126640115|ref|NC_009085.1| | 3477535 | 3477556 | 22 |
| exprReg_23693 | gi|126640115|ref|NC_009085.1| | 3477570 | 3477600 | 31 |
| exprReg_23694 | gi|126640115|ref|NC_009085.1| | 3477760 | 3477805 | 46 |
| exprReg_23707 | gi|126640115|ref|NC_009085.1| | 3479835 | 3479905 | 71 |
| exprReg_23708 | gi|126640115|ref|NC_009085.1| | 3480009 | 3480027 | 19 |
| exprReg_23724 | gi|126640115|ref|NC_009085.1| | 3482543 | 3482595 | 53 |
| exprReg_23739 | gi|126640115|ref|NC_009085.1| | 3484632 | 3484747 | 116 |
| exprReg_23745 | gi|126640115|ref|NC_009085.1| | 3485687 | 3485736 | 50 |
| exprReg_23746 | gi|126640115|ref|NC_009085.1| | 3485777 | 3485801 | 25 |
| exprReg_23753 | gi|126640115|ref|NC_009085.1| | 3486879 | 3486984 | 106 |
| exprReg_23760 | gi|126640115|ref|NC_009085.1| | 3488022 | 3488042 | 21 |
| exprReg_23766 | gi|126640115|ref|NC_009085.1| | 3488672 | 3488692 | 21 |
| exprReg_23800 | gi|126640115|ref|NC_009085.1| | 3494212 | 3494235 | 24 |
| exprReg_23803 | gi|126640115|ref|NC_009085.1| | 3495179 | 3495203 | 25 |
| exprReg_23814 | gi|126640115|ref|NC_009085.1| | 3497038 | 3497122 | 85 |
| exprReg_23815 | gi|126640115|ref|NC_009085.1| | 3497244 | 3497317 | 74 |
| exprReg_23828 | gi|126640115|ref|NC_009085.1| | 3499130 | 3499154 | 25 |
| exprReg_23850 | gi|126640115|ref|NC_009085.1| | 3508767 | 3508959 | 193 |
| exprReg_23851 | gi|126640115|ref|NC_009085.1| | 3508968 | 3509077 | 110 |
| exprReg_23856 | gi|126640115|ref|NC_009085.1| | 3509698 | 3510126 | 429 |
| exprReg_23857 | gi|126640115|ref|NC_009085.1| | 3510241 | 3510257 | 17 |
| exprReg_23861 | gi|126640115|ref|NC_009085.1| | 3510655 | 3510709 | 55 |
| exprReg_23867 | gi|126640115|ref|NC_009085.1| | 3511658 | 3511772 | 115 |
| exprReg_23868 | gi|126640115|ref|NC_009085.1| | 3511853 | 3511873 | 21 |
| exprReg_23869 | gi|126640115|ref|NC_009085.1| | 3511877 | 3511907 | 31 |
| exprReg_23886 | gi|126640115|ref|NC_009085.1| | 3514565 | 3514645 | 81 |
| exprReg_23887 | gi|126640115|ref|NC_009085.1| | 3514685 | 3514708 | 24 |
| exprReg_23891 | gi|126640115|ref|NC_009085.1| | 3515507 | 3515558 | 52 |
| exprReg_23907 | gi|126640115|ref|NC_009085.1| | 3517529 | 3517563 | 35 |
| exprReg_23910 | gi|126640115|ref|NC_009085.1| | 3517929 | 3517969 | 41 |
| exprReg_23917 | gi|126640115|ref|NC_009085.1| | 3519132 | 3519210 | 79 |
| exprReg_23925 | gi|126640115|ref|NC_009085.1| | 3519809 | 3519826 | 18 |
| exprReg_23926 | gi|126640115|ref|NC_009085.1| | 3519879 | 3519895 | 17 |
| exprReg_23939 | gi|126640115|ref|NC_009085.1| | 3522061 | 3522102 | 42 |
| exprReg_23940 | gi|126640115|ref|NC_009085.1| | 3522104 | 3522223 | 120 |
| exprReg_23941 | gi|126640115|ref|NC_009085.1| | 3522247 | 3522373 | 127 |
| exprReg_23957 | gi|126640115|ref|NC_009085.1| | 3524406 | 3524495 | 90 |
| exprReg_23958 | gi|126640115|ref|NC_009085.1| | 3524505 | 3524552 | 48 |
| exprReg_23974 | gi|126640115|ref|NC_009085.1| | 3526574 | 3526616 | 43 |
| exprReg_23975 | gi|126640115|ref|NC_009085.1| | 3526641 | 3526658 | 18 |
| exprReg_23980 | gi|126640115|ref|NC_009085.1| | 3528164 | 3528404 | 241 |
| exprReg_23997 | gi|126640115|ref|NC_009085.1| | 3531195 | 3531263 | 69 |
| exprReg_24000 | gi|126640115|ref|NC_009085.1| | 3531761 | 3531827 | 67 |
| exprReg_24001 | gi|126640115|ref|NC_009085.1| | 3531861 | 3532087 | 227 |
| exprReg_24002 | gi|126640115|ref|NC_009085.1| | 3532130 | 3532147 | 18 |
| exprReg_24024 | gi|126640115|ref|NC_009085.1| | 3534964 | 3534982 | 19 |
| exprReg_24025 | gi|126640115|ref|NC_009085.1| | 3534994 | 3535029 | 36 |
| exprReg_24026 | gi|126640115|ref|NC_009085.1| | 3535043 | 3535069 | 27 |
| exprReg_24027 | gi|126640115|ref|NC_009085.1| | 3535135 | 3535224 | 90 |
| exprReg_24028 | gi|126640115|ref|NC_009085.1| | 3535234 | 3535306 | 73 |
| exprReg_24035 | gi|126640115|ref|NC_009085.1| | 3536948 | 3536999 | 52 |
| exprReg_24036 | gi|126640115|ref|NC_009085.1| | 3537031 | 3537051 | 21 |
| exprReg_24037 | gi|126640115|ref|NC_009085.1| | 3537094 | 3537133 | 40 |
| exprReg_24038 | gi|126640115|ref|NC_009085.1| | 3537163 | 3537257 | 95 |
| exprReg_24054 | gi|126640115|ref|NC_009085.1| | 3539533 | 3539573 | 41 |
| exprReg_24055 | gi|126640115|ref|NC_009085.1| | 3539682 | 3539702 | 21 |
| exprReg_24056 | gi|126640115|ref|NC_009085.1| | 3539714 | 3539730 | 17 |
| exprReg_24061 | gi|126640115|ref|NC_009085.1| | 3540379 | 3540446 | 68 |
| exprReg_24069 | gi|126640115|ref|NC_009085.1| | 3541482 | 3541572 | 91 |
| exprReg_24070 | gi|126640115|ref|NC_009085.1| | 3541708 | 3541778 | 71 |
| exprReg_24071 | gi|126640115|ref|NC_009085.1| | 3541850 | 3541923 | 74 |
| exprReg_24072 | gi|126640115|ref|NC_009085.1| | 3541929 | 3541956 | 28 |
| exprReg_24073 | gi|126640115|ref|NC_009085.1| | 3542013 | 3542157 | 145 |
| exprReg_24074 | gi|126640115|ref|NC_009085.1| | 3542450 | 3542519 | 70 |
| exprReg_24075 | gi|126640115|ref|NC_009085.1| | 3542616 | 3542684 | 69 |
| exprReg_24094 | gi|126640115|ref|NC_009085.1| | 3545717 | 3545796 | 80 |
| exprReg_24095 | gi|126640115|ref|NC_009085.1| | 3545811 | 3545831 | 21 |
| exprReg_24153 | gi|126640115|ref|NC_009085.1| | 3560689 | 3560729 | 41 |
| exprReg_24178 | gi|126640115|ref|NC_009085.1| | 3563649 | 3563690 | 42 |
| exprReg_24179 | gi|126640115|ref|NC_009085.1| | 3563696 | 3563718 | 23 |
| exprReg_24180 | gi|126640115|ref|NC_009085.1| | 3563726 | 3563747 | 22 |
| exprReg_24192 | gi|126640115|ref|NC_009085.1| | 3565015 | 3565122 | 108 |
| exprReg_24198 | gi|126640115|ref|NC_009085.1| | 3565887 | 3565986 | 100 |
| exprReg_24199 | gi|126640115|ref|NC_009085.1| | 3566001 | 3566018 | 18 |
| exprReg_24200 | gi|126640115|ref|NC_009085.1| | 3566039 | 3566070 | 32 |
| exprReg_24201 | gi|126640115|ref|NC_009085.1| | 3566118 | 3566183 | 66 |
| exprReg_24222 | gi|126640115|ref|NC_009085.1| | 3569223 | 3569336 | 114 |
| exprReg_24223 | gi|126640115|ref|NC_009085.1| | 3569365 | 3569399 | 35 |
| exprReg_24224 | gi|126640115|ref|NC_009085.1| | 3569412 | 3569484 | 73 |
| exprReg_24225 | gi|126640115|ref|NC_009085.1| | 3569486 | 3569502 | 17 |
| exprReg_24226 | gi|126640115|ref|NC_009085.1| | 3569515 | 3569566 | 52 |
| exprReg_24253 | gi|126640115|ref|NC_009085.1| | 3572837 | 3572916 | 80 |
| exprReg_24254 | gi|126640115|ref|NC_009085.1| | 3572930 | 3573072 | 143 |
| exprReg_24262 | gi|126640115|ref|NC_009085.1| | 3573814 | 3573910 | 97 |
| exprReg_24278 | gi|126640115|ref|NC_009085.1| | 3576058 | 3576094 | 37 |
| exprReg_24283 | gi|126640115|ref|NC_009085.1| | 3576804 | 3576858 | 55 |
| exprReg_24284 | gi|126640115|ref|NC_009085.1| | 3576871 | 3576927 | 57 |
| exprReg_24285 | gi|126640115|ref|NC_009085.1| | 3576949 | 3577003 | 55 |
| exprReg_24286 | gi|126640115|ref|NC_009085.1| | 3577051 | 3577071 | 21 |
| exprReg_24290 | gi|126640115|ref|NC_009085.1| | 3577668 | 3577732 | 65 |
| exprReg_24291 | gi|126640115|ref|NC_009085.1| | 3577744 | 3577761 | 18 |
| exprReg_24300 | gi|126640115|ref|NC_009085.1| | 3578824 | 3578860 | 37 |
| exprReg_24303 | gi|126640115|ref|NC_009085.1| | 3579234 | 3579252 | 19 |
| exprReg_24304 | gi|126640115|ref|NC_009085.1| | 3579254 | 3579277 | 24 |
| exprReg_24308 | gi|126640115|ref|NC_009085.1| | 3579830 | 3579850 | 21 |
| exprReg_24319 | gi|126640115|ref|NC_009085.1| | 3582071 | 3582152 | 82 |
| exprReg_24320 | gi|126640115|ref|NC_009085.1| | 3582156 | 3582197 | 42 |
| exprReg_24321 | gi|126640115|ref|NC_009085.1| | 3582264 | 3582481 | 218 |
| exprReg_24333 | gi|126640115|ref|NC_009085.1| | 3584452 | 3584469 | 18 |
| exprReg_24334 | gi|126640115|ref|NC_009085.1| | 3584474 | 3584668 | 195 |
| exprReg_24342 | gi|126640115|ref|NC_009085.1| | 3585697 | 3585732 | 36 |
| exprReg_24350 | gi|126640115|ref|NC_009085.1| | 3587616 | 3587664 | 49 |
| exprReg_24351 | gi|126640115|ref|NC_009085.1| | 3587681 | 3587729 | 49 |
| exprReg_24352 | gi|126640115|ref|NC_009085.1| | 3587768 | 3587890 | 123 |
| exprReg_24353 | gi|126640115|ref|NC_009085.1| | 3587909 | 3587930 | 22 |
| exprReg_24354 | gi|126640115|ref|NC_009085.1| | 3587972 | 3588012 | 41 |
| exprReg_24363 | gi|126640115|ref|NC_009085.1| | 3589654 | 3589704 | 51 |
| exprReg_24364 | gi|126640115|ref|NC_009085.1| | 3589771 | 3589800 | 30 |
| exprReg_24383 | gi|126640115|ref|NC_009085.1| | 3593043 | 3593068 | 26 |
| exprReg_24384 | gi|126640115|ref|NC_009085.1| | 3593108 | 3593135 | 28 |
| exprReg_24385 | gi|126640115|ref|NC_009085.1| | 3593181 | 3593258 | 78 |
| exprReg_24409 | gi|126640115|ref|NC_009085.1| | 3595743 | 3595865 | 123 |
| exprReg_24410 | gi|126640115|ref|NC_009085.1| | 3595917 | 3595944 | 28 |
| exprReg_24417 | gi|126640115|ref|NC_009085.1| | 3597457 | 3597473 | 17 |
| exprReg_24418 | gi|126640115|ref|NC_009085.1| | 3597503 | 3597549 | 47 |
| exprReg_24429 | gi|126640115|ref|NC_009085.1| | 3599384 | 3599435 | 52 |
| exprReg_24430 | gi|126640115|ref|NC_009085.1| | 3599510 | 3599661 | 152 |
| exprReg_24431 | gi|126640115|ref|NC_009085.1| | 3599752 | 3599803 | 52 |
| exprReg_24439 | gi|126640115|ref|NC_009085.1| | 3601112 | 3601128 | 17 |
| exprReg_24440 | gi|126640115|ref|NC_009085.1| | 3601169 | 3601288 | 120 |
| exprReg_24441 | gi|126640115|ref|NC_009085.1| | 3601360 | 3601380 | 21 |
| exprReg_24442 | gi|126640115|ref|NC_009085.1| | 3601433 | 3601454 | 22 |
| exprReg_24452 | gi|126640115|ref|NC_009085.1| | 3602426 | 3602497 | 72 |
| exprReg_24453 | gi|126640115|ref|NC_009085.1| | 3602522 | 3602596 | 75 |
| exprReg_24468 | gi|126640115|ref|NC_009085.1| | 3604560 | 3604667 | 108 |
| exprReg_24469 | gi|126640115|ref|NC_009085.1| | 3604683 | 3604700 | 18 |
| exprReg_24470 | gi|126640115|ref|NC_009085.1| | 3604720 | 3604764 | 45 |
| exprReg_24471 | gi|126640115|ref|NC_009085.1| | 3604815 | 3604842 | 28 |
| exprReg_24472 | gi|126640115|ref|NC_009085.1| | 3604852 | 3604882 | 31 |
| exprReg_24473 | gi|126640115|ref|NC_009085.1| | 3604911 | 3604931 | 21 |
| exprReg_24474 | gi|126640115|ref|NC_009085.1| | 3605248 | 3605296 | 49 |
| exprReg_24475 | gi|126640115|ref|NC_009085.1| | 3605335 | 3605360 | 26 |
| exprReg_24476 | gi|126640115|ref|NC_009085.1| | 3605492 | 3605510 | 19 |
| exprReg_24478 | gi|126640115|ref|NC_009085.1| | 3605739 | 3605790 | 52 |
| exprReg_24479 | gi|126640115|ref|NC_009085.1| | 3605871 | 3605928 | 58 |
| exprReg_24487 | gi|126640115|ref|NC_009085.1| | 3607816 | 3607841 | 26 |
| exprReg_24488 | gi|126640115|ref|NC_009085.1| | 3607920 | 3607954 | 35 |
| exprReg_24495 | gi|126640115|ref|NC_009085.1| | 3609044 | 3609060 | 17 |
| exprReg_24511 | gi|126640115|ref|NC_009085.1| | 3611580 | 3611616 | 37 |
| exprReg_24512 | gi|126640115|ref|NC_009085.1| | 3611690 | 3611707 | 18 |
| exprReg_24513 | gi|126640115|ref|NC_009085.1| | 3611770 | 3611847 | 78 |
| exprReg_24514 | gi|126640115|ref|NC_009085.1| | 3611972 | 3612013 | 42 |
| exprReg_24515 | gi|126640115|ref|NC_009085.1| | 3612172 | 3612190 | 19 |
| exprReg_24525 | gi|126640115|ref|NC_009085.1| | 3613188 | 3613209 | 22 |
| exprReg_24533 | gi|126640115|ref|NC_009085.1| | 3613966 | 3614175 | 210 |
| exprReg_24559 | gi|126640115|ref|NC_009085.1| | 3616871 | 3617001 | 131 |
| exprReg_24584 | gi|126640115|ref|NC_009085.1| | 3620646 | 3620725 | 80 |
| exprReg_24585 | gi|126640115|ref|NC_009085.1| | 3620740 | 3620874 | 135 |
| exprReg_24586 | gi|126640115|ref|NC_009085.1| | 3620964 | 3621068 | 105 |
| exprReg_24587 | gi|126640115|ref|NC_009085.1| | 3621081 | 3621220 | 140 |
| exprReg_24588 | gi|126640115|ref|NC_009085.1| | 3621707 | 3621758 | 52 |
| exprReg_24600 | gi|126640115|ref|NC_009085.1| | 3623405 | 3623445 | 41 |
| exprReg_24611 | gi|126640115|ref|NC_009085.1| | 3624930 | 3624949 | 20 |
| exprReg_24612 | gi|126640115|ref|NC_009085.1| | 3624951 | 3624972 | 22 |
| exprReg_24613 | gi|126640115|ref|NC_009085.1| | 3624980 | 3625225 | 246 |
| exprReg_24626 | gi|126640115|ref|NC_009085.1| | 3626945 | 3626985 | 41 |
| exprReg_24645 | gi|126640115|ref|NC_009085.1| | 3629675 | 3629695 | 21 |
| exprReg_24646 | gi|126640115|ref|NC_009085.1| | 3629706 | 3629742 | 37 |
| exprReg_24651 | gi|126640115|ref|NC_009085.1| | 3630335 | 3630352 | 18 |
| exprReg_24652 | gi|126640115|ref|NC_009085.1| | 3630467 | 3630488 | 22 |
| exprReg_24663 | gi|126640115|ref|NC_009085.1| | 3631619 | 3631705 | 87 |
| exprReg_24674 | gi|126640115|ref|NC_009085.1| | 3633983 | 3634007 | 25 |
| exprReg_24679 | gi|126640115|ref|NC_009085.1| | 3634673 | 3634689 | 17 |
| exprReg_24680 | gi|126640115|ref|NC_009085.1| | 3634928 | 3634949 | 22 |
| exprReg_24700 | gi|126640115|ref|NC_009085.1| | 3637557 | 3637575 | 19 |
| exprReg_24701 | gi|126640115|ref|NC_009085.1| | 3637658 | 3637746 | 89 |
| exprReg_24702 | gi|126640115|ref|NC_009085.1| | 3637760 | 3637813 | 54 |
| exprReg_24703 | gi|126640115|ref|NC_009085.1| | 3637828 | 3637870 | 43 |
| exprReg_24704 | gi|126640115|ref|NC_009085.1| | 3637972 | 3638096 | 125 |
| exprReg_24705 | gi|126640115|ref|NC_009085.1| | 3638154 | 3638303 | 150 |
| exprReg_24715 | gi|126640115|ref|NC_009085.1| | 3639409 | 3639499 | 91 |
| exprReg_24733 | gi|126640115|ref|NC_009085.1| | 3641757 | 3641806 | 50 |
| exprReg_24734 | gi|126640115|ref|NC_009085.1| | 3641827 | 3641871 | 45 |
| exprReg_24750 | gi|126640115|ref|NC_009085.1| | 3643584 | 3643682 | 99 |
| exprReg_24767 | gi|126640115|ref|NC_009085.1| | 3646294 | 3646465 | 172 |
| exprReg_24771 | gi|126640115|ref|NC_009085.1| | 3647174 | 3647225 | 52 |
| exprReg_24819 | gi|126640115|ref|NC_009085.1| | 3652968 | 3653045 | 78 |
| exprReg_24820 | gi|126640115|ref|NC_009085.1| | 3653224 | 3653268 | 45 |
| exprReg_24821 | gi|126640115|ref|NC_009085.1| | 3653276 | 3653319 | 44 |
| exprReg_24822 | gi|126640115|ref|NC_009085.1| | 3653326 | 3653819 | 494 |
| exprReg_24828 | gi|126640115|ref|NC_009085.1| | 3654699 | 3654958 | 260 |
| exprReg_24829 | gi|126640115|ref|NC_009085.1| | 3654995 | 3655131 | 137 |
| exprReg_24830 | gi|126640115|ref|NC_009085.1| | 3655135 | 3655197 | 63 |
| exprReg_24831 | gi|126640115|ref|NC_009085.1| | 3655358 | 3655417 | 60 |
| exprReg_24832 | gi|126640115|ref|NC_009085.1| | 3655470 | 3655530 | 61 |
| exprReg_24833 | gi|126640115|ref|NC_009085.1| | 3655763 | 3655815 | 53 |
| exprReg_24834 | gi|126640115|ref|NC_009085.1| | 3655818 | 3655876 | 59 |
| exprReg_24839 | gi|126640115|ref|NC_009085.1| | 3656883 | 3656924 | 42 |
| exprReg_24840 | gi|126640115|ref|NC_009085.1| | 3656926 | 3656942 | 17 |
| exprReg_24841 | gi|126640115|ref|NC_009085.1| | 3656944 | 3656997 | 54 |
| exprReg_24846 | gi|126640115|ref|NC_009085.1| | 3658125 | 3658151 | 27 |
| exprReg_24847 | gi|126640115|ref|NC_009085.1| | 3658227 | 3658409 | 183 |
| exprReg_24887 | gi|126640115|ref|NC_009085.1| | 3663641 | 3663668 | 28 |
| exprReg_24888 | gi|126640115|ref|NC_009085.1| | 3663670 | 3663787 | 118 |
| exprReg_24893 | gi|126640115|ref|NC_009085.1| | 3664480 | 3664499 | 20 |
| exprReg_24914 | gi|126640115|ref|NC_009085.1| | 3667827 | 3667868 | 42 |
| exprReg_24947 | gi|126640115|ref|NC_009085.1| | 3674069 | 3674085 | 17 |
| exprReg_24948 | gi|126640115|ref|NC_009085.1| | 3674089 | 3674312 | 224 |
| exprReg_24984 | gi|126640115|ref|NC_009085.1| | 3679224 | 3679243 | 20 |
| exprReg_24985 | gi|126640115|ref|NC_009085.1| | 3679278 | 3679297 | 20 |
| exprReg_25004 | gi|126640115|ref|NC_009085.1| | 3682245 | 3682529 | 285 |
| exprReg_25024 | gi|126640115|ref|NC_009085.1| | 3686690 | 3686745 | 56 |
| exprReg_25025 | gi|126640115|ref|NC_009085.1| | 3686757 | 3686793 | 37 |
| exprReg_25040 | gi|126640115|ref|NC_009085.1| | 3688598 | 3688729 | 132 |
| exprReg_25041 | gi|126640115|ref|NC_009085.1| | 3688741 | 3688758 | 18 |
| exprReg_25055 | gi|126640115|ref|NC_009085.1| | 3690197 | 3690300 | 104 |
| exprReg_25077 | gi|126640115|ref|NC_009085.1| | 3693175 | 3693441 | 267 |
| exprReg_25084 | gi|126640115|ref|NC_009085.1| | 3694526 | 3694607 | 82 |
| exprReg_25085 | gi|126640115|ref|NC_009085.1| | 3694832 | 3694973 | 142 |
| exprReg_25086 | gi|126640115|ref|NC_009085.1| | 3694989 | 3695018 | 30 |
| exprReg_25106 | gi|126640115|ref|NC_009085.1| | 3697201 | 3697269 | 69 |
| exprReg_25116 | gi|126640115|ref|NC_009085.1| | 3698740 | 3698777 | 38 |
| exprReg_25123 | gi|126640115|ref|NC_009085.1| | 3700137 | 3700155 | 19 |
| exprReg_25124 | gi|126640115|ref|NC_009085.1| | 3700173 | 3700203 | 31 |
| exprReg_25125 | gi|126640115|ref|NC_009085.1| | 3700225 | 3700376 | 152 |
| exprReg_25126 | gi|126640115|ref|NC_009085.1| | 3700398 | 3700547 | 150 |
| exprReg_25127 | gi|126640115|ref|NC_009085.1| | 3700579 | 3700965 | 387 |
| exprReg_25128 | gi|126640115|ref|NC_009085.1| | 3700976 | 3701065 | 90 |
| exprReg_25129 | gi|126640115|ref|NC_009085.1| | 3701069 | 3701096 | 28 |
| exprReg_25130 | gi|126640115|ref|NC_009085.1| | 3701125 | 3701197 | 73 |
| exprReg_25131 | gi|126640115|ref|NC_009085.1| | 3701359 | 3701380 | 22 |
| exprReg_25140 | gi|126640115|ref|NC_009085.1| | 3702532 | 3702604 | 73 |
| exprReg_25141 | gi|126640115|ref|NC_009085.1| | 3702614 | 3702646 | 33 |
| exprReg_25142 | gi|126640115|ref|NC_009085.1| | 3702659 | 3702676 | 18 |
| exprReg_25178 | gi|126640115|ref|NC_009085.1| | 3707050 | 3707148 | 99 |
| exprReg_25192 | gi|126640115|ref|NC_009085.1| | 3709103 | 3709142 | 40 |
| exprReg_25193 | gi|126640115|ref|NC_009085.1| | 3709191 | 3709239 | 49 |
| exprReg_25194 | gi|126640115|ref|NC_009085.1| | 3709290 | 3709347 | 58 |
| exprReg_25195 | gi|126640115|ref|NC_009085.1| | 3709495 | 3709513 | 19 |
| exprReg_25196 | gi|126640115|ref|NC_009085.1| | 3709560 | 3709743 | 184 |
| exprReg_25197 | gi|126640115|ref|NC_009085.1| | 3709814 | 3709830 | 17 |
| exprReg_25198 | gi|126640115|ref|NC_009085.1| | 3710086 | 3710106 | 21 |
| exprReg_25199 | gi|126640115|ref|NC_009085.1| | 3710192 | 3710482 | 291 |
| exprReg_25200 | gi|126640115|ref|NC_009085.1| | 3710521 | 3710616 | 96 |
| exprReg_25216 | gi|126640115|ref|NC_009085.1| | 3712346 | 3712435 | 90 |
| exprReg_25217 | gi|126640115|ref|NC_009085.1| | 3712670 | 3712738 | 69 |
| exprReg_25235 | gi|126640115|ref|NC_009085.1| | 3715245 | 3715272 | 28 |
| exprReg_25236 | gi|126640115|ref|NC_009085.1| | 3715387 | 3715548 | 162 |
| exprReg_25237 | gi|126640115|ref|NC_009085.1| | 3715570 | 3715618 | 49 |
| exprReg_25238 | gi|126640115|ref|NC_009085.1| | 3715678 | 3715698 | 21 |
| exprReg_25239 | gi|126640115|ref|NC_009085.1| | 3715704 | 3715814 | 111 |
| exprReg_25240 | gi|126640115|ref|NC_009085.1| | 3715869 | 3715909 | 41 |
| exprReg_25241 | gi|126640115|ref|NC_009085.1| | 3715933 | 3715985 | 53 |
| exprReg_25242 | gi|126640115|ref|NC_009085.1| | 3716215 | 3716290 | 76 |
| exprReg_25259 | gi|126640115|ref|NC_009085.1| | 3719530 | 3719596 | 67 |
| exprReg_25260 | gi|126640115|ref|NC_009085.1| | 3719705 | 3719721 | 17 |
| exprReg_25273 | gi|126640115|ref|NC_009085.1| | 3721487 | 3721513 | 27 |
| exprReg_25278 | gi|126640115|ref|NC_009085.1| | 3722361 | 3722414 | 54 |
| exprReg_25303 | gi|126640115|ref|NC_009085.1| | 3726752 | 3726840 | 89 |
| exprReg_25309 | gi|126640115|ref|NC_009085.1| | 3727307 | 3727323 | 17 |
| exprReg_25310 | gi|126640115|ref|NC_009085.1| | 3727345 | 3727404 | 60 |
| exprReg_25321 | gi|126640115|ref|NC_009085.1| | 3729741 | 3729760 | 20 |
| exprReg_25322 | gi|126640115|ref|NC_009085.1| | 3729771 | 3729795 | 25 |
| exprReg_25326 | gi|126640115|ref|NC_009085.1| | 3730271 | 3730290 | 20 |
| exprReg_25327 | gi|126640115|ref|NC_009085.1| | 3730333 | 3730353 | 21 |
| exprReg_25339 | gi|126640115|ref|NC_009085.1| | 3732416 | 3732440 | 25 |
| exprReg_25364 | gi|126640115|ref|NC_009085.1| | 3735862 | 3735884 | 23 |
| exprReg_25365 | gi|126640115|ref|NC_009085.1| | 3735909 | 3735933 | 25 |
| exprReg_25375 | gi|126640115|ref|NC_009085.1| | 3737341 | 3737486 | 146 |
| exprReg_25376 | gi|126640115|ref|NC_009085.1| | 3737490 | 3737520 | 31 |
| exprReg_25386 | gi|126640115|ref|NC_009085.1| | 3738470 | 3738488 | 19 |
| exprReg_25387 | gi|126640115|ref|NC_009085.1| | 3738495 | 3738511 | 17 |
| exprReg_25419 | gi|126640115|ref|NC_009085.1| | 3743958 | 3744005 | 48 |
| exprReg_25420 | gi|126640115|ref|NC_009085.1| | 3744013 | 3744052 | 40 |
| exprReg_25422 | gi|126640115|ref|NC_009085.1| | 3744480 | 3744510 | 31 |
| exprReg_25423 | gi|126640115|ref|NC_009085.1| | 3744588 | 3744609 | 22 |
| exprReg_25435 | gi|126640115|ref|NC_009085.1| | 3746139 | 3746206 | 68 |
| exprReg_25436 | gi|126640115|ref|NC_009085.1| | 3746238 | 3746280 | 43 |
| exprReg_25437 | gi|126640115|ref|NC_009085.1| | 3746410 | 3746506 | 97 |
| exprReg_25438 | gi|126640115|ref|NC_009085.1| | 3746579 | 3746615 | 37 |
| exprReg_25439 | gi|126640115|ref|NC_009085.1| | 3746658 | 3746674 | 17 |
| exprReg_25440 | gi|126640115|ref|NC_009085.1| | 3746679 | 3746767 | 89 |
| exprReg_25441 | gi|126640115|ref|NC_009085.1| | 3746800 | 3746842 | 43 |
| exprReg_25444 | gi|126640115|ref|NC_009085.1| | 3747690 | 3747717 | 28 |
| exprReg_25445 | gi|126640115|ref|NC_009085.1| | 3747774 | 3747862 | 89 |
| exprReg_25449 | gi|126640115|ref|NC_009085.1| | 3748754 | 3748813 | 60 |
| exprReg_25454 | gi|126640115|ref|NC_009085.1| | 3749420 | 3749468 | 49 |
| exprReg_25455 | gi|126640115|ref|NC_009085.1| | 3749620 | 3749649 | 30 |
| exprReg_25456 | gi|126640115|ref|NC_009085.1| | 3749711 | 3749799 | 89 |
| exprReg_25457 | gi|126640115|ref|NC_009085.1| | 3749813 | 3749833 | 21 |
| exprReg_25458 | gi|126640115|ref|NC_009085.1| | 3749852 | 3749889 | 38 |
| exprReg_25459 | gi|126640115|ref|NC_009085.1| | 3749897 | 3749927 | 31 |
| exprReg_25460 | gi|126640115|ref|NC_009085.1| | 3749971 | 3749987 | 17 |
| exprReg_25461 | gi|126640115|ref|NC_009085.1| | 3749993 | 3750034 | 42 |
| exprReg_25462 | gi|126640115|ref|NC_009085.1| | 3750092 | 3750131 | 40 |
| exprReg_25472 | gi|126640115|ref|NC_009085.1| | 3751243 | 3751277 | 35 |
| exprReg_25473 | gi|126640115|ref|NC_009085.1| | 3751348 | 3751382 | 35 |
| exprReg_25474 | gi|126640115|ref|NC_009085.1| | 3751388 | 3751505 | 118 |
| exprReg_25475 | gi|126640115|ref|NC_009085.1| | 3751588 | 3751610 | 23 |
| exprReg_25476 | gi|126640115|ref|NC_009085.1| | 3751631 | 3751708 | 78 |
| exprReg_25477 | gi|126640115|ref|NC_009085.1| | 3751736 | 3751778 | 43 |
| exprReg_25478 | gi|126640115|ref|NC_009085.1| | 3751884 | 3752026 | 143 |
| exprReg_25479 | gi|126640115|ref|NC_009085.1| | 3752029 | 3752167 | 139 |
| exprReg_25480 | gi|126640115|ref|NC_009085.1| | 3752253 | 3752300 | 48 |
| exprReg_25481 | gi|126640115|ref|NC_009085.1| | 3752373 | 3752391 | 19 |
| exprReg_25482 | gi|126640115|ref|NC_009085.1| | 3752396 | 3752413 | 18 |
| exprReg_25483 | gi|126640115|ref|NC_009085.1| | 3752438 | 3752463 | 26 |
| exprReg_25484 | gi|126640115|ref|NC_009085.1| | 3752527 | 3752549 | 23 |
| exprReg_25493 | gi|126640115|ref|NC_009085.1| | 3754155 | 3754179 | 25 |
| exprReg_25494 | gi|126640115|ref|NC_009085.1| | 3754216 | 3754603 | 388 |
| exprReg_25495 | gi|126640115|ref|NC_009085.1| | 3754705 | 3754746 | 42 |
| exprReg_25496 | gi|126640115|ref|NC_009085.1| | 3754777 | 3754844 | 68 |
| exprReg_25497 | gi|126640115|ref|NC_009085.1| | 3754875 | 3754891 | 17 |
| exprReg_25498 | gi|126640115|ref|NC_009085.1| | 3754899 | 3754934 | 36 |
| exprReg_25499 | gi|126640115|ref|NC_009085.1| | 3754937 | 3755028 | 92 |
| exprReg_25500 | gi|126640115|ref|NC_009085.1| | 3755044 | 3755541 | 498 |
| exprReg_25516 | gi|126640115|ref|NC_009085.1| | 3758143 | 3758224 | 82 |
| exprReg_25523 | gi|126640115|ref|NC_009085.1| | 3759016 | 3759044 | 29 |
| exprReg_25544 | gi|126640115|ref|NC_009085.1| | 3762343 | 3762403 | 61 |
| exprReg_25546 | gi|126640115|ref|NC_009085.1| | 3762586 | 3762605 | 20 |
| exprReg_25547 | gi|126640115|ref|NC_009085.1| | 3762750 | 3762810 | 61 |
| exprReg_25551 | gi|126640115|ref|NC_009085.1| | 3763230 | 3763362 | 133 |
| exprReg_25571 | gi|126640115|ref|NC_009085.1| | 3766283 | 3766421 | 139 |
| exprReg_25577 | gi|126640115|ref|NC_009085.1| | 3767388 | 3767455 | 68 |
| exprReg_25578 | gi|126640115|ref|NC_009085.1| | 3767535 | 3767560 | 26 |
| exprReg_25606 | gi|126640115|ref|NC_009085.1| | 3772006 | 3772135 | 130 |
| exprReg_25607 | gi|126640115|ref|NC_009085.1| | 3772144 | 3772161 | 18 |
| exprReg_25608 | gi|126640115|ref|NC_009085.1| | 3772177 | 3772196 | 20 |
| exprReg_25609 | gi|126640115|ref|NC_009085.1| | 3772219 | 3772246 | 28 |
| exprReg_25610 | gi|126640115|ref|NC_009085.1| | 3772263 | 3772280 | 18 |
| exprReg_25626 | gi|126640115|ref|NC_009085.1| | 3775231 | 3775285 | 55 |
| exprReg_25627 | gi|126640115|ref|NC_009085.1| | 3775420 | 3775485 | 66 |
| exprReg_25660 | gi|126640115|ref|NC_009085.1| | 3780673 | 3780690 | 18 |
| exprReg_25661 | gi|126640115|ref|NC_009085.1| | 3780744 | 3780818 | 75 |
| exprReg_25662 | gi|126640115|ref|NC_009085.1| | 3780826 | 3780878 | 53 |
| exprReg_25665 | gi|126640115|ref|NC_009085.1| | 3781365 | 3781405 | 41 |
| exprReg_25670 | gi|126640115|ref|NC_009085.1| | 3782129 | 3782157 | 29 |
| exprReg_25683 | gi|126640115|ref|NC_009085.1| | 3783658 | 3783809 | 152 |
| exprReg_25684 | gi|126640115|ref|NC_009085.1| | 3783836 | 3784173 | 338 |
| exprReg_25685 | gi|126640115|ref|NC_009085.1| | 3784222 | 3784245 | 24 |
| exprReg_25718 | gi|126640115|ref|NC_009085.1| | 3788738 | 3788966 | 229 |
| exprReg_25719 | gi|126640115|ref|NC_009085.1| | 3789032 | 3789142 | 111 |
| exprReg_25720 | gi|126640115|ref|NC_009085.1| | 3789189 | 3789249 | 61 |
| exprReg_25721 | gi|126640115|ref|NC_009085.1| | 3789386 | 3789402 | 17 |
| exprReg_25722 | gi|126640115|ref|NC_009085.1| | 3789481 | 3789519 | 39 |
| exprReg_25723 | gi|126640115|ref|NC_009085.1| | 3789635 | 3789689 | 55 |
| exprReg_25724 | gi|126640115|ref|NC_009085.1| | 3789693 | 3789725 | 33 |
| exprReg_25725 | gi|126640115|ref|NC_009085.1| | 3789735 | 3789751 | 17 |
| exprReg_25726 | gi|126640115|ref|NC_009085.1| | 3789758 | 3789803 | 46 |
| exprReg_25736 | gi|126640115|ref|NC_009085.1| | 3792670 | 3792696 | 27 |
| exprReg_25741 | gi|126640115|ref|NC_009085.1| | 3793398 | 3793453 | 56 |
| exprReg_25759 | gi|126640115|ref|NC_009085.1| | 3795789 | 3795959 | 171 |
| exprReg_25783 | gi|126640115|ref|NC_009085.1| | 3799494 | 3799523 | 30 |
| exprReg_25795 | gi|126640115|ref|NC_009085.1| | 3801172 | 3801272 | 101 |
| exprReg_25796 | gi|126640115|ref|NC_009085.1| | 3801309 | 3801331 | 23 |
| exprReg_25804 | gi|126640115|ref|NC_009085.1| | 3802451 | 3802470 | 20 |
| exprReg_25811 | gi|126640115|ref|NC_009085.1| | 3803508 | 3803573 | 66 |
| exprReg_25813 | gi|126640115|ref|NC_009085.1| | 3803983 | 3804023 | 41 |
| exprReg_25814 | gi|126640115|ref|NC_009085.1| | 3804027 | 3804056 | 30 |
| exprReg_25819 | gi|126640115|ref|NC_009085.1| | 3804646 | 3804664 | 19 |
| exprReg_25831 | gi|126640115|ref|NC_009085.1| | 3806567 | 3806689 | 123 |
| exprReg_25832 | gi|126640115|ref|NC_009085.1| | 3806734 | 3806770 | 37 |
| exprReg_25840 | gi|126640115|ref|NC_009085.1| | 3807528 | 3807576 | 49 |
| exprReg_25860 | gi|126640115|ref|NC_009085.1| | 3810442 | 3810608 | 167 |
| exprReg_25861 | gi|126640115|ref|NC_009085.1| | 3810620 | 3810675 | 56 |
| exprReg_25868 | gi|126640115|ref|NC_009085.1| | 3812234 | 3812259 | 26 |
| exprReg_25877 | gi|126640115|ref|NC_009085.1| | 3813427 | 3813481 | 55 |
| exprReg_25878 | gi|126640115|ref|NC_009085.1| | 3813708 | 3813743 | 36 |
| exprReg_25897 | gi|126640115|ref|NC_009085.1| | 3816211 | 3816233 | 23 |
| exprReg_25910 | gi|126640115|ref|NC_009085.1| | 3818223 | 3818241 | 19 |
| exprReg_25911 | gi|126640115|ref|NC_009085.1| | 3818265 | 3818408 | 144 |
| exprReg_25925 | gi|126640115|ref|NC_009085.1| | 3821317 | 3821340 | 24 |
| exprReg_25926 | gi|126640115|ref|NC_009085.1| | 3821562 | 3821607 | 46 |
| exprReg_25939 | gi|126640115|ref|NC_009085.1| | 3823372 | 3823395 | 24 |
| exprReg_25940 | gi|126640115|ref|NC_009085.1| | 3823403 | 3823430 | 28 |
| exprReg_25942 | gi|126640115|ref|NC_009085.1| | 3823724 | 3823745 | 22 |
| exprReg_25949 | gi|126640115|ref|NC_009085.1| | 3824316 | 3824580 | 265 |
| exprReg_25950 | gi|126640115|ref|NC_009085.1| | 3824697 | 3824713 | 17 |
| exprReg_25951 | gi|126640115|ref|NC_009085.1| | 3824795 | 3824819 | 25 |
| exprReg_25984 | gi|126640115|ref|NC_009085.1| | 3829858 | 3829964 | 107 |
| exprReg_25987 | gi|126640115|ref|NC_009085.1| | 3830497 | 3830656 | 160 |
| exprReg_25988 | gi|126640115|ref|NC_009085.1| | 3830672 | 3830748 | 77 |
| exprReg_25989 | gi|126640115|ref|NC_009085.1| | 3830754 | 3831075 | 322 |
| exprReg_26014 | gi|126640115|ref|NC_009085.1| | 3834860 | 3834876 | 17 |
| exprReg_26043 | gi|126640115|ref|NC_009085.1| | 3839479 | 3839506 | 28 |
| exprReg_26044 | gi|126640115|ref|NC_009085.1| | 3839512 | 3839547 | 36 |
| exprReg_26045 | gi|126640115|ref|NC_009085.1| | 3839564 | 3839612 | 49 |
| exprReg_26050 | gi|126640115|ref|NC_009085.1| | 3840545 | 3840604 | 60 |
| exprReg_26064 | gi|126640115|ref|NC_009085.1| | 3843385 | 3843517 | 133 |
| exprReg_26065 | gi|126640115|ref|NC_009085.1| | 3843595 | 3843627 | 33 |
| exprReg_26066 | gi|126640115|ref|NC_009085.1| | 3843631 | 3843682 | 52 |
| exprReg_26082 | gi|126640115|ref|NC_009085.1| | 3846840 | 3846875 | 36 |
| exprReg_26083 | gi|126640115|ref|NC_009085.1| | 3846889 | 3846935 | 47 |
| exprReg_26084 | gi|126640115|ref|NC_009085.1| | 3846965 | 3847072 | 108 |
| exprReg_26094 | gi|126640115|ref|NC_009085.1| | 3847934 | 3848007 | 74 |
| exprReg_26113 | gi|126640115|ref|NC_009085.1| | 3850200 | 3850296 | 97 |
| exprReg_26123 | gi|126640115|ref|NC_009085.1| | 3851771 | 3851788 | 18 |
| exprReg_26124 | gi|126640115|ref|NC_009085.1| | 3851801 | 3851832 | 32 |
| exprReg_26133 | gi|126640115|ref|NC_009085.1| | 3853178 | 3853276 | 99 |
| exprReg_26148 | gi|126640115|ref|NC_009085.1| | 3856553 | 3856572 | 20 |
| exprReg_26149 | gi|126640115|ref|NC_009085.1| | 3856605 | 3856724 | 120 |
| exprReg_26161 | gi|126640115|ref|NC_009085.1| | 3858318 | 3858376 | 59 |
| exprReg_26162 | gi|126640115|ref|NC_009085.1| | 3858388 | 3858511 | 124 |
| exprReg_26163 | gi|126640115|ref|NC_009085.1| | 3858592 | 3858617 | 26 |
| exprReg_26164 | gi|126640115|ref|NC_009085.1| | 3858692 | 3858710 | 19 |
| exprReg_26174 | gi|126640115|ref|NC_009085.1| | 3860243 | 3860268 | 26 |
| exprReg_26193 | gi|126640115|ref|NC_009085.1| | 3862761 | 3862824 | 64 |
| exprReg_26201 | gi|126640115|ref|NC_009085.1| | 3864432 | 3864493 | 62 |
| exprReg_26202 | gi|126640115|ref|NC_009085.1| | 3864638 | 3864680 | 43 |
| exprReg_26203 | gi|126640115|ref|NC_009085.1| | 3864721 | 3864795 | 75 |
| exprReg_26204 | gi|126640115|ref|NC_009085.1| | 3864971 | 3864988 | 18 |
[truncated: 12,249 more chars]
