# Supplementary material for: Global assessment of small RNAs reveals a non-coding transcript involved in biofilm formation and attachment in Acinetobacter baumannii ATCC 17978
Source: PLoS One. 2017 Aug 1;12(8):e0182084. doi: 10.1371/journal.pone.0182084 (PMC5538643; doi:10.1371/journal.pone.0182084)
Supplement: S2 Dataset — List of normalized expression of 255 coding regions (expression score should be equal or above 7.6 in some growing condition: Bio, Exp or Sta), their length and locations in the genome (NC_009085.1) or plasmid (NC_009084.1) of A. baumannii ATCC 17978. (DOCX) [file pone.0182084.s009.docx]

**S2 Dataset.** **Coding regions.** List of normalized expression of 255 coding regions (expression score should be equal or above 7.6 in some growing condition: Bio, Exp or Sta), their length and locations in the genome (NC_009085.1) or plasmid (NC_009084.1) of *A. baumannii* ATCC 17978.

| **Expressed region** | **Genetic element** | **Start** | **End** | **Length** |
| --- | --- | --- | --- | --- |
| exprReg_29 | gi\|126640109\|ref\|NC_009084.1\| | 11279 | 11302 | 25 |
| exprReg_165 | gi\|126640115\|ref\|NC_009085.1\| | 7618 | 7679 | 62 |
| exprReg_424 | gi\|126640115\|ref\|NC_009085.1\| | 45985 | 46023 | 39 |
| exprReg_497 | gi\|126640115\|ref\|NC_009085.1\| | 55988 | 56010 | 23 |
| exprReg_598 | gi\|126640115\|ref\|NC_009085.1\| | 68496 | 68582 | 87 |
| exprReg_601 | gi\|126640115\|ref\|NC_009085.1\| | 68862 | 68924 | 63 |
| exprReg_602 | gi\|126640115\|ref\|NC_009085.1\| | 68993 | 69200 | 208 |
| exprReg_611 | gi\|126640115\|ref\|NC_009085.1\| | 70158 | 70322 | 165 |
| exprReg_786 | gi\|126640115\|ref\|NC_009085.1\| | 95494 | 95609 | 116 |
| exprReg_788 | gi\|126640115\|ref\|NC_009085.1\| | 95795 | 95833 | 39 |
| exprReg_795 | gi\|126640115\|ref\|NC_009085.1\| | 96670 | 96783 | 114 |
| exprReg_801 | gi\|126640115\|ref\|NC_009085.1\| | 97585 | 97700 | 116 |
| exprReg_805 | gi\|126640115\|ref\|NC_009085.1\| | 98405 | 98649 | 245 |
| exprReg_833 | gi\|126640115\|ref\|NC_009085.1\| | 103545 | 103589 | 45 |
| exprReg_1004 | gi\|126640115\|ref\|NC_009085.1\| | 131905 | 131929 | 25 |
| exprReg_1136 | gi\|126640115\|ref\|NC_009085.1\| | 151616 | 151683 | 68 |
| exprReg_1221 | gi\|126640115\|ref\|NC_009085.1\| | 161963 | 162062 | 100 |
| exprReg_1368 | gi\|126640115\|ref\|NC_009085.1\| | 184272 | 184399 | 128 |
| exprReg_1435 | gi\|126640115\|ref\|NC_009085.1\| | 201350 | 201426 | 77 |
| exprReg_1571 | gi\|126640115\|ref\|NC_009085.1\| | 220077 | 220117 | 41 |
| exprReg_1994 | gi\|126640115\|ref\|NC_009085.1\| | 283843 | 283983 | 141 |
| exprReg_2005 | gi\|126640115\|ref\|NC_009085.1\| | 285684 | 285752 | 69 |
| exprReg_2006 | gi\|126640115\|ref\|NC_009085.1\| | 285754 | 285836 | 83 |
| exprReg_2119 | gi\|126640115\|ref\|NC_009085.1\| | 304207 | 304365 | 159 |
| exprReg_2247 | gi\|126640115\|ref\|NC_009085.1\| | 323784 | 323872 | 89 |
| exprReg_2304 | gi\|126640115\|ref\|NC_009085.1\| | 334867 | 335065 | 199 |
| exprReg_2345 | gi\|126640115\|ref\|NC_009085.1\| | 341597 | 341666 | 70 |
| exprReg_2350 | gi\|126640115\|ref\|NC_009085.1\| | 342271 | 342369 | 99 |
| exprReg_2359 | gi\|126640115\|ref\|NC_009085.1\| | 343455 | 343514 | 60 |
| exprReg_2384 | gi\|126640115\|ref\|NC_009085.1\| | 348114 | 348174 | 61 |
| exprReg_2492 | gi\|126640115\|ref\|NC_009085.1\| | 364098 | 364184 | 87 |
| exprReg_2675 | gi\|126640115\|ref\|NC_009085.1\| | 392646 | 392809 | 164 |
| exprReg_2677 | gi\|126640115\|ref\|NC_009085.1\| | 393097 | 393243 | 147 |
| exprReg_2711 | gi\|126640115\|ref\|NC_009085.1\| | 398734 | 398921 | 188 |
| exprReg_2766 | gi\|126640115\|ref\|NC_009085.1\| | 406231 | 406340 | 110 |
| exprReg_2811 | gi\|126640115\|ref\|NC_009085.1\| | 412784 | 412829 | 46 |
| exprReg_2872 | gi\|126640115\|ref\|NC_009085.1\| | 422652 | 422726 | 75 |
| exprReg_2892 | gi\|126640115\|ref\|NC_009085.1\| | 425564 | 425594 | 31 |
| exprReg_2988 | gi\|126640115\|ref\|NC_009085.1\| | 438612 | 439314 | 703 |
| exprReg_3084 | gi\|126640115\|ref\|NC_009085.1\| | 453718 | 453748 | 31 |
| exprReg_3176 | gi\|126640115\|ref\|NC_009085.1\| | 467676 | 467846 | 171 |
| exprReg_3275 | gi\|126640115\|ref\|NC_009085.1\| | 481382 | 481405 | 24 |
| exprReg_3326 | gi\|126640115\|ref\|NC_009085.1\| | 488732 | 488918 | 187 |
| exprReg_3333 | gi\|126640115\|ref\|NC_009085.1\| | 489769 | 489878 | 110 |
| exprReg_3380 | gi\|126640115\|ref\|NC_009085.1\| | 496363 | 496466 | 104 |
| exprReg_3440 | gi\|126640115\|ref\|NC_009085.1\| | 506150 | 506259 | 110 |
| exprReg_3477 | gi\|126640115\|ref\|NC_009085.1\| | 511249 | 511338 | 90 |
| exprReg_3641 | gi\|126640115\|ref\|NC_009085.1\| | 534346 | 534381 | 36 |
| exprReg_3667 | gi\|126640115\|ref\|NC_009085.1\| | 537765 | 537823 | 59 |
| exprReg_4045 | gi\|126640115\|ref\|NC_009085.1\| | 590214 | 590424 | 211 |
| exprReg_4486 | gi\|126640115\|ref\|NC_009085.1\| | 651955 | 652382 | 428 |
| exprReg_4493 | gi\|126640115\|ref\|NC_009085.1\| | 653394 | 653451 | 58 |
| exprReg_4505 | gi\|126640115\|ref\|NC_009085.1\| | 654771 | 654826 | 56 |
| exprReg_4724 | gi\|126640115\|ref\|NC_009085.1\| | 682472 | 682771 | 300 |
| exprReg_4750 | gi\|126640115\|ref\|NC_009085.1\| | 686048 | 686151 | 104 |
| exprReg_4776 | gi\|126640115\|ref\|NC_009085.1\| | 690255 | 690310 | 56 |
| exprReg_4846 | gi\|126640115\|ref\|NC_009085.1\| | 700057 | 700126 | 70 |
| exprReg_4946 | gi\|126640115\|ref\|NC_009085.1\| | 713784 | 713875 | 92 |
| exprReg_4953 | gi\|126640115\|ref\|NC_009085.1\| | 714551 | 714600 | 50 |
| exprReg_4954 | gi\|126640115\|ref\|NC_009085.1\| | 714618 | 714967 | 350 |
| exprReg_5036 | gi\|126640115\|ref\|NC_009085.1\| | 725168 | 725281 | 114 |
| exprReg_5059 | gi\|126640115\|ref\|NC_009085.1\| | 728051 | 728270 | 220 |
| exprReg_5077 | gi\|126640115\|ref\|NC_009085.1\| | 730587 | 730639 | 53 |
| exprReg_5098 | gi\|126640115\|ref\|NC_009085.1\| | 733843 | 733864 | 22 |
| exprReg_5148 | gi\|126640115\|ref\|NC_009085.1\| | 740901 | 740938 | 38 |
| exprReg_5168 | gi\|126640115\|ref\|NC_009085.1\| | 743902 | 743921 | 20 |
| exprReg_5286 | gi\|126640115\|ref\|NC_009085.1\| | 761346 | 761400 | 55 |
| exprReg_5428 | gi\|126640115\|ref\|NC_009085.1\| | 782361 | 782378 | 18 |
| exprReg_5483 | gi\|126640115\|ref\|NC_009085.1\| | 795059 | 795568 | 510 |
| exprReg_5498 | gi\|126640115\|ref\|NC_009085.1\| | 799756 | 799861 | 106 |
| exprReg_5505 | gi\|126640115\|ref\|NC_009085.1\| | 800862 | 800919 | 58 |
| exprReg_5682 | gi\|126640115\|ref\|NC_009085.1\| | 837562 | 837776 | 215 |
| exprReg_5881 | gi\|126640115\|ref\|NC_009085.1\| | 871472 | 871833 | 362 |
| exprReg_5882 | gi\|126640115\|ref\|NC_009085.1\| | 872010 | 872197 | 188 |
| exprReg_5954 | gi\|126640115\|ref\|NC_009085.1\| | 882356 | 882380 | 25 |
| exprReg_5958 | gi\|126640115\|ref\|NC_009085.1\| | 882910 | 882973 | 64 |
| exprReg_5985 | gi\|126640115\|ref\|NC_009085.1\| | 887664 | 887684 | 21 |
| exprReg_6714 | gi\|126640115\|ref\|NC_009085.1\| | 987073 | 987168 | 96 |
| exprReg_6904 | gi\|126640115\|ref\|NC_009085.1\| | 1016932 | 1017048 | 117 |
| exprReg_7112 | gi\|126640115\|ref\|NC_009085.1\| | 1045778 | 1046079 | 302 |
| exprReg_7178 | gi\|126640115\|ref\|NC_009085.1\| | 1055718 | 1056190 | 473 |
| exprReg_7203 | gi\|126640115\|ref\|NC_009085.1\| | 1059464 | 1059648 | 185 |
| exprReg_7208 | gi\|126640115\|ref\|NC_009085.1\| | 1060103 | 1060362 | 260 |
| exprReg_7294 | gi\|126640115\|ref\|NC_009085.1\| | 1073607 | 1073634 | 28 |
| exprReg_7297 | gi\|126640115\|ref\|NC_009085.1\| | 1073971 | 1074008 | 38 |
| exprReg_7468 | gi\|126640115\|ref\|NC_009085.1\| | 1098721 | 1098759 | 39 |
| exprReg_7562 | gi\|126640115\|ref\|NC_009085.1\| | 1114427 | 1114450 | 24 |
| exprReg_7565 | gi\|126640115\|ref\|NC_009085.1\| | 1114591 | 1114615 | 25 |
| exprReg_7566 | gi\|126640115\|ref\|NC_009085.1\| | 1114627 | 1114674 | 48 |
| exprReg_7667 | gi\|126640115\|ref\|NC_009085.1\| | 1131636 | 1131939 | 304 |
| exprReg_7923 | gi\|126640115\|ref\|NC_009085.1\| | 1167542 | 1167561 | 20 |
| exprReg_8014 | gi\|126640115\|ref\|NC_009085.1\| | 1182460 | 1182529 | 70 |
| exprReg_8052 | gi\|126640115\|ref\|NC_009085.1\| | 1188512 | 1188604 | 93 |
| exprReg_8136 | gi\|126640115\|ref\|NC_009085.1\| | 1200447 | 1200679 | 233 |
| exprReg_8382 | gi\|126640115\|ref\|NC_009085.1\| | 1234413 | 1234481 | 69 |
| exprReg_8583 | gi\|126640115\|ref\|NC_009085.1\| | 1261680 | 1261901 | 222 |
| exprReg_8776 | gi\|126640115\|ref\|NC_009085.1\| | 1290847 | 1290897 | 51 |
| exprReg_9139 | gi\|126640115\|ref\|NC_009085.1\| | 1349954 | 1349975 | 22 |
| exprReg_9255 | gi\|126640115\|ref\|NC_009085.1\| | 1369692 | 1369766 | 75 |
| exprReg_9276 | gi\|126640115\|ref\|NC_009085.1\| | 1372640 | 1372840 | 201 |
| exprReg_9378 | gi\|126640115\|ref\|NC_009085.1\| | 1385884 | 1386195 | 312 |
| exprReg_9507 | gi\|126640115\|ref\|NC_009085.1\| | 1405051 | 1405103 | 53 |
| exprReg_9656 | gi\|126640115\|ref\|NC_009085.1\| | 1427782 | 1427945 | 164 |
| exprReg_9657 | gi\|126640115\|ref\|NC_009085.1\| | 1428086 | 1428282 | 197 |
| exprReg_9660 | gi\|126640115\|ref\|NC_009085.1\| | 1428931 | 1429082 | 152 |
| exprReg_9720 | gi\|126640115\|ref\|NC_009085.1\| | 1438838 | 1438864 | 27 |
| exprReg_9803 | gi\|126640115\|ref\|NC_009085.1\| | 1450363 | 1450379 | 17 |
| exprReg_9878 | gi\|126640115\|ref\|NC_009085.1\| | 1461038 | 1461089 | 52 |
| exprReg_9941 | gi\|126640115\|ref\|NC_009085.1\| | 1469423 | 1469790 | 368 |
| exprReg_9947 | gi\|126640115\|ref\|NC_009085.1\| | 1470399 | 1470435 | 37 |
| exprReg_9950 | gi\|126640115\|ref\|NC_009085.1\| | 1470884 | 1471200 | 317 |
| exprReg_10021 | gi\|126640115\|ref\|NC_009085.1\| | 1481683 | 1481716 | 34 |
| exprReg_10022 | gi\|126640115\|ref\|NC_009085.1\| | 1481787 | 1482138 | 352 |
| exprReg_10085 | gi\|126640115\|ref\|NC_009085.1\| | 1492105 | 1492135 | 31 |
| exprReg_10089 | gi\|126640115\|ref\|NC_009085.1\| | 1492824 | 1492979 | 156 |
| exprReg_10090 | gi\|126640115\|ref\|NC_009085.1\| | 1493140 | 1493445 | 306 |
| exprReg_10434 | gi\|126640115\|ref\|NC_009085.1\| | 1546041 | 1546152 | 112 |
| exprReg_10453 | gi\|126640115\|ref\|NC_009085.1\| | 1549197 | 1549402 | 206 |
| exprReg_10493 | gi\|126640115\|ref\|NC_009085.1\| | 1555745 | 1555844 | 100 |
| exprReg_10665 | gi\|126640115\|ref\|NC_009085.1\| | 1580921 | 1581212 | 292 |
| exprReg_10800 | gi\|126640115\|ref\|NC_009085.1\| | 1605174 | 1605243 | 70 |
| exprReg_10859 | gi\|126640115\|ref\|NC_009085.1\| | 1613999 | 1614097 | 99 |
| exprReg_10907 | gi\|126640115\|ref\|NC_009085.1\| | 1620810 | 1621033 | 224 |
| exprReg_11025 | gi\|126640115\|ref\|NC_009085.1\| | 1638554 | 1638607 | 54 |
| exprReg_11065 | gi\|126640115\|ref\|NC_009085.1\| | 1646089 | 1646202 | 114 |
| exprReg_11473 | gi\|126640115\|ref\|NC_009085.1\| | 1710119 | 1710168 | 50 |
| exprReg_11798 | gi\|126640115\|ref\|NC_009085.1\| | 1759130 | 1759182 | 53 |
| exprReg_11841 | gi\|126640115\|ref\|NC_009085.1\| | 1764241 | 1764257 | 17 |
| exprReg_11854 | gi\|126640115\|ref\|NC_009085.1\| | 1765388 | 1765466 | 79 |
| exprReg_12046 | gi\|126640115\|ref\|NC_009085.1\| | 1790539 | 1790609 | 71 |
| exprReg_12048 | gi\|126640115\|ref\|NC_009085.1\| | 1790680 | 1790702 | 23 |
| exprReg_12150 | gi\|126640115\|ref\|NC_009085.1\| | 1803495 | 1803578 | 84 |
| exprReg_12158 | gi\|126640115\|ref\|NC_009085.1\| | 1804229 | 1804257 | 29 |
| exprReg_12361 | gi\|126640115\|ref\|NC_009085.1\| | 1831821 | 1831950 | 130 |
| exprReg_12370 | gi\|126640115\|ref\|NC_009085.1\| | 1833173 | 1833196 | 24 |
| exprReg_12371 | gi\|126640115\|ref\|NC_009085.1\| | 1833214 | 1833261 | 48 |
| exprReg_12407 | gi\|126640115\|ref\|NC_009085.1\| | 1841099 | 1841136 | 38 |
| exprReg_12460 | gi\|126640115\|ref\|NC_009085.1\| | 1850622 | 1850875 | 254 |
| exprReg_12684 | gi\|126640115\|ref\|NC_009085.1\| | 1883356 | 1883408 | 53 |
| exprReg_12706 | gi\|126640115\|ref\|NC_009085.1\| | 1886082 | 1886176 | 95 |
| exprReg_12732 | gi\|126640115\|ref\|NC_009085.1\| | 1889811 | 1889881 | 71 |
| exprReg_12757 | gi\|126640115\|ref\|NC_009085.1\| | 1893571 | 1893605 | 35 |
| exprReg_13053 | gi\|126640115\|ref\|NC_009085.1\| | 1930787 | 1930856 | 70 |
| exprReg_13107 | gi\|126640115\|ref\|NC_009085.1\| | 1938252 | 1938275 | 24 |
| exprReg_13276 | gi\|126640115\|ref\|NC_009085.1\| | 1963320 | 1963418 | 99 |
| exprReg_13281 | gi\|126640115\|ref\|NC_009085.1\| | 1963997 | 1964135 | 139 |
| exprReg_13573 | gi\|126640115\|ref\|NC_009085.1\| | 2008712 | 2008787 | 76 |
| exprReg_13621 | gi\|126640115\|ref\|NC_009085.1\| | 2015329 | 2015364 | 36 |
| exprReg_13625 | gi\|126640115\|ref\|NC_009085.1\| | 2015479 | 2015561 | 83 |
| exprReg_13631 | gi\|126640115\|ref\|NC_009085.1\| | 2016553 | 2016569 | 17 |
| exprReg_13859 | gi\|126640115\|ref\|NC_009085.1\| | 2055146 | 2055452 | 307 |
| exprReg_13953 | gi\|126640115\|ref\|NC_009085.1\| | 2071244 | 2071287 | 44 |
| exprReg_14216 | gi\|126640115\|ref\|NC_009085.1\| | 2114454 | 2114647 | 194 |
| exprReg_14242 | gi\|126640115\|ref\|NC_009085.1\| | 2119088 | 2119122 | 35 |
| exprReg_14286 | gi\|126640115\|ref\|NC_009085.1\| | 2125128 | 2125153 | 26 |
| exprReg_14414 | gi\|126640115\|ref\|NC_009085.1\| | 2146771 | 2146841 | 71 |
| exprReg_14612 | gi\|126640115\|ref\|NC_009085.1\| | 2180375 | 2180471 | 97 |
| exprReg_14802 | gi\|126640115\|ref\|NC_009085.1\| | 2210834 | 2210986 | 153 |
| exprReg_14887 | gi\|126640115\|ref\|NC_009085.1\| | 2219309 | 2219333 | 25 |
| exprReg_15146 | gi\|126640115\|ref\|NC_009085.1\| | 2257149 | 2257219 | 71 |
| exprReg_15147 | gi\|126640115\|ref\|NC_009085.1\| | 2257604 | 2257740 | 137 |
| exprReg_15214 | gi\|126640115\|ref\|NC_009085.1\| | 2266406 | 2266433 | 28 |
| exprReg_15677 | gi\|126640115\|ref\|NC_009085.1\| | 2330052 | 2330070 | 19 |
| exprReg_15861 | gi\|126640115\|ref\|NC_009085.1\| | 2363453 | 2363475 | 23 |
| exprReg_15870 | gi\|126640115\|ref\|NC_009085.1\| | 2365007 | 2365127 | 121 |
| exprReg_15894 | gi\|126640115\|ref\|NC_009085.1\| | 2371051 | 2371114 | 64 |
| exprReg_15955 | gi\|126640115\|ref\|NC_009085.1\| | 2380211 | 2380304 | 94 |
| exprReg_16106 | gi\|126640115\|ref\|NC_009085.1\| | 2397995 | 2398090 | 96 |
| exprReg_16107 | gi\|126640115\|ref\|NC_009085.1\| | 2398110 | 2398131 | 22 |
| exprReg_16117 | gi\|126640115\|ref\|NC_009085.1\| | 2399766 | 2399806 | 41 |
| exprReg_16257 | gi\|126640115\|ref\|NC_009085.1\| | 2421738 | 2421754 | 17 |
| exprReg_16325 | gi\|126640115\|ref\|NC_009085.1\| | 2432018 | 2432233 | 216 |
| exprReg_16412 | gi\|126640115\|ref\|NC_009085.1\| | 2445139 | 2445179 | 41 |
| exprReg_16531 | gi\|126640115\|ref\|NC_009085.1\| | 2462500 | 2462543 | 44 |
| exprReg_16559 | gi\|126640115\|ref\|NC_009085.1\| | 2466256 | 2466360 | 105 |
| exprReg_16574 | gi\|126640115\|ref\|NC_009085.1\| | 2468535 | 2468556 | 22 |
| exprReg_16705 | gi\|126640115\|ref\|NC_009085.1\| | 2487149 | 2487251 | 103 |
| exprReg_16723 | gi\|126640115\|ref\|NC_009085.1\| | 2490084 | 2490217 | 134 |
| exprReg_16724 | gi\|126640115\|ref\|NC_009085.1\| | 2490221 | 2490336 | 116 |
| exprReg_16912 | gi\|126640115\|ref\|NC_009085.1\| | 2516685 | 2516740 | 56 |
| exprReg_17288 | gi\|126640115\|ref\|NC_009085.1\| | 2570483 | 2570804 | 322 |
| exprReg_17299 | gi\|126640115\|ref\|NC_009085.1\| | 2573519 | 2573540 | 22 |
| exprReg_17531 | gi\|126640115\|ref\|NC_009085.1\| | 2606865 | 2607055 | 191 |
| exprReg_17548 | gi\|126640115\|ref\|NC_009085.1\| | 2609422 | 2609458 | 37 |
| exprReg_17642 | gi\|126640115\|ref\|NC_009085.1\| | 2620144 | 2620160 | 17 |
| exprReg_17826 | gi\|126640115\|ref\|NC_009085.1\| | 2643795 | 2643822 | 28 |
| exprReg_17879 | gi\|126640115\|ref\|NC_009085.1\| | 2650744 | 2650987 | 244 |
| exprReg_18098 | gi\|126640115\|ref\|NC_009085.1\| | 2683074 | 2683104 | 31 |
| exprReg_18150 | gi\|126640115\|ref\|NC_009085.1\| | 2690051 | 2690131 | 81 |
| exprReg_18205 | gi\|126640115\|ref\|NC_009085.1\| | 2697668 | 2697690 | 23 |
| exprReg_18260 | gi\|126640115\|ref\|NC_009085.1\| | 2705935 | 2706076 | 142 |
| exprReg_18366 | gi\|126640115\|ref\|NC_009085.1\| | 2721668 | 2721782 | 115 |
| exprReg_18451 | gi\|126640115\|ref\|NC_009085.1\| | 2734326 | 2734390 | 65 |
| exprReg_18718 | gi\|126640115\|ref\|NC_009085.1\| | 2773218 | 2773234 | 17 |
| exprReg_18755 | gi\|126640115\|ref\|NC_009085.1\| | 2778687 | 2778891 | 205 |
| exprReg_18763 | gi\|126640115\|ref\|NC_009085.1\| | 2780112 | 2780244 | 133 |
| exprReg_18900 | gi\|126640115\|ref\|NC_009085.1\| | 2803769 | 2803984 | 216 |
| exprReg_19276 | gi\|126640115\|ref\|NC_009085.1\| | 2853518 | 2853581 | 64 |
| exprReg_19347 | gi\|126640115\|ref\|NC_009085.1\| | 2863332 | 2863384 | 53 |
| exprReg_19511 | gi\|126640115\|ref\|NC_009085.1\| | 2885554 | 2885606 | 53 |
| exprReg_19604 | gi\|126640115\|ref\|NC_009085.1\| | 2896923 | 2896942 | 20 |
| exprReg_19647 | gi\|126640115\|ref\|NC_009085.1\| | 2904150 | 2904188 | 39 |
| exprReg_19890 | gi\|126640115\|ref\|NC_009085.1\| | 2937272 | 2937320 | 49 |
| exprReg_19898 | gi\|126640115\|ref\|NC_009085.1\| | 2938250 | 2938272 | 23 |
| exprReg_19931 | gi\|126640115\|ref\|NC_009085.1\| | 2942858 | 2942916 | 59 |
| exprReg_20262 | gi\|126640115\|ref\|NC_009085.1\| | 2987531 | 2987547 | 17 |
| exprReg_20685 | gi\|126640115\|ref\|NC_009085.1\| | 3046681 | 3046755 | 75 |
| exprReg_20762 | gi\|126640115\|ref\|NC_009085.1\| | 3057544 | 3057570 | 27 |
| exprReg_20798 | gi\|126640115\|ref\|NC_009085.1\| | 3063823 | 3064045 | 223 |
| exprReg_20837 | gi\|126640115\|ref\|NC_009085.1\| | 3069184 | 3069389 | 206 |
| exprReg_21011 | gi\|126640115\|ref\|NC_009085.1\| | 3092508 | 3092624 | 117 |
| exprReg_21223 | gi\|126640115\|ref\|NC_009085.1\| | 3122701 | 3122752 | 52 |
| exprReg_21275 | gi\|126640115\|ref\|NC_009085.1\| | 3131213 | 3131229 | 17 |
| exprReg_21358 | gi\|126640115\|ref\|NC_009085.1\| | 3141846 | 3142045 | 200 |
| exprReg_21392 | gi\|126640115\|ref\|NC_009085.1\| | 3148401 | 3148666 | 266 |
| exprReg_21417 | gi\|126640115\|ref\|NC_009085.1\| | 3152777 | 3152828 | 52 |
| exprReg_21442 | gi\|126640115\|ref\|NC_009085.1\| | 3156591 | 3156701 | 111 |
| exprReg_21533 | gi\|126640115\|ref\|NC_009085.1\| | 3167946 | 3167964 | 19 |
| exprReg_21563 | gi\|126640115\|ref\|NC_009085.1\| | 3171131 | 3171341 | 211 |
| exprReg_21637 | gi\|126640115\|ref\|NC_009085.1\| | 3180836 | 3180903 | 68 |
| exprReg_21762 | gi\|126640115\|ref\|NC_009085.1\| | 3198650 | 3198744 | 95 |
| exprReg_21850 | gi\|126640115\|ref\|NC_009085.1\| | 3213397 | 3213507 | 111 |
| exprReg_22036 | gi\|126640115\|ref\|NC_009085.1\| | 3238676 | 3238721 | 46 |
| exprReg_22275 | gi\|126640115\|ref\|NC_009085.1\| | 3270621 | 3270642 | 22 |
| exprReg_22490 | gi\|126640115\|ref\|NC_009085.1\| | 3299307 | 3299331 | 25 |
| exprReg_22686 | gi\|126640115\|ref\|NC_009085.1\| | 3327351 | 3327395 | 45 |
| exprReg_22981 | gi\|126640115\|ref\|NC_009085.1\| | 3368615 | 3368662 | 48 |
| exprReg_23002 | gi\|126640115\|ref\|NC_009085.1\| | 3371281 | 3371331 | 51 |
| exprReg_23172 | gi\|126640115\|ref\|NC_009085.1\| | 3394048 | 3394091 | 44 |
| exprReg_23283 | gi\|126640115\|ref\|NC_009085.1\| | 3410172 | 3410233 | 62 |
| exprReg_23341 | gi\|126640115\|ref\|NC_009085.1\| | 3418937 | 3419153 | 217 |
| exprReg_23620 | gi\|126640115\|ref\|NC_009085.1\| | 3465987 | 3466003 | 17 |
| exprReg_23869 | gi\|126640115\|ref\|NC_009085.1\| | 3511877 | 3511907 | 31 |
| exprReg_24321 | gi\|126640115\|ref\|NC_009085.1\| | 3582264 | 3582481 | 218 |
| exprReg_24430 | gi\|126640115\|ref\|NC_009085.1\| | 3599510 | 3599661 | 152 |
| exprReg_25004 | gi\|126640115\|ref\|NC_009085.1\| | 3682245 | 3682529 | 285 |
| exprReg_25126 | gi\|126640115\|ref\|NC_009085.1\| | 3700398 | 3700547 | 150 |
| exprReg_25193 | gi\|126640115\|ref\|NC_009085.1\| | 3709191 | 3709239 | 49 |
| exprReg_25478 | gi\|126640115\|ref\|NC_009085.1\| | 3751884 | 3752026 | 143 |
| exprReg_25494 | gi\|126640115\|ref\|NC_009085.1\| | 3754216 | 3754603 | 388 |
| exprReg_25499 | gi\|126640115\|ref\|NC_009085.1\| | 3754937 | 3755028 | 92 |
| exprReg_25551 | gi\|126640115\|ref\|NC_009085.1\| | 3763230 | 3763362 | 133 |
| exprReg_25571 | gi\|126640115\|ref\|NC_009085.1\| | 3766283 | 3766421 | 139 |
| exprReg_25719 | gi\|126640115\|ref\|NC_009085.1\| | 3789032 | 3789142 | 111 |
| exprReg_25811 | gi\|126640115\|ref\|NC_009085.1\| | 3803508 | 3803573 | 66 |
| exprReg_25840 | gi\|126640115\|ref\|NC_009085.1\| | 3807528 | 3807576 | 49 |
| exprReg_25942 | gi\|126640115\|ref\|NC_009085.1\| | 3823724 | 3823745 | 22 |
| exprReg_26235 | gi\|126640115\|ref\|NC_009085.1\| | 3869034 | 3869061 | 28 |
| exprReg_26247 | gi\|126640115\|ref\|NC_009085.1\| | 3870546 | 3870595 | 50 |
| exprReg_26276 | gi\|126640115\|ref\|NC_009085.1\| | 3876036 | 3876060 | 25 |
| exprReg_26388 | gi\|126640115\|ref\|NC_009085.1\| | 3890666 | 3890805 | 140 |
| exprReg_26474 | gi\|126640115\|ref\|NC_009085.1\| | 3904795 | 3905036 | 242 |
| exprReg_26511 | gi\|126640115\|ref\|NC_009085.1\| | 3910220 | 3910275 | 56 |
| exprReg_26579 | gi\|126640115\|ref\|NC_009085.1\| | 3922555 | 3922606 | 52 |
| exprReg_26627 | gi\|126640115\|ref\|NC_009085.1\| | 3929898 | 3930486 | 589 |
