# Supplementary material for: Global assessment of small RNAs reveals a non-coding transcript involved in biofilm formation and attachment in Acinetobacter baumannii ATCC 17978
Source: PLoS One. 2017 Aug 1;12(8):e0182084. doi: 10.1371/journal.pone.0182084 (PMC5538643; doi:10.1371/journal.pone.0182084)

**S2 Fig.** **Distribution of normalized expression scores for the 5564 expressed regions** **not overlapping with known genes.** Blue: biofilm samples. Orange: exponential phase samples. Yellow: stationary phase samples.


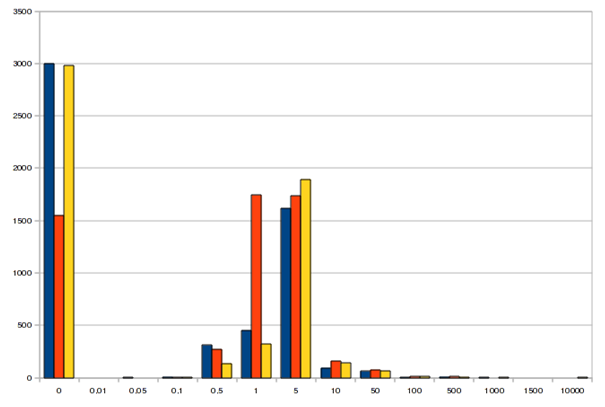

Supplement: S2 Fig — Blue: biofilm samples. Orange: exponential phase samples. Yellow: stationary phase samples. (DOCX) [file pone.0182084.s011.docx]
