# Supplementary material for: Global assessment of small RNAs reveals a non-coding transcript involved in biofilm formation and attachment in Acinetobacter baumannii ATCC 17978
Source: PLoS One. 2017 Aug 1;12(8):e0182084. doi: 10.1371/journal.pone.0182084 (PMC5538643; doi:10.1371/journal.pone.0182084)

**S3 Fig.** **Distribution of normalized expression scores for the 255 expressed regions not overlapping with known genes and having a normalized expression score equal or higher than 7.6 in some of the growing conditions.** Blue: biofilm samples. Orange: exponential phase samples. Yellow: stationary phase samples.


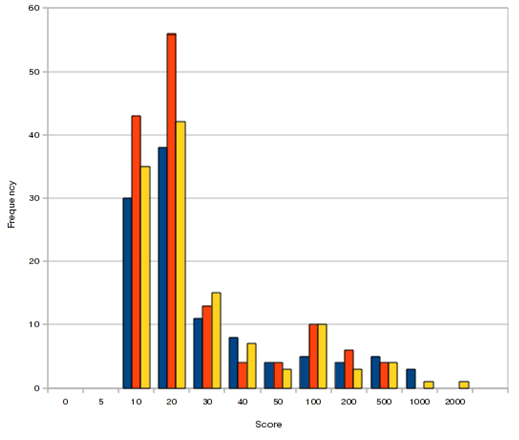

Supplement: S3 Fig — Blue: biofilm samples. Orange: exponential phase samples. Yellow: stationary phase samples. (DOCX) [file pone.0182084.s012.docx]
