# Supplementary material for: Global assessment of small RNAs reveals a non-coding transcript involved in biofilm formation and attachment in Acinetobacter baumannii ATCC 17978
Source: PLoS One. 2017 Aug 1;12(8):e0182084. doi: 10.1371/journal.pone.0182084 (PMC5538643; doi:10.1371/journal.pone.0182084)
Supplement: S4 Fig — Expression levels of 8 sRNA regions in planktonic and sessile cells determined by qRT-PCR using Taqman probes. Y axis represents the relative expression of the genes taking the housekeeping gene gyrB as value 1. (DOCX) [file pone.0182084.s013.docx]

**S4 Fig. qRT-PCR assays.** Expression levels of 8 sRNA regions in planktonic and sessile cells determined by qRT-PCR using Taqman probes. Y axis represents the relative expression of the genes taking the housekeeping gene *gyrB* as value 1.
