# Supplementary material for: Global assessment of small RNAs reveals a non-coding transcript involved in biofilm formation and attachment in Acinetobacter baumannii ATCC 17978
Source: PLoS One. 2017 Aug 1;12(8):e0182084. doi: 10.1371/journal.pone.0182084 (PMC5538643; doi:10.1371/journal.pone.0182084)

**S5 Fig. Biofilm formation at different times.** Staining at 12, 24 and 48 h of biofilm formation of *A. baumannii* ATCC 17978 (17978), *A. baumannii* ATCC 17978 Δ13573 (Δ13573), *A. baumannii* ATCC Δ13573 harbouring pETRA with sRNA 13575 (Δ13573 complemented), *A. baumannii* ATCC 17978 harbouring pETRA with sRNA 13575 (13573), and *A. baumannii* ATCC 17978 harbouring pETRA (17978 with empty pETRA).


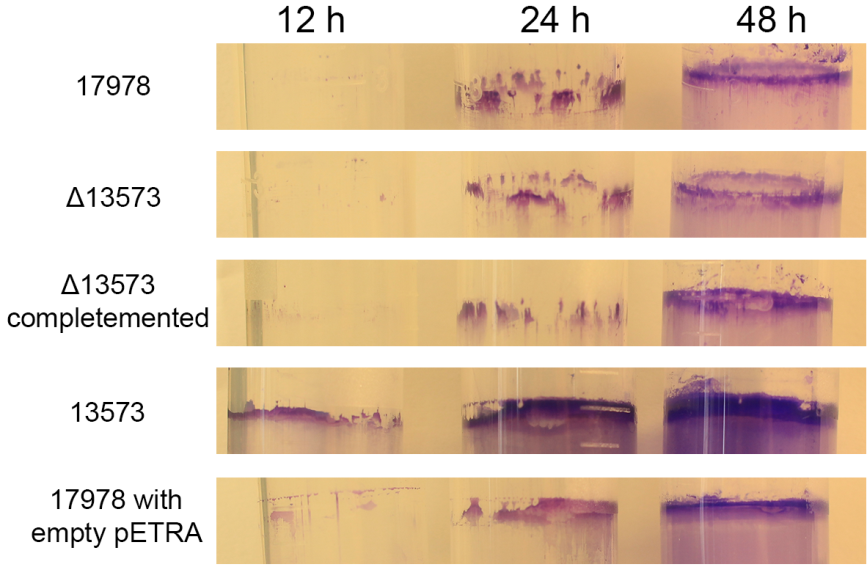

Supplement: S5 Fig — Staining at 12, 24 and 48 h of biofilm formation of A. baumannii ATCC 17978 (17978), A. baumannii ATCC 17978 Δ13573 (Δ13573), A. baumannii ATCC Δ13573 harbouring pETRA with sRNA 13575 (Δ13573 complemented), A. baumannii ATCC 17978 harbouring pETRA with sRNA 13575 (13573), and A. baumannii ATCC 17978 harbouring pETRA (17978 with empty pETRA). (DOCX) [file pone.0182084.s014.docx]
