# Supplementary material for: Global assessment of small RNAs reveals a non-coding transcript involved in biofilm formation and attachment in Acinetobacter baumannii ATCC 17978
Source: PLoS One. 2017 Aug 1;12(8):e0182084. doi: 10.1371/journal.pone.0182084 (PMC5538643; doi:10.1371/journal.pone.0182084)
Supplement: S6 Fig — Growth curves of wild type A. baumannii (17978), A. baumannii ATCC 17978 lacking the sRNA 13573 (Δ13573), A. baumannii Δ13573 over-expressing sRNA 13575 (Δ13573 complemented), A. baumannii ATCC 17978 harbouring pETRA over-expressing sRNA 13575 (13573) and A. baumannii ATCC 17978 harbouring the empty pETRA vector (17978 with empty pETRA). (DOCX) [file pone.0182084.s015.docx]

**S6 Fig.** **Growth curves.** Growth curves of wild type *A. baumannii* (17978), *A. baumannii* ATCC 17978 lacking the sRNA 13573 (Δ13573), *A. baumannii* Δ13573 over-expressing sRNA 13575 (Δ13573 complemented), *A. baumannii* ATCC 17978 harbouring pETRA over-expressing sRNA 13575 (13573) and *A. baumannii* ATCC 17978 harbouring the empty pETRA vector (17978 with empty pETRA).
